# Cranial anatomy of the mekosuchine crocodylian *Trilophosuchus rackhami* Willis, 1993

**DOI:** 10.1002/ar.25050

**Published:** 2022-08-29

**Authors:** Jorgo Ristevski, Vera Weisbecker, John D. Scanlon, Gilbert J. Price, Steven W. Salisbury

**Affiliations:** ^1^ School of Biological Sciences The University of Queensland Brisbane Queensland Australia; ^2^ College of Science and Engineering Flinders University Bedford Park South Australia Australia; ^3^ Phoenix Environmental Sciences Osborne Park Western Australia Australia; ^4^ School of Earth and Environmental Sciences The University of Queensland Brisbane Queensland Australia

**Keywords:** Crocodylia, Longirostres, Mekosuchinae, Mekosuchini, *Trilophosuchus*

## Abstract

One of the best‐preserved crocodylian fossil specimens from the Cenozoic of Australia is the holotype of the mekosuchine *Trilophosuchus rackhami*, from the middle Miocene (13.56 ± 0.67 Ma) Ringtail Site at Riversleigh, northwestern Queensland. Although lacking most of the snout, the holotype skull of *T. rackhami* (QMF16856) has an exceptionally well‐preserved cranium. Micro‐CT scanning of the holotype has allowed for all the preserved cranial bones to be digitally disarticulated, facilitating an unprecedented insight into the cranial anatomy of not just *T. rackhami*, but any mekosuchine. *Trilophosuchus rackhami* was a small‐bodied crocodylian and one of the most morphologically distinct mekosuchines, characterized by a unique combination of cranial characteristics several of which are exclusive to the species. Fossil material that is definitively referrable to the species *T. rackhami* is currently known solely from the middle Miocene Ringtail Site. However, an isolated parietal from Hiatus Site at Riversleigh demonstrates that *Trilophosuchus* also occurred during the late Oligocene (~25 Ma), extending the range of the genus by more than 10 million years. The new description of *T. rackhami* also allowed for a reevaluation of its phylogenetic relationships. Our results reaffirm the placement of *T. rackhami* as a member of Mekosuchinae within the subclade Mekosuchini. In all analyses, Mekosuchinae was consistently found to be monophyletic and part of the larger crocodylian clade Longirostres. However, the assignment of Mekosuchinae as a subset of Crocodylidae is brought into question, suggesting that the status of Mekosuchinae as a subfamily should be reconsidered.

## 1 INTRODUCTION

Present‐day Australia is home to only two crocodylian species from the globally distributed genus *Crocodylus* Laurenti, 1768—*Crocodylus johnstoni* (Krefft, 1873) and *Crocodylus porosus* Schneider, 1801. However, crocodylian diversity from the continent's prehistoric past was significantly greater. Most extinct Australian crocodylians come from Cenozoic deposits and belong to Mekosuchinae, with known taxa ranging from the early Eocene (e.g., Buchanan, 2009; Holt et al., 2005; Salisbury & Willis, 1996; Willis et al., 1993) to the late Pleistocene (e.g., Molnar, 1981; Ristevski et al., 2020a; Willis & Molnar, 1997). In addition to mekosuchines and *Crocodylus*, Australia also hosted gavialoid crocodylians during the Middle–Late Cenozoic (Lee & Yates, 2018; Megirian et al., 1991; Ristevski et al., 2021). Understanding the anatomy, diversity, and evolutionary relationships of crocodylians is dependent on the preservational condition and completeness of available fossil specimens. Many crocodylian fossil remains from Australia are fragmentary, and few taxa are represented by specimens that are of outstanding preservational quality. One such fossil specimen is the holotype for the focus taxon of this study—*Trilophosuchus rackhami* Willis, 1993, from the middle Miocene of the Riversleigh World Heritage Area, northwestern Queensland.

The Riversleigh World Heritage Area (henceforth abbreviated as WHA) is one of the most famous fossil localities from Australia, renowned for its taxonomic wealth and the remarkable preservational quality of the material derived from its numerous Cenozoic deposits (Archer et al., 2000). Although best known for its palaeodiversity of Middle–Late Cenozoic mammals, deposits from the Riversleigh WHA have also been important sources of reptile and bird fossils (Archer et al., 2006). Crocodylian remains are some of the most abundant reptile fossils from the Riversleigh WHA, with some taxa known from nowhere else. Almost all crocodylians from the Riversleigh WHA, including *T. rackhami*, belong to Mekosuchinae. Aside from mekosuchines, the Riversleigh area has also yielded the only reported fossil remains from the still extant *C. johnstoni* (Willis & Archer, 1990). At present, there are nine named mekosuchine species known from fossil sites at the Riversleigh WHA (Stein et al., 2016; Willis, 1993, 1997a, 2001; Willis & Molnar, 1997; Yates, 2017). These are known from material from late Oligocene deposits, such as the Low Lion Site, D Site Plateau (Stein et al., 2016), and the White Hunter Site (Willis, 1997a; see also Yates, 2017); the middle Miocene Ringtail Site (Willis, 1993, 2001; see also Yates, 2017); and the late Pleistocene Terrace Site (Willis & Molnar, 1997; see also Ristevski et al., 2020a).

The type locality of *T. rackhami* is Ringtail Site. First described and formally published by Willis (1993), *T. rackhami* has been one of the key taxa in defining the clade Mekosuchinae and its subclade Mekosuchini (see Salisbury & Willis, 1996). *Trilophosuchus rackhami* is the only named species of the genus *Trilophosuchus* Willis, 1993, and is best represented by its holotype QMF16856. Alongside *T. rackhami*, other mekosuchines recognized from material collected from the Ringtail Site include *Mekosuchus sanderi* Willis, 2001 as well as remains referrable to *Baru* Willis et al., 1990 (Archer et al., 2006; Willis, 2001; Willis et al., 1990; Yates, 2017).

**FIGURE 1:**
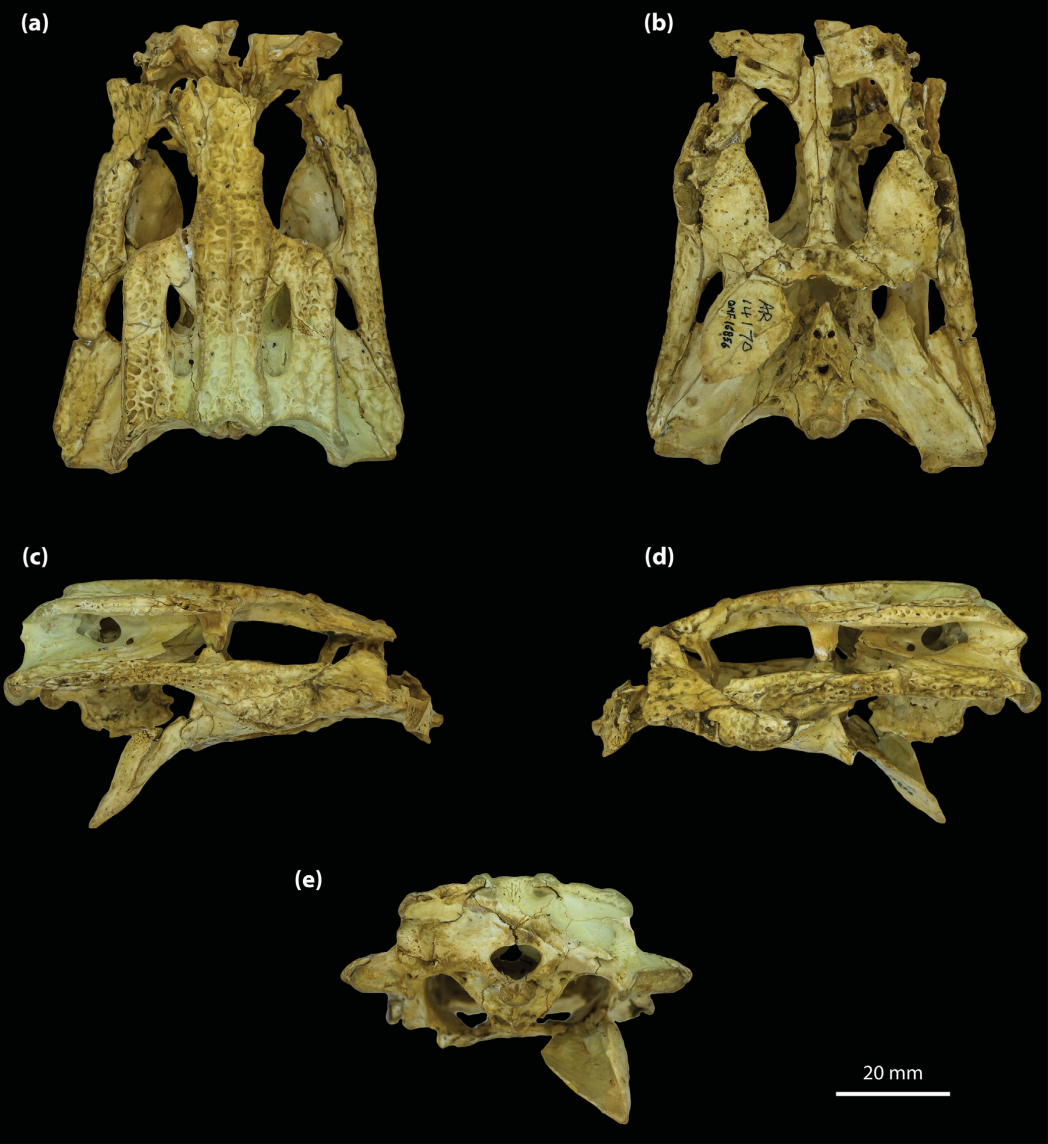
*Trilophosuchus rackhami* Willis, 1993, QMF16856, holotype. Cranium in (a) dorsal, (b) ventral, (c) right lateral, (d) left lateral, and (e) posterior views

Although the holotype of *T. rackhami* is missing much of the snout, the rest of the cranium is exceptionally well‐preserved (Figure 1). As such, QMF16856 is one of the most morphologically informative mekosuchine specimens ever discovered. Despite its extraordinary preservation, little to no attention has been devoted to the anatomy of this taxon since the original publication by Willis (1993). Therefore, the aim of this study is to provide an in‐depth description of its cranial osteology with the aid of high‐resolution μCT scan data. A detailed description of the neuroanatomy of QMF16856 is provided by Ristevski (2022). Based on the results from this study, we also reassess the phylogenetic relationships of *T. rackhami* and Mekosuchinae.

## 2 MATERIALS AND METHODS

### 2.1 Micro‐computed tomographic scanning and 3D digital models

The holotype of *T. rackhami* was subjected to micro‐computed tomographic (μCT) scanning. The specimen was scanned on the March 20, 2019, at the Centre for Advanced Imaging at The University of Queensland using a Siemens Inveon multimodality PET‐CT imaging scanner. The scan parameters were set to 0.05116 mm of slice thickness, 80 kV voltage, 500 μA current, and exposure time of 900 ms. The resulting dataset contains 2,344 slices in DICOM format. The images acquired from the μCT scan were imported into the specialized 3D image processing software Mimics (Materialize NV, Belgium) at The University of Queensland and Flinders University. Digital models of the cranial bones were generated in several versions of Mimics (Mimics 21.0, 22.0, and 24.0), where each cranial bone was manually isolated using several tools from the Segment menu (primarily the lasso tool in Multiple Slice Edit). Afterwards, the digital models of the cranial bones were exported as STL files. These STL files were then imported into Materialize 3‐matic 16.0 in order to create an interactive 3D PDF document of the *T. rackhami* holotype that is added as a supplement to this article.

The above‐mentioned 3D PDF, alongside the individual STL files of the isolated cranial bones can be accessed at the Dryad Digital Repository via the following link: https://doi.org/10.5061/dryad.gmsbcc2qm. Additional information can be found in Documents S1–S3 for this article. Remaining supplementary material can be freely accessed at Zenodo via: https://zenodo.org/record/6968350. The raw μCT data for QMF16856 is available upon request from the Queensland Museum at MorphoSource: https://www.morphosource.org/concern/media/000431912.

### 2.2 Mensuration

The external linear measurements of QMF16856 were taken directly using a hand‐held 150 mm digital caliper. Measurements of the digitally isolated bones were measured in Mimics 22.0 using the Distance tool from the Measure menu.

### 2.3 Body size estimates

In order to estimate the body size of the *T. rackhami* holotype individual, we used the regressions proposed by O'Brien et al. (2019). Based on data from extant crocodylians, O'Brien et al. (2019) developed regressions for estimating the total body length (TL), snout‐vent length (SVL), and body weight/mass (M) in crocodyliforms based on the trans‐quadrate width (TQW) of the skull. The TQW of the *T. rackhami* holotype is 58.5 mm, or 5.85 cm. We used the following regressions:

1. Regression for estimating TL: ln(TL) = 0.8024(ln(TQW)) + 3.05
2. Regression for estimating SVL: ln(SVL) = 0.768(ln(TQW)) + 2.525
3. Regression for estimating M: ln(M) = 2.953(ln(TQW)) − 4.785
4. Conservative regression for estimating M (on the basis that noncaptive living crocodylians may be 25% smaller than captive individuals; O'Brien et al., 2019): ln(M) = 2.953(ln(TQW)) − 5.072

The results from following the regressions by O'Brien et al. (2019) are:

1. TL of 87.1 cm
2. SVL of 48.5 cm
3. Body mass of 1.5 kg
4. Body mass (conservative estimate) of 1.2 kg

Based on these results, we estimate that the *T. rackhami* holotype individual had a TL between 70 and 90 cm, SVL between 35 and 50 cm, and a body mass between 1 and 2 kg.

### 2.4 Anatomical terminology

The anatomical terminology, abbreviations, and descriptive approach used herein follow those employed by Ristevski et al. (2020a, 2021). Recently, Kuzmin et al. (2021) provided a comprehensive assessment of the braincase anatomy in extant crocodylians. Thus, the braincase components of *T. rackhami* are described by applying the terminology proposed by Kuzmin et al. (2021). Cranial components not related to the braincase follow the terminology established by Kley et al. (2010). Virtually all anatomical terms used here are in English instead of standard Latin. Traditional directional terms are used throughout, such as “anterior” and “posterior” as opposed to “rostral” and “caudal.” Exempt from these directional phrases is the dental terminology (in the case of QMF16856 applicable only to the preserved maxillary alveoli), to which “mesial” and “distal” are applied instead of “anterior” and “posterior,” and “labial” and “lingual” as opposed to “lateral” and “medial” (following Hendrickx et al., 2015).

### 2.5 Phylogenetic analyses

The phylogenetic assessments undertaken in this study are based on an updated and expanded version of the character matrix by Ristevski et al. (2020a, 2020b, 2021). This version of the matrix has 257 morphological characters and 147 operational taxonomic units (OTUs). Out of the 257 morphological characters, *T. rackhami* could be scored for 110 (i.e., 42.8%) characters in total. Serving as an outgroup taxon was the goniopholidid crocodyliform *Anteophthalmosuchus epikrator* Ristevski et al., 2018. The phylogenetic analyses were carried out in TNT v1.5 Willi Hennig Society Edition (Goloboff et al., 2008; Goloboff & Catalano, 2016). Eight separate analyses were conducted with the new matrix. The first set of four analyses was performed by using the Traditional search option (TrS), while the other set of four analyses used the New Technology search option (NTS). In each set, one analysis was run under a “traditional” equal weighting (EW) principal search methodology, whereas the other three analyses used the implied weighting (IW) methodology (Goloboff, 1993). In the analyses that utilized the IW method, the *k* (concavity constant) values were set to 5.0 (*k* = 5), 12.0 (*k* = 12), and 25.0 (*k* = 25). In all analyses, 23 out of the 257 characters were treated as ordered (characters 21, 39, 49, 50, 54, 55, 82, 104, 118, 125, 142, 148, 149, 157, 159, 174, 200, 202, 221, 222, 239, 248, and 256). The program was set to 900 Mb of RAM, with the maximum number of held trees being 99,999.

For the analyses performed with the TrS option, the settings used one random seed and 1,000 replicates of Wagner trees, and the tree bisection reconnection (TBR) swapping algorithm saved 10 trees per replication. As for the analyses performed under the NTS option, the same search protocols as in Ristevski et al. (2020a, 2020b, 2021) were used here as well. The parameters applied in these analyses follow Young et al. (2016), which implement the new technology searches (sectorial search, ratchet, drift, and tree fusion) set to 1,000 random addition sequences (RAS). For the sectorial search, the selection size above 75 used 1,000 drifting cycles, 1,000 starts below 75 and trees were fused 1,000 times. Additionally, the consensus sectorial search (CSS) and exclusive sectorial search (XSS) were set to 1,000 rounds. For ratchet, the parameters were set to stop the perturbation phase when 1,000 substitutions were made, or 99% of the swapping was completed and a total of 1,000 iterations. For drift, the perturbation phase stopped when 1,000 substitutions were made, or 99% of the swapping was completed, and the number of cycles was set to 1,000. No changes were made to the tree fusion settings which were left at the default three rounds.

Nodal support was assessed by conducting Bremer support and bootstrap analyses in TNT v1.5. The Bremer support was performed by running the script “BREMER.RUN” and used the default settings. The bootstrap analysis (Efron, 1979; Felsenstein, 1985) was set to 1,000 replicates, showing values of 50% and above. Two homoplasy metrics, the consistency index (CI; Kluge & Farris, 1969) and retention index (RI; Farris, 1989), were calculated by running the script “STATS.RUN” in TNT v1.5. Additional information on the taxon matrix and character dataset are given in the Document S3 to this manuscript, with the raw data of the matrix (in NEXUS format) and results (in native .tnt formats) also provided as supplements.

### 2.6 Institutional abbreviations mentioned in the text

OUVC, Ohio University Vertebrate Collections, Athens, Ohio, USA; QM, Queensland Museum, Brisbane, Queensland, Australia (F, fossil).

## 3 SYSTEMATIC PALEONTOLOGY

MEKOSUCHINAE Willis et al., 1993.

MEKOSUCHINI Salisbury & Willis, 1996.

*TRILOPHOSUCHUS* Willis, 1993.

**Type species**—*Trilophosuchus rackhami* Willis, 1993.

**Generic diagnosis (updated from Willis,** **1993** **):** Small‐sized mekosuchine crocodylian characterized by the following unique combination of features (autapomorphies indicated with *): (1) altirostral snout with trapezoid cross‐section anterior to the orbits; (2) dorsolaterally oriented orbits; (3) a lateroventrally sloping cranial table morphology that is retained in post‐hatchling ontogeny*; (4) width to length ratio of the cranial table is ≤1.1; (5) supratemporal fenestra with an elliptical outline and an anteroposterior length that is more than twice the mediolateral width; (6) maxilla closely approaches the orbital margin, separated from the latter by a narrow contact formed by the lacrimal and jugal*; (7) maxillary foramen for the palatine ramus of the trigeminal nerve very large, with a diameter almost as large as some maxillary alveoli; (8) posterior tip of the nasal reaches to the same level as the anterior‐most margin of the orbit; (9) ascending process of the jugal (=base of postorbital bar) flush with the lateral surface of the jugal; (10) jugal with an ornamented ventral lamina; (11) medial surface of the jugal anterior to the postorbital bar covered almost completely by the maxilla and ectopterygoid; (12) dorsal surface of the cranial table with a prominent mid‐sagittal crest, extending over the frontal and parietal; (13) frontal with a relatively short anterior process that is ~28–29% of the total anteroposterior length of the element; (14) frontoparietal suture linear; (15) descending process of the parietal perforated by multiple foramina; (16) large pneumatic recess within the parietal; (17) cranial table with a pair of longitudinal and continuous parasagittal crests, extending over the lateral (=orbital) margins of the frontal, lateral (=supratemporal) margins of the parietal, medial margins of the anteromedial processes of the postorbitals, and the medial margins of the medial processes of the squamosals*; (18) descending process of the postorbital with a sub‐triangular cross‐section; (19) attachment area for the upper earlid musculature on the lateral surface of the squamosal is ornamented with sub‐circular pits so that only the ventrolateral rim remains smooth; (20) ectopterygoid with a large plate that projects medially within the suborbital fenestra; (21) ventral surface of the ectopterygoid plate bears a large and shallow sub‐circular concavity*; (22) ectopterygoid forms the lingual margins of the last three maxillary alveoli; (23) pterygoids fused medially; (24) anterior process of the pterygoid forms ~33% of the total length of the palatal bar; (25) large dorsal exposure of the supraoccipital that does not exclude the parietal from reaching the posterior margin of the cranial table; (26) the medial process of the squamosal contacts the dorsal lamina of the supraoccipital; (27) flat occipital lamina of the supraoccipital that is lacking a nuchal crest and concavities, but has a wrinkled texture*; (28) occipital surface of the basioccipital faces posteroventrally; (29) basioccipital plate bears a well‐developed crest; (30) ventral margin of the basioccipital, posterior to the median pharyngeal tube foramen, without a concave edge; (31) short sutural contact between the anteromedial process of the quadrate and the dorsal lamina of the supraoccipital; (32) condylar surface of the quadrate with a prominent dorsal peak situated close to the dorsal margin of the medial hemicondyle; (33) condylar surface of the quadrate with subparallel dorsal and ventral margins; (34) quadratosquamosal suture extends to the posteroventral corner of the external auditory meatus; (35) partially interlocking dentition, with one labially occluding dentary tooth between the fifth and ninth maxillary alveoli; (36) alveoli of the maxilla with circular to sub‐circular outlines.

*Trilophosuchus rackhami* Willis, 1993.

***Holotype***—QMF16856 (formerly AR 14170), mostly complete and well‐preserved cranium, missing a significant portion of the snout (Figures 1, 2, 3, 4, 5, 6, 7, 8, 9, 10, 11, 12, 13, 14, 15, 16, 17, 18, 19, 20, 21, 22, 23).

***Type locality and horizon***—Ringtail Site, northern Gag Plateau, Riversleigh Station, northwestern Queensland (Figure S1.1). Radiometric dating determined that the Ringtail Site is middle Miocene in age (maximum estimate of 14.2 Ma, late Langhian, and minimum estimate of 12.9 Ma, middle Serravallian; Woodhead et al., 2016).

***Referred specimens***—QMF16857 (formerly AR 8532), an isolated frontal, and QMF16858 (formerly AR 16980), an isolated right postorbital (Figure S2.1). Both from Ringtail Site, northern Gag Plateau, Riversleigh Station, northwestern Queensland.

***Specific diagnosis***: Because *Trilophosuchus rackhami* is currently the only recognized species within its genus, the generic and specific diagnoses are the same.

*Trilophosuchus* sp.

***Referred specimen***—QMF60374, an isolated parietal (Figure S2.3) from the Hiatus Site (Queensland Museum Locality 941), Riversleigh Station, northwestern Queensland. Hiatus A Local Fauna, late Oligocene age (middle Chattian ~25 Ma; Archer et al., 1997; Scanlon, 2006a, 2006b).

## 4 OSTEOLOGICAL DESCRIPTIONS OF THE *TRILOPHOSUCHUS RACKHAMI* HOLOTYPE SPECIMEN (QMF16856)

### 4.1 General remarks

**FIGURE 2:**
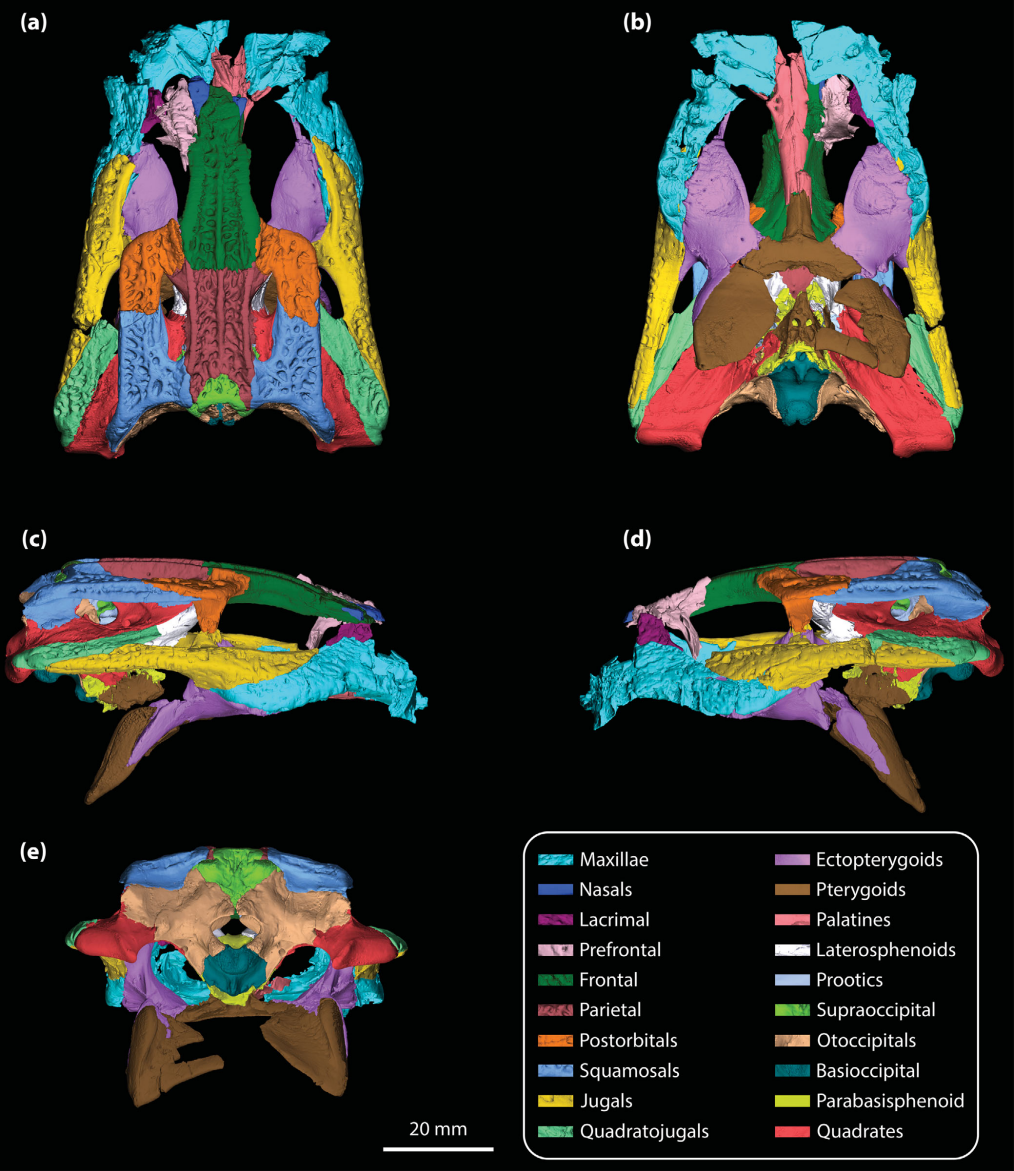
*Trilophosuchus rackhami* Willis, 1993, QMF16856, holotype. Digital model of the cranium in (a) dorsal, (b) ventral, (c) right lateral, (d) left lateral, and (e) posterior views

**FIGURE 3:**
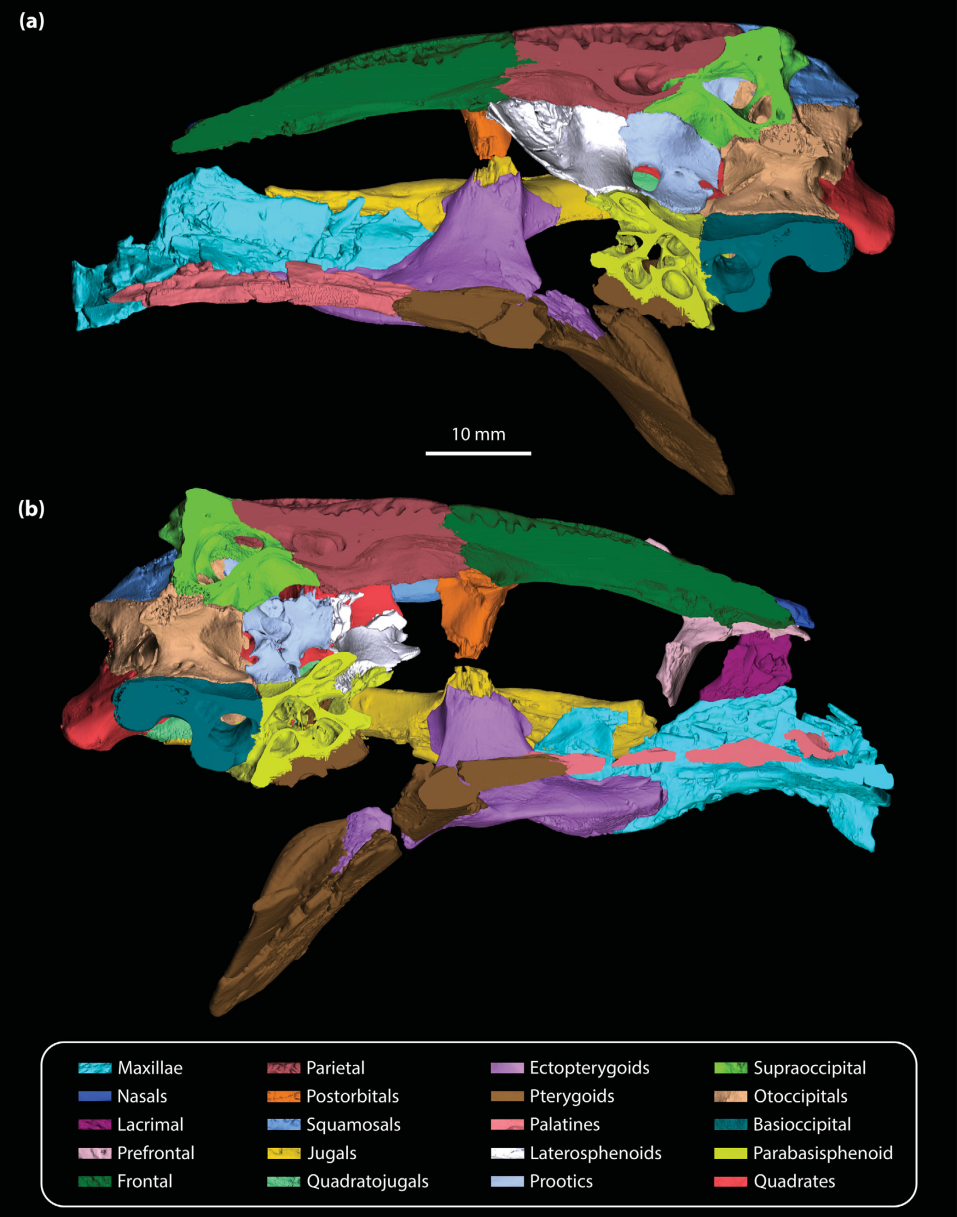
Sagittal sections of the digital model of the cranium of *Trilophosuchus rackhami* Willis, 1993, QMF16856, holotype. Sagittal section in (a) right medial and (b) left medial views

The skull of the *T. rackhami* holotype is incomplete as it is missing the snout (rostrum) almost in its entirety (Figures 1, 2, 3). In contrast to the snout, the rest of the skull is remarkably complete. According to Willis (1993, p. 96), the *T. rackhami* holotype was crushed post‐mortem and subsequent to its recovery it had to be subjected to substantial reconstruction. Regardless, the specimen in its current state appears in excellent preservational condition. When its overall completeness and preservational quality are accounted for, QMF16856 is one of the best‐preserved crocodylian fossil specimens yet discovered from Australia. There are however several broken and/or fractured portions on the braincase, palate, and periorbital region, and several nondescript fragments also accompany the holotype (Figure S1.16). Minor superficial cracks also affect some elements, although these have insignificant impact on interpreting the morphology of the bones. On the whole, the specimen appears to maintain the integrity of its three‐dimensional form quite well.

The skull of the holotype is relatively small, with its maximum preserved dimensions being ~79 mm in anteroposterior length, ~43 mm in dorsoventral height, and ~62 mm in mediolateral width. Although the snout is largely missing, what remains of it strongly indicates that it was relatively short and tall, with a trapezoidal cross‐section anterior to the orbits (i.e., altirostral snout). The relative shortness of the snout is best appreciated when the specimen is observed in dorsal and ventral views (Figures 1a,b and 2a,b), whereas the inferred altirostry and its sub‐trapezoidal cross‐section is most obvious in anterior view (Figure S1.2; see the interactive 3D PDF supplementary file). In dorsal view, the cranial table (as framed by the postorbitals and squamosals; Figures 1a and 2a) is sub‐rectangular and with parallel lateral margins (~35 mm in anteroposterior length, as measured from the frontal–postorbital suture anteriorly to the posterior margin of the medial process of the squamosal posteriorly; ~37 mm in mediolateral width, as measured at the level of the postorbital–squamosal sutures).

In total, the holotype of *T. rackhami* preserves 32 individual elements, 12 of which are paired (i.e., consisting of a left and right counterpart, for a total of 24: maxillae, nasals, postorbitals, squamosals, jugals, quadratojugals, ectopterygoids, palatines, laterosphenoids, prootics, otoccipitals, and quadrates) and six unpaired (frontal, parietal, pterygoid, supraoccipital, basioccipital, and parabasisphenoid). Lastly, two elements that were also paired in the complete skull—the lacrimal and the prefrontal—have only their left counterparts preserved. Out of all cranial elements, the only ones not preserved in the holotype (or any known specimen of *T. rackhami*) are the premaxillae, the vomers, the palpebrals, and the stapedes. Likewise, there are no known teeth, mandibular, or postcranial elements that can be attributed to *Trilophosuchus* at present.

### 4.2 Major cranial fenestrae, fossae, foramina, and nonpneumatic canals

#### 4.2.1 Orbits

Both orbits (=orbital fenestrae) are relatively well preserved (Figures 1a–d, 2a–d, 3, and 6). Although their margins are incomplete, what is preserved of them adequately depicts their outlines and dimensions. The right orbit has less complete margins than the left, as it is missing its anterior (due to the missing right lacrimal and prefrontal) and a small section of its posterior margin (Figures 1a and 2a). The left orbit is also slightly incomplete along its posterior margin for the same reason as the right orbit, which is due to breakage on both the left and right postorbital bars. The orbits are mainly oriented laterally and slightly dorsally, with a medial inclination of ~40° relative to the vertical plane. Based on the almost complete left orbit, the shape is sub‐oval with an anteroposterior length of ~24 mm and a dorsoventral height of ~18 mm. The entire orbital outline is relatively smooth and with a regular curvature that is devoid of constrictions and/or notches (character 138, state 0). The dorsomedial margin of each orbit is bounded by the frontal and prefrontal (with the prefrontal contributing anteromedially), anterior by the orbital lamina of the lacrimal, ventral/ventrolateral by the anterior process of the jugal, posterior by the postorbital bar (formed by the ascending process of the jugal ventrolaterally, ascending process of the ectopterygoid ventromedially, and descending process of the postorbital dorsally), and posterodorsal by the orbital lamina of the postorbital. Unlike *Mekosuchus* Balouet & Buffetaut, 1987, the maxillae of *T. rackhami* do not contribute to the orbital margins (Figure 6; see Section 4.3.1).

#### 4.2.2 Infratemporal fenestrae

The margins of both infratemporal (=laterotemporal) fenestrae are incomplete (Figures 1a–d and 2a–d), which hinders precise interpretation of their outlines. Of the two, the right infratemporal fenestra has better preserved margins (particularly its posterior and posterodorsal margins relative to the left). The anterodorsal margins are not preserved, and the breakage of the postorbital bars obscures the full extent of their anterior margins. The fenestrae are oriented dorsolaterally and have a medial inclination of ~22° relative to the vertical plane. The only margins of the infratemporal fenestrae that are sufficiently complete are the ventrolateral, with each being ~10 mm long. Coincidentally, the ventrolateral margin of the fenestra corresponds to its maximum anteroposterior length, which is 31% of the anteroposterior length of the cranial table (the latter being ~32 mm long, as measured in dorsal view of the skull from the anterior margin of the postorbital anteriorly to the posterior margin of the squamosal posteriorly). As preserved, the entire ventrolateral margin of each fenestra is formed by the posterior process of the jugal, anteroventral by the postorbital bar (comprised the ascending processes of the jugal and ectopterygoid), posterior by the posterior process of the jugal and ascending process of the quadratojugal, and posterodorsal by the ascending process of the quadratojugal. The sutural contact between the jugal and quadratojugal occurs at the posterior angle/margin of the infratemporal fenestra, so that both of these elements form the posterior fenestral margin (character 142, state 1; Figure 2a). As evident from the right infratemporal fenestra, its posterior margin is smoothly rounded and blunt.

#### 4.2.3 Suborbital fenestrae

The suborbital (=palatal) fenestrae (Figures 1b, 2b, and 14) are large openings on the palate, with the maximum anteroposterior length of each being ~30 mm. Their margins are imperfectly preserved, having suffered minor distortions and breakages. Regardless, the fenestrae's margins are almost complete, and their general contours can be interpreted accurately. The palatines form the entire anteromedial as well as most of the medial margins of the fenestrae. Comparatively smaller contributions to the fenestrae's margins come from the maxillae, which form their anterior and anterolateral margins. The ectopterygoids form a substantial part of the lateral and posterolateral margins, where the expansive ectopterygoid plates constrict the fenestrae laterally. Because of the incomplete preservational condition of the anterior processes of the ectopterygoids, a precise estimate of their contribution to the lateral margins of the fenestrae is not possible; however, it can be inferred that each ectopterygoid formed at least 21 mm, or 70%, of the lateral margin (character 182, state 1). Finally, the pterygoid bounds the suborbital fenestrae along their posterior and posteromedial margins. The suborbital fenestrae are longer than wide, but due to the lateral constriction from the ectopterygoids, the width of each fenestra is not constant. Thus, the suborbital fenestra is widest just anterior to the medial expansion of the ectopterygoid plate (~10 mm for the right fenestra; the left fenestra is missing its lateral margin at the point where it would have attained its maximum width) and narrowest at the point where the ectopterygoid plate projects furthest medially within the fenestra (~6 mm). Both the anterior and posterior margins of the fenestrae are rounded. The lateral margins of the suborbital fenestrae do not approach the lingual margins of the alveoli. The anterior‐most margins of the suborbital fenestrae extend to the level of the seventh (or possibly the sixth; see Section 4.6) maxillary alveoli (character 201, state 2).

#### 4.2.4 Supratemporal fenestrae

The supratemporal fenestrae (=dorsotemporal or upper temporal fenestrae; supratemporal foramina of Salisbury et al., 2003) are complete and with undistorted margins (Figures 1a and 2a). Each supratemporal fenestra has an elliptical outline (character 205, state 4) with a pointed anterior tip/margin (described as “almond‐shaped” by Willis, 1993, p. 93). The supratemporal fenestra is longer (~17 mm anteroposterior length) than wide (~6 mm transverse width), with its major axis oriented parasagittally. Thus, the length of the supratemporal fenestra is 53% of the length of the cranial table (the latter being ~32 mm long, as measured in dorsal view of the skull from the anterior margin of the postorbital anteriorly to the posterior margin of the squamosal posteriorly). The entire medial and posteromedial margins of the supratemporal fenestra are bounded by the parietal, posterior margin by the medial process of the squamosal, posterolateral, and part of the lateral margins by the anterior process of the squamosal, anterolateral margin by the posterior process of the postorbital, and anterior margin/tip by the anteromedial process of the postorbital. The anterior process of the squamosal comprises ~65% whereas the posterior process of the postorbital comprises ~35% of the lateral margin of each fenestra. None of the surrounding cranial table bones overhang the rims of the supratemporal fenestra (character 151, state 0). Because the lateral elements of the cranial table (i.e., postorbitals and squamosals) slope lateroventrally from the sagittal plane (character 155, state 0; Figures 1e, 2e, and S1.2a), the medial margins of the supratemporal fenestrae as formed by the parietal are on a more dorsal level relative to the lateral, anterior, and posterior margins. The anteromedial, anterolateral and lateral rims of the fenestrae are smooth, although the frontoparietal fossae (sensu Holliday et al., 2020) have no appreciable dorsal exposure on the cranial table (character 208, state 0).

#### 4.2.5 Supratemporal fossae

The supratemporal fossae (=dorsotemporal or upper temporal fossae) extend ventrally from the dorsal rims of the supratemporal fenestrae. Each supratemporal fossa is formed by the parietal, postorbital, squamosal, supraoccipital, and quadrate, although the degree of participation by each of these elements to the fossa is variable. The descending process of the parietal (=crista cranii parietalis; parietal descending lamina of Sertich & O'Connor, 2014; Figure S1.3) forms most of the medial wall of the fossa, the posteroventral wall is formed by the anteromedial process of the quadrate, and the posterolateral wall by the medial margin of anterior process of the squamosal. The supraoccipital has small exposure at the posteromedial wall of the fossa, ventral to the medial process of the squamosal (Figure 2a; see also Figure S1.3 and the interactive 3D PDF). Finally, the anterolateral wall of each fossa is formed by the medial margin of the posterior process of the postorbital. All walls of the supratemporal fossae comprised by the aforementioned bones are smooth. More details on the morphology and features of each element that forms the walls of the supratemporal fossae are given below. The supratemporal fossa is greatly expanded posteriorly such that its posterior and posteroventral walls/floor extend for a length of ~8 mm, or ~47% the anteroposterior length of the supratemporal fenestra. The ventral floor of the fossa is almost exclusively comprised by the anteromedial process of the quadrate (Figures 1a and 2a).

#### 4.2.6 External auditory meatus

The meatal chamber regions (sensu Montefeltro et al., 2016) are well preserved on both the left and right side, and the external auditory meatus (=otic aperture) is complete on both sides of the cranium. Each external auditory meatus has an outline reminiscent of the letter D, except that its posterior margin is gently bowed (Figures 1c,d and 2c,d; also, see the interactive 3D PDF). The squamosal bounds the entire dorsal and posterior margins of each external auditory meatus, whereas the quadrate bounds the anterior, ventral, and posteroventral margins. Following the terminology of Montefeltro et al. (2016), the margins of the external auditory meatus can be further categorized into three separate incisures. Most prominent is the semilunar otic incisure which delimits the anterior, anterodorsal and anteroventral margins of the meatus, with the incisure being primarily bound by the otic process of the quadrate. The semilunar otic incisure is deeply bowed anteriorly and forms the bowl of the D‐like outline of the meatus. The posteroventral margin is delimited by the incisure of the otic aperture of the cranioquadrate passage, which is bounded by the anterior margin of the posterodorsal process of the quadrate and the posterolateral descending lamina of the squamosal. The dual contribution to the incisure of the otic aperture of the cranioquadrate passage by the quadrate and squamosal is due to the quadratosquamosal suture being present at this point along with the posteroventral margin of the meatus (character 147, state 2; Figure 2c,d). Finally, the posterodorsal margin of the external auditory meatus is delimited by the dorsal otic incisure which is bounded by the posterolateral descending lamina of the squamosal. Each external auditory meatus has an anteroposterior length of ~8 mm (measured midway from the posterior to anterior margins) and a dorsoventral height of ~6 mm (measured from the incisure of the otic aperture of the cranioquadrate passage ventrally to the dorsal otic incisure dorsally). The subtympanic foramen (=preotic siphoneal foramen of Kley et al., 2010) is situated ~2 mm anterior to the external auditory meatus, and each foramen has a diameter of ~4 mm (Figures 22a and 23a).

#### 4.2.7 Secondary choana

Little is preserved of the secondary choana (sensu Witmer, 1995), with its anterior and small lengths of the lateral margins being the only remnants (Figures 1b, 2b and 13d). As in other eusuchians (Huxley, 1875), the choana's anterior margin is fully bounded by the pterygoid (character 121, state 1). The anterior choanal margin is relatively straight for most of its length, only gently curving at its lateral preserved ends (character 250, state 0). The preserved transverse length of the anterior margin is ~16 mm. Since the pterygoid is incomplete, the position of the secondary choana relative to the posterior‐most pterygoidal margin is unclear. However, the anterior margin of the choana is relatively close to the posterior‐most margins of the suborbital fenestrae (character 248, state 2), separated from the latter by a length of just ~3 mm. The anterior margin of the secondary choana has a smooth and faintly concave surface. Inferences on the orientation of the choana, or whether it had a septum, cannot be made due to its incompleteness.

#### 4.2.8 Cranioquadrate passages

The cranioquadrate passages are nonpneumatic canals that are enclosed by the quadrates laterally and otoccipitals medially (Figures 18, 19, 22, and 23c). They are tubular in shape, with a diameter of ~3.5 mm for most of their length. More posteriorly, each cranioquadrate passage becomes more compressed. The total length of each passage is ~12 mm. Anteriorly, the cranioquadrate passage opens within the meatal chamber at the posterior of the external auditory meatus. They exit posteriorly on the occiput through an opening formed by the otoccipitals and quadrates (Figures 1e and 2e).

#### 4.2.9 Temporal canals

The temporal canals are elongated nonpneumatic canals that, in life, provided passage for the temporoorbital arteries and veins (Kuzmin et al., 2021; Porter et al., 2016; Sedlmayr, 2002). A circular anterior temporal foramen (=temporo‐orbital foramen, temporal canal, or orbitotemporal passage of some authors), ~2 mm in diameter, opens posteriorly into each supratemporal fossa and exits posteriorly through the posttemporal fenestra (Figure S1.5). The temporal canals have a length of ~8 mm (right canal) and ~7 mm (left canal), and have a circular to sub‐circular cross‐section for the anterior half of their lengths. The posterior half of each canal is strongly compressed. Each canal is bound dorsally by the medial process of the squamosal, and ventrally by the prootic buttress, supraoccipital, otoccipital (a relatively small contribution), and the anteromedial process of the quadrate.

#### 4.2.10 Posttemporal fenestrae

The posttemporal fenestrae (Figures 1e, 2e and S1.5) are two relatively small openings found dorsally on the occiput, and both are completely preserved and undistorted. Each posttemporal fenestra is quite elongated and has a length of ~5 mm along its major axis but only ~0.6 mm along its minor axis. Its major axis is oriented largely horizontally, although the fenestra also displays a gentle lateroventral curvature. The postoccipital processes of the supraoccipital (sensu Kälin, 1933) bound much of the ventral margins of the fenestrae, whereas the otoccipitals form their lateroventral margins. Dorsally and dorsolaterally, the fenestrae are bound by the medial processes of the squamosals, dorsomedially by the supraoccipital, and smaller dorsomedial sections are also bound by the posterior processes of the parietal.

#### 4.2.11 Foramen magnum

The foramen magnum opens at the approximate center of the occiput (Figures 1e and 2e). The foramen has a sub‐oval outline, with the ventral margin having a more concave contour than the dorsal, whereas the lateral margins are smoothly rounded. The dimensions of the foramen magnum on the occiput are ~9 mm in transverse width and ~6 mm in dorsoventral height. The otoccipitals bound most of the foramen including the entire dorsal, lateral, and lateroventral margins, while the basioccipital comprises the foramen's ventromedial margin.

### 4.3 Dermatocranial bones

#### 4.3.1 Maxillae

**FIGURE 4:**
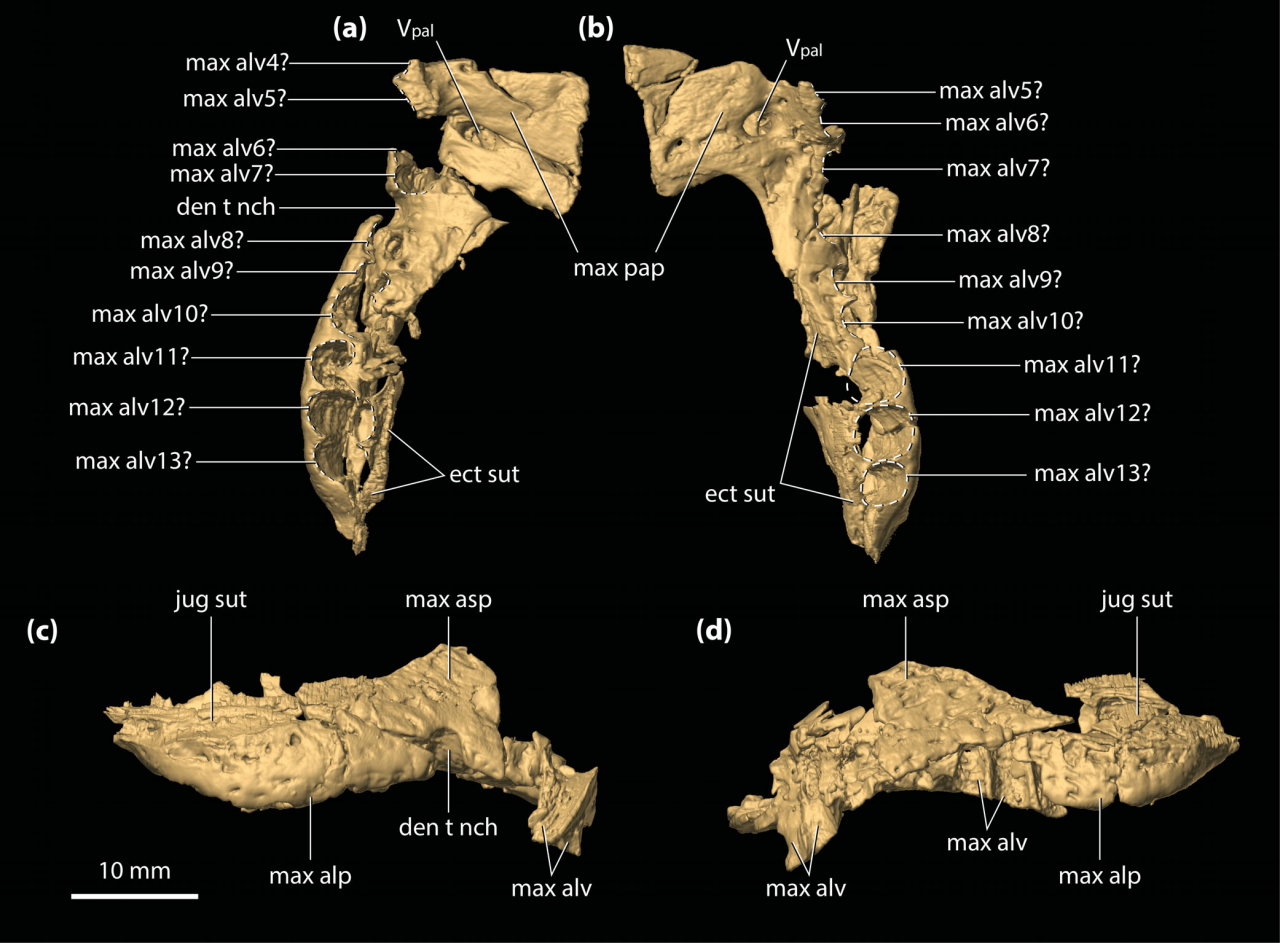
Maxillae of *Trilophosuchus rackhami* Willis, 1993, QMF16856, holotype. Right maxilla in (a) ventral, and (c) lateral views. Left maxilla in (b) ventral, and (d) lateral views. Note that both maxillae are incomplete. Also note that the lingual margins of the last three maxillary alveoli are bound by the ectopterygoid (see text for details). den t nch, notch for reception of a dentary tooth; ect sut, sutural surface for articulation with the ectopterygoid; jug sut, sutural surface for articulation with the jugal; max alp, alveolar process of the maxilla; max alv, maxillary alveolus (question marks denote the tentative interpretation of the number/position of the alveoli); max asp, ascending process of the maxilla; max pap, palatal process of the maxilla; V_pal_, maxillary foramen for the palatine branch of the trigeminal nerve

The maxillae are broken and are missing significant anterior sections (Figures 1, 2, 3, 4 and 6). Despite their incompleteness, what remains of the maxillae provides relevant information on their morphology and assists with inferring the cross‐sectional outline of the snout anterior to the orbits. As preserved, the maxillae are in sutural contact with the lacrimal (only the left maxilla, as the right lacrimal is missing), palatines, jugals, and ectopterygoids. The preserved lateral surfaces of the maxillae are lightly sculptured with sub‐circular pits that range from 0.2 to 0.8 mm in diameter, with the left maxilla having more pronounced ornamentation on its lateral surface when compared to the right. In contrast to the lateral, the ventral and medial surfaces of the maxillae are smooth and unornamented. Each maxilla preserves three processes—an alveolar process, a palatal process, and an ascending process.

The ventrally oriented maxillary alveolar process is comprised of two well‐developed and sub‐parallel laminae, a lateral (=labial) and a medial (=lingual) lamina, and together they bound most of the maxillary alveoli (see also Sections 4.3.11 and 4.6 below). It is along with the medial alveolar lamina of the maxilla that the contact with the ectopterygoid occurs. The alveolar processes of both maxillae are relatively poorly preserved, with their lateral laminae having suffered substantial breakage. Hence, the more anterior sections of the lateral laminae of the alveolar processes are missing. The external preserved surfaces of the lateral laminae bear conspicuous neurovascular foramina. Preserved on the right lateral alveolar process is a labially exposed reception pit for a dentary tooth, indicating that the dentary dentition partially interlocked with the maxillary (Figures 1c, 2c, and 4c; Willis, 1993; see Section 4.6). The medial alveolar process is most developed at the level of the (presumed) fifth maxillary alveolus. Posterior to the attained peak at the level of the fifth (?) alveolus, the medial lamina of the alveolar process becomes significantly reduced in height along the last 6–8 alveoli. Likewise, the lateral lamina is also reduced in height along the last alveoli, with the ventral margins of both laminae being on the same level relative to each other. When observed from a lateral aspect, each maxilla exhibits pronounced festooning, thus giving the maxillary alveolar process a strongly sinusoidal lateral outline (Figures 1c,d, 2c,d, 4c,d, and 6). As inferred on the more complete left side, the festoon of the alveolar process peaks at the level of the (presumed) fifth maxillary alveolus (which also corresponds to the tallest portion of the medial lamina of the alveolar process). After that, the festooned “wave” ascends to the level where the dentary reception pit is located (inferred from the right side). Afterwards, it begins to descend for a second time to form the subsequent festooned “wave”. The second “wave” peaks at the level intermediate to the penultimate alveolus and the alveolus anterior to it (presumed 11th and 12th alveoli), after which it starts to gently ascend again. In dorsal and ventral views, the maxillary festooning is also evident as a mediolaterally undulating line, although the damage on the lateral laminae makes the discerning of the festooning more obscure from these perspectives (Figures 1a,b, 2a,b, and 4a,b). No scalloping is recognizable between the alveoli along with the maxillary alveolar processes, at least not between the best‐preserved alveolar margins (the last five alveoli on the right side, and the last three alveoli on the left). The posterior‐most extent of the maxillae extends ventrally for the entire length of the anterior process of the jugal, terminating to a level that is ventral to the postorbital bar (see also Rio & Mannion, 2021).

The palatal processes of the maxillae (Figure 4a,b) formed part of the secondary bony palate. The relatively small sections that are preserved of the palatal processes have smooth ventral (=palatal) and dorsal (=internal) surfaces. Additionally, the ventral surface of each palatal process is relatively flat. The palatal processes are affected by minor fractures that are most evident at their anterior preserved sections. Medially, the palatal processes are in sutural contact with the anterior processes of the palatines, although the breakage present anterior to their contact with the palatines does not preserve the intermaxillary contact/suture. Each maxillary palatal process is pierced by an enlarged maxillary foramen for the palatine branch of the trigeminal nerve (character 103, state 1). These foramina are located ~3 mm lingual to the medial laminae of the maxillary alveolar processes, and ~3 mm anterolateral to the anterior margins of the suborbital fenestrae. The foramen on the left side (Figure 4b) is circular and has a diameter of ~2 mm, which is approximately 1 mm less than the estimated diameter of the presumed sixth alveolus on the left maxilla. The right foramen (Figure 4a) is more elliptical, having a length of ~3.7 mm along with its major axis and ~1.6 mm along its minor axis. As previously noted by Willis (1993), a shallow sulcus extends from the right maxillary foramen for the palatine branch of the trigeminal nerve that is spread in a medial and slightly posterior direction. An enlarged maxillary foramen for the palatine branch of the trigeminal nerve also occurs in some other mekosuchines (e.g., species of *Baru*, *Quinkana babarra* Willis & Mackness, 1996, *M. sanderi* and *M. whitehunterensis* Willis, 1997a; see Document S1 for detailed morphological comparisons).

**FIGURE 5:**
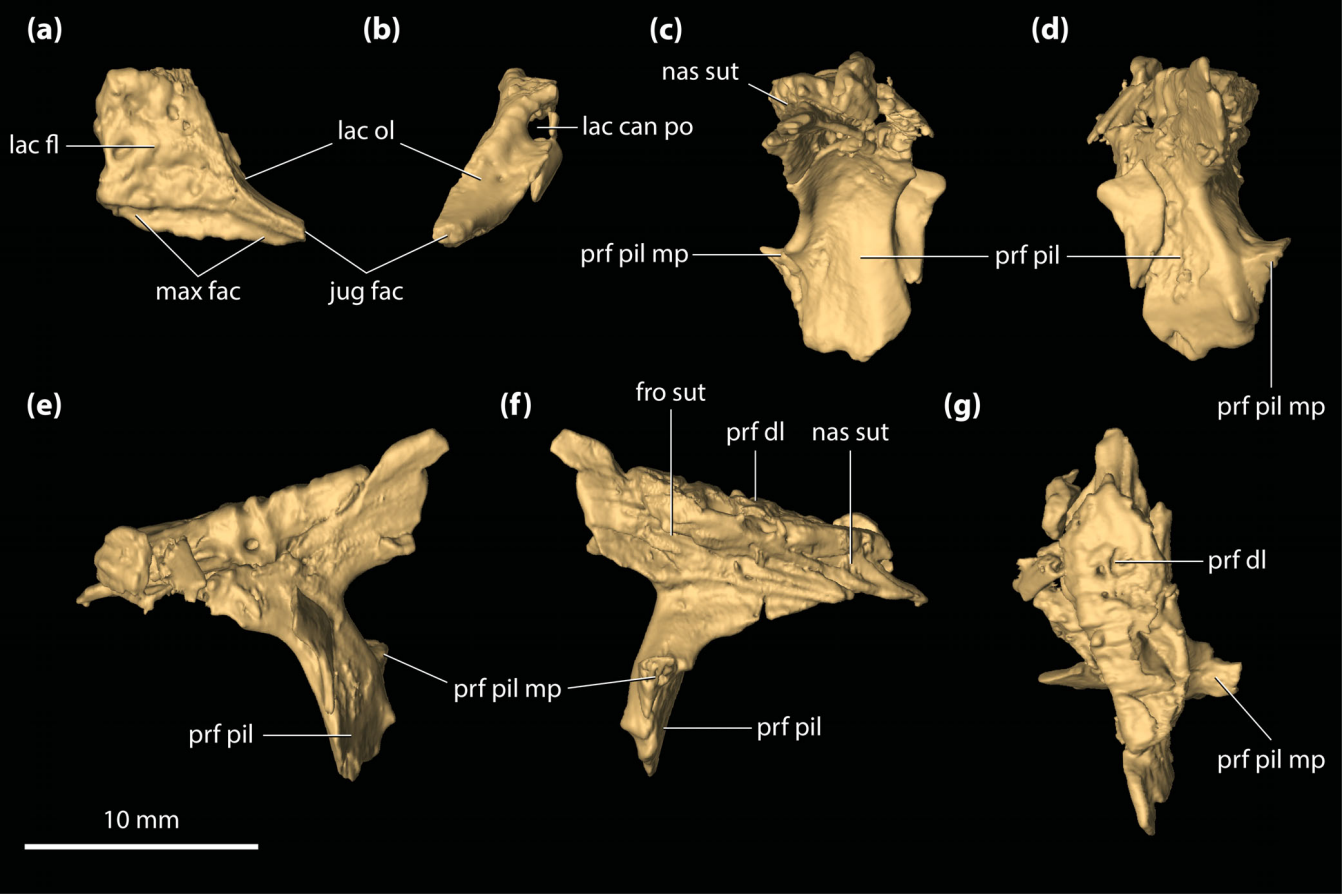
Left lacrimal and prefrontal of *Trilophosuchus rackhami* Willis, 1993, QMF16856, holotype. Left lacrimal in (a) lateral, and (b) posterior views. Left prefrontal in (c) anterior, (d) posterior, (e) lateral, (f) medial, and (g) dorsal views. Note that neither the left lacrimal or left prefrontal are complete. fro sut, sutural surface for articulation with the frontal; jug fac, jugal facet; lac can po, posterior opening of the lacrimal canal; lac fl, facial lamina of the lacrimal; lac ol, orbital lamina of the lacrimal; max fac, maxillary facet; nas sut, sutural surface for articulation with the nasal; prf dl, dorsal lamina of the prefrontal; prf pil, prefrontal pillar; prf pil mp, medial process of the prefrontal pillar

**FIGURE 6:**
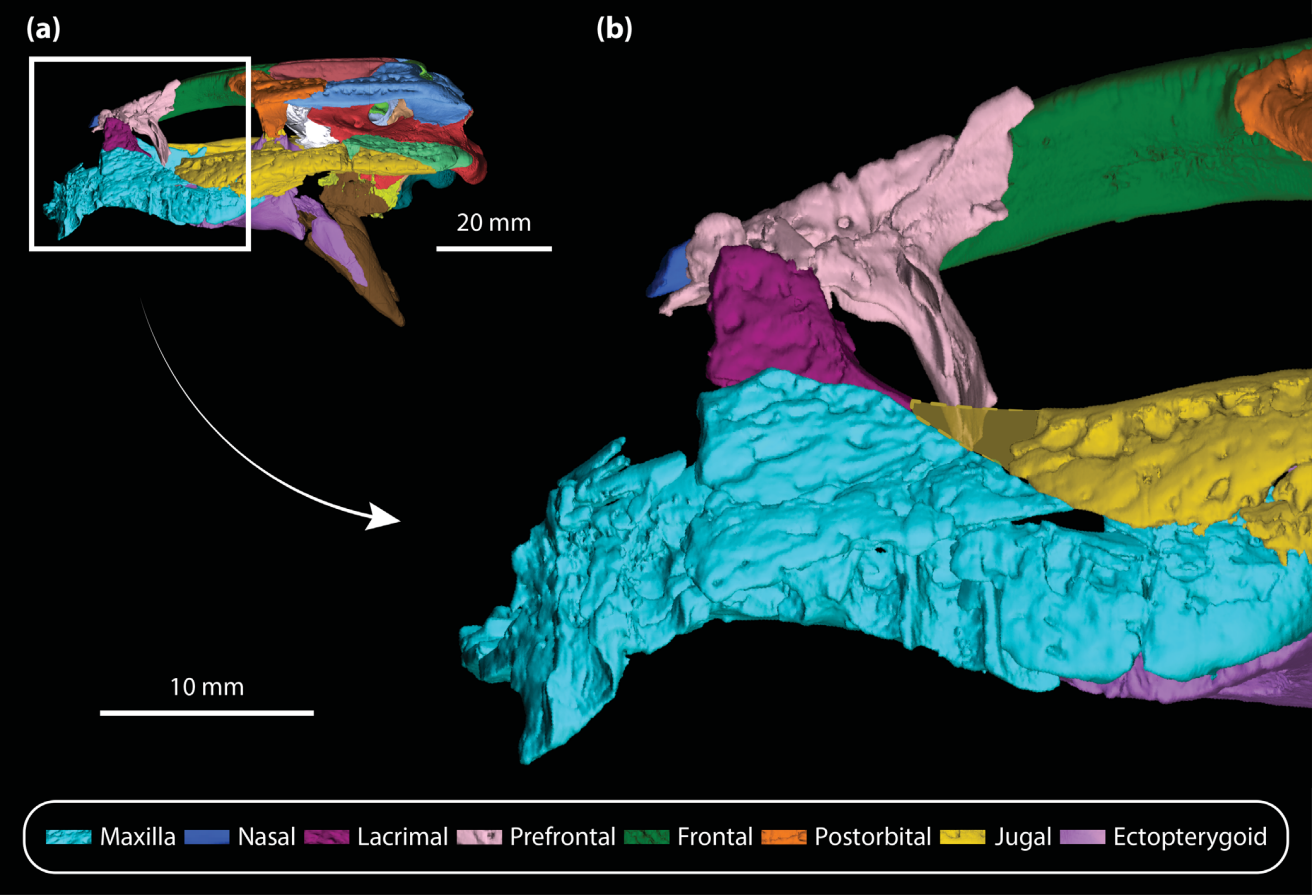
Interpretation of the contact between the left lacrimal and jugal in *Trilophosuchus rackhami* Willis, 1993, QMF16856, holotype. (a) Inset of the digital model of the cranium in left lateral view, with the white box highlighting the area shown in (b), which is a close‐up of the left preorbital region. In (b), the elements from the right side of the cranium are hidden in order to aid the visualization. The dashed yellow lines represent the reconstructed outline of the anterior process of the left jugal. The legend indicates the color codes only of the elements shown in (b). For the complete color code legend, see Figures 2 and 3

Relatively small posterior sections of the ascending processes of the maxillae are the only remains of these maxillary parts. The ascending process of the maxilla is in sutural contact with the anterior process of the jugal posteriorly, and with the lacrimal dorsally (with the only preserved lacrimal fragment being on the left side). The external (or in this case lateral) surface is largely verticalized. As stated above (see Section 4.2.1), the maxillae do not contribute to the orbital margins. However, the relationship between the maxilla, lacrimal and jugal around the orbit is unique to *T. rackhami*. Except for *Mekosuchus* where the maxillae form part of the orbital margins (character 202, state 2), other crocodylians have the maxillae excluded from contributing to the orbits by a wide contact formed between the lacrimals and jugals. An intermediate condition occurs in *T. rackhami*, where the maxilla is very narrowly separated from the orbit by the contact between the lacrimal and the jugal (character 202, state 1). On the left side, the anterior tip of the anterior process of the jugal is broken off, and so the contact between the jugal and lacrimal is not preserved in QMF16856. Fortunately, the posterior portion of the left lacrimal is sufficiently complete so that the lacrimal–jugal contact can be reconstructed (Figure 6; see also figure 23C in Appendix 2 of Rio & Mannion, 2021). As reconstructed, the lacrimal and jugal established a narrow contact that would have separated the maxilla from the orbit by a minimum of ~0.6 mm. This peculiar morphology was previously recognized by Lee and Yates (2018) in their phylogenetic character list and scoring of *T. rackhami* (character 38, state 1 in Appendix 1 of Lee & Yates, 2018) and Rio and Mannion (2021). Willis (1993) tentatively suggested that the maxillae of *T. rackhami* may contribute to the orbital margins, however, the relatively poor preservation of these elements prevented him from confirming this. Nevertheless, as evident from his reconstruction in figure 5C, Willis (1993) interpreted a relatively narrow lacrimal–jugal contact that excluded the maxilla from the orbit.

#### 4.3.2 Nasals

Meager portions of the nasals are preserved (Figures 1a–d, 2a–d, 3b, and 6), which deny a meaningful assessment of their morphology. Only the posterior processes of the nasals remain, with a slightly larger section of the left posterior process being preserved as compared to the right. The left posterior process tapers posteriorly and wedges between the prefrontal laterally and the anterior process of the frontal medially. Their preserved dorsoventral thickness is ~2 mm. As evident from the left nasal, its posterior process extends to the same level as the anterior‐most margin of the left orbit (character 191, state 0).

#### 4.3.3 Lacrimal

A small posterior section of the left lacrimal (Figures 2a–d, 3b, 5a,b, and 6) is the only preserved portion of this element. As preserved, the lacrimal contacts the maxilla ventrolaterally, and dorsomedially the left prefrontal. The preserved sutural contact between the maxilla and lacrimal is such that the posterior section of the maxilla overlays the posteroventral margin of the lacrimal. Moreover, as can be gleaned from the μCT data, the preserved lacrimal is not strongly sutured to the left maxilla, but rather contacts it along a relatively smooth facet. Posteriorly, the lacrimal would have made a point contact with the tip of the anterior process of the jugal.

The preserved fragment of the left lacrimal has an external surface, or facial lamina of the lacrimal, with a predominantly lateral orientation. This is a consequence of the altirostral snout morphology of *T. rackhami* which results in largely verticalized lateral surfaces of the maxilla and lacrimal. Faint ornamentation comprised by few relatively shallow sub‐circular pits adorns the preserved lateral surface of the lacrimal (Figure 5a). What remains of its medial surface is smooth and unornamented, although the medial surface is rather poorly preserved. The orbital lamina of the lacrimal is the best‐preserved portion of the bone. It has a smooth surface that forms part of the anterior and a small part of the anteroventral walls of the left orbit. Furthermore, the orbital lamina of the lacrimal has a concave surface that contributes to the contour of the orbit. For the most part, the orbital lamina has a relatively constant mediolateral width (~3 mm) but gets slightly narrower towards its posteroventral margin (~1 mm) near the contact with the jugal. A small foramen (~0.4 mm in diameter) pierces the orbital lamina and continues internally into the element, however, damage within the lacrimal prevents tracing of its canal. The posterior opening for the lacrimal canal (Figure 5b) is incomplete as it is missing a substantial portion of its mediodorsal margin. Nonetheless, the posterior opening of the lacrimal canal is conspicuous, and is situated near the dorsal edge of the orbital lamina. The relatively large posterior opening of the lacrimal canal appears to have had an elliptical outline with a length of ~2 mm along its major axis and a length of ~1 mm along its minor axis. Tracing of the lacrimal canal is not possible due to the relatively poor preservation and incompleteness of the lacrimal.

#### 4.3.4 Prefrontal

Only the left prefrontal (Figures 1a–d, 2a–d, 3b, 5c–g, and 6) is preserved. Although mostly complete, the prefrontal has sustained some damage, and small fragments remain adherent to its surface. Moreover, the prefrontal has experienced a slight displacement from its original position as it subtly slants anteroventrally. As preserved, the prefrontal maintains contact with the lacrimal anterolaterally, the fragment of the left nasal anteromedially, and the frontal posteromedially. It also forms the anteromedial margin of the orbit. In crocodylians, the prefrontals also tend to contact the palatines ventrally, however, this contact is not preserved in QMF16856 as the prefrontal is missing its ventral‐most portion. The maximum preserved dimensions of the left prefrontal are ~18.5 mm of anteroposterior length and ~13 mm in dorsoventral height. The prefrontal has two main components—a dorsal lamina (=dorsal surface) and a robust prefrontal pillar (descending process).

The dorsal lamina of the prefrontal is relatively flat and with a mediolateral width of ~4 mm. Due to its relatively small surface, the dorsal lamina is ornamented by only two or three subcircular pits (Figure 5g). There are no ridges or tubercles on the prefrontal (character 107, state 0). It appears that the prefrontal is incomplete dorsally and is missing a more anterior portion. Anteromedially, the posterior process of the left nasal wedges between the prefrontal and the anterior process of the frontal. Therefore, it is evident that the frontal and nasals widely separated the two prefrontals from contacting each other (character 129, state 0). Along its medial contact with the anterior process of the frontal, the prefrontal is gently curved in a posterolateral direction (in contrast to *M. sanderi* where the equivalent suture curves at an approximately 90° angle; see Figures S1.14a,b). Additionally, the posterior portion of the dorsal lamina is somewhat tapered. The contact with the lacrimal is poorly preserved as the two elements are in rather loose articulation, with several small fragments surrounding the sutural area between the two. The lateral margin of the prefrontal dorsal to the prefrontal pillar is unornamented and pierced by a singular small foramen that is ~0.5 mm in diameter (Figure 5e).

The robust prefrontal pillar extends ventrally from the approximate center of the prefrontal, and has a mild medial inclination. All surfaces of the prefrontal pillar are smooth and unornamented. In anterior view (Figure 5C), the prefrontal pillar has a mostly flat to subtly concave surface, whereas posteriorly (Figure 5d) the pillar is gently convex. The pillar has a mediolateral width that is equal to slightly greater than the width of the dorsal lamina (~5.5 mm), whereas it is quite narrow anteroposteriorly. The anteroposterior length of the prefrontal pillar is greatest along its dorsal portion (~7 mm; character 108, state 1), while ventrally, the pillar is progressively tapered until it attains its narrowest anteroposterior point ventrally (~2 mm). Internally, the prefrontal pillar does not house a pneumatic recess (character 110, state 0). Medially, the prefrontal pillar bears a process that appears to be mostly complete (Figure 5c–g); nevertheless, it is possible that some portions of the process are missing. As preserved, the medial process of the prefrontal pillar attains a sub‐triangular appearance both in anterior, posterior as well as medial views. Its greatest mediolateral and anteroposterior lengths occur along its dorsal margin (both being ~2 mm), after which it gradually decreases its dimensions until it becomes almost flush with the ventral preserved part of the pillar. The dorsal surface of the medial process is flat. Continuing dorsomedially from the medial process, the medial surface of the prefrontal pillar attains a lateral inclination.

#### 4.3.5 Frontal

**FIGURE 7:**
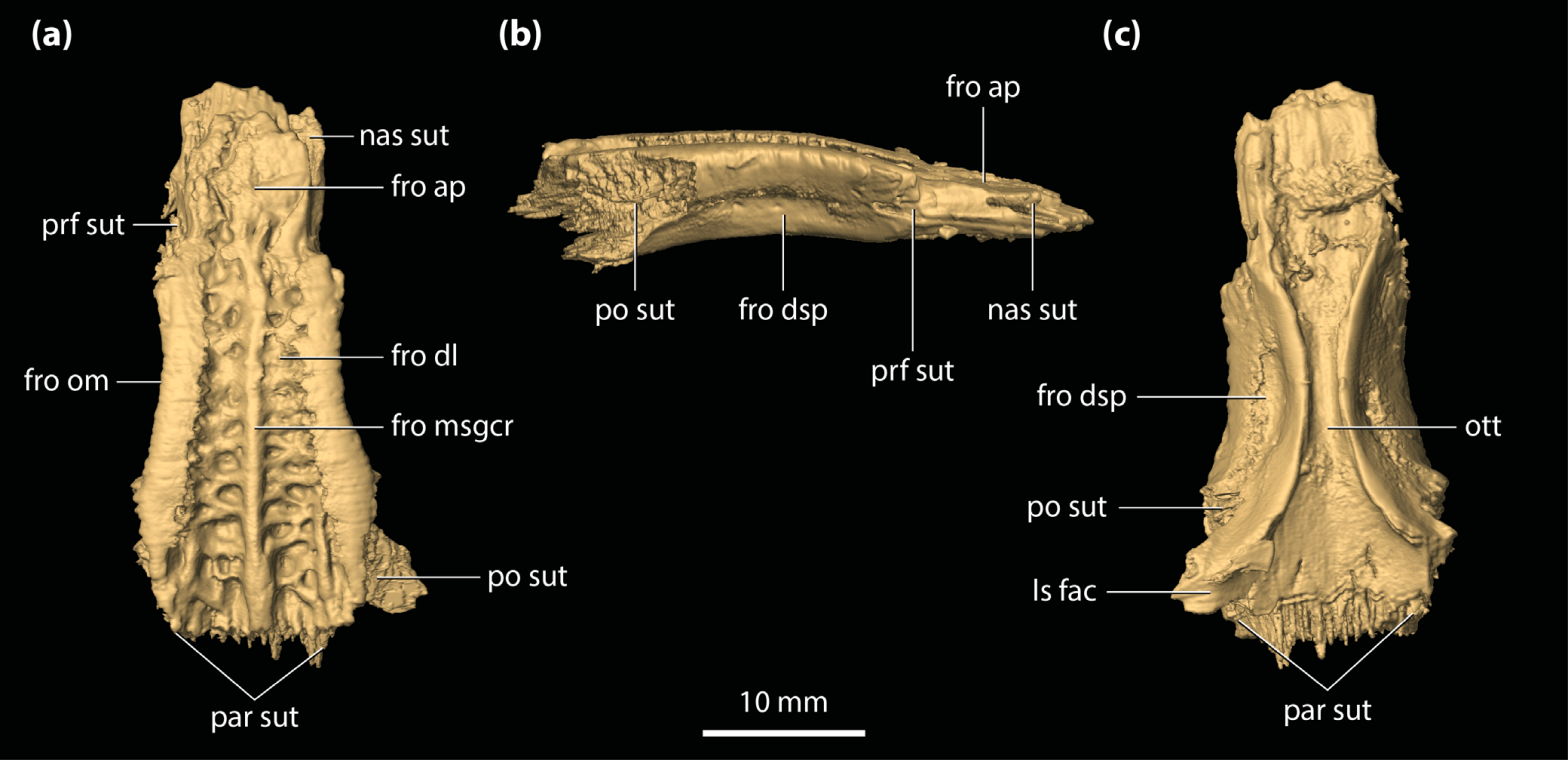
Frontal of *Trilophosuchus rackhami* Willis, 1993, QMF16856, holotype. Frontal in (a) dorsal, (b) right lateral, and (c) ventral views. fro ap, anterior process of the frontal; fro dl, dorsal lamina of the frontal; fro dsp, descending process of the frontal; fro msgcr, midsagittal crest on the frontal; fro om, orbital margin of the frontal; ls fac, laterosphenoid facet; nas sut, sutural surface for articulation with the nasal; ott, olfactory tract trough; par sut, sutural surface for articulation with the parietal; po sut, sutural surface for articulation with the postorbital; prf sut, sutural surface for articulation with the prefrontal

The frontal (Figures 1a–d, 2a–d, 3, 6, and 7) is almost complete (missing the facet for contact with laterosphenoid on the left side) and in a good preservational condition. The frontal of *T. rackhami* is an undivided element that represents a significant component of the skull, specifically the anterior portion of the cranial table. Anteriorly, the frontal would have contacted the nasals (since the nasals of QMF16856 are mostly missing, the frontal is now in anterolateral contact with their remaining fragments), anterolaterally the prefrontals, posterolaterally is in contact with the postorbitals, posteriorly with the parietal, and ventrally with the laterosphenoids (contact is preserved only with the right laterosphenoid). The frontal of *T. rackhami* primarily consists of a dorsal lamina (=dorsal surface), an anterior process, and a pair of descending processes. The maximum dorsoventral thickness of the bone is ~2 mm. Internally, the frontal does not host a pneumatic recess.

A hallmark on the dorsal lamina of the frontal are the three prominent crests (Figure 7a)—a midline sagittal (character 185, state 1) and two parasagittal crests. The crests stretch for almost the entire length of the cranial table and are found on the dorsal surfaces of the frontal, parietal, postorbitals, and to a lesser degree the squamosals. The total length of the midline sagittal crest is ~41 mm, whereas the parasagittal crests have a total length of ~48 mm. On the frontal, the midline sagittal crest has a length of ~24 mm, while the parasagittal crests stretch for ~21 mm. Therefore, the frontal hosts 59% of the total length of the midline sagittal crest and 44% of the parasagittal. The rest of the midline sagittal crest is located on the parietal (character 243, state 1), which holds 41% of the crests' length. The midline sagittal crest arises posteriorly to the anterior process of the frontal. Upon emergence, the midline sagittal crest is relatively low, but becomes more prominent as it extends posteriorly where it attains its maximum height (~1 mm) and width (~2 mm) on the frontal near the contact with the parietal. The midline sagittal crest is relatively straight for its entire length. The left and right parasagittal crests are positioned along the lateral margins of the frontal and thus comprise the orbital margins of the element. The parasagittal crests emerge immediately posterior to the prefrontals and have a width of ~2.5 mm for most of their lengths on the frontal. Their dorsal edges are blunt. The lateral edges of the parasagittal crests are concave as they follow the outlines of the dorsomedial margins of the orbits. Because of the parasagittal crests, the orbital margins of the frontal are upturned (character 137, state 1). This condition contrasts with the broadly convex orbital margins of the frontal seen in species of *Mekosuchus* (see figure S1.11 in Ristevski et al., 2020b).

Between the crests, the dorsal lamina of the frontal is ornamented with conspicuous circular to subcircular pits that are delimited by low ridges (Figure 7a). The interorbital surface is formed mostly by the frontal, with the minimum width of the bone between the orbits being ~11 mm. The frontal is widest posterolaterally, upon its contact with the anteromedial processes of the postorbitals where it attains a maximum width of ~15 mm. Posteriorly, the frontal contacts the parietal along a linear (character 150, state 1) and mildly serrate suture. Additionally, the frontal is excluded by the parietal and postorbitals from reaching the anterior margins of the supratemporal fenestrae (character 149, state 2; Figures 1a and 2a).

The anterior process of the frontal is laterally bordered by the prefrontals (of which only the left prefrontal remains) and anterolaterally by the posterior processes of the nasals. Anteriorly, it can be inferred that the anterior process of the frontal would have contacted the rest of the nasals as part of the anterior sutural surface for their contact is exposed at the anterior of the process. The dorsal lamina of the anterior process is ornamented with circular to subcircular pits that are comparatively shallower than those found posteriorly on the dorsal surface of the bone. Also, the midline sagittal crest does not extend over the anterior process of the frontal (Figure 7a). In QMF16856, the anterior process of the frontal is relatively short, having a length of ~10 mm which is 29% of the total anteroposterior length of the frontal (character 256, state 0). The anterior process is quite broad as well (~7 mm) and has a wide and blunt anterior tip (character 131, state 1). The anterior‐most margin of the anterior process of the frontal terminates slightly more posterior to the anterior tip of the prefrontal. The isolated frontal (QMF16857) that is referred to *T. rackhami* also displays a short anterior process that comprises ~28% of the total length of the element (Figures S2.1b–e).

The descending processes of the frontal (=cristae cranii frontales; frontal descending laminae of Sertich & O'Connor, 2014) are ventral extensions of the element. Both left and right descending process have markedly concave lateral surfaces and they bound the trough for the olfactory tract medially (Figure 7b,c). The descending processes are smooth and unornamented, as is the rest of the ventral surface of the frontal. Anteriorly, the descending processes are separated from each other by ~6 mm. As they continue further posteriorly, they quickly begin to close their distance, with the minimum length between them being ~2 mm. Posteriorly they diverge from each other yet again, with their maximum distance being ~9 mm. The posteroventral margin of the right descending process preserves the facet for contact with the anterior process of the right laterosphenoid.

#### 4.3.6 Parietal

**FIGURE 8:**
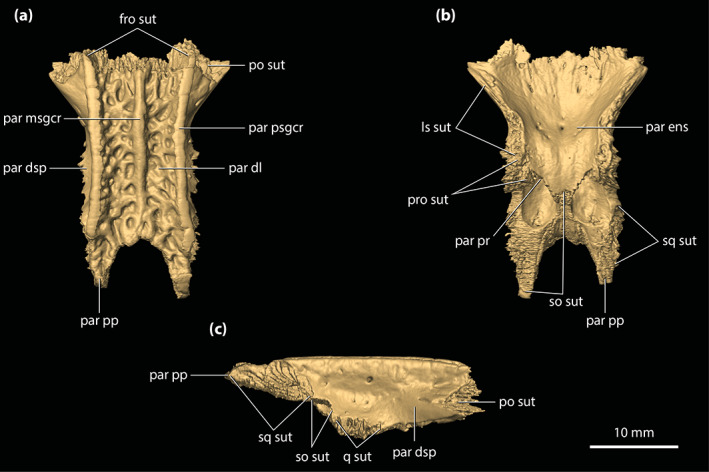
Parietal of *Trilophosuchus rackhami* Willis, 1993, QMF16856, holotype. Parietal in (a) dorsal, (b) ventral, and (c) right lateral views. fro sut, sutural surface for articulation with the frontal; ls sut, sutural surface for articulation with the laterosphenoid; par dl, dorsal lamina of the parietal; par dsp, descending process of the parietal; par ens, endocranial surface of the parietal; par msgcr, midsagittal crest on the parietal; par pp, posterior process of the parietal; par pr, parietal pneumatic recess; par psgcr, parasagittal crest on the parietal; po sut, sutural surface for articulation with the postorbital; pro sut, sutural surface for articulation with the prootic; q sut, sutural surface for articulation with the quadrate; so sut, sutural surface for articulation with the supraoccipital; sq sut, sutural surface for articulation with the squamosal

The parietal (Figures 1, 2, 3 and 8) is complete and well‐preserved. It is an unpaired element that forms a substantial portion of the cranial table and roof of the braincase. Anteriorly, the parietal contacts the frontal, anterolaterally the postorbitals, posterolaterally the squamosals, posteriorly and posteroventrally the supraoccipital, and ventrolaterally the laterosphenoids, the prootics, and the quadrates. The parietal consists of a dorsal lamina, two descending processes, an endocranial (or in this case ventral) surface, and two posterior processes. The maximum dorsoventral thickness/depth of the bone at its anterior contact with the frontal is ~8 mm. Internally, the parietal hosts a considerable pneumatic recess (character 165, state 0; Figure 3; for a description, see Ristevski, 2022).

Similar to the frontal, one of the most striking features on the parietal are the three crests that adorn its dorsal lamina (Figure 8a). The three crests are continuous from the frontal and extend for almost the entire length of the element. On the parietal, the midline sagittal crest is straight and has a length of ~17 mm, width (thickness) of ~1.5 mm, and height of ~2 mm. Posteriorly, the midline sagittal crest terminates just ~2 mm anterior to the sutural contact with the dorsal exposure of the supraoccipital. The two parasagittal crests bound the medial margins of the supratemporal fenestrae and stretch for the entire length of the parietal, to the exclusion of the parietal's posterior processes. The parasagittal crests continue from the parietal's dorsolateral margins over to the medial margins of the medial processes of the squamosals (Figure 10a,b). Both left and right parasagittal crest is relatively straight, bar some subtle medial curvature. On the parietal, the parasagittal crests have a length of ~22 mm, width of ~1 mm, and height of ~2 mm. As such, the parietal holds 44% of the total length of the parasagittal crests. The remaining 12% of their length is found on the medial processes of the squamosals. Between the crests, the dorsal lamina of the parietal has a surface that is heavily ornamented with conspicuous circular to subcircular pits delimited by low ridges. The lateral margins of the dorsal lamina are subparallel and without any, or only negligible lateral constriction. Hence, the parietal does not attain an hourglass‐appearance between the supratemporal fenestrae when observed in dorsal view (Figures 1a, 2a and 8a). The interfenestral bar (between the supratemporal fenestrae), which is formed by the parietal's dorsal lamina, has a width of ~11 mm and is therefore almost 30% of the total cranial table width (character 206, state 2).

Upon its contact with the dorsal exposure of the supraoccipital, the parietal continues to the posterior‐most margin of the cranial table in the form of two subparallel posterior processes (Figures 2a, e, 8, and S1.5). The posterior processes of the parietal are medially in contact with the dorsal exposure of the supraoccipital and laterally with the medial processes of the squamosals. Their posteroventral edges bound the dorsomedial margins of the posttemporal fenestrae. In dorsal view, the posterior processes gently taper towards the occiput. The dorsal surface of each posterior process is ornamented with few subcircular pits.

Extending ventrally from the dorsal lamina are the well‐developed descending processes (Figure 8a,c and S1.3). Both the left and right descending processes of the parietal are complete, except for minor damage on the left descending process at its posterior sutural contact with the supraoccipital and the quadrate. Along its anteroventral margin, the descending process bears a sutural surface/facet for contact with the laterosphenoid (this contact is preserved only on the right side as the left laterosphenoid is incomplete), along the ventromedial with the prootic (likewise, this contact is preserved only with the right prootic), and posteroventral with the supraoccipital and the quadrate. Each descending process has an unornamented and concave lateral surface. Anteriorly, at the contact between the parietal and the anteromedial process of the postorbital, the descending process of the parietal is convex and expanded anterolaterally. At the ventral margin of the anterior temporal foramen within the supratemporal fossa, the quadrate along with a small section of the supraoccipital prevent contact between the parietal and squamosal (character 154, state 0). Multiple small, yet conspicuous foramina perforate the descending processes of the parietal (character 153, state 1; Figure S1.3). Six foramina can be counted on the left descending process, all of which are concentrated relatively close to the dorsal margin of the supratemporal fenestra. The right descending process is pierced by 11 foramina, with six of them (including the largest) situated near the dorsal margin of the supratemporal fenestra whereas the rest are found further ventral relative to the fenestra's dorsal rim. These sub‐circular (and few sub‐elliptical) foramina range in size from 0.2 to 0.7 mm in diameter.

The endocranial surface of the parietal (Figure 8b) overlays a significant portion of the endocranial cavity. Its anterior half is deeply concave and pierced by several small foramina. Approximately mid‐length, the endocranial surface becomes convex as the parietal attains its greatest dorsoventral depth due to the spacious pneumatic recess inside. The more anterior section of the element, where the endocranial surface is concave, is not pneumatized by the parietal recess. More posteriorly, the endocranial surface of the parietal contacts the supraoccipital by a W‐shaped suture. Two large communicating foramina between the parietal recess and the intertympanic recess open ventrally on the parietal at the elements' contact with the supraoccipital (Figure 8b). A scarf joint sutural facet that articulates with the dorsal lamina of the supraoccipital separates the left and right foramen (see Section 4.4.3).

#### 4.3.7 Postorbitals

**FIGURE 9:**
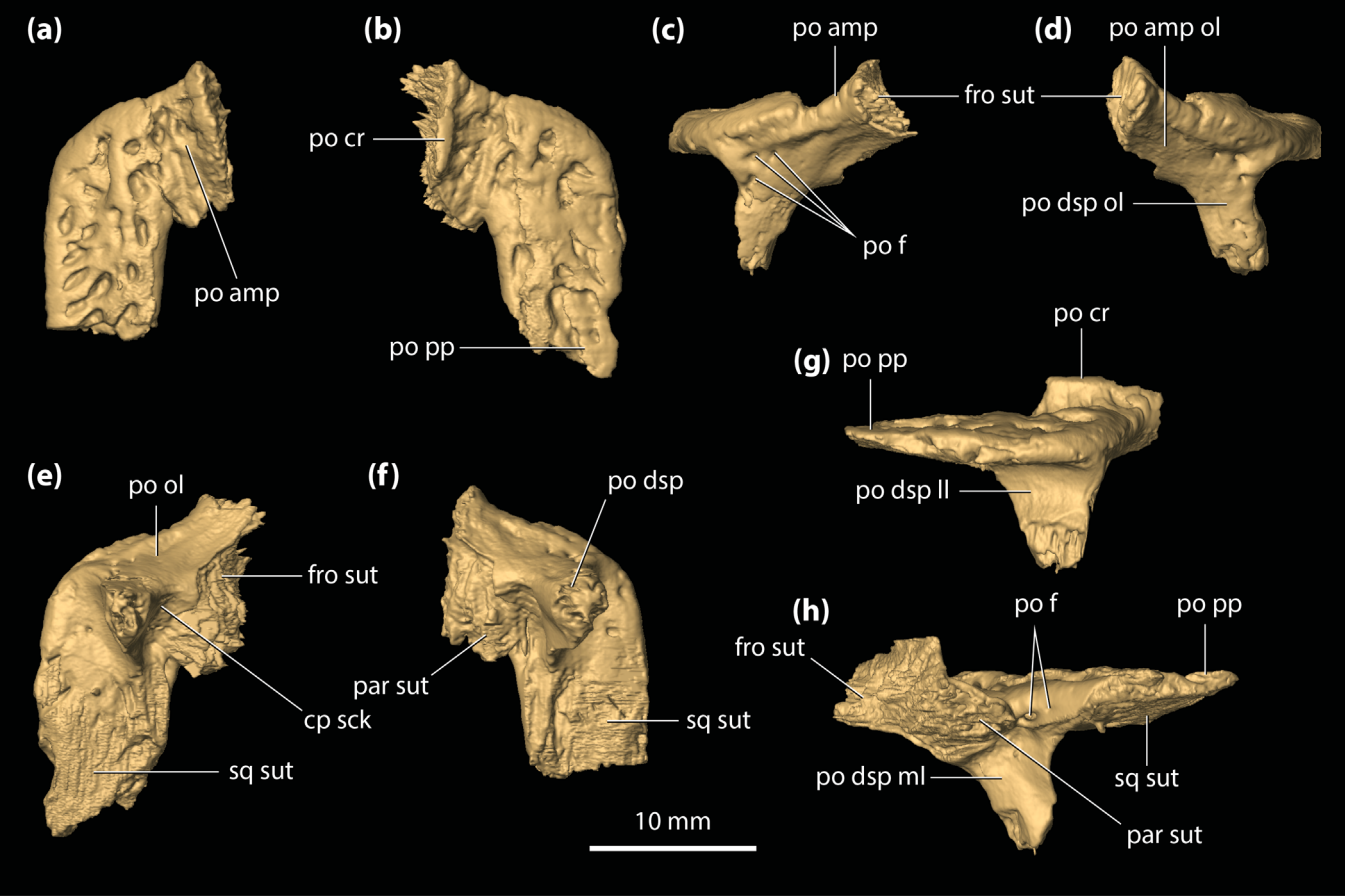
Postorbitals of *Trilophosuchus rackhami* Willis, 1993, QMF16856, holotype. Left postorbital in (a) dorsal, (d) anterior, and (f) ventral views. Right postorbital in (b) dorsal, (c) anterior, (e) ventral, (g) lateral, and (h) oblique medial views. Note that the posterior process of the left postorbital is incomplete. cp sck, socket for the capitate process of the laterosphenoid; fro sut, sutural surface for articulation with the frontal; par sut, sutural surface for articulation with the parietal; po amp, anteromedial process of the postorbital; po amp ol, orbital lamina of the anteromedial process of the postorbital; po cr, postorbital crest (part of the parasagittal crest on the cranial table); po dsp, descending process of the postorbital; po dsp ll, lateral lamina of the descending process of the postorbital; po dsp ml, medial lamina of the descending process of the postorbital; po dsp ol, orbital lamina of the descending process of the postorbital; po f, vascular foramina on the postorbital; po ol, orbital lamina of the postorbital; po pp, posterior process of the postorbital; sq sut, sutural surface for articulation with the squamosal

Comprising the anterolateral portions of the cranial table are the postorbitals (Figures 1a–d, 2a–d, 3, and 9). The postorbitals are well‐preserved and virtually complete. They are triradiate elements that can be subdivided into three main components, which are the anteromedial process, posterior process, and descending process. Each postorbital preserves its contact with the frontal and parietal anteromedially, and squamosal posteriorly. The sutural contact between the right postorbital, frontal and parietal is well‐preserved, whereas the left postorbital is slightly disarticulated from its anteromedially neighboring elements. Ventrally, the descending process of the postorbital was in contact with the ascending process of the jugal, although breakage did not preserve this contact on either postorbital in the specimen. The postorbitals do not contact the quadrates, but it is unknown if they had established contact with the quadratojugals. In dorsal aspect, the lateral and medial margins of the postorbital are parallel with each other. When the cranium is observed in anterior view, the postorbitals gently slope lateroventrally (Figure 9c,d and S1.2a; see also the interactive 3D PDF). Dorsally, the postorbitals are heavily ornamented with pits, whereas their ventral and anterior surfaces, and the descending processes are smooth. The maximum anteroposterior length of the (more complete) right postorbital is ~20 mm, and maximum mediolateral width is ~10 mm.

The relatively short anteromedial process of the postorbital extends from the posterior process at an angle of approximately 90°. Medially, much of the anterior portion of the anteromedial process sutures to the frontal, while more posteromedially it sutures to the parietal. The posterior margin of each anteromedial process bounds the tip of the supratemporal fenestra. The anteromedial edge of the process that sutures to the frontal and parietal bears part of the parasagittal crest of the cranial table. However, the parasagittal crest located on the postorbital is shared between it, and the frontal and parietal. The anteriorly facing orbital lamina of the anteromedial process of the postorbital is relatively broad. The orbital lamina of the anteromedial process has a smooth surface that is pierced by several conspicuous vascular foramina concentrated toward the lateral edge of the orbital surface.

The descending process of the postorbital is a sub‐vertical component of the bone that formed the dorsal half of the postorbital bar. Relative to the anterolateral margin of the cranial table, the descending process of the postorbital is deeply inset (character 136, state 1). When the postorbital is observed in anterior view, the descending process has a mild but noticeable medial inclination (Figure 9c,d and S1.2a). Three surfaces/laminae may be recognized on each descending process—an anterior or orbital lamina, a lateral, and a medial lamina. Anteriorly, the descending process of each postorbital is characterized by its smooth orbital lamina that is dorsally continuous with the orbital lamina of the anteromedial process of the postorbital. The cross‐section of the descending process is sub‐triangular, as the lateral and medial laminae converge posteriorly to render a posterior surface that is essentially a thin edge.

The posterior process of each postorbital is dorsoventrally compressed. Like the rest of the dorsal surface of the element, the posterior process of the postorbital is also ornamented with sub‐circular pits. Ventrally, almost the entire surface of the posterior process is in contact with the anterior process of the squamosal. The posterior process of the postorbital overlays the squamosal by forming a scarf‐joint suture. As evident from the better preserved right postorbital, the posterior process has a diagonal margin (Figures 2a and 9b).

#### 4.3.8 Squamosals

**FIGURE 10:**
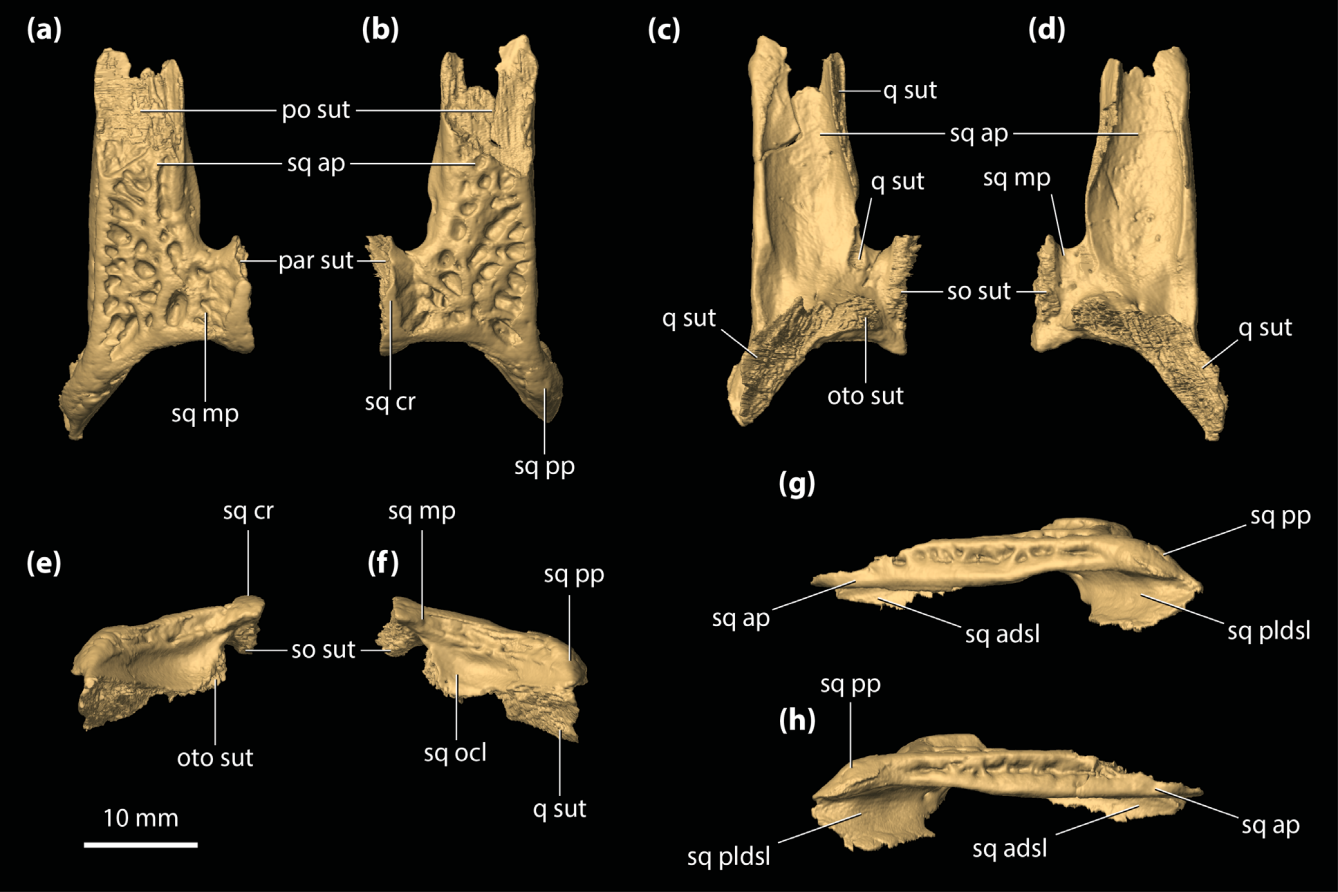
Squamosals of *Trilophosuchus rackhami* Willis, 1993, QMF16856, holotype. Left squamosal in (a) dorsal, (d) ventral, (e) posterior, and (g) lateral views. Right squamosal in (b) dorsal, (c) ventral, (f) posterior, and (h) lateral views. oto sut, sutural surface for articulation with the otoccipital; par sut, sutural surface for articulation with the parietal; po sut, sutural surface for articulation with the postorbital; q sut, sutural surface for articulation with the quadrate; so sut, sutural surface for articulation with the supraoccipital; sq adsl, anterior descending lamina of the squamosal; sq ap, anterior process of the squamosal; sq cr, crest on the medial process of the squamosal (part of the parasagittal crest on the cranial table); sq mp, medial process of the squamosal; sq ocl, occipital lamina of the squamosal; sq pldsl, posterolateral descending lamina of the squamosal; sq pp, posterior process of the squamosal

The squamosals are virtually complete (Figures 1, 2, 3 and 10). Each squamosal contacts the postorbital anteriorly, parietal posteromedially, supraoccipital ventromedially, otoccipital posteroventrally, and quadrate ventrally. The squamosals are triradiate elements, as each can be subdivided into three main processes: an anterior, a medial, and a posterior process. In addition to these three processes, three descending laminae may also be recognized: an anterior descending, a posterolateral descending, and an occipital lamina. The heavily ornamented dorsal lamina of the squamosal is comprised of its anterior and medial processes, and forms the posterolateral portion of the cranial table. In dorsal aspect, the lateral and medial margins of the squamosal are parallel. This gives a “straight” appearance of the squamosal in dorsal view for most of its length and contributes significantly to the parallel lateral margins of the cranial table (Figures 1a and 2a). The “straight” appearance of the element in dorsal aspect is interrupted posterolaterally as the posterior process of the squamosal diverges laterally. In occipital view of the cranium, the squamosals slope ventrolaterally from the sagittal plane (Figures 1e, 2e, and 10e,f). The maximum anteroposterior length of each squamosal is ~34 mm (measured in dorsal view, from the anterior tip of the anterior process to the posterior tip of the posterior process). Ventrally, the squamosals possess smooth, unornamented surfaces (Figure 10c,d).

The anterior process of the squamosal is quite long (~18 mm) and comprises 53% of the total anteroposterior length of the element. More than half (~61%) of its dorsal surface is a sutural facet for articulation with the postorbital anteriorly. The anterior process of the right squamosal is slightly damaged due to a crack that affects its portion for contact with the adjacent postorbital (Figure 10b,c). Its contact with the postorbital is a scarf joint sutural facet, with the posterior process of the postorbital overlaying much (for a length of ~11 mm) of the anterior section of the anterior process of the squamosal. When the squamosal and postorbital are observed in lateral view, their dorsal and ventral edges are sub‐parallel to each other (character 146, state 0). The dorsoventrally short anterior descending lamina projects ventromedially from the anterior process of the squamosal to establish contact with the anterodorsal process of the quadrate. The anterior descending lamina is smooth and unornamented and has a mildly concave lateral surface. The lateral margin of the anterior process is the location where the musculature for the upper earlid attached. This area lacks a well‐defined sulcus. Instead, most of this lateral margin is sculptured with conspicuous sub‐circular pits that leaves only the ventrolateral rim smooth and unornamented (character 210, state 1).

The medial process of the squamosal forms the entire postfenestral bar and bounds the posterior margin of the supratemporal fenestra. Its anteroposterior length (~8 mm) is ~22% of the cranial table width as measured at the level of the postorbital–squamosal sutures (~37 mm; character 207, state 0). When the squamosal is observed in dorsal view (Figures 1a, 2a, and 10a,b), the anterior and medial processes are virtually at 90° relative to each other. Medially, the process is in sutural contact with the parietal, whereas its ventromedial margin contacts the supraoccipital. The posteroventral margin of the medial squamosal process bounds the dorsal margin of the posttemporal fenestra. The posterior‐most extent of the parasagittal crests is found on the dorsomedial margins of the medial processes of the squamosals, where they stretch for a length of ~6 mm.

The posterior process of the squamosal (=posterolateral prong of the squamosal, or posterolateral ramus of the squamosal of some authors; e.g., Brochu, 1999; Rio & Mannion, 2021) is an elongated (~6 mm) and unornamented extension that, in dorsal view, projects posterolaterally from the element. In lateral view, the posterior process has a flat dorsal margin that lacks a horn‐like projection (character 156, state 0), but also gradually descends ventrally at an angle of ~144° (character 211, state 0). The posterolateral descending lamina of the squamosal contacts the posterodorsal process of the quadrate. The posterolateral descending lamina is exposed only in lateral view and has a concave and smooth lateral surface that partially bounds the posterior margin of the external auditory meatus. Anterodorsally, near the dorsal otic incisure, the right posterodorsal lamina is pierced by two foramina while the lamina on the left is pierced by a single foramen. In occipital view (Figures 1e, 2e, and 10e,f), the squamosal forms another descending projection herein termed the occipital lamina. The occipital lamina of each squamosal is fully exposed dorsolaterally on the occiput, and has a smooth and concave surface. Ventrally, the occipital lamina of the squamosal sutures to the paroccipital process of the otoccipital.

#### 4.3.9 Jugals

**FIGURE 11:**
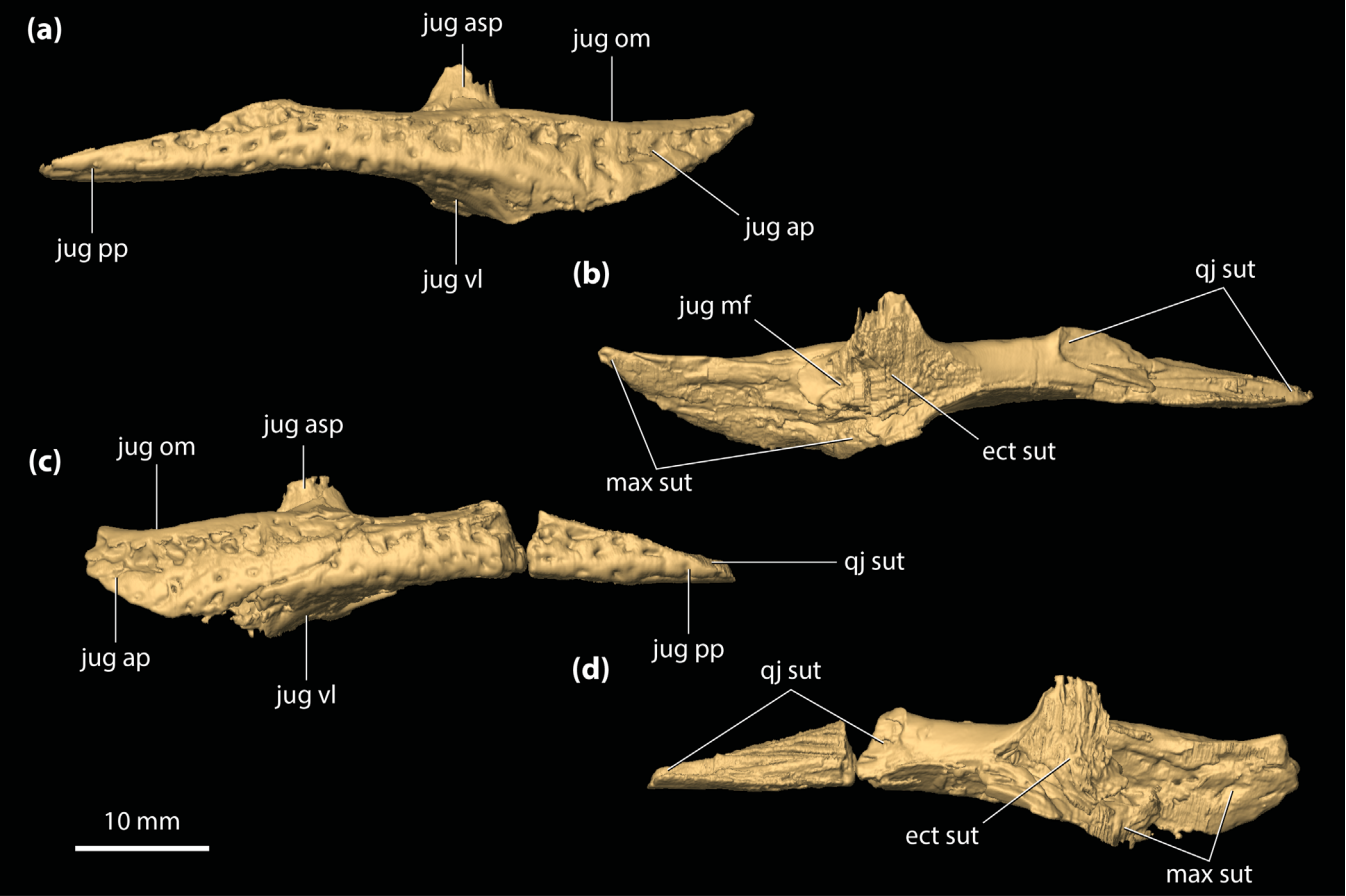
Jugals of *Trilophosuchus rackhami* Willis, 1993, QMF16856, holotype. Right jugal in (a) lateral, and (b) medial views. Left jugal in (c) lateral, and (d) medial views. Note that the left jugal is fractured at its posterior process, and is also missing the tip of its anterior process. ect sut, sutural surface for articulation with the ectopterygoid; jug ap, anterior process of the jugal; jug asp, ascending process of the jugal; jug mf, medial jugal foramen; jug om, orbital margin of the jugal; jug pp, posterior process of the jugal; jug vl, ventral lamina of the jugal; max sut, sutural surface for articulation with the maxilla; qj sut, sutural surface for articulation with the quadratojugal

Both jugals (Figures 1, 2, 3, 6, and 11; see also Figure S1.7) are well‐preserved, with the right jugal being virtually complete whereas the left is fractured. The jugals are elongated and relatively slender elements that are in contact with the maxillae anteriorly, the ectopterygoids medially, and quadratojugals posteromedially. Additionally, the jugals were in contact with the lacrimals anteriorly (Figure 6). Together with the postorbitals, the jugals formed the postorbital bars although the point of contact between these elements has been obliterated on both sides. The right jugal is complete for its entire anteroposterior length, which is ~53 mm. Each jugal can be subdivided into an anterior process, a ventral lamina, an ascending process, and a posterior process.

The anterior (suborbital) process of the right jugal is complete, whereas on the left jugal it has its tip pinched off. The smooth dorsal edge of the anterior process forms the ventral/ventrolateral margin of the orbit. The orbital margin of the anterior process of the jugal is quite thin, with its mediolateral width being ~1.5 mm. In lateral view, the orbital margin is subtly sinusoidal such that the tapered anterior tip of the jugal is pointed anterodorsally. The contact with the lacrimal would have occurred at the anterior tip of the jugal. Contact with the maxilla occurs medially so that the anterior process of the jugal overlays the maxilla posterolaterally. In lateral view, the maxilla‐jugal suture is oriented posteroventrally. As can be inferred from the more complete right jugal, its anterior process terminates posterior to the level of the anterior process of the frontal (character 192, state 2; Figure 2a). The lateral surface of the anterior process is heavily ornamented with circular to sub‐circular pits, and it is also pierced by neurovascular foramina. Towards the middle of the element, almost directly ventral to the postorbital bar, the jugal is bent ventromedially to form a ventral lamina (character 203, state 1; Figure S1.7). The ventral lamina of the jugal bears ornamentation in the form of large subcircular pits that is more expressive on the right jugal versus the left. An ornamented ventral lamina of the jugal also occurs in other crocodylians, including mekosuchines such as *Australosuchus clarkae* Willis & Molnar, 1991, *M. sanderi* and *Quinkana* Molnar, 1981 (Figure S1.8–S1.10). Anteroventral to the ascending process, a relatively small foramen (~0.8 mm in diameter) can be discerned on the medial surface of the right jugal (character 102, state 0; Figure 11b). As the medial surface on the left jugal has sustained more damage, the margins of this foramen on the left side are poorly defined. As noted by Rio and Mannion (2021), the medial surface of the anterior process is almost completely covered by the maxilla and ectopterygoid, thus exposing only a small section around the medial jugal foramen (character 242, state 1; Figure 3). Unlike *Paludirex* Ristevski et al., 2020a, *T. rackhami* does not possess a notable concavity on the ventromedial surface of the jugal.

The ascending processes of the jugals are preserved on both sides, however, they are missing their dorsal portions for contact with the descending processes of the postorbitals. Thus, the postorbital bars are incomplete. The ascending process of the jugal emerges approximately mid‐length on the element, between the anterior and posterior processes and serves as a divider between the orbit and the infratemporal fenestra. Each ascending process is subvertical and with very subtle medial inclination. The ventrolateral margin of each ascending process is almost flush with the rest of the lateral surface of the jugal, only subtly inset medially (character 135, state 0; Figure S1.2A). Laterally, at the base of each ascending process, the otherwise smooth surface is pierced by a small but conspicuous sub‐circular neurovascular foramen that is ~1 mm in diameter. Medially, the ascending process of the jugal is in firm sutural contact with the ascending process of the ectopterygoid (character 132, state 0). At its base, the anteroposterior length of the right ascending process is ~8 mm (the anteroventral margin of the left ascending process is slightly incomplete).

The posterior process of the jugal forms the ventrolateral and posterior margins of the infratemporal fenestra. In lateral view, the posterior process is relatively straight and with a heavily sculptured surface that is pierced by neurovascular foramina, just like the lateral surface of the anterior process. Upon posteromedial contact with the quadratojugal, the posterior process of the jugal begins to taper towards its posterior tip. The medial surface of the posterior process that is not in sutural contact with the quadratojugal is smooth and unornamented. Posteromedially, the sutural surface for contact with the quadratojugal extends for more than half the length of the posterior process. In ventral view of the articulated skull, it is evident that the posterior‐most tips of the jugals extend further posteriorly than the margins of the basioccipital tuberosities, and are almost in line with the occipital condyle (Figure 2b).

#### 4.3.10 Quadratojugals

**FIGURE 12:**
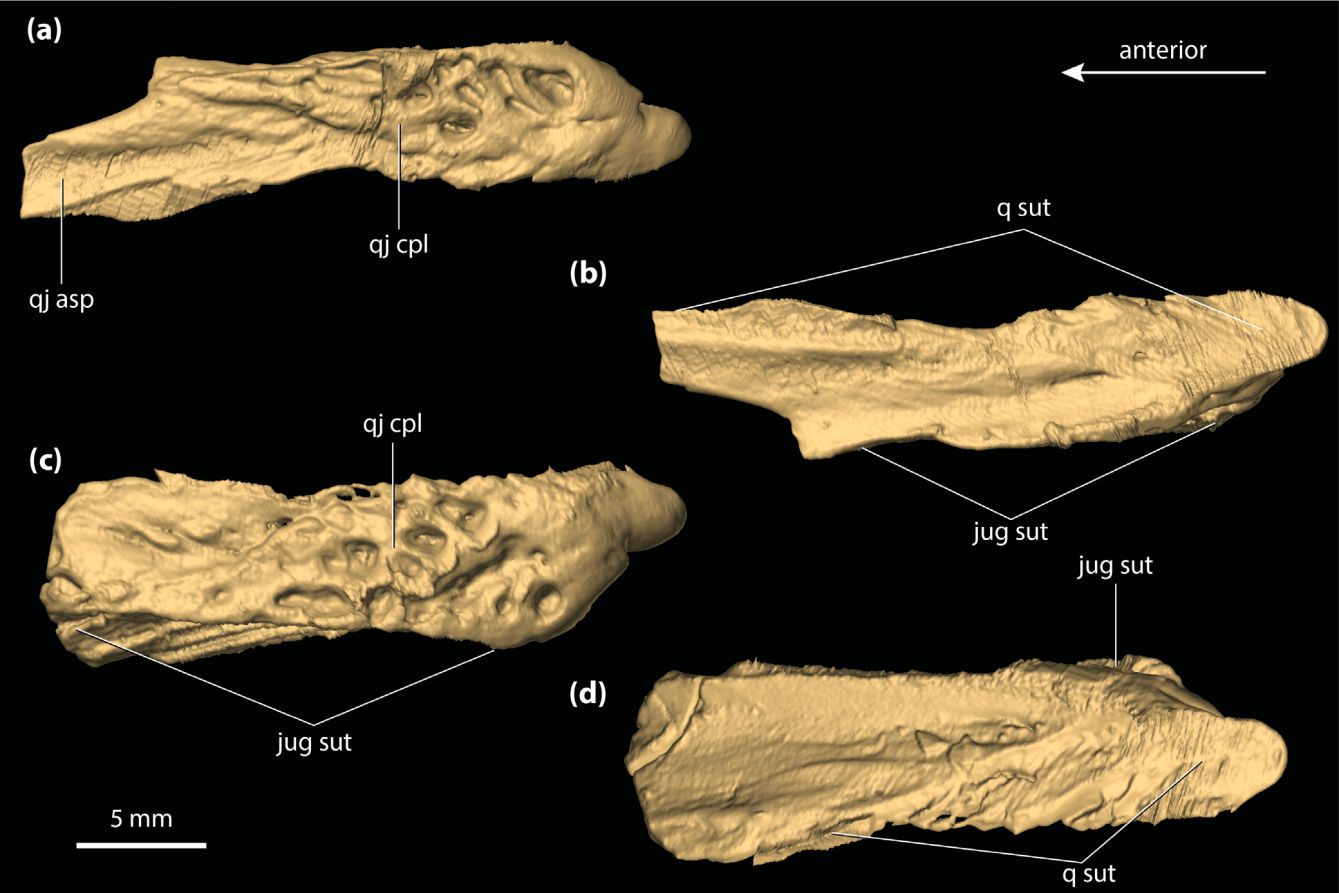
Quadratojugals of *Trilophosuchus rackhami* Willis, 1993, QMF16856, holotype. Right quadratojugal in (a) dorsolateral, and (b) ventromedial views. Left quadratojugal in (c) dorsolateral, and (d) ventromedial views. jug sut, sutural surface for articulation with the jugal; q sut, sutural surface for articulation with the quadrate; qj asp, ascending process of the quadratojugal; qj cpl, central plate of the quadratojugal

The quadratojugals (Figures 1, 2, 3 and 12) have a relatively simple morphology, by being flat and elongated plate‐like bones. Both quadratojugals are mostly complete, although they are missing their anterior portions. The preserved dimensions of the more complete right quadratojugal are ~31 mm in anteroposterior length and ~7 mm in mediolateral width. They contact the jugals lateroventrally and mediodorsally the quadrates. The quadratojugals form the posterodorsal margins of the infratemporal fenestrae and together with the jugals contribute to the formation of the infratemporal bars. As preserved, each quadratojugal is comprised of a central plate and an ascending process. The quadratojugals of *T. rackhami* lack quadratojugal spines (character 140, state 1).

The dorsolaterally oriented surface of the central plate is the only portion of the quadratojugal that bears ornamentation (Figure 12a,c). Indeed, the ornamentation on the quadratojugal occurs towards the ventrolateral portion near its contact with the posterior process of the jugal. More medially, toward the contact with the quadrate, the central plate of the quadratojugal has a smooth surface. The ventrolateral edge of the quadratojugal that is in contact with the posterior process of the jugal represents the dorsoventrally widest part of the element, having a thickness of ~4 mm. In contrast, the dorsomedial edge of the central plate is quite thin, with a dorsoventral thickness of ~1.5 mm. Posteromedially, the quadratojugal bears a low tubercle with a smooth surface that is adjacent to the lateral hemicondyle of the quadrate.

What is preserved of the ascending process of the quadratojugal is relatively nondescript—it is a flat portion of the element that is strongly dorsoventrally compressed (~0.5 mm). No sculpturing occurs on the ascending process. The ascending process of the quadratojugal forms the posterodorsal margin of the infratemporal fenestra. The right quadratojugal preserves more of the ascending process than its left counterpart, however both ascending processes are incomplete. The ventral surface of each quadratojugal is smooth, and flat to subtly concave (Figure 12b,d).

#### 4.3.11 Ectopterygoids

**FIGURE 13:**
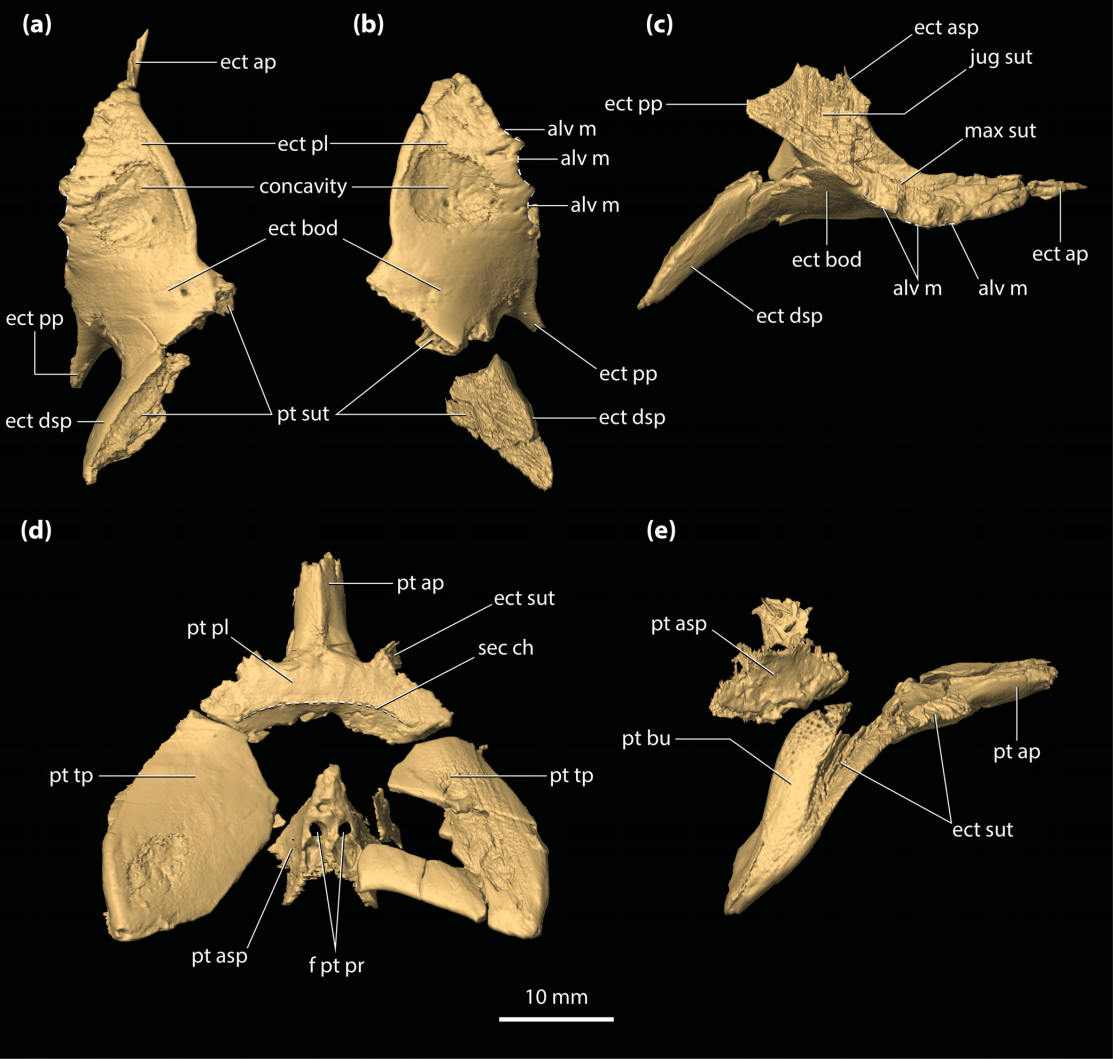
Ectopterygoids and pterygoid of *Trilophosuchus rackhami* Willis, 1993, QMF16856, holotype. Right ectopterygoid in (a) ventral, and (c) lateral views. Left ectopterygoid in (b) ventral view. Pterygoid in (d) ventral, and (e) right lateral views. Note that the ectopterygoids and pterygoid are incomplete. alv m, alveolar margin; ect ap, anterior process of the ectopterygoid; ect asp, ascending process of the ectopterygoid; ect bod, body of the ectopterygoid; ect dsp, descending process of the ectopterygoid; ect pl, ectopterygoid plate; ect pp, posterior process of the ectopterygoid; ect sut, sutural surface or articulation with the ectopterygoid; f pt pr, foramina for the pterygoid pneumatic recess; jug sut, sutural surface for articulation with the jugal; max sut, sutural surface for articulation with the maxilla; pt ap, anterior process of the pterygoid; pt asp, ascending process of the pterygoid; pt bu, pterygoid buttress; pt pl, pterygoid plate; pt sut, sutural surface for articulation with the pterygoid; pt tp, transverse process of the pterygoid; sec ch, secondary choana (anterior margin)

The ectopterygoids (Figures 1, 2, 3 and 13a–c) are relatively well‐preserved yet incomplete, having sustained some breakages. Of the two, the right ectopterygoid is the more complete and better preserved. The ectopterygoids contact the maxillae anteromedially, the jugals posteromedially, and the pterygoid posteriorly. The ectopterygoid consists of a main component—the ectopterygoid body—from which arise four processes: an anterior, an ascending, a posterior, and a descending process. In addition to these processes, the ectopterygoid of *T. rackhami* is further characterized by an expansive ectopterygoid plate. Scattered over the external surface of each ectopterygoid are small foramina.

The body of the ectopterygoid is the central component of the element. The body itself is medially sutured to the pterygoid, and it also bounds the posteromedial margin of the suborbital fenestra. There, the posteromedial margin of the suborbital fenestra is mildly concave due to a medial embayment formed by the ectopterygoid and pterygoid (character 119, state 1). Expanding anteriorly from the body is the large ectopterygoid plate. In *T. rackhami*, the ectopterygoid plate is well developed and contributes significantly to the overall size of the bone. Ventrally, the ectopterygoid plate bears a large (~7.5–8 mm in diameter), but relatively shallow sub‐circular concavity (Figure 13a,b). The presence of such concavity on the ventral surface of the expansive ectopterygoid plate is here regarded as an autapomorphic feature of *T. rackhami*. Dorsally, the ectopterygoid plate has a mildly convex surface that is inclined medially towards the ascending process of the element. Anteriorly, the ectopterygoid plate progressively decreases its width and continues towards the anterior process of the ectopterygoid.

The anterior (or maxillary) process of the ectopterygoid is partially preserved only on the right side (Figure 13a,c). It is a relatively small process that tapers to a single point (character 114, state 0). The ascending process of the ectopterygoid forms the ventromedial portion of the postorbital bar (character 132, state 0). As preserved, the ascending process is sutured to the ascending process of the jugal. Whether the ascending process of the ectopterygoid contacted the descending process of the postorbital is unknown, as both left and right postorbital bars are incomplete. The mediolateral width of the ectopterygoid's ascending process is ~7 mm.

Continuous from the ascending process is the posterior process of the ectopterygoid. In *T. rackhami*, the posterior process of the ectopterygoid is a horizontally oriented and sub‐triangular projection that is sutured to the medial surface of the jugal. Each posterior process is relatively short (~4 mm) and gradually tapers posteriorly into a blunt tip. The medial (in this case external) surface of the posterior process is subtly convex, as is the medial surface of the ascending process of the ectopterygoid.

The descending (or pterygoid) process projects posteroventrally from the body, and is in exclusive contact with the pterygoid. The descending process of the right ectopterygoid is virtually complete, whereas the left is broken off (Figure S1.4). On the right side, the descending process has a length of ~17 mm and has its ventromedial and dorsal surfaces bound by the pterygoid. In ventral view, the descending process stretches posterolaterally and has a gentle lateral bend. When observed from a lateral aspect, the descending process has a posteroventral orientation (Figure 13c). The descending process is tapered towards its tip, although it terminates well anterior to the posterior tip of the pterygoid (character 127, state 1).

One of the most notable features of the ectopterygoids is that each forms the lingual margins of the last three maxillary alveoli (character 104, state 3). The alveolar margins of the ectopterygoid are located at the medial edge of the ectopterygoid plate. The alveolar margins of the ectopterygoids have a slightly rugose texture that contrasts with the otherwise smooth surface of the bone.

#### 4.3.12 Pterygoid

Although incomplete, the pterygoid (Figures 1b–e, 2b–e, 3, 13, and S1.4) is a large element that comprises a significant portion on the ventral surface of the skull. The partially preserved pterygoid appears to be fused (as noted by Willis, 1993). The interpterygoid suture is only faintly discernable along the anterior process of the element both in the μCT scan and upon direct visual observation of the physical specimen. However, posterior to the anterior pterygoid process and anterior to the secondary choana, the interpterygoid suture is not detectable in the μCT scan nor upon direct inspection of the fossil, indicating that here the pterygoids are fully fused. The pterygoid has established sutural contact with multiple elements, including the palatines anteriorly, the ectopterygoids laterally, and dorsally with the laterosphenoids, parabasisphenoid, and quadrates. Contact between the pterygoid and the prootics is not preserved in QMF16856. Although the right prootic is nearly in touch with a fragment of the ascending pterygoid process, it remains unclear if this is a consequence of poor preservation or a genuine lack of contact between these elements. Better preserved specimens of *T. rackhami* are required in order to clarify the relationship between the pterygoid and prootics in this species. The preserved pterygoid may be sub‐divided into a large, yet incomplete central component called the pterygoid plate, from which arise an anterior process, a pair of large and laterally expansive transverse processes, and a dorsally rising ascending process.

Not much remains of the pterygoid plate, with its preserved portion being found anterior to the secondary choana. The ventral surface of the pterygoid plate anterior to the secondary choana is smooth and flat, whereas dorsally it is poorly preserved and covered by adherent fragments. The anterior edge of the pterygoid plate bounds the posterior margins of the suborbital fenestrae, whereas laterally the plate establishes sutural contact with the ectopterygoids.

Extending anteromedially from the pterygoid plate is the anterior (or palatine) process of the pterygoid (Figure 13d,e). While not broken off from the rest of the element, the pterygoid is affected by a superficial crack at the junction between the anterior process and the pterygoid plate. The anterior process forms the posteromedial margins of the suborbital fenestrae and is in direct contact with the palatines anteriorly. As mentioned above, the only trace of the interpterygoid suture is barely detectable at the anterior process. The anterior pterygoid process has a relatively consistent transverse width that is just mildly tapered anteriorly (~4 mm of transverse width near the contact with the palatines; ~4.5 mm of transverse width at the approximate mid‐length of the process; ~6 mm of transverse width posteriorly at the junction between the anterior process and the plate of the pterygoid). Additionally, this process is relatively long, having a length of ~9 mm which contributes ~33% to the total length of the palatal bar (the rest is formed by the palatines; character 118, state 2). The ventral surface of the anterior pterygoid process is convex while its dorsal surface appears to be gently concave.

The most prominent components of the pterygoid are its transverse processes. While neither the left nor right transverse process is complete, it is evident that they are large and wing‐like extensions of the bone. The right transverse process is still in articulation with the pterygoid plate, whereas the left transverse process is broken off from the rest of the element. The widest portion of the pterygoid is formed by the transverse processes, with the maximum estimated width being ~39–40 mm. The transverse processes are dorsoventrally compressed and have relatively flat dorsal and ventral surfaces. In the articulated skull, the ventral surface of each transverse process is inclined so that it faces anteroventrally. As it extends towards its posteroventral tip, the transverse process becomes gently tapered into a rather blunt tip. Laterally, each transverse process develops a hypertrophied flange called the pterygoid buttress (Figures 13e and S1.4a,b). In lateral view, the pterygoid buttress has a smoothly sigmoidal dorsal margin and a maximum dorsoventral height of ~4 mm. The lateral surface of the pterygoid flange is relatively rugose. The descending process of the ectopterygoid is wedged at the lateral margin of the transverse process of the pterygoid, with the pterygoid buttress dorsally bounding the ectopterygoid's descending process. The dorsal edge of each pterygoid buttress is blunt and smooth, with a maximum width of ~2 mm.

The ascending (or dorsal) process of the pterygoid is relatively complete but poorly preserved (Figure 13e). Due to breakage, the ascending process is detached from the rest of the pterygoid, and is preserved in tight sutural articulation with the basicranium. As preserved, the ascending pterygoid process is a cradle‐like section, with its lateral walls being formed by dorsally rising and thin bony laminae that suture to several elements of the braincase. The dorsolateral laminae are fractured and poorly preserved, particularly the lamina on the right side. Dorsomedially, the ascending process of the pterygoid establishes strong sutural contact with the parabasisphenoid. The ascending process is in contact with the laterosphenoids anterodorsally, and posterodorsally with the quadrates. On its ventral surface, the ascending process of the pterygoid is pierced by a pair of large (~1.6 mm in diameter) sub‐circular foramina related to the pterygoid pneumatic recess (Figure 13d). Posterior to these foramina, the ventral surface of the ascending pterygoid process bears a pair of deep concavities that may also be related to diverticular expansion that occurred within the pterygoid. The left and right concavities are separated from one another by a low and blunt ridge (~0.7 mm transverse width) that extends from the posterior edge of the ascending process to the posterior‐most margins of the aforementioned foramina for the pterygoid pneumatic recess.

#### 4.3.13 Palatines

**FIGURE 14:**
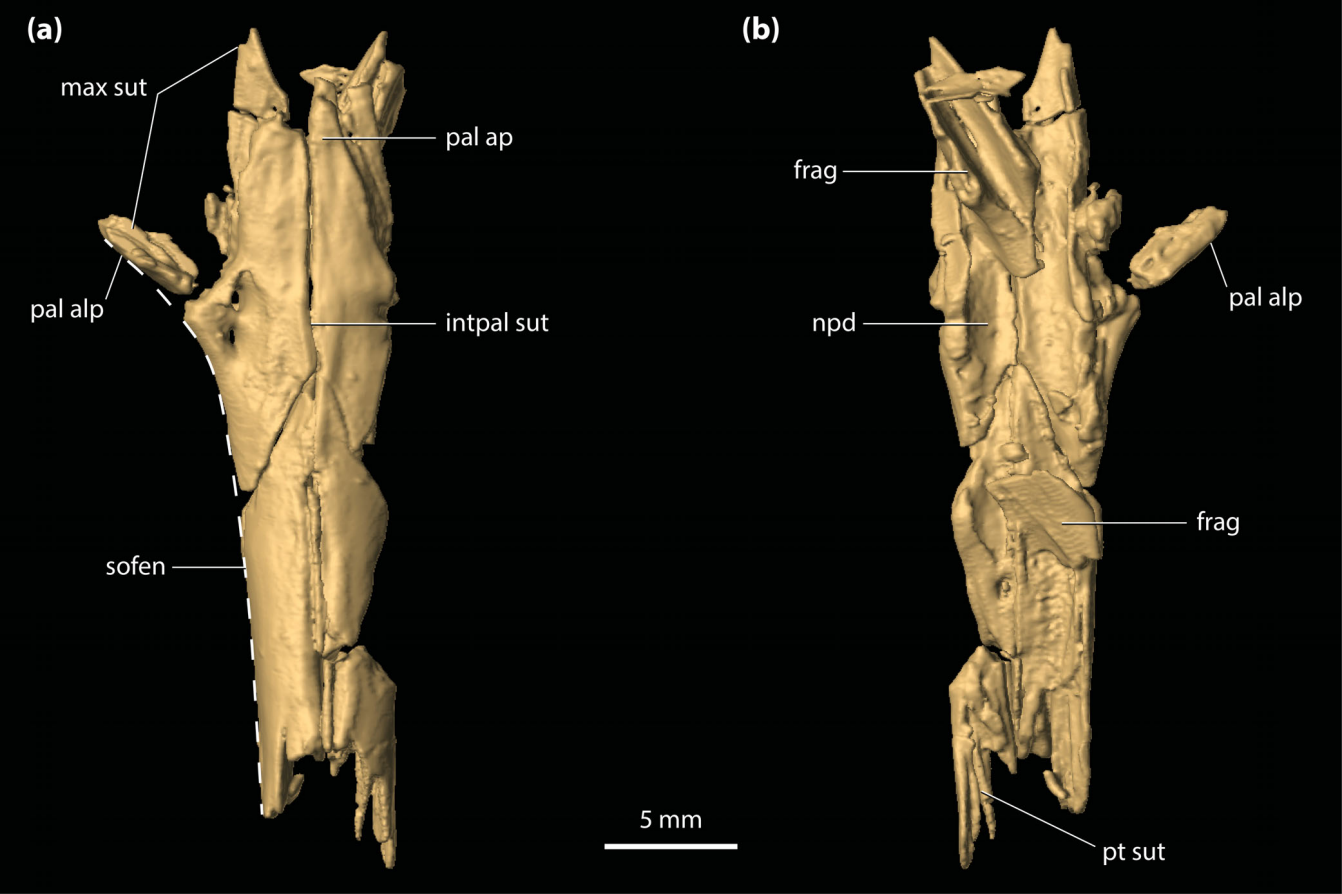
Palatines of *Trilophosuchus rackhami* Willis, 1993, QMF16856, holotype. Palatines in (a) ventral, and (b) dorsal (internal) views. Note that the palatines are incomplete and have sustained fractures. frag, fragment; intpal sut, interpalatine suture; max sut, sutural surface for articulation with the maxilla; npd, nasopharyngeal duct; pal alp, anterolateral process of the palatine; pal ap, anterior process of the palatine; pt sut, sutural surface for articulation with the pterygoid; sofen, suborbital fenestra (medial margin)

The palatines (Figures 1b, 2b, 3, and 14) are imperfectly preserved, having sustained substantial damage. They are relatively slender elements that form an important component of the secondary bony palate. As preserved, the palatines are in contact with the maxillae anteriorly, the pterygoid posteriorly, and medially they contact each other. The dorsal surface of each palatine is quite poorly preserved, as it is covered in adherent fragments and the ventral floor of the nasopharyngeal duct is exposed. Consequently, their contact with the prefrontals (expected to have occurred dorsally on the palatines) is not preserved.

In ventral aspect, the paired palatines form most of the long and narrow palatal bar (~29 maximum preserved anteroposterior length, and ~5 mm minimum transverse width as measured mediolaterally across both palatines). Their ventral (=palatal) surfaces are flat. The palatal bar is situated between the suborbital fenestrae, with the lateral margin of each palatine bounding much of the medial margin of its respective suborbital fenestra. The interpalatine suture between the bones is straight. The lateral margins of the palatines are parallel to each other, with the exception of the anterolateral process that, as suggested by its name, curves anterolaterally. The small anterolateral process of the right palatine is partially preserved in articulation with the posterior rim of the palatal process of the maxilla; however, it is detached from the right palatine by a fracture that has occurred at the base of the process (Figure 14a). As preserved, the anterolateral palatine process is ~4 mm long and its lateral margin bounds the anteromedial edge of the suborbital fenestra. Posteromedially to the anterolateral process, the right palatine is pierced by two foramina. The anterior processes of the palatines are incomplete at their tips. Based on what remains of them, they wedged between the palatal processes of the maxillae and they may have gradually tapered toward their anterior tips. Relative to the anterior‐most margins of the suborbital fenestrae, the anterior processes of the palatines extend slightly more anteriorly (character 115, state 0; Figures 1b and 2b). Extending posteriorly towards their contact with the anterior process of the pterygoid, the palatines maintain their straight trajectory and remain parallel with each other (character 120, state 0).

### 4.4 Chondrocranial bones

#### 4.4.1 Laterosphenoids

**FIGURE 15:**
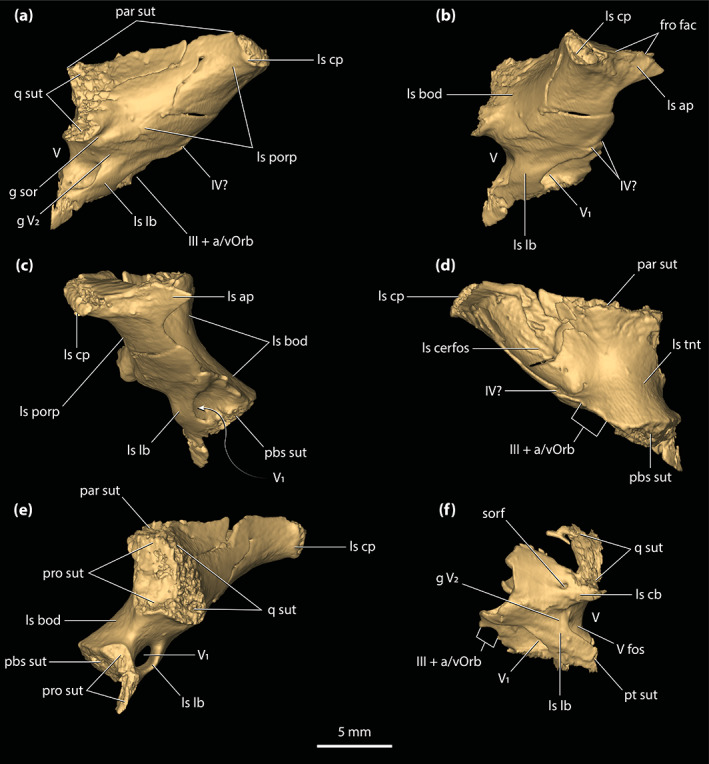
Laterosphenoids of *Trilophosuchus rackhami* Willis, 1993, QMF16856, holotype. Right laterosphenoid in (a) lateral, (b) anterolateral, (c) anterior, (d) medial, and (e) posterior views. Left laterosphenoid in (f) lateral view. Note that much of the left laterosphenoid is missing. fro fac, frontal facet; g sor, groove for the supraorbital branch of the trigeminal nerve; g V_2_, groove for the maxillary branch of the trigeminal nerve; III + a/vOrb, common foramen the oculomotor nerve and the orbital artery and vein; IV?, trochlear foramen (tentative); ls ap, anterior process of the laterosphenoid; ls bod, body of the laterosphenoid; ls cb, caudal bridge of the laterosphenoid; ls cerfos, cerebral fossa of the laterosphenoid; ls cp, capitate process of the laterosphenoid; ls lb, lateral bridge of the laterosphenoid; ls porp, postorbital process of the laterosphenoid; ls tnt, tentorial crest of the laterosphenoid; par sut, sutural surface for articulation with the parietal; pbs sut, sutural surface for articulation with the parabasisphenoid; pro sut, sutural surface for articulation with the prootic; pt sut, sutural surface for articulation with the pterygoid; q sut, sutural surface for articulation with the quadrate; sorf, foramen for the supraorbital branch of the trigeminal nerve; V, trigeminal foramen; V_1_, foramen for the ophthalmic branch of the trigeminal nerve; V fos, trigeminal fossa

As in other crocodylians (see Holliday & Witmer, 2009; Kuzmin et al., 2021), the complex laterosphenoids (Figures 2c,d, 3, and 15) of *T. rackhami* comprise the orbitotemporal region of the skull and may be divided into two main divisions: anteriorly it is the orbital surface, and posteriorly is the temporal surface (laterosphenoid posterolateral lamina sensu Kley et al., 2010). The laterosphenoid can be further subdivided into: the body, the anterior process (that forms the orbital surface), the postorbital and capitate processes (that form the temporal surface; the body of the laterosphenoid also contributes to the formation of the temporal surface), and smaller processes such as the lateral and caudal bridges. Crocodylian laterosphenoids also possess another small process, the slender process (which contributes to the orbital surface); however, the slender processes are not preserved in QMF16856. The right laterosphenoid (Figure 15a–e) is mostly complete, whereas the left (Figure 15f) only partially preserves the body along with the lateral and caudal bridges. Overall, the preservational quality of the right laterosphenoid is good, having suffered relatively minor cracks and breakages. However, the anterior process of the right laterosphenoid may not be complete and is likely missing some of its anteromedial portion. The preserved surfaces of the laterosphenoids are smooth. This description is based primarily on the right laterosphenoid as it is the more complete of the two. Where appropriate, additional comments will be made based on the left laterosphenoid.

The laterosphenoid body is well‐preserved on both sides (although less complete on the left). Dorsally, the body of the laterosphenoid is in contact with the parietal, ventrally with the parabasisphenoid, posteriorly it contacts the prootic, and posterolaterally the quadrate. Contact with the ascending process of the pterygoid also occurs ventrally, however, it is relatively poorly preserved due to the condition of the pterygoid. The posterior portion of the laterosphenoid body bounds the anterior margins of the trigeminal foramen (=foramen ovale) and fossa. The lateral surface of the laterosphenoid body (together with the lateral bridge) encloses the shared canal for the ophthalmic branch of the maxillary nerve and trigeminal artery and associated veins (= cavum epiptericum sensu Holliday & Witmer, 2009; for other crocodylians, see Lessner & Holliday, 2020, Porter et al., 2016, Sedlmayr, 2002, and Kuzmin et al., 2021). From an anterior view, the cavum epiptericum has an elliptical outline, having a length of ~2 mm along its major axis and ~0.9 mm along its minor axis. Internally, the body of each laterosphenoid is occupied by a substantial pneumatic recess (see Ristevski, 2022).

Each laterosphenoid possesses a well‐developed lateral bridge. The lateral bridge of the laterosphenoid is a mediolaterally thin structure (<1 mm) that encloses the cavum epiptericum laterally. It has a mildly convex lateral surface and a mildly concave medial surface. The anteroposterior length of each lateral bridge is ~2 mm. Immediately posterodorsal to each lateral bridge is a shallow groove made by the maxillary branch of the trigeminal nerve. Posteriorly on the left laterosphenoid is the small but stout caudal (=posterior) bridge (Figure 15f). Its thickness/mediolateral width is ~1 mm whereas the dorsoventral length of the caudal bridge is ~1.5 mm. The caudal bridge of the left laterosphenoid bounds the canal for the supraorbital branch of the maxillary nerve and separates it from the maxillary branch of the trigeminal nerve. The canal for the supraorbital branch of the maxillary nerve pierces the left laterosphenoid through a foramen found ventral to the caudal bridge and exits via another foramen positioned dorsal to the caudal bridge. Both of these subcircular foramina are small, being ~0.5 mm in diameter. On the right laterosphenoid, there is no caudal bridge, nor a foramen for the supraorbital branch of the maxillary nerve. Instead, a conspicuous groove is the only mark that the supraorbital nerve has left on the right laterosphenoid (Figure 15a). It appears that this asymmetry between the left and right laterosphenoids is not a result of preservation, as there is no obvious damage on the right laterosphenoid to explain the lack of the caudal bridge or a foramen for the supraorbital nerve. Like most crocodylians, *T. rackhami* lacks an epipterygoid (see Holliday & Witmer, 2009).

The preserved orbital surface of the laterosphenoid is formed by the anterior process (sensu Kuzmin et al., 2021; laterosphenoid anterolateral lamina sensu Kley et al., 2010). Here, the laterosphenoid establishes contact with the frontal (specifically, at the posterior margin of the frontal's descending process) dorsally along the dorsal margin of its anterior process (i.e., the frontal facet of the laterosphenoid). The anterior process of the right laterosphenoid may not be completely preserved, and thus, confident interpretation regarding the degree of anterior ossification of the braincase is precluded. A small fissure‐like crack partially affects the anterior process of the right element. On both laterosphenoids, the margin of the common foramen for the oculomotor nerve and the orbital artery and vein is distinguishable as a semicircular notch. A small foramen present on the right laterosphenoid is here tentatively interpreted as the foramen for the trochlear nerve (Figure 15a,b). The putative trochlear foramen is very small (~0.3 mm in diameter) and pierces the anterior process of the right laterosphenoid.

The columnar postorbital process of the laterosphenoid is not strongly verticalized as it displays an anterior inclination. Minor cracks have affected the right postorbital process. Crocodyliform laterosphenoids are commonly characterized by crests that lie on the postorbital process and/or orbital surface (i.e., the antotic crest, the tensor crest, and the cotylar crest; e.g., Busbey III, 1989; Holliday & Witmer, 2009; Kley et al., 2010; Kuzmin et al., 2021; Ristevski et al., 2020a, 2021; Sertich & O'Connor, 2014). In the *T. rackhami* holotype, the surface of the postorbital process is quite smooth and convex, which makes discerning of these crests difficult. Situated dorsally on the postorbital process is the capitate process. The capitate process of the right laterosphenoid has a relatively rugose tip/dorsal surface that fits (but is not in direct contact) within a socket/concavity found on the ventral surface of the postorbital, as in other crocodylians (Figure 9e,f; see also Holliday & Witmer, 2009, Iordansky, 1973, Kuzmin et al., 2021). When the skull is observed in ventral aspect (see the interactive 3D PDF), the anterodorsal‐most preserved margin of the anterior process of the right laterosphenoid extends slightly more anterior than the capitate process. This indicates that the relation between the capitate process of the laterosphenoid and the olfactory foramen in *T. rackhami* is akin to that of most crocodylians (character 164, state 1), with the exception of some gavialoids which have the capitate processes and olfactory foramen in line with each other (character 164, state 0; e.g., Brochu, 1997; Kuzmin et al., 2021; Rio & Mannion, 2021).

In medial aspect, the right laterosphenoid is more informative than the left (Figure 15d). Anteromedially, the laterosphenoid is characterized by a deeply concave cerebral fossa, and posterior to it is the blunt tentorial crest. The tentorial crest is present on the medial side of the laterosphenoid body and has a convex medial surface that is inclined laterally. The medial surface of the right laterosphenoid, within the cerebral fossa, has sustained some fractures. Posterodorsally, at the contact with the prootic and parietal, the medial surface of the laterosphenoid posterior to the tentorial crest is not distinctly concave, and thus the mesencephalic fossa (sensu Kuzmin et al., 2021) has no prominent appearance on this element.

#### 4.4.2 Prootics

**FIGURE 16:**
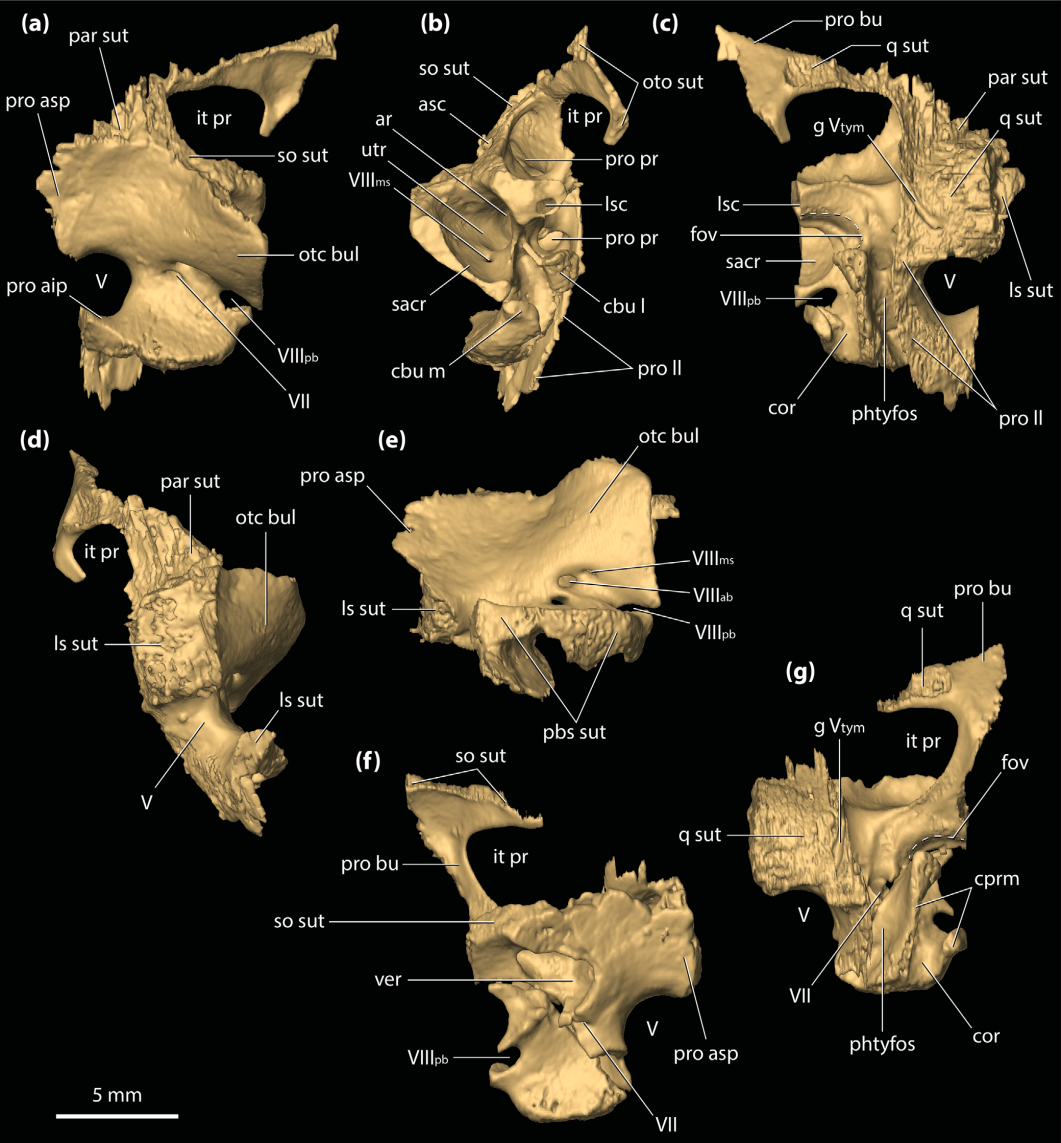
Prootics of *Trilophosuchus rackhami* Willis, 1993, QMF16856, holotype. Right prootic in (a) medial, (b) posterior, (c) lateral, (d) anterior, and (e) ventral views. Left prootic in (f) medial, and (g) lateral views. Note that the left prootic is less complete than the right. ar, ampullary recess; asc, anterior semicircular canal; cbu l, lateral bulge of the cochlear prominence; cbu m, medial bulge of the cochlear prominence; cor, cochlear recess; cprm, cochlear prominence; fov, fenestra ovalis; g V_tym_, groove for the tympanic branch of the trigeminal nerve; it pr, intertympanic pneumatic recess; ls sut, sutural surface for articulation with the laterosphenoid; lsc, lateral semicircular canal; otc bul, otic bulla; oto sut, sutural surface for articulation with the otoccipital; par sut, sutural surface for articulation with the parietal; pbs sut, sutural surface for articulation with the parabasisphenoid; phtyfos, pharyngotympanic pneumatic fossa; pro aip, anterior inferior process of the prootic; pro asp, anterior superior process of the prootic; pro bu, prootic buttress; pro ll, lateral lamina of the prootic; pro pr, prootic facial pneumatic recess; q sut, sutural surface for articulation with the quadrate; sac r, saccular recess; so sut, sutural surface for articulation with the supraoccipital; utr, utricular recess; V, trigeminal foramen; ver, vestibular recess (damaged); VII, foramen for the facial nerve; VIII_ab_, foramen for the anterior branch of the vestibulocochlear nerve; VIII_ms_, foramen for the macula sacculi branch of the vestibulocochlear nerve; VIII_pb_, foramen for the posterior branch of the vestibulocochlear nerve

The prootics of *T. rackhami* (Figures 3 and 16) are complex elements of the braincase that are in tight sutural contact with several bones of the cranium. Anteriorly, each prootic sutures to the laterosphenoid, posteriorly to the otoccipital, anterodorsally to the parietal (direct sutural contact is preserved only with the right prootic), posterodorsally to the supraoccipital, ventrally to the parabasisphenoid (due to how the specimen is preserved, the prootic‐parabasisphenoid contact has been disarticulated in QMF16856), and laterally to the quadrate. Ventrally, the prootics of crocodylians also contact the pterygoids (Kuzmin et al., 2021). This was likely the case in the *T. rackhami* holotype as well, since the ascending process of the pterygoid approaches the prootics ventrally. However, due to damage on the lateral wall of the braincase, the prootic‐pterygoid contact is not preserved on either side. Of the two prootics, the right prootic (Figures 3a and 16a–e) is the more complete and better preserved. The maximum dimensions of the right prootic are ~18 mm in dorsoventral height, ~10.5 mm in anteroposterior length, and ~8 mm in mediolateral width. In the articulated skull, the prootics are largely obscured from view by its adjacent bones, being only partially visible in lateral aspect as forming the posterior margins of the trigeminal foramen and fossa (character 162, state 1). Small sections of each prootic are also visible through the external auditory meatus (Figure 2c,d). Internally, the prootic is pneumatized by the prootic facial recess (Ristevski, 2022). As proposed by Kuzmin et al. (2021), the crocodylian prootic may be subdivided into several portions, with the same subdivisions being applicable to the prootic of *T. rackhami* as well. These subdivisions are the capsular portion, the superior and inferior anterior processes, the lateral lamina, and the prootic buttress.

The capsular portion of the prootic comprises the anterior part of the ossified otic capsule that bulges medially into the endocranial cavity as the otic bulla of the prootic. The otic bulla (=tympanic bulla of Kley et al., 2010) is also completed by the capsular portions of the supraoccipital dorsally and the otoccipital posteriorly. The otic bulla is complete on the right prootic, whereas the bulla of the left has been destroyed (Figure 16f). Within the otic bulla is the large vestibular recess. Internally, the complete vestibular recess of the right prootic can be subdivided into additional smaller recesses (the saccular, utricular, and ampullary recesses), each subdivided by low and blunt ridges (Figure 16b). Anterodorsally, the capsular portion is pierced by the anterior semicircular canal and laterally by the lateral semicircular canal. On the capsular portion of the prootic, the opening for the anterior semicircular canal is subcircular and has a diameter of ~0.5 mm, whereas the one for the lateral semicircular canal is slightly elliptical and has a length of ~0.8 mm along its major axis and ~0.5 mm along its minor axis. At the ventromedial margin of the right otic bulla, the prootic is pierced by four foramina, one related to the facial and three to the vestibulocochlear nerves: the foramen for the facial nerve, the foramen for the anterior branch of the vestibulocochlear nerve, the foramen for the posterior branch of the vestibulocochlear nerve, and the foramen for the macula sacculi branch of the vestibulocochlear nerve (Figure 16a,e). On the left prootic, only the foramen for the facial nerve partially preserves its margins ventromedially on the element (Figure 16f).

Ventrolaterally on the prootic is the cochlear prominence, which is bounded by its lateral bulge anterolaterally and its medial bulge posteroventrally. Bounded anteriorly and posteriorly by the cochlear prominence is the cochlear recess. The cochlear recess is a smooth and concave space on the lateral surface of the prootic that contained the cochlear duct of the inner ear. Located dorsally to the cochlear recess of each prootic is a notched depression that corresponds to the fenestra ovalis (=fenestra vestibuli, or oval window of Witmer et al., 2008). Due to damage, the fenestra ovalis of the left prootic is not as well defined as the one on the right. Anteriorly, the prootic bounds the trigeminal foramen and fossa ventrally, posteriorly, and dorsally. The ventral margin of the trigeminal foramen is formed by the anterior inferior process of the prootic, whereas the dorsal by the anterior superior process of the prootic. Both of these processes suture to the laterosphenoid anteriorly, with the anterior inferior process additionally having contacted the parabasisphenoid ventrally (this contact is loosely preserved in QMF16856), while the anterior superior process is in contact with the quadrate laterally. The anterior inferior process of the left prootic is broken off and missing.

The lateral lamina is completely preserved on the right prootic, and is almost complete on the left. The lateral surface of the lateral lamina is tightly sutured to the quadrate, and ventrally it would have sutured to the parabasisphenoid and (likely) pterygoid as well, although this contact is imperfectly preserved. Dorsolateral to the trigeminal foramen, the lateral lamina of each prootic is marked by a diagonal groove for the tympanic branch of the trigeminal nerve. Medially, the lateral lamina is smooth and bounds the concave wall of the pharyngotympanic fossa. The pneumatic pharyngotympanic fossa has an anteroposterior length of ~1 mm.

The dorsal component of the element is the prootic buttress. The prootic buttress is an elongated, mediolaterally thin structure that curves posterolaterally and dorsally. It is incomplete on both the left and right prootic. The prootic buttress on the right side arises dorsally from the anterior superior process of the element and has a width of ~3 mm at its base. It then proceeds to extend in a laterodorsal direction, where it attains a minimum width of ~1 mm. As it continues posterolaterally, the buttress gradually expands its dorsoventral height until it establishes its contact with the otoccipital posteriorly. It then begins to gradually constrict again as it continues ventromedially to eventually merge with the posterior of the capsular portion of the prootic. This ventromedial extension that connects to the capsular portion is not preserved on the right prootic but is still present on the left. The intertympanic pneumatic recess is surrounded by the prootic buttress. Posterolaterally and posteromedially, the prootic buttress has smooth surfaces, whereas its more anterior surfaces suture to neighboring elements, specifically with the supraoccipital dorsally, and laterally with the quadrate. In the articulated skull, each prootic buttress is faintly visible through the anterior temporal foramen as it forms the floor of the temporal canal, although its exposure is significantly obscured by other cranial bones that surround the canal. Additionally, the prootic buttress is visible through the external auditory meatus (Figure 2c,d).

#### 4.4.3 Supraoccipital

**FIGURE 17:**
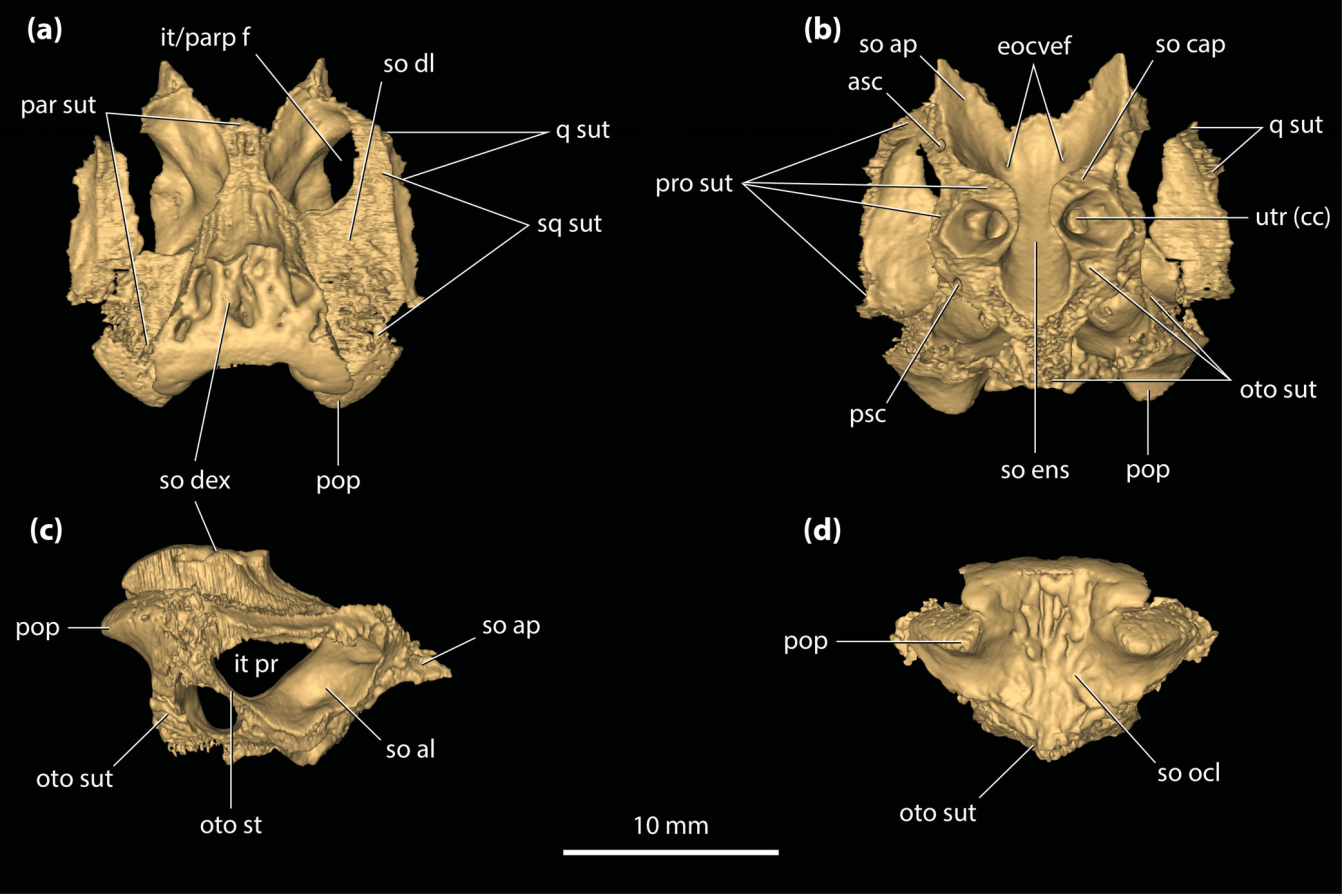
Supraoccipital of *Trilophosuchus rackhami* Willis, 1993, QMF16856, holotype. Supraoccipital in (a) dorsal, (b) ventral, (c) right lateral, and (d) posterior views. Note that the dorsal lamina on the left side is incomplete. asc, anterior semicircular canal; eocvef, foramina for the external occipital veins; it pr, intertympanic pneumatic recess; it/parp f, foramen for communication between the intertympanic and parietal pneumatic recesses; oto st, otoccipital strut; oto sut, sutural surface for articulation with the otoccipital; par sut, sutural surface for articulation with the parietal; pop, postoccipital process of the supraoccipital; pro sut, sutural surface for articulation with the prootic; psc, posterior semicircular canal; q sut, sutural surface for articulation with the quadrate; so al, anterior lamina of the supraoccipital; so ap, anterior process of the supraoccipital; so cap, capsular portion of the supraoccipital; so dex, dorsal exposure of the supraoccipital; so dl, dorsal lamina of the supraoccipital; so ens, endocranial surface of the supraoccipital; so ocl, occipital lamina of the supraoccipital; sq sut, sutural surface for articulation with the squamosal; utr (cc), utricular recess (common crus)

The *T. rackhami* holotype has a well‐preserved supraoccipital (Figures 2a,e, 3, and 17). This element is in contact with the parietal anteriorly and dorsally, the prootics anteroventrally, and the otoccipitals posteroventrally. Additionally, the supraoccipital also contacts the squamosals dorsolaterally, and a relatively short contact with the quadrates is established along the elements' lateral margins. The supraoccipital may be subdivided into three major components: the anterior lamina, the occipital (=posterior) lamina, and the dorsal lamina. Internally, the supraoccipital has a substantial cavity that housed the intertympanic pneumatic recess (see Ristevski, 2022).

The anterior lamina of the supraoccipital (Figure 17a–c) has an anteroventral orientation and forms the posterodorsal portion of the braincase. The anterior lamina is subdivided into the capsular portions and the central endocranial portion/surface. The paired capsular portion of the supraoccipital form the dorsal sections of the otic bullae. They face ventrally and have markedly convex medial surfaces. Internally, each capsular portion has a large space to accommodate the common crus (corresponding with the utricular recess; for other crocodylians see Kuzmin et al., 2021). The opening for the common crus on each capsular portion of the supraoccipital is roughly subcircular, with an anteroposterior length of ~2 mm and a mediolateral width of ~3 mm. Two additional foramina also pierce the capsular portion along its sutural surfaces/facets with the prootic and the otoccipital. The foramen that pierces the prootic facet corresponds to that for the anterior semicircular canal, while the foramen piercing the otoccipital facet corresponds to the posterior semicircular canal. The foramina for the semicircular canals are sub‐oval and have diameters of ~0.5 to ~0.7 mm. Internally within the capsular portion, the anterior and posterior semicircular canals meet the common crus (see Ristevski, 2022). The thickness of the capsular portion walls is variable, such that the anterior wall is thickest (~1 mm) whereas the wall is thinnest medially (~0.2 mm).

The central endocranial surface of the anterior lamina is deeply concave, with its most notable feature being the pair of foramina for the external occipital veins (Figure 17b). These foramina pierce the anterior of the central endocranial surface near the suture with the parietal (~1.4 mm minimum distance between the right foramen and the parietal–supraoccipital suture; ~1.6 mm minimum distance between the left foramen and the parietal–supraoccipital suture). Each foramen for the external occipital vein has an elliptical outline with a length of ~0.2 mm along its minor and ~0.4 mm along its major axis, and are separated from each other by a distance of ~2 mm. These are the only two foramina that pierce the central endocranial surface of the anterior lamina of the supraoccipital ventrally. Dorsally, the anterior lamina has a convex surface. Dorsolaterally, near the facets for the prootics and otoccipitals (or, dorsal to the capsular portions) the dorsal surface has small concave areas. Extending anterolaterally from the anterior lamina on its left and right sides are the anterior processes. Each anterior process has an anteroposterior length of ~3 mm and tapers towards its anterior margin. Dorsally, each anterior process bears a facet that contacts the parietal. Posteroventrally, the anterior lamina merges with the occipital lamina. This posterior section of the anterior lamina is sutured to the otoccipitals. Extending posterolaterally on each side of the anterior lamina of the supraoccipital is an otoccipital strut. The otoccipital struts are thin structures that are inclined posterodorsally and laterally, with their ventral surfaces being sutured to the otoccipitals. Distally, each strut connects to the occipital lamina of the supraoccipital, specifically to the anterior (i.e., internal) surface of the postoccipital process. The length of each otoccipital strut is ~4 mm and has a minimum thickness of ~0.5 mm (measured approximately mid‐length).

Among known Australian crocodylians, the occipital lamina of the supraoccipital of *T. rackhami* has a unique morphology (Figures 1e, 2e, 17d, and S1.5; see Document S1 for morphological comparisons). In occipital aspect, the occipital lamina has a subtriangular outline, being widest at its dorsal margin (~14 mm, as measured at the dorsolateral‐most levels of the postoccipital processes) and bluntly tapering ventrally along its sutural contact with the otoccipitals. The total dorsoventral height of the supraoccipital as measured on its occipital lamina is ~9 mm. The occipital lamina has a fully posterior orientation and a relatively flat surface that is devoid of a sagittal nuchal crest or any concavities (character 257, state 2). Furthermore, the occipital lamina is not ornamented with pits like it is in *M. sanderi* (Figure S1.6), however, along its midline it displays a distinctly wrinkled texture (Figures 17d and S1.5). These wrinkles spread mid‐sagittally on the occipital lamina but are primarily concentrated on the dorsal half, between the postoccipital processes and posttemporal fenestrae. In addition, the wrinkled surface is also pierced by several tiny foramina that are dispersed over the dorsal half of the occipital lamina. Similar to *T. rackhami* is the condition in *A. clarkae*, where the occipital lamina of the supraoccipital is also flat and without a nuchal crest or concavities. However, unlike *T. rackhami*, the occipital lamina of the supraoccipital in *A. clarkae* has a smooth surface without wrinkles or any notable foramina (Figure S3.4c). Situated dorsolaterally on the occipital lamina are the postoccipital processes. The postoccipital processes project posteriorly from the supraoccipital and are visible in dorsal view on the articulated skull (Figures 1a and 2a). Each postoccipital process has a roughly pyramidal shape, with a pointed but blunt tip that projects posteriorly. The dorsal surface of each postoccipital process is relatively flat. Although well‐developed, the postoccipital processes are only moderately large, and proportionally they are not as extensive as the postoccipital processes of some gavialoids (character 225, state 0; see Ristevski et al., 2021). Furthermore, the postoccipital processes of *T. rackhami* are separated from each other by a distance of ~6.5 mm (which is ~46% the total width of the supraoccipital) and are thus not spaced as closely to one another as in some members of Gavialidae (Ristevski et al., 2021).

The supraoccipital of *T. rackhami* has a prominent dorsal exposure, although it does not exclude the parietal from reaching the posterior margins of the cranial table (character 159, state 2; Figure 2a). The dorsal exposure occupies the posteromedial portion of the cranial table and attains a roughly trapezoid shape (character 198, state 0). The dorsal exposure of the supraoccipital is in contact only with the parietal, with the parietal's posterior processes bounding the dorsal exposure laterally. The dorsal exposure has a flat dorsal surface that does not slope ventrally. The posterior margin of the dorsal exposure is smooth, whereas more anteriorly the dorsal surface is ornamented with several sub‐circular pits akin to the rest of the cranial table. The dorsal exposure of the supraoccipital is widest near its posterior margin, with a transverse width of ~8.5 mm. Its maximum anteroposterior length is ~5 mm (measured dorsally on the cranial table from the parietal–supraoccipital suture anteriorly to the poster‐most margin of the dorsal exposure posteriorly), indicating that the supraoccipital comprises approximately 9% of the total anteroposterior length of the cranial table (as measured from the anterior process of the frontal anteriorly to the posterior‐most margin of the supraoccipital's dorsal exposure posteriorly). Extending anterior to the dorsal exposure, the supraoccipital bears an anteroventrally inclined sutural facet for contact with the ventral surface of the parietal (a scarf joint sutural contact, with the parietal overlaying this part of the supraoccipital). Relative to the dorsal lamina, the dorsal exposure of the supraoccipital is elevated by ~2 mm (Figure 17c).

The dorsal lamina of the supraoccipital is a relatively wide (~14 mm transverse width) but thin (~0.4 mm) section of the element. The dorsal lamina is horizontal and flat, with its dorsal surface serving as the sutural establishment between the supraoccipital and parietal, and the supraoccipital and squamosals. The contact with the medial processes of the squamosals occurs along the lateral edges of the dorsal lamina. According to Kuzmin et al. (2021), contact between the supraoccipital and the squamosals is also present in extant caimanines. The dorsal lamina bears two large openings for the foramina that serve as communications between the intertympanic and parietal pneumatic recesses. As the dorsal lamina is incomplete on the left side, the left foramen is also incomplete along its posterior and lateral margins. The foramen on the right side of the dorsal lamina is complete, displaying a sub‐circular outline with an anteroposterior length of ~5 mm and a mediolateral width of ~4 mm. The lateral margins of the dorsal lamina are smooth and unornamented, and bound the medial walls of the temporal canals. The anterior sections of the aforementioned lateral margins are also exposed on the medial walls of the supratemporal fossae (Figure S1.3). For brief lengths, the lateroventral margins of the dorsal lamina establish contact with the anteromedial processes of the quadrates.

#### 4.4.4 Otoccipitals

**FIGURE 18:**
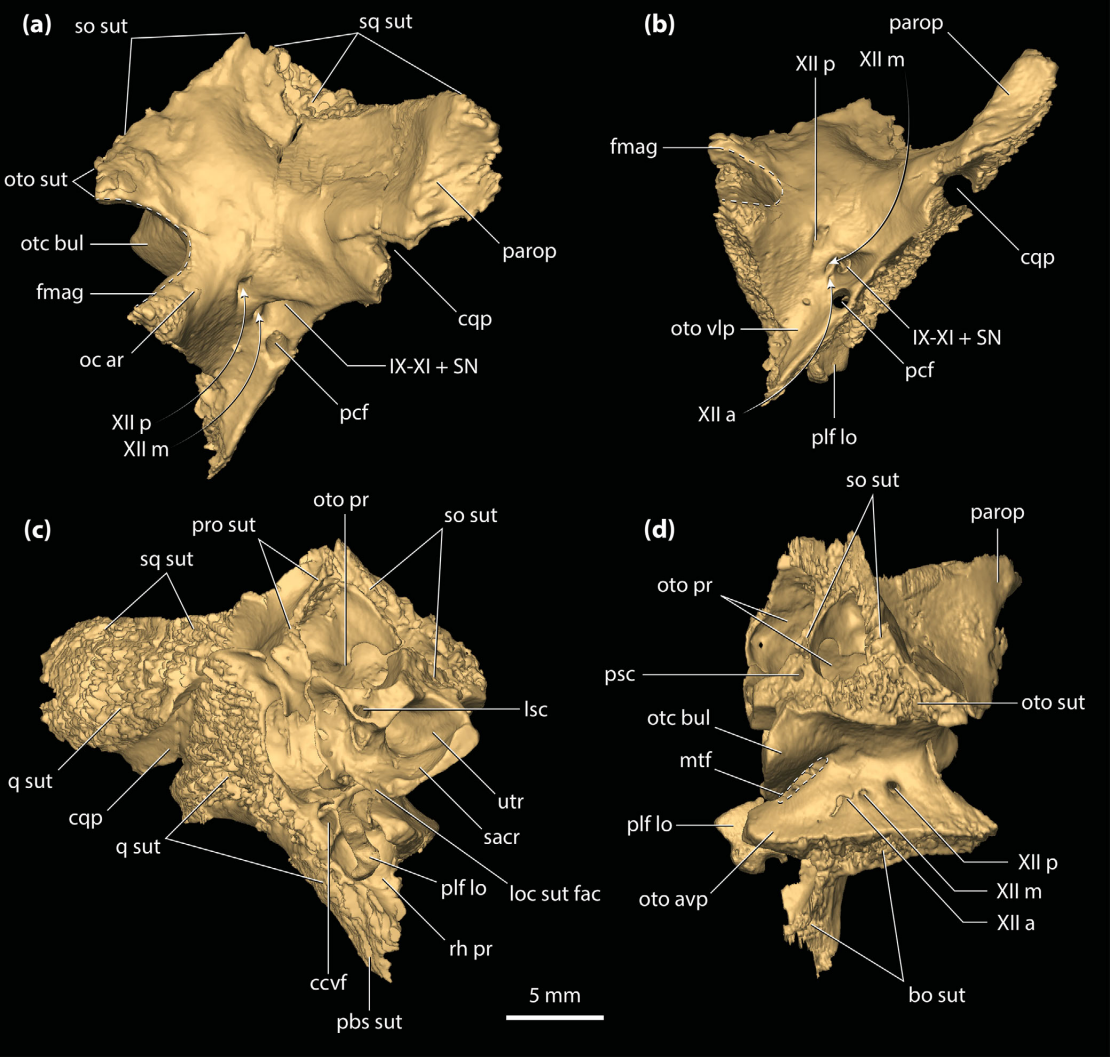
Right otoccipital of *Trilophosuchus rackhami* Willis, 1993, QMF16856, holotype. Right otoccipital in (a) posterior, (b) oblique ventral, (c) anterior, and (d) medial views. Note that the perilymphatic loop is incomplete and disarticulated. bo sut, sutural surface for articulation with the basioccipital; ccvf, cerebral carotid vasculature foramen; cqp, cranioquadrate passage; fmag, foramen magnum; loc sut fac, facet for the loop closure suture; lsc, lateral semicircular canal; mtf, metotic foramen; IX‐XI + SN, common external foramen for the glossopharyngeal, vagus, accessory, and sympathetic nerves; oc ar, occipital arch; oto avp, anteroventral process of the otoccipital; otc bul, otic bulla; oto pr, otoccipital pneumatic recess; oto sut, sutural surface for articulation with the otoccipital; oto vlp, ventrolateral process of the otoccipital; parop, paroccipital process; pbs sut, sutural surface for articulation with the parabasisphenoid; pcf, posterior carotid foramen; plf lo, perilymphatic loop (fragment); pro sut, sutural surface for articulation with the prootic; psc, posterior semicircular canal; q sut, sutural surface for articulation with the quadrate; rh pr, rhomboidal pneumatic recess; sacr, saccular recess; so sut, sutural surface for articulation with the supraoccipital; sq sut, sutural surface for articulation with the squamosal; utr, utricular recess; XII a, anterior hypoglossal foramen; XII m, middle hypoglossal foramen; XII p, posterior hypoglossal foramen

**FIGURE 19:**
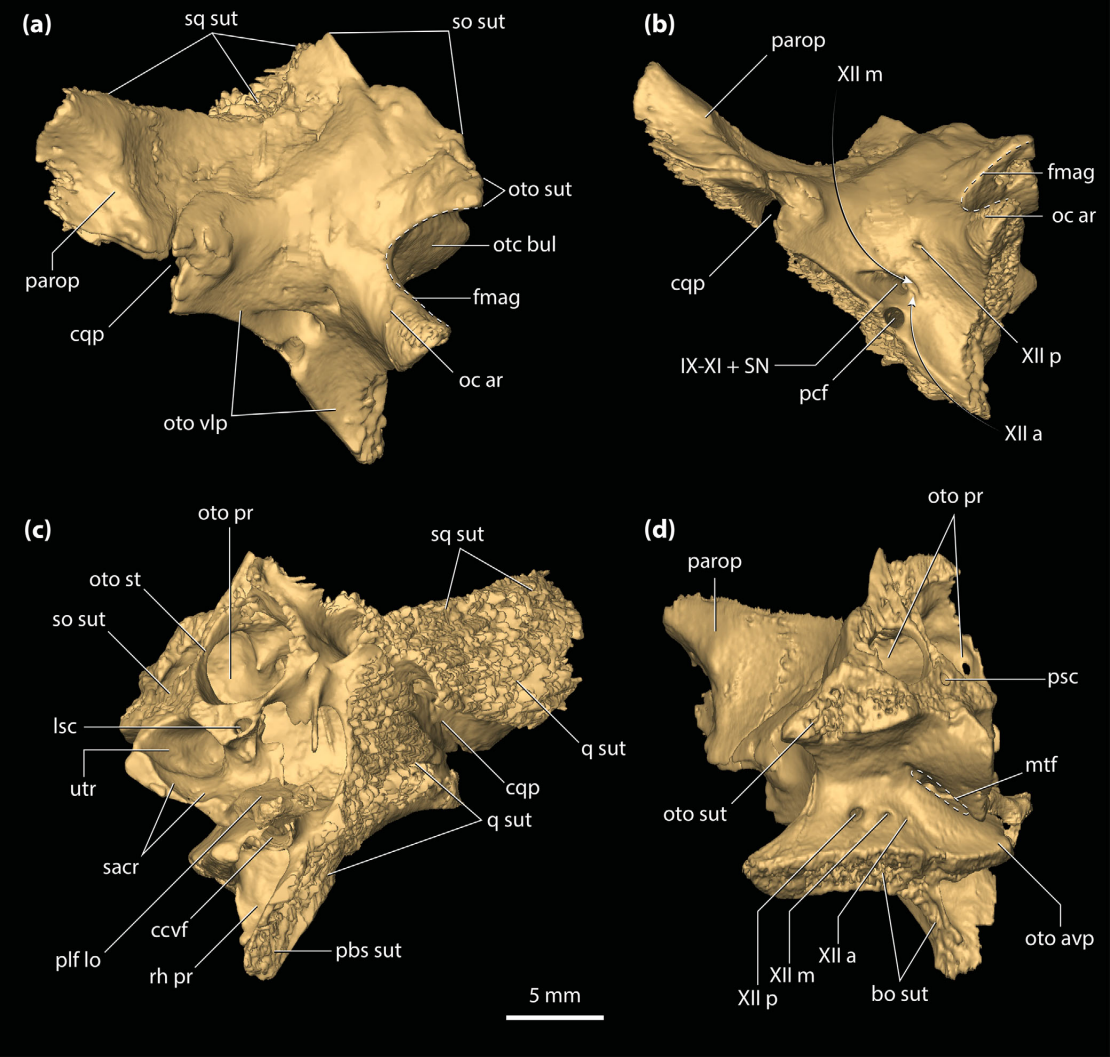
Left otoccipital of *Trilophosuchus rackhami* Willis, 1993, QMF16856, holotype. Left otoccipital in (a) posterior, (b) oblique ventral, (c) anterior, and (d) medial views. Note that the perilymphatic loop is incomplete and disarticulated. bo sut, sutural surface for articulation with the basioccipital; ccvf, cerebral carotid vasculature foramen; cqp, cranioquadrate passage; fmag, foramen magnum; lsc, lateral semicircular canal; mtf, metotic foramen; IX–XI + SN, common external foramen for the glossopharyngeal, vagus, accessory, and sympathetic nerves; oc ar, occipital arch; oto avp, anteroventral process of the otoccipital; otc bul, otic bulla; oto pr, otoccipital pneumatic recess; oto st, otoccipital strut; oto sut, sutural surface for articulation with the otoccipital; oto vlp, ventrolateral process of the otoccipital; parop, paroccipital process; pbs sut, sutural surface for articulation with the parabasisphenoid; pcf, posterior carotid foramen; plf lo, perilymphatic loop (fragment); psc, posterior semicircular canal; q sut, sutural surface for articulation with the quadrate; rh pr, rhomboidal pneumatic recess; sacr, saccular recess; so sut, sutural surface for articulation with the supraoccipital; sq sut, sutural surface for articulation with the squamosal; utr, utricular recess; XII a, anterior hypoglossal foramen; XII m, middle hypoglossal foramen; XII p, posterior hypoglossal foramen

The otoccipitals (Figures 1e, 2e, 3, 18, and 19) are well‐preserved, although they have suffered relatively minor breakages and the sutural contact between them and some of their neighboring elements has been sightly displaced. The otoccipitals (opisthotics–exoccipitals) are complex bones that comprise a significant portion of the posterior of the braincase and are the most prominent elements when the articulated skull is observed in posterior view (Figures 1e and 2e). The otoccipitals are in contact with the prootics anteriorly, the supraoccipital dorsally, the squamosals dorsolaterally, the basioccipital ventromedially, the parabasisphenoid anteroventrally, and they are also in complex sutural communication with the quadrates laterally. Finally, the two otoccipitals were in contact with each other dorsomedially via a serrated suture, although taphonomic distortion has opened the interotoccipital suture (Figure S1.5). The medial contact between the two otoccipitals dorsal to the foramen magnum that occurs via the aforementioned interotoccipital suture has a length of ~4 mm, which is more than half the dorsoventral height of the foramen magnum (character 194, state 0). Internally, the otoccipitals host extensive pneumatic recesses (Ristevski, 2022). As preserved, each otoccipital may be subdivided into the capsular portion, the anteroventral process, the ventrolateral process, the occipital arch, and the paroccipital process.

The capsular portion of the otoccipital comprises the posterior part of the ossified otic capsule that bulges medially as the otic bulla. The partition of the otic bulla composed by the otoccipital is partially visible in posterior view through the foramen magnum (Figures 1e and 2e). Contained within the otic capsule of the otoccipital is the posterior portion of the vestibular recess. Two smaller recesses can be identified within the vestibular recess—the saccular and the utricular recess—which are delimited from each other by a low and blunt ridge (Figures 18c and 19c). The canals of the posterior and lateral semicircular canals pierce the capsular portion of the otoccipital. The subcircular opening for the posterior semicircular canal (~0.7 mm in diameter on the right otoccipital; ~0.8 mm in diameter on the left otoccipital) is found dorsally on the capsular portion of the otoccipital, whereas the opening for the lateral semicircular canal is sub‐elliptical (~1 mm along major axis, and ~0.5 mm along minor axis) and situated anterolaterally. The anterior margin of the capsular portion of the otoccipital serves as a facet for articulation with the capsular portion of the prootic. However, the direct contact between the otoccipital and prootic in this location is lost due to taphonomic damage—the contact between the right otoccipital and prootic is dislocated, whereas the contact on the left side is gone due to the missing capsular portion of the left prootic. Lateral and dorsolateral to the capsular portion, the otoccipital bears several large openings leading to the otoccipital pneumatic recess within the bone.

Present anteroventrally to each otoccipital is a small and squarish fragment that appears to be a remnant of a now disarticulated portion of the otoccipital. These fragments are not detectable by external inspection of the physical specimen and can only be examined from the *μ*CT data. Both the left and right fragment are nearly identical in appearance and dimensions (~3 × 2 × 1 mm). The right fragment is largely displaced, whereas the left seems close to its correct anatomical position which would have been in articulation with the loop closure suture facet on the ventromedial margin of the capsular portion of the otoccipital. Therefore, these squarish pieces are interpreted as the broken fragments of the (now incomplete) perilymphatic loops of the otoccipitals (Figures 18c and 19c; see the interactive 3D PDF).

The anteroventral process of the otoccipital contacted the prootic, although post‐mortem displacement of these elements has disarticulated this connection. The anteroventral process is positioned medially to the fossa for the rhomboidal pneumatic recess and ventral to the capsular portion. It also bounds the ventral margin of the metotic foramen. The metotic foramen is an elongated opening located between the anteroventral process ventrally and capsular process dorsally that serves as an exit for the glossopharyngeal (cranial nerve IX), vagus (cranial nerve X), accessory (cranial nerve XI) and sympathetic nerves (abbreviated as SN) and their associated vessels. Along its major axis, the metotic foramen has a length of ~3 mm and a length of ~1 mm along its minor axis. In medial view (Figures 18d and 19d), the major axis of the metotic foramen is inclined.

The ventrolateral process is the ventral portion of the otoccipital. In occipital view, the ventrolateral process appears as a sub‐triangular ventral extension of the otoccipital that tapers to a pointed ventral tip. The ventral‐most tip of the ventrolateral process on the left otoccipital is pinched off, while the one on the right otoccipital is complete. The ventral tip of each ventrolateral process terminates on a more dorsal level relative to the ventral‐most margins of the basioccipital tuberosities (character 174, state 0; Figure 2e). Passing through the ventrolateral process are the canals for cranial nerves IX–XI, two of the three canals for the hypoglossal nerve, as well as the canals for the cerebral carotid vasculature. These canals make their exit on the occipital surface of the ventrolateral process via several foramina (Figures 18a,b and 19a,b). The largest foramen on the occipital surface of this process is the common external foramen for cranial nerves IX–XI + SN (sometimes called the foramen vagi; e.g., Iordansky, 1973; Kuzmin et al., 2021). The common external foramen for IX–XI + SN is sub‐elliptical, with its major axis being ~3 mm and its minor ~2 mm. Immediately medial to the common external foramen for IX–XI + SN are a pair of foramina related to the hypoglossal nerve. The smallest of these is the foramen related to the anterior hypoglossal canal (~0.25 mm in diameter), while dorsal to the foramen for the anterior hypoglossal canal is the slightly larger foramen related to the middle hypoglossal canal (~0.3 mm in diameter). Finally, the cerebral carotid vasculature canal exits on the occiput via the posterior carotid foramen. The posterior carotid foramen is the second largest foramen on the occipital face of the ventrolateral process. The posterior carotid foramen is subcircular and has a diameter of ~1.2 mm. On both otoccipitals, the posterior carotid foramen is located immediately ventral to the common external foramen for IX–XI + SN, and it is not distanced from the latter by any considerable length. Furthermore, the posterior carotid foramen is set at the lateral edge of the ventrolateral process. Other than these conspicuous foramina, the occipital surface of the ventrolateral process is smooth and gently concave. Along its medial edge, the ventrolateral process sutures to the basioccipital, anteroventrally it sutures to the alar process of the parabasisphenoid, and laterally and anterolaterally it bears an extensive sutural surface for contact with the quadrate. In addition, the ventrolateral process bounds the floor of the cranioquadrate passage. The anterior (or in this case, endocranial) surface of the ventrolateral process also bears a fossa/concavity related to the rhomboidal pneumatic recess.

The occipital arch of the otoccipital forms much of the medial portion of the element and bounds the lateral margin of the foramen magnum. Dorsally, the occipital arch sutures to the supraoccipital, dorsomedially to the occipital arch of the other otoccipital, and ventromedially to the basioccipital. Its medial surface is markedly concave, while ventromedially (posterior to the anteroventral process) it is pierced by three foramina related to the hypoglossal nerve—an anterior, a middle and a posterior hypoglossal foramen (Figures 18d and 19d). The hypoglossal foramina on the occipital arch are positioned in line with each other. The largest hypoglossal foramen is that of the posterior canal, which exits on the occipital surface of the occipital arch. On the occiput, the sub‐circular posterior hypoglossal foramen has a diameter of ~0.8 mm and is located dorsomedially to the common external foramen for IX–XI + SN.

The paroccipital process of the otoccipital is a prominent lateral extension with a superficially wing‐like appearance. Dorsomedially, the paroccipital process is sutured to the supraoccipital and dorsally to the squamosal. Anterolaterally, the paroccipital processes have broad sutural surfaces for contact with the quadrates, and they also bound the cranioquadrate passages dorsally.

#### 4.4.5 Basioccipital

**FIGURE 20:**
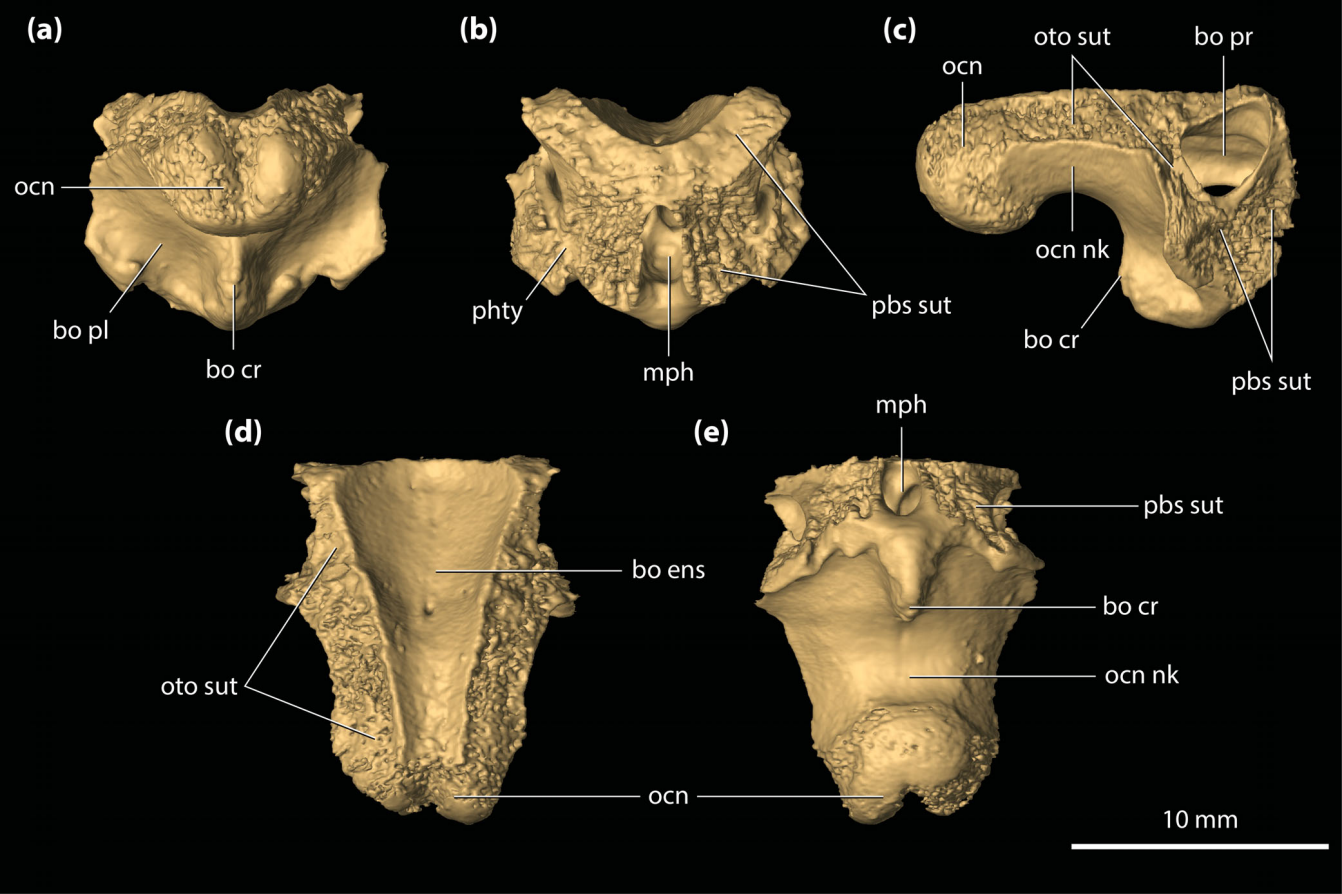
Basioccipital of *Trilophosuchus rackhami* Willis, 1993, QMF16856, holotype. Basioccipital in (a) posterior, (b) anterior, (c) right lateral, (d) dorsal, and (e) ventral views. bo cr, basioccipital crest; bo ens, endocranial surface of the basioccipital; bo pl, basioccipital plate; bo pr, basioccipital pneumatic recess; mph, median pharyngeal pneumatic canal; ocn, occipital condyle; ocn nk, neck of the occipital condyle; oto sut, sutural surface for articulation with the otoccipital; pbs sut, sutural surface for articulation with the parabasisphenoid; phty, pharyngotympanic canal

Forming the posterior portion of the basicranium is the basioccipital (Figures 1, 2, 3 and 20). The basioccipital is excellently preserved and without any (or at most, negligible) deformities or breakages. In the articulated skull, the basioccipital has its posterior surface fully exposed, while its ventral surface is exposed to a small degree. Also, the articulated skull allows some observation of the basioccipitals' endocranial surface through the foramen magnum. The digital disarticulation of the cranium reveals that anteriorly the basioccipital is in contact with the parabasisphenoid, and with the otoccipitals laterally and dorsolaterally. The basioccipital has a maximum length of ~14 mm, maximum height of ~10 mm, and maximum width of ~12 mm. Internally, the basioccipital hosts an expansive pneumatic recess (Ristevski, 2022). This element may be subdivided into the endocranial surface of the basioccipital, the occipital condyle, and the basioccipital plate.

The endocranial (or in this case, dorsal) surface of the basioccipital forms the posterior floor of the endocranial cavity. It is notably concave, and laterally it is bounded by the sutural surfaces for articulation with the otoccipitals. The concave endocranial surface is smooth and devoid of protuberances or crests, such that may develop in some individual crocodylians (Ristevski et al., 2020a), although several tiny foramina are dispersed over it (Figure 20d). The endocranial surface is widest anteriorly (~7 mm) and gradually narrows posteriorly toward the occipital condyle, with its narrowest portion corresponding with the ventromedial margin of the foramen magnum (~2 mm). Upon observation of the sagittally sectioned basioccipital, it is apparent that its endocranial surface has a mildly undulating profile, with the surface over the occipital condyle neck being on a slightly more dorsal level relative to the surface over the anterior half of the basioccipital (Figure 3).

The sub‐spherical occipital condyle is oriented posteriorly and sits on a relatively long neck (Figure 20a,c). The neck of the occipital condyle is sub‐horizontal and has a length of ~8.5 mm, which is 61% of the total length of the basioccipital. The occipital condyle itself has smaller dimensions than those of the foramen magnum (~7 mm in transverse width and ~5 mm in dorsoventral height). The condyle's surface is relatively smooth, being slightly coarser than the other external surfaces of the basioccipital. A shallow and narrow sulcus runs vertically on the surface of the occipital condyle. Unlike the anterior half of the basioccipital (i.e., the precondylar portion of the element), the occipital condyle neck and the occipital condyle (i.e., the condylar portion of the basioccipital) are not affected by the paratympanic pneumatic diverticula.

Anteriorly, the basioccipital plate is firmly sutured to the parabasisphenoid (specifically, to the descending lamina of the latter). The anterodorsal portion of the surface that sutures to the parabasisphenoid is vertical, whereas more ventrally the sutural surface is inclined as it adopts an anteroventral orientation. Together with the parabasisphenoid, the basioccipital bounds the walls of the pharyngotympanic and median pharyngeal canals. Specifically, the basioccipital forms the posterior walls of the pharyngotympanic and median pharyngeal canals, whereas the parabasisphenoid bounds their anterior walls. Opening anteromedially on the basioccipital is the posterior wall of the median pharyngeal canal (Figure 20b). The opening of the median pharyngeal canal on the basioccipital is sub‐vertical and oval, and terminates ventrally as the median pharyngeal tube foramen (sensu Young & Bierman, 2019; =ostium for the median pharyngeal sinus sensu Dufeau & Witmer, 2015). The basioccipital bounds the foramen's posterior and lateral margins, whereas the anterior margin of the foramen is formed by the parabasisphenoid. The median pharyngeal tube foramen is sub‐circular and has a diameter of ~2 mm. The posterior walls of the pharyngotympanic canals course anterolaterally on the basioccipital, being located ~3 mm lateral to the median pharyngeal canal. The pharyngotympanic canals are relatively narrow (~1.5 mm) and exit the basicranium ventrally through the pharyngotympanic foramina. Similarly to the median pharyngeal tube foramen, the pharyngotympanic foramina have their posterior margins bound by the basioccipital and their anterior by the parabasisphenoid. The pharyngotympanic foramina are elliptical, having a length of ~2 mm along their major axes and <1 mm along their minor axes. Anterolaterally, the basioccipital bears a pair of large (~4 mm) sub‐circular openings, one on each side (Figure 20c). These are the openings for the basioccipital pneumatic recess (=communicating ostia between the pharyngotympanic sinus and basioccipital diverticulum sensu Dufeau & Witmer, 2015).

The posterior (or, occipital) surface of the basioccipital plate is fully exposed in occipital view of the articulated skull. It has a height of ~5 mm, which is sub‐equal to the height of the occipital condyle. The posterior surface of the basioccipital has a posteroventral orientation (character 168, state 0). Its lateral margins are not parallel to each other, but are oriented in a manner that they converge ventromedially. Developed at the center of the posterior surface is a sizeable basioccipital crest (=basioccipital eminence sensu Young & Bierman, 2019; midline, or median crest of the basioccipital), with the surface lateral to the crest being smooth and faintly concave. The basioccipital crest is vertical and blunted, with a thickness of ~1 mm and dorsoventral height of ~3 mm. The crests' ventral margin extends almost to the same level anterior to the median pharyngeal tube foramen, and thus forms the ventral‐most margin of the basioccipital (Figure 20a). The basioccipital tuberosities (or, basioccipital tubera) in *T. rackhami* are not prominent (unlike in certain gavialoids, for example). In posterior view, the ventral margins of the weakly developed basioccipital tuberosities are separated from the more medial portions of the basioccipital plate by the notch‐like appearance of the pharyngotympanic foramina (Figure 20a). The posteroventral margins of the basioccipital plate, including the margins of the basioccipital tuberosities are slightly thickened and project marginally more posteriorly than the rest of the occipital surface (although they do not come close to projecting as posteriorly as the basioccipital crest). Measured transversely at the basioccipital tuberosities, the width of the basioccipital is ~11 mm.

#### 4.4.6 Parabasisphenoid

**FIGURE 21:**
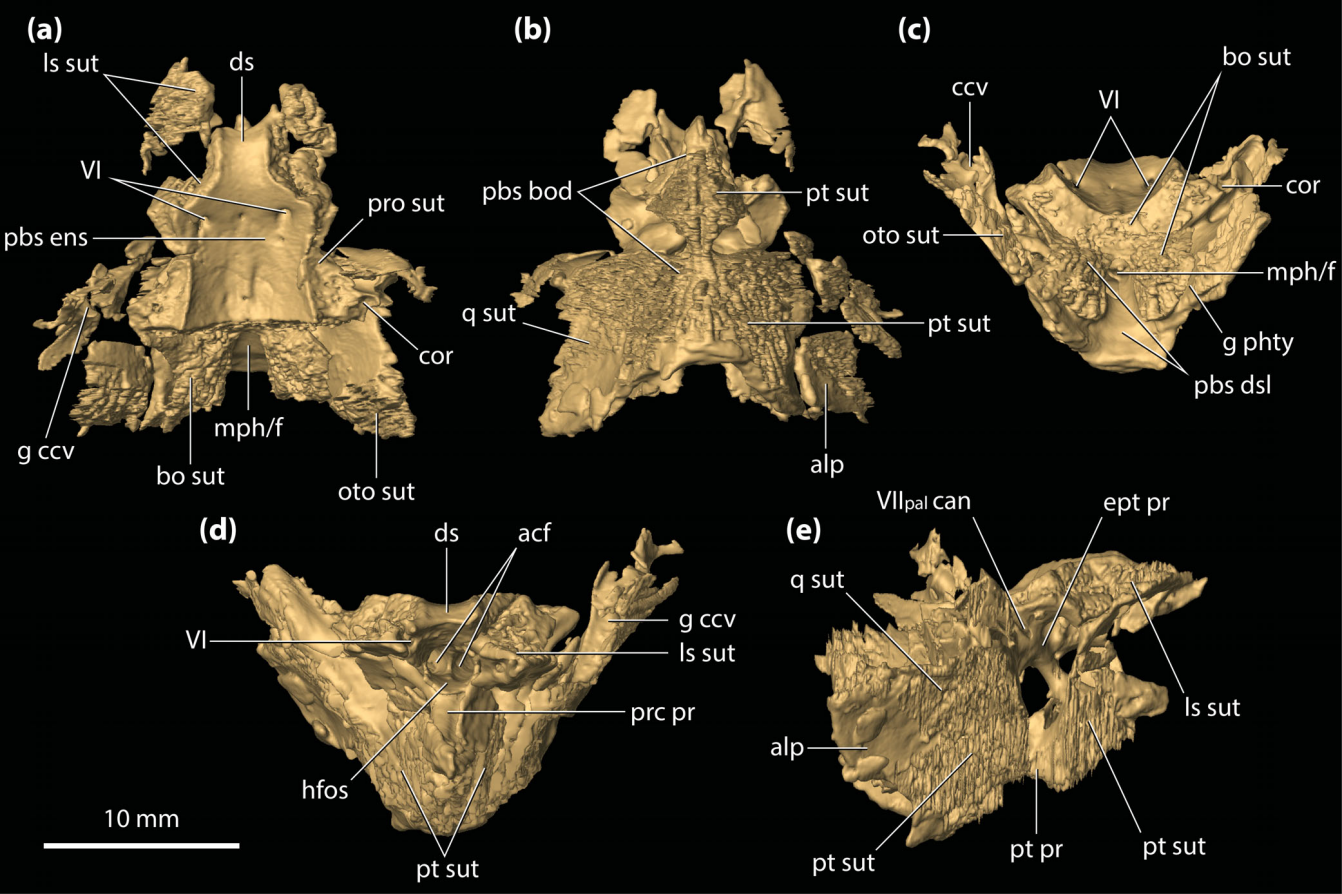
Parabasisphenoid of *Trilophosuchus rackhami* Willis, 1993, QMF16856, holotype. Parabasisphenoid in (a) dorsal, (b) ventral, (c) posterior, (d) anterior, and (e) right lateral views. Note that the parabasisphenoid is imperfectly preserved and incomplete. acf, anterior carotid foramina; alp, alar process; bo sut, sutural surface for articulation with the basioccipital; ccv, cerebral carotid vasculature canal; cor, cochlear recess; ds, dorsum sellae; ept pr, pneumatic recessus epitubaricus; g ccv, groove for the cerebral carotid vasculature canal; g phty, groove for the pharyngotympanic canal; hfos, hypophyseal fossa; ls sut, sutural surface for articulation with the laterosphenoid; mph/f, median pharyngeal canal/foramen; oto sut, sutural surface for articulation with the otoccipital; pbs bod, body of the parabasisphenoid; pbs ens, endocranial surface of the parabasisphenoid; pbs dsl, descending lamina of the parabasisphenoid; prc pr, precarotid pneumatic recess; pro sut, sutural surface for articulation with the prootic; pt pr, pneumatic pterygoid recess; pt sut, sutural surface for articulation with the pterygoid; q sut, sutural surface for articulation with the quadrate; VI, abducens foramina; VII_pal_ can, canal for the palatine branch of the facial nerve

The parabasisphenoid is mostly complete but imperfectly preserved (Figures 2b–e, 3, and 21). The parabasisphenoid has suffered several fractures, relatively mild deformities, and some portions, such as the parabasisphenoid rostrum (=cultriform process), are missing. Together with the basioccipital, the parabasisphenoid forms the floor of the braincase, with the parabasisphenoid occupying the anterior portion of the basicranium. In the articulated skull, the parabasisphenoid is largely hidden from view by its neighboring elements, being partially visible when the basicranium is observed in posterior, ventral, and lateral views. Also, the articulated skull allows some observation of the parabasisphenoids' endocranial surface through the foramen magnum. Several bones are in contact with the parabasisphenoid: anterodorsally, the parabasisphenoid contacts the laterosphenoids, ventrally the pterygoid, dorsomedially the prootics, dorsolaterally the quadrates, posterolaterally the otoccipitals, and posteriorly the basioccipital. Internally, the parabasisphenoid is hollowed by pneumatic recesses (Ristevski, 2022). The parabasisphenoid may be subdivided into the body (or central part), the posterior descending lamina, and the posterolateral alar processes. The crocodylian parabasisphenoid also includes a fourth subdivision, the rostrum (Kuzmin et al., 2021), although it is not preserved in the *T. rackhami* holotype.

The main component of the parabasisphenoid is the body. In lateral view of the basicranium, the body of the parabasisphenoid is almost entirely hidden from view by the pterygoid processes of the quadrates and the ascending process of the pterygoid. Nonetheless, relatively small anterior sections of the body are still exposed anterior to the ascending process of the pterygoid and ventral to the laterosphenoids (character 161, state 1; Figure 2c,d). However, as this anterolaterally exposed portion is fractured, interpreting its superficial features (such as the presence or absence of a sulcus; character 160) is hampered. Much of the lateral surfaces of the body serve to contact the quadrates and the pterygoid. Additionally, as preserved the lateral surfaces of the parabasisphenoid expose the cavities for the pterygoid pneumatic recesses and the recessus epitubaricus (Figure 21e). At the anterior of the body is the hypophyseal (pituitary) fossa (Figure 21d). The base of the parabasisphenoid rostrum bounds the fossa ventrally whereas the dorsum sellae bounds the fossa posteriorly and dorsally. The hypophyseal fossa has incomplete outer rims, although what is preserved depicts a circular external outline that is ~3 mm in diameter. The fossa is quite deep, and within it are the anterior carotid foramina. The cerebral carotid vasculature canals coursed through the parabasisphenoid, with some of the canals still present on the element, albeit somewhat poorly preserved.

Dorsally on the body of the parabasisphenoid is its concave endocranial surface (Figure 21a). The ventral surface of the braincase/endocranial cavity, or the dorsal/endocranial surface of the basicranium is completed by the endocranial surfaces of the parabasisphenoid and basioccipital. The total preserved length of the braincase floor is ~23 mm (as measured from the foramen magnum posteriorly to the dorsum sellae anteriorly), with the endocranial surface of the basioccipital having a length of ~13 mm (measured from the foramen magnum to the parabasisphenoid‐basioccipital suture) whereas the endocranial surface of the parabasisphenoid is ~10 mm (measured from the parabasisphenoid‐basioccipital suture to the dorsum sellae). This indicates that the basioccipital comprises more of the braincase floor length as opposed to the parabasisphenoid (~56% vs. ~44%, respectively). Compared to the holotype basicranium (QMF59017) of *Paludirex vincenti* Ristevski et al., 2020a, the parabasisphenoid of *T. rackhami* (QMF16856) has a proportionally longer endocranial surface (see Ristevski et al., 2020a). In QMF16856, the endocranial surface of the parabasisphenoid is smooth and markedly concave at its posterior. More anteriorly, as it transitions to the dorsal surface of the dorsum sellae, the parabasisphenoid becomes more convex, giving the endocranial surface an inclined profile (Figure 3). As per Ristevski et al. (2020a), the endocranial surface of the parabasisphenoid in QMF16856 bears a low and blunt sagittal crest (Figure 21a). This faint crest is ~3 mm long and ~0.5 mm wide, and anterior and posterior to it are two small foramina that are roughly 0.2 mm in diameter. The crest is present only on the endocranial surface of the parabasisphenoid and does not transition over to the basioccipital like in *P. vincenti* (QMF59017). Sagittally aligned basicranial crests occasionally occur in some individual crocodylians of varying ontogenetic stages [e.g., OUVC 10606 a hatchling *Alligator mississippiensis* (Daudin, 1802), see Dufeau & Witmer, 2015; QMF59017, an adult individual of *P. vincenti*], however, they are unlikely to be of diagnostic value (see “Basioccipital” subsection in Ristevski et al., 2020a for more details). Anterolateral to the aforementioned crest are the paired abducens foramina. The abducens foramina are fairly conspicuous, having diameters of ~0.6 mm. These foramina lead into the abducens canals that course through the parabasisphenoid. The abducens canals exit anteriorly on the parabasisphenoid via a second, anterior pair of abducens foramina located dorsolaterally to the hypophyseal fossa (the abducens foramen and canal on the left is not as complete as its right‐side counterpart). Another pair of canals, those related to the palatine branches of the facial nerves, also course through the parabasisphenoid and are partially visible in lateral view of the body (Figure 21e). Dorsally, the body establishes its contact with the laterosphenoids, prootics, and dorsolaterally with the quadrates. At the posterodorsal margins of the body, at the junction that continues as the descending lamina, are a pair of shallow concavities that correspond to the floor of the cochlear recesses (Figure 21a,c).

Continuing posteroventrally from the body is the descending lamina of the parabasisphenoid (Figure 21c). Much of the descending lamina serves as a sutural surface for articulation with the basioccipital. The dorsal portion of the descending lamina is vertical, but as it progresses ventrally it becomes inclined. In the center of the descending lamina is the opening for the median pharyngeal canal. The parabasisphenoid bounds the anterior of the median pharyngeal canal and median pharyngeal (tube) foramen. More laterally, the descending lamina also bounds the anterior walls of the pharyngotympanic canals. Ventrally, the descending lamina terminates its sutural contact with the basioccipital and adopts a smooth surface that is exposed in posterior view of the skull (ventral to the basioccipital and median pharyngeal foramen) for a preserved dorsoventral height of ~1.5 mm (Figures 2e and 21c). In ventral view, the parabasisphenoid has conspicuous exposure between the basioccipital and pterygoid (character 170, state 1; Figures 2b and 21b). Here, the exposure of the parabasisphenoid adopts a semi‐lunar appearance, with its maximum exposed anteroposterior length being ~2 mm.

Continuing from the lateral surface of the parabasisphenoid and projecting posterolaterally are the alar processes. Each alar process is exposed on the posterolateral surface of the braincase, being exposed for an anteroposterior length of ~4 mm and dorsoventral height of ~7 mm (~6 mm on the right side). The alar processes are mediolaterally thin segments of the bone, with their posteromedial surfaces contacting the ventrolateral processes of the otoccipitals. On their lateral sides, the alar processes are in contact with the pterygoid processes of the quadrates anterodorsally, and anteroventrally they contact the ascending process of the pterygoid.

### 4.5 Splanchnocranial bones

#### 4.5.1 Quadrates

**FIGURE 22:**
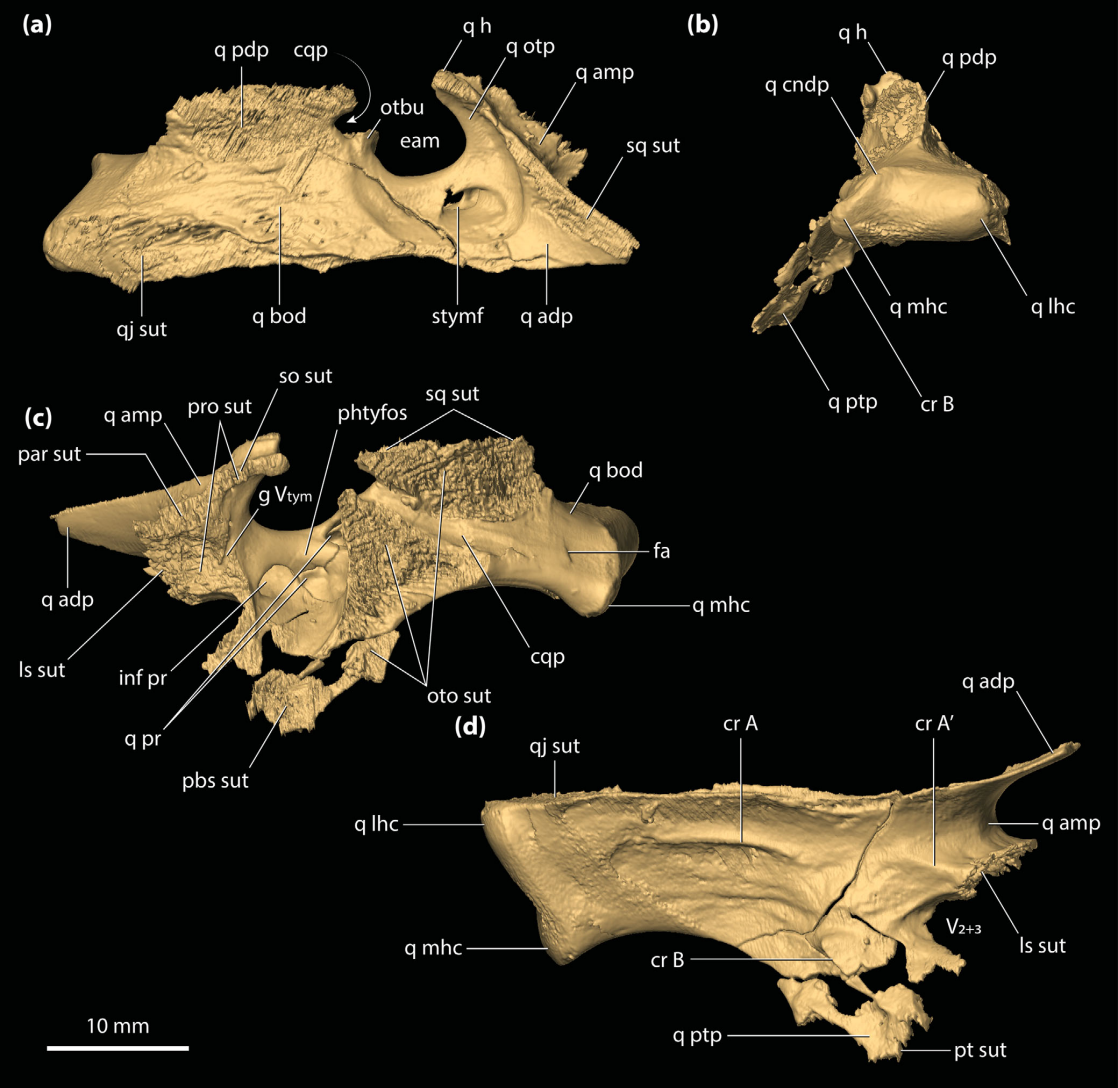
Right quadrate of *Trilophosuchus rackhami* Willis, 1993, QMF16856, holotype. Right quadrate in (a) dorsal, (b) posterior, (c) medial, and (d) ventral views. Note that the pterygoid process of the quadrate is poorly preserved. cqp, cranioquadrate passage; cr A, crest A of quadrate; cr A', crest A' of quadrate; cr B, crest B of quadrate; eam, external auditory meatus; fa, foramen aëreum (pneumatic siphonium); g V_tym_, groove for the tympanic branch of the trigeminal nerve; inf pr, infundibular pneumatic recess; ls sut, sutural surface for articulation with the laterosphenoid; otbu, otic buttress; oto sut, sutural surface for articulation with the otoccipital; par sut, sutural surface for articulation with the parietal; pbs sut, sutural surface for articulation with the parabasisphenoid; phtyfos, pharyngotympanic fossa; pro sut, sutural surface for articulation with the prootic; pt sut, sutural surface for articulation with the pterygoid; q adp, anterodorsal process of the quadrate; q amp, anteromedial process of the quadrate; q bod, body of the quadrate; q cndp, dorsal projection between the lateral and medial hemicondyles of the quadrate; q h, head of the quadrate; q lhc, lateral hemicondyle of the quadrate; q mhc, medial hemicondyle of the quadrate; q otp, otic process of the quadrate; q pdp, posterodorsal process of the quadrate; q pr, quadrate pneumatic recess; q ptp, pterygoid process of the quadrate; qj sut, sutural surface for articulation with the quadratojugal; so sut, sutural surface for articulation with the supraoccipital (short contact); sq sut, sutural surface for articulation with the squamosal; stymf, subtympanic foramen; V_2+3_, maxillomandibular foramen

**FIGURE 23:**
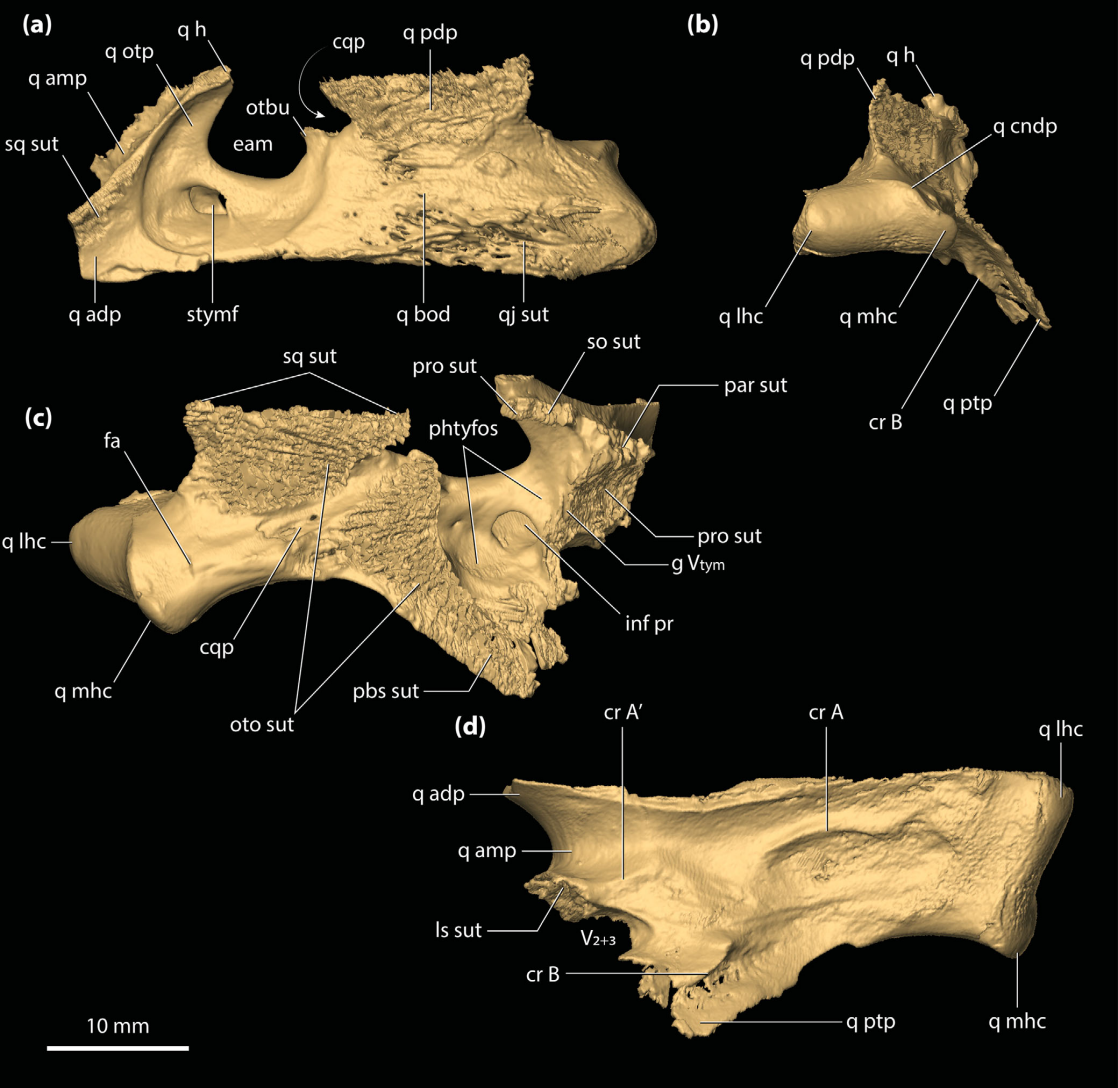
Left quadrate of *Trilophosuchus rackhami* Willis, 1993, QMF16856, holotype. Left quadrate in (a) dorsal, (b) posterior, (c) medial, and (d) ventral views. Note that the anterodorsal process of the quadrate is missing its tip, and the pterygoid process of the quadrate is poorly preserved. cqp, cranioquadrate passage; cr A, crest A of quadrate; cr A', crest A' of quadrate; cr B, crest B of quadrate; eam, external auditory meatus; fa, foramen aëreum (pneumatic siphonium); g V_tym_, groove for the tympanic branch of the trigeminal nerve; inf pr, infundibular pneumatic recess; ls sut, sutural surface for articulation with the laterosphenoid; otbu, otic buttress; oto sut, sutural surface for articulation with the otoccipital; par sut, sutural surface for articulation with the parietal; pbs sut, sutural surface for articulation with the parabasisphenoid; phtyfos, pharyngotympanic fossa; pro sut, sutural surface for articulation with the prootic; q adp, anterodorsal process of the quadrate; q amp, anteromedial process of the quadrate; q bod, body of the quadrate; q cndp, dorsal projection between the lateral and medial hemicondyles of the quadrate; q h, head of the quadrate; q lhc, lateral hemicondyle of the quadrate; q mhc, medial hemicondyle of the quadrate; q otp, otic process of the quadrate; q pdp, posterodorsal process of the quadrate; q ptp, pterygoid process of the quadrate; qj sut, sutural surface for articulation with the quadratojugal; so sut, sutural surface for articulation with the supraoccipital (short contact); sq sut, sutural surface for articulation with the squamosal; stymf, subtympanic foramen; V_2+3_, maxillomandibular foramen

The quadrates (Figures 1, 2, 3, 22, 23, S1.11, and S1.12) are relatively well‐preserved and almost complete. They are complex components of the braincase that are in contact with multiple elements. The multifaceted morphology of the quadrate may be sectioned into several parts: the otic process and the head, the anterodorsal process, the anteromedial process, the pterygoid process, the body of the quadrate along with the quadrate hemicondyles, and the posterodorsal process. Internally, the quadrates possess pneumatic cavities (Ristevski, 2022).

The otic process is complete in both the left and right quadrate. It is a relatively short and posterodorsally arched process that bounds the anterior margin of the external auditory meatus (i.e., the semilunar otic incisure) and terminates into the quadrate head. Along their medial margin, the otic process and quadrate head suture to the prootic buttress, whereas laterally they establish a relatively short (~2 mm in length) sutural contact with the squamosal.

The right quadrate has a completely preserved anterodorsal process, whereas the anterodorsal process of the left quadrate has its anterior tip broken off. Located anterior to the periotic fossa (sensu Montefeltro et al., 2016), the anterodorsal process extends further anteriorly than any other component of the quadrate. It has a sub‐triangular appearance when observed in lateral and medial aspects as it gradually tapers anteriorly. Furthermore, the anterodorsal process of the quadrate is strongly compressed lateromedially. Along its dorsolateral margin, the anterodorsal process of the quadrate bears a sutural surface for articulation with the anterior descending lamina of the squamosal. On the lateral surface of the quadrate, posterior to the anterodorsal process lies the periotic fossa, which is a relatively shallow yet noticeable concavity around the external auditory meatus.

Anteriorly continuous from the otic process and the body of the quadrate, and posteromedial to the anterodorsal process is the anteromedial process of the quadrate. The anteromedial process of the quadrate forms the posteroventral wall of the supratemporal fossa, and when the articulated cranium is observed in dorsal view the anteromedial process is largely exposed through the supratemporal fenestra (Figure 2a). Dorsally, the anteromedial process of both the left and right quadrate has its otherwise smooth surface covered by several small foramina. Medially, the anteromedial process sutures to several elements of the braincase, which include the laterosphenoid body (anteriorly), the prootic (medially), as well as the descending process of the parietal (anterodorsally). Additionally, the anteromedial process of the quadrate in *T. rackhami* establishes short contact with the lateral margin of the supraoccipital's dorsal lamina. This contact with the supraoccipital (partially visible in the fully articulated skull; e.g., Figures 2a and S1.3) occurs immediately posterior to the contact with the descending process of the parietal. The anteromedial process of the quadrate bounds the posterior margin of the maxillomandibular foramen, and on its ventral surface it bears the crest A' of the quadrate (Figure 22d and 23d). The muscular crest A' is well‐developed, although it is the least acute of the crests that spread on the ventral surface of the quadrate. Crest A' has a relatively short anteroposterior length (~3.5 mm) and is comparatively blunter than the other quadrate crests.

The ventrally projecting pterygoid processes are the most poorly preserved portions of the quadrates, with the pterygoid process of the right quadrate being largely incomplete. Nevertheless, what is preserved of the pterygoid processes is still quite informative. Ventrally, the pterygoid process of the quadrate is sutured to the ascending process of the pterygoid, ventrolateral process of the otoccipital, and the alar process of the parabasisphenoid. Also, it bounds the ventral margin of the maxillomandibular foramen. The muscular crest B lies on the pterygoid process and is expressed as a very sharp ridge. In posterior view, the pterygoid process of the quadrate has minimal visibility, with crest B being barely discernable at best (Figure 2e; character 212, state 0). This contrasts with some other mekosuchines where the pterygoid process of the quadrate is largely exposed in posterior view of the skull (e.g., *Baru*, *P. vincenti*, *Q. timara* Megirian, 1994; see Document S1 for more detailed morphological comparisons).

The main component of the quadrate is the body. In the *T. rackhami* holotype, the body of the quadrate is robust but relatively short. The length of the body as measured from the posterior tip of the paroccipital process of the otoccipital to the quadrate condylar margin (~4 mm) is shorter than the width of the quadrate body as measured at the quadrate hemicondyles (~12 mm; character 213, state 0). Laterally, the body is in sutural contact with the quadratojugal for most of its length. There are no obvious crests that adorn the dorsal surface of the quadrate body (character 177, state 0), but found on its ventral surface is the elongated and relatively sharp crest A (Figures 22d and 23d). The sharp muscular crest A has a length of ~9 mm and is relatively straight for its entire span bar its subtle medial curvature. On both quadrates, crest A is situated more closely to the lateral (a distance of ~3 mm) than to the medial margin (a distance of ~7 mm) of the quadrate body. Other than the prominent crest A, the quadrate body has a relatively flat to gently concave ventral surface.

In posterior view, the condylar surface of the quadrate (=the distal surface of the quadrate for articulation with the mandible) is relatively flat and subtly convex, with a mildly coarse texture that is not too dissimilar to the surface texture of the occipital condyle (Figure S1.11). The intercondylar groove is very faint, which makes the limit between the lateral and medial hemicondyles somewhat arbitrary. The lateral hemicondyle is larger than the medial (lateral hemicondyle = ~5 mm dorsoventral height and ~7 mm transverse width; medial hemicondyle = ~4 mm dorsoventral height and ~3.5 mm transverse width). When observed in a fully articulated skull, it is evident that the medial hemicondyle is positioned marginally more posterior than the lateral hemicondyle. Also obvious in posterior view, the condylar surface of the quadrate is aligned with the occipital condyle (Figures 1e and 2e; character 214, state 0). For most of their length, the dorsal and ventral margins of the condylar surface are subparallel with each other such that the overall condylar outline is sub‐rectangular (character 245, state 0). The dorsal condylar margin is relatively straight for most of its length, culminating in a blunt and low dorsal projection (character 179, state 1). This dorsal projection is positioned closer to the medial hemicondyle than to the lateral (Figures 22b, 23b, S1.11, and S1.12). Further medially, the dorsal condylar margin begins to suddenly slope in a ventromedial direction over the medial hemicondyle.

Each quadrate bears a singular opening for the pneumatic siphonium—the foramen aëreum (Figure S1.11). The foramen aëreum is small (~1 mm transverse width; character 176, state 0) and located mediodorsally on the body of the quadrate (~1 mm distance from the medial margin of the foramen to the medial edge of the quadrate; character 175, state 0). The foramen aëreum is located relatively close to the medial hemicondyle and is set ~3 mm posterior to the dorsal condylar margin.

Arising at the ventral margin of the external auditory meatus is the otic buttress. The otic buttress is a relatively small structure that is merely ~1 mm tall. Posterodorsal to the otic buttress is the anterodorsal opening for the cranioquadrate passage. Additionally, posterior to the otic buttress is a prominent dorsal projection from the quadrate body—the posterodorsal process of the quadrate. The posterodorsal process is a relatively tall (~4 mm) and subvertical extension that laterally sutures to the posterolateral descending lamina of the squamosal and medially to the otoccipital.

Medially, much of the quadrates' surface is dedicated to establishing sutural articulation with adjacent bones (Figures 22c and 23c). However, also found on the medial surface of the quadrate is a wide concave area that corresponds to the pharyngotympanic fossa, and there are several large openings that lead into the pneumatic recesses that invade the inside of the quadrate. Additionally, the lateral wall of the cranioquadrate passage is evident as a long and mild concavity set ventromedially to the posterodorsal process.

### 4.6 Dentition

The limited inferences on the dentition of the *T. rackhami* holotype are based on what is preserved of its maxillary alveoli. Neither the holotype nor any referred specimens of *Trilophosuchus* preserve teeth, preventing a description of their morphology.

The maxillary alveoli of QMF16856 have suffered taphonomic distortion and breakage such that no alveolus is complete. On both the left and right side, the last three alveoli are preserved best and have sub‐circular outlines (Figures 1b, 2b, and 4a,b; character 79, state 0). The last three alveoli are not entirely bound by the maxilla as the ectopterygoid bounds their lingual margins. Due to poor preservation, accurate measurements of the alveolar dimensions are hampered. Nevertheless, the last three alveoli have slightly different mesiodistal lengths which indicates that *T. rackhami* has some degree of size differentiation between the alveoli. Another ambiguity concerns the exact number of maxillary alveoli. Because the maxillae are incomplete, determining the precise position of each alveolus is uncertain. Based on our interpretation in Figure 4a,b, we tentatively infer the total number of alveoli in each maxilla to be either 12 or 13 (character 221, state 1). The alveolar walls are nondescript, other than mentioning that they are somewhat rugose. There is little to no interalveolar space between the alveoli, and there is no evident scalloping between them either. The dentition of *T. rackhami* was partially interlocking, as evident by a prominent notch that received a dentary tooth that is exposed labially on the right maxilla (Figure 4a,c). The singular notch for receiving a dentary tooth during jaw occlusion is located somewhere between the fifth and ninth maxillary alveoli (more specifically, its possible position may be between the seventh and eighth alveoli; character 92, state 1). The equivalent portion on the left maxilla that bore the dentary tooth notch is missing.

## 5 REMARKS ON THE MORPHOLOGICALLY MATURE AND IMMATURE FEATURES OF THE *TRILOPHOSUCHUS RACKHAMI* HOLOTYPE SPECIMEN (QMF16856)

Willis (1993) noticed that the *T. rackhami* holotype displays a combination of both morphologically mature and immature features. The features listed by Willis (1993) as typical of morphologically immature crocodylians were: the interconnected last two or three maxillary alveoli, relative size of the trigeminal foramen, relative size of the foramen magnum, the inclination of the basioccipital plate, morphology of the supratemporal fenestrae, and the overall small size of the cranium. On the other hand, Willis (1993) also pointed out that the prominent cranial sculpturing and the strongly sutured cranial elements are suggestive of a mature individual. Here, we review the morphologically mature and immature features of the holotype cranium that were originally listed by Willis (1993), but we also comment on previously undiscussed features. We also consider some of the criteria for assessing maturity in saurian reptiles as proposed by Griffin et al. (2021): cranial suture fusion, cranial morphology, cranial ossification, cranial dermal bone texture, and number of teeth/alveoli.

### 5.1 Cranial suture fusion

Willis (1993) remarked that the cranial elements of QMF16856 are strongly sutured together. We agree with this observation, however we also note that certain elements (e.g., left postorbital–frontal suture; prootic‐otoccipital suture; right quadratojugal and quadrate suture; suture between the otoccipitals) are in loose contact. This appears to be a result of taphonomic distortion. For example, the right postorbital is strongly sutured to the frontal and parietal, whereas the left postorbital is in a looser contact and is slightly displaced. If the cranial elements were not strongly sutured, it would be expected that the right postorbital would have a similarly loose contact as the left. However, that is clearly not the case. Furthermore, as deduced from the μCT data, some internal braincase elements have established such sutural relations that delimiting them proved challenging (e.g., laterosphenoids and prootics; otoccipitals and squamosals; ventral sutural portions between the basioccipital and parabasisphenoid; quadrates and laterosphenoids and prootics). Indeed, the cranium of QMF16856 has been crushed during fossilization, and substantial reparation had to be administered to it before being studied by Willis (1993).

Nevertheless, cranial sutural fusion alone is a poor indicator of morphological maturity in crocodylians. For example, in the extant *Alligator mississippiensis*, Bailleul et al. (2016) found that the cranial sutures become more separated in large individuals than they are in younger members of that species. Brochu (1997) and Brochu & Gingerich (2000) also expressed doubt on the reliability of cranial sutural fusion as the only indicator of morphological maturity in crocodylians. Instead, these authors suggested that the anterior ossification of the laterosphenoids is a more reliable method of assessing maturity in extinct taxa. In extant crocodylians, the anterior portion of the braincase as formed by the anterior processes of the laterosphenoids is unossified early in ontogeny (Kuzmin et al., 2021). Unfortunately, the anterior processes of the laterosphenoids in QMF16856 are poorly preserved and incomplete. What little remains of the anterior laterosphenoid process on the right side indicates that the anterior of the braincase was poorly ossified. If this was indeed the case, then this would be a morphologically immature feature.

In summation, all well‐preserved cranial elements of QMF16856 are strongly sutured. Cranial elements that may appear slightly disarticulated or in loose contact with adjacent bones are a result of post‐mortem distortion, damage, and/or a consequence of the fossil reparation for the specimen. Therefore, the cranial sutural fusion of QMF16856 is consistent with the morphologically mature condition. However, the ossification at the anterior of the braincase, as preserved, seems to be morphologically immature. Unfortunately, the degree of ossification at the anterior of the braincase cannot be assessed with certainty in QMF16856.

### 5.2 Cranial morphology

In QMF16856, the width of the foramen magnum is greater than that of the occipital condyle. In morphologically immature crocodylians, the occipital condyle is smaller than the foramen magnum, but in adults they become sub‐equal in width. According to Jouve (2017), the occipital condyle in small‐bodied extant crocodylians like *Paleosuchus palpebrosus* Cuvier, 1807 and *Osteolaemus tetraspis* Cope, 1861 remains smaller than the foramen magnum even at adulthood. Importantly, Jouve (2017) stated that there was not enough data at the time to conclude if this is a consistent feature in these small‐bodied taxa. Based on our observations of digital skull models of *Pa. palpebrosus* and *O. tetraspis* (available as supplementary to Sookias, 2019), the occipital condyle seems to be sub‐equal in width to the foramen magnum. Therefore, even in *Pa. palpebrosus* and *O. tetraspis*, the foramen magnum and occipital condyle attain sub‐equal dimensions at some point during ontogeny. It is unclear if *Pa. palpebrosus* and *O. tetraspis* retain an occipital condyle that is smaller than the foramen magnum later in ontogeny than other crocodylians; assessment of more specimens for these taxa is needed in order to clarify this issue. Unfortunately, none were available to us for first‐hand examination. In sum, the smaller size of the occipital condyle relative to the foramen magnum of QMF16856 is a morphologically immature feature.

The supratemporal fenestrae have variable outlines and sizes that differ between taxa (e.g., figure S1.14 in Ristevski et al., 2020b). The outline and size of the supratemporal fenestrae within a species is also subject to ontogenetic change (Cossette et al., 2021). Typically, hatchling and juvenile crocodylians have supratemporal fenestrae that are sub‐elliptical in outline and have their main axes diverging laterally (Kälin, 1933; Iordansky, 1973; Dodson, 1975; Clark & Norell, 1992; see figure 8 in Fernandez Blanco et al., 2018). The holotype of *T. rackhami* has relatively large and elliptical supratemporal fenestrae that have their main axes aligned parasagittally (Figures 1a and 2a). Therefore, the overall condition of the supratemporal fenestrae in QMF16856 is morphologically mature.

One of the diagnostic features of *T. rackhami* is a cranial table that slopes lateroventrally from the sagittal plane at maturity (Figures 1e, 2e, and S1.2A). In hatchling crocodylians, the cranial table slopes lateroventrally from the sagittal axis (Brochu, 1997; Brochu & Gingerich, 2000; Mook, 1921), however, in post‐hatchling ontogeny the cranial table becomes planar. Since QMF16856 is demonstrably not a hatchling individual, it can be concluded that *T. rackhami* has retained this paedomorphic condition in its post‐hatchling ontogeny. Like *T. rackhami*, a nonplanar cranial table is also present at maturity in some gavialoids and the non‐crocodylian eusuchian *Hylaeochampsa vectiana* Owen, 1874 (Brochu, 1997; Clark & Norell, 1992).

In morphologically immature individuals of all extant crocodylian species, the suture between the ectopterygoid and pterygoid posterior to the suborbital fenestra is characterized by a “flexure,” where the pterygoid sends a process within the ectopterygoid (Brochu, 1997; see also Appendix 2 of Rio & Mannion, 2021). As noted by Brochu (1997), this “flexure” is lost at maturity in all extant crocodylians except for caimanines. In *T. rackhami* QMF16856, the “flexure” between the pterygoid and ectopterygoids is clearly absent (Figures 1b, 2b, and 13; character 126, state 0), indicating that (1) this feature is consistent with morphologically mature crocodylians, and that (2) in this regard, *T. rackhami* is unlike extant caimanines.

The basicranium (parabasisphenoid and basioccipital) of hatchling and juvenile crocodylians is dorsoventrally compressed, whereas at later stages of ontogeny it becomes dorsoventrally deeper, or more “verticalized” (i.e., the cranial metamorphosis of Tarsitano, 1985; Dufeau & Witmer, 2015; Kuzmin et al., 2021; pers. obs. of *C. porosus* specimens). In QMF16856, the basicranium is relatively deep dorsoventrally and the parabasisphenoid is exposed in occipital view (Figures 1e and 2e)—this is unlike the condition in hatchling or juvenile individuals. However, it bears notice that the basicranium of QMF16856 is nonetheless not as deep as that of fully mature extant crocodylians (excluding *Gavialis gangeticus* [Gmelin, 1789]). Willis (1993) reported that the basioccipital plate of QMF16856 has an inclination such that its occipital surface is facing posteroventrally. A posteroventrally facing basioccipital plate like that of *T. rackhami* is a plesiomorphic condition for Eusuchia (e.g., Pol et al., 2009), as the majority of known Crocodylia have a posteriorly facing basioccipital plate at maturity.

In hatchlings of extant crocodylians, the condylar surfaces of the quadrates are approximately on the same level as the occipital condyle when observed in dorsal view (Kuzmin et al., 2021). This is mainly a result of the proportionately short length of the body of the quadrate. During ontogeny, the quadrate body of crocodylians becomes progressively more elongated (Kuzmin et al., 2021). Thus, more mature individuals have their quadrate condyles project posteriorly to the occipital condyle, which is also the condition present in QMF16856 (Figures 1a and 2a). Regardless, the quadrate body of QMF16856 is relatively short. There is no doubt that this condition of QMF16856 is unlike that of hatchlings, and is similar to the condition in most mature crocodylians. However, it can be concluded that the quadrate body of *T. rackhami* did not attain proportional lengths like that of *A. clarkae*, where both adult and juvenile individuals of the latter have quadrate bodies that are longer than the width at the quadrate condyles (J. Ristevski, pers. obs. of QMF17433; Willis & Molnar, 1991; Appendix 2 of Rio & Mannion, 2021).

In extant mature crocodylians, the ventral surface of the quadrate body is covered by several well‐developed crests (discussed in detail by Iordansky, 1964, 1973). In morphologically immature crocodylians, the ventral surface of the quadrate tends to be relatively smooth and with no obvious crests, or crests that are only weakly developed (Iordansky, 1964, 1973; Kley et al., 2010; Kuzmin et al., 2021). In QMF16856, the quadrate crests are well‐developed and expressed as sharp ridges—crests A and B are especially prominent on both quadrates (Figures 22d and 23d). The degree of development of crests A and B (sensu Iordansky, 1964) on the quadrates of QMF16856 is a morphologically mature feature.

### 5.3 Cranial ossification

The cranium of QMF16856 shows signs of ossification that are absent in morphologically immature crocodylians, particularly hatchlings (e.g., Dufeau & Witmer, 2015; Iordansky, 1973; Kälin, 1933). For example, the elements of the cranial table, such as the frontal and parietal are fully fused.

### 5.4 Cranial dermal bone texture

The cranium of the *T. rackhami* holotype is heavily sculptured with prominent and deep sub‐circular to sub‐rectangular pits that are separated from each other by ridges. Such cranial ornamentation is intensive on the dorsal surfaces of the frontal, parietal, postorbitals, squamosals, the dorsal exposure of the supraoccipital, the lateral surfaces of the jugals, quadratojugals, and what is preserved of the maxillae, nasals (particularly the left nasal), lacrimal, and prefrontal is also ornamented. The ample size of the pits results in some of them crossing sutural boundaries (e.g., the frontoparietal suture). Furthermore, the cranial table of *T. rackhami* is characterized by its three prominent crests. The midsagittal crest, spread on the dorsal surfaces of the frontal and parietal, is extremely developed in *T. rackhami*. In morphologically immature individuals of extant taxa, the level of cranial ornamentation increases with age such that hatchling and juvenile crocodylians have relatively smooth bone textures and the cranial pitting is comparatively shallower than in adults (e.g., Clarac et al., 2015; de Buffrénil, 1982; de Buffrénil et al., 2015; Mook, 1921). We agree with Willis' (1993) observation that the degree of cranial sculpturing in QMF16856 is consistent with that seen in morphologically mature crocodylians.

### 5.5 Number of teeth/alveoli

The number of teeth in QMF16856 is uncertain as there could have been either 12 or 13 teeth in each maxilla. Regardless of whether the exact maxillary tooth count was 12 or 13, both are within the limits of morphologically immature and mature crocodylians. The tooth morphology of QMF16856 cannot be assessed directly since no teeth are preserved in situ.

Young crocodylians have the last few alveoli of the maxilla and dentary set in a groove (e.g., Bertin et al., 2018). Throughout ontogeny, the last alveoli usually become separated from each other with the development of interalveolar septa. The last maxillary alveoli of QMF16856 are preserved as lacking interalveolar septa, a feature especially obvious between the last two alveoli on the right maxilla. However, the left maxilla indicates that the interalveolar septum between the last two alveoli is well‐developed, and the apparent lack of closure of the septa may be a result of taphonomic distortion. Due to the relatively poor‐preservation and asymmetry between the alveoli on the left and right side, it is unclear if the alveoli were indeed interconnected as inferred by Willis (1993). Nevertheless, it seems more likely that the alveoli were separated by septa that have been obliterated as a result of post‐mortem decay. Unfortunately, precise interpretation of this condition in QMF16856 is not possible.

### 5.6 Festooning of the maxillae

A feature related to the tooth row is the festooning of the maxillae. In QMF16856, the maxillae have deep undulations (Figures 1c,d, 2c,d, and 4c,d). In most nonslender snouted crocodylians, the festooning of the maxillae tends to become deeper with age (Mook, 1921; Kälin, 1933; pers. obs. of numerous *C. porosus* specimens). The relative depth of the snout and pronounced festooning in QMF16856 is reminiscent of morphologically mature crocodylians. However, it may be expected that a short‐snouted crocodylian like *T. rackhami* would have deeper maxillary festooning than a crocodylian with a proportionally longer snout.

## 6 REMARKS ON OTHER SPECIMENS REFERRED TO *TRILOPHOSUCHUS*

### 6.1 *Trilophosuchus rackhami*

In addition to the holotype, Willis (1993) assigned three other specimens to *T. rackhami* that were also recovered from the type locality, the Ringtail Site: QMF16857 an isolated frontal (Figure S2.1B–S2.1E), QMF16858 an isolated right postorbital (Figure S2.1G and S2.1H), and QMF16859 an isolated basioccipital (Figure S2.2).

The frontal (QMF16857) is completely preserved and displays a morphology that is consistent with that of the holotype. It has a short anterior process, a well‐developed mid‐sagittal crest, and the orbital margins are also acute and crest‐like. Thus, we concur with Willis (1993) and consider QMF16857 to be referrable to *T. rackhami*.

The right postorbital (QMF16858) is mostly complete (its descending process is only partially preserved) and displays a morphology that is consistent with that of the holotype. Evident at the medial margin of the anteromedial process is a ridge that conforms with the parasagittal crest that extends over the postorbital. Additionally, the portion of the postorbital that bounded the anterolateral margin of the supratemporal fenestra is relatively straight, as it is in the *T. rackhami* holotype. Lastly, the posterior process of the postorbital has a diagonal posterior edge which agrees with the condition present at the postorbital‐squamosal suture of the holotype. Therefore, we concur with Willis (1993) and regard QMF16858 to also be referrable to *T. rackhami*.

### 6.2 QMF16859, a fragmentary basioccipital

Specimen QMF16859 (formerly AR 17037; Figure S2.2) is a poorly preserved basioccipital that is broken into five pieces—four small and nondescript fragments, and the fifth piece consists of the occipital condyle with a partial basioccipital plate. Although the overall size of QMF16859 is nearly the same as the basioccipital of the *T. rackhami* holotype, there are several morphological inconsistencies between them. For one, the occipital condyle neck on the holotype is quite elongated, whereas in QMF16859 it is proportionally shorter. Furthermore, the basioccipital crest in the holotype is prominent whereas the crest in QMF16859 is comparatively less developed. Another notable difference is present in the proportions of the basioccipital plate. In the holotype, the basioccipital plate has a width/height ratio of approximately 2.3, whereas the same ratio in QMF16859 is approximately 1.5 (ratio estimated from figure 8.5 in Willis, 1995b, where the nonfragmented QMF16859 is figured). Based on these differences, we reject the assignment of QMF16859 to *T. rackhami*.

Specimen QMF16859 has morphological resemblances with another crocodylian known from the Ringtail Site, *Mekosuchus sanderi*. The basioccipital of *M. sanderi* (QMF31166; see Figure S1.15) is similar to QMF16859 in overall size, but also in the possession of a relatively short occipital condyle neck, a less developed basioccipital crest, and a basioccipital plate with a nearly identical width/height ratio (approximately 1.53 in QMF31166). Therefore, it is possible that QMF16859 belongs to *M. sanderi* rather than *T. rackhami*. However, since QMF16859 is fragmentary, we refrain from assigning it to any particular genus or species. To err on the side of caution, we propose a provisional referral for QMF16859 as Mekosuchinae gen. et sp. indet., although note that it may prove to be referrable to *M. sanderi*.

### 6.3 *Trilophosuchus* sp.

A previously undescribed isolated parietal (QMF60374; Figure S2.3) from the Hiatus Site at the Riversleigh WHA is demonstrably referrable to the genus *Trilophosuchus*. Recognizing the morphology of QMF60374 as consistent with *Trilophosuchus* by J. D. Scanlon in 2005 led to the first report on the presence of the genus from the Hiatus Local Fauna (Scanlon, 2006a, 2014; also, Archer et al., 2006). Specimen QMF60374 is an almost complete and well‐preserved parietal that has a strong morphological resemblance to *Trilophosuchus*. Like the parietal of the *T. rackhami* holotype, QMF60374 has a dorsal lamina that bears a conspicuous midsagittal crest, and along the dorsolateral (=supratemporal) margins of the parietal are a pair of parasagittal crests. Anteriorly, the sutural margin that contacted the frontal is linear, whereas posteriorly are a pair of posterior processes that would have flanked the dorsal exposure of the supraoccipital. As can be seen when QMF60374 is observed in dorsal view, the posteromedial margin of the element gives away the sutural outline for contact with a prominent dorsal exposure of the supraoccipital which is similar to the contour in the *T. rackhami* holotype. Moreover, the descending processes of the parietal in QMF60374 are pierced by foramina. Finally, evident in ventral view of QMF60374 are a pair of large foramina that lead into a spacious pneumatic recess within the parietal. Overall, the morphology of QMF60374 is remarkably consistent with that of the parietal from the *T. rackhami* holotype. The parietal of *T. rackhami* is highly distinctive and no other known mekosuchine has a parietal with such unique morphology. Based on these observations, we are confident in assigning QMF60374 to the genus *Trilophosuchus*.

However, there are few minor morphological differences between the parietal of the *T. rackhami* holotype and QMF60374. For instance, the lateral margins (corresponding to the medial margins of the supratemporal fenestrae) of QMF60374 are gently concave, whereas in the *T. rackhami* holotype they are straight and without any, or negligible at most, concave outlines. Also, the right posterior process of QMF60374 (the left posterior process is incomplete) is proportionally shorter and with a more tapered tip when compared to that of the *T. rackhami* holotype. Admittedly, these differences are minor and could be attributed to either intraspecific or ontogenetic variation (QMF60374 is smaller than the parietal of QMF16856). On morphological grounds alone, no strong arguments can be made in favor of erecting a new species based on QMF60374.

The Hiatus Site is considered to be late Oligocene in age (Scanlon, 2006a, 2006b; Woodhead et al., 2016), which is approximately 10 million years older than the Ringtail Site from where the type material of *T. rackhami* derives. Thus, considering the significant age difference between all material referred to *T. rackhami* and QMF60374, as well as the subtle morphological differences it is certainly possible that the Hiatus Site *Trilophosuchus* may represent a new species within this genus. However, naming a new species of *Trilophosuchus* must be founded on solid morphological basis which would be categorized by unambiguous autapomorphic features. Since unambiguous autapomorphic features are clearly lacking in QMF60374, we refer to this specimen as *Trilophosuchus* sp. More complete material is needed in order to elucidate the specific status of the *Trilophosuchus* material from the Hiatus Site.

## 7 RESULTS FROM THE PHYLOGENETIC ANALYSES

**FIGURE 24:**
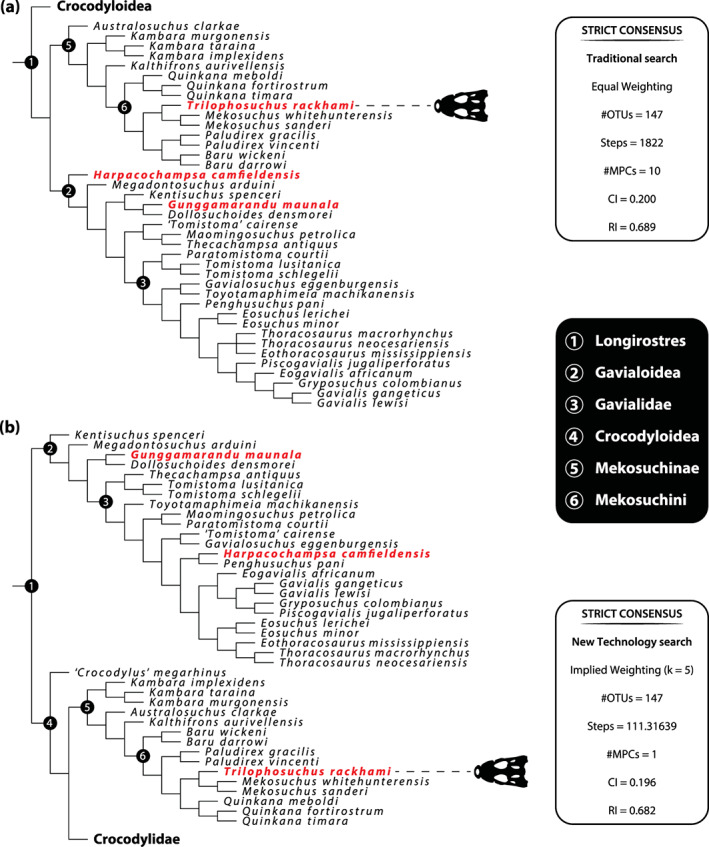
Select results from the phylogenetic analyses with the focus on Longirostres, particularly the variable position of Mekosuchinae and the Australian gavialoids. (a) Strict consensus of 10 MPCs from the analysis run under TrS, without IW. (b) Strict consensus of 1 MPC from the analysis run under NTS and used IW *k* = 5. For the complete strict consensus topologies, nodal values, and more information on all phylogenetic analyses performed in this study, see Document S3. CI, consistency index; #MPCs, number of most parsimonious cladograms; #OTUs, number of operational taxonomic units; RI, retention index

**FIGURE 25:**
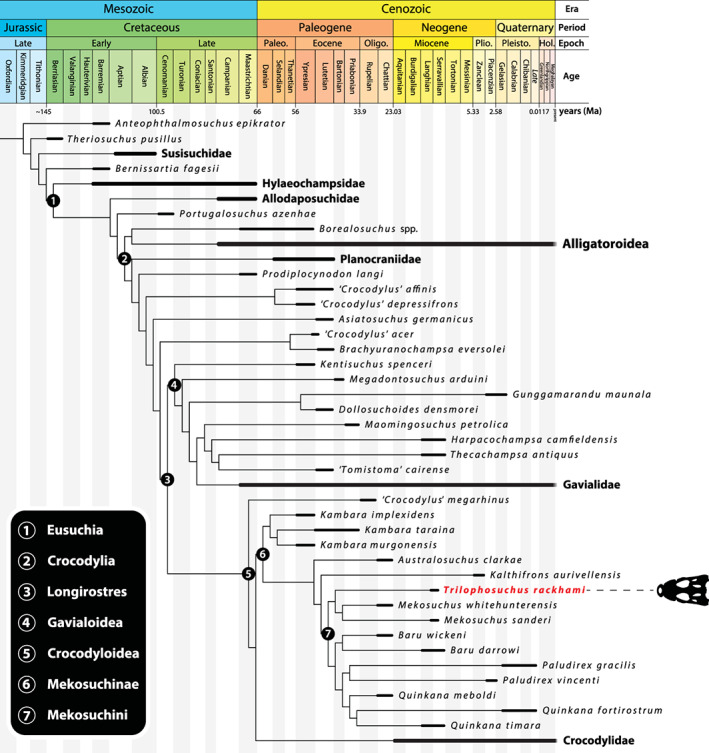
Time‐calibrated phylogeny based on the strict consensus results from a single fully‐resolved most parsimonious cladogram. The analysis that produced this topology was run under the New Technology search procedures and used the Implied Weighting method, with the *k* (concavity constant) value set to 25.0 (*k* = 25). The first and last appearance dates for the taxa were acquired from the Paleobiology Database (https://paleobiodb.org) on the January 7, 2022. The geologic dates are based on version 2021/10 of the International Chronostratigraphic Chart (https://stratigraphy.org/chart). For the complete strict consensus topology, nodal values, and more information on the phylogenetic analyses, see Document S3. Hol., Holocene; Oligo., Oligocene; Paleo., Paleocene; Pleisto., Pleistocene; Plio., Pliocene

The phylogenetic analyses resulted in generally consistent and largely resolved topologies (Figures 24 and 25; for the complete results see Document S3). The resulting cladograms have either entirely resolved (in three of the eight analyses) or almost completely resolved (in four of the eight analyses) relationships, although the nodal support is relatively weak. Only one analysis (run under the NTS option and used the EW method) produced a more poorly resolved cladogram. All eight analyses consistently recovered a monophyletic Longirostres (Crocodyloidea + Gavialoidea), with Mekosuchinae as part of this larger clade. The nodal support for Longirostres ranges from very weak in some analyses (Bremer = −3 in one analysis; bootstrap = <50% in all analyses) to being one of the best supported clades (Bremer = 33 in two analyses). Two nonmekosuchine Australian taxa belong to Gavialoidea—*Gunggamarandu maunala* Ristevski et al., 2021 and *Harpacochampsa camfieldensis* Megirian et al., 1991. As in Ristevski et al. (2021), *G. maunala* was again recovered in a relatively basal position within Gavialoidea, usually as the sister taxon to *Dollosuchoides densmorei* Brochu, 2007. However, unlike the results of Ristevski et al. (2020a, 2021), *H. camfieldensis* is also found within Gavialoidea. Although repeatedly placed as a gavialoid, the position of *H. camfieldensis* within Gavialoidea is variable, ranging from a position as the basal‐most gavialoid (Figure 24a), to a more derived nongavialid gavialoid (e.g., Figure 25), to a gavialid gavialoid (e.g., Figure 24b). None of the analyses managed to recover a sister taxon relationship between *G. maunala* and *H. camfieldensis*.

The clade Mekosuchinae was found to be monophyletic and consists of *Kambara* Willis et al., 1993, *Australosuchus clarkae*, *Kalthifrons aurivellensis* Yates & Pledge, 2016 and Mekosuchini. In turn, Mekosuchini incorporates all other mekosuchines included in the matrix, which are the species of *Baru*, *Mekosuchus*, *Paludirex*, and *Quinkana*, as well as *T. rackhami*. A monophyletic Mekosuchini was recovered in all analyses. *Trilophosuchus rackhami* was persistently found forming a clade with *Mekosuchus*, but the position of the *Trilophosuchus* + *Mekosuchus* clade within Mekosuchini is unstable. In four of the eight analyses, the *Trilophosuchus* + *Mekosuchus* clade is in a basal position within Mekosuchini (Figure 25), whereas two analyses found (*Trilophosuchus* + *Mekosuchus*) in a more derived position. One such analysis recovered (*Trilophosuchus* + *Mekosuchus*) as a sister clade to (*Baru* + *Paludirex*) (Figure 24a), while another found (*Trilophosuchus* + *Mekosuchus*) to be a sister clade of *Quinkana* (Figure 24b). A potential solution towards recovering a stable position for the *Trilophosuchus* + *Mekosuchus* clade likely depends on increasing the number of scored characters for these taxa as well as other mekosuchines. This, of course, is contingent on the discovery of more complete fossil specimens than what is currently known. The nodal support for the *Trilophosuchus* + *Mekosuchus* clade is rather poor in all analyses (Bremer = 1 in five analyses, and Bremer = 2 in three analyses; bootstrap = <50% in all analyses). The results from the phylogenetic analyses identified a maximum of 11 autapomorphies for *T. rackhami*. Seven of these character states were found to be autapomorphies in all eight analyses, however, four of them were inconsistently recognized as such. These are summarized in Table S3.2 in Document S3.

## 8 DISCUSSION

### 8.1 Was *Trilophosuchus rackhami* a small‐sized crocodylian at maturity?

The combination of both morphologically mature and immature characteristics led Willis (1993) to propose that *T. rackhami* could be a small‐bodied crocodylian with paedomorphic features (see also Willis, 1997b and 2006). Most cranial features of QMF16856 are demonstrably morphologically mature, such as: the degree of cranial sutural fusion and ossification, the intensity of the cranial ornamentation and development of cranial crests, the morphology of the supratemporal fenestrae, the loss of sutural “flexure” between the pterygoid and ectopterygoids, the strong development of crests A and B on the ventral surface of the quadrate body, and the relatively deep festooning of the maxillary tooth row. Nonetheless, QMF16856 also displays a few morphological features that would normally be associated with immature individuals of most extant taxa, most notable of which are the (possible) poor ossification at the anterior of the braincase, and a foramen magnum with a greater width than the occipital condyle. The retention of these immature traits into maturity is most parsimoniously considered an example of paedomorphosis, as was original postulated by Willis (1993, 1997b, and 2006). Intriguingly, the cranial table of QMF16856 slopes lateroventrally—this feature is usually lost in post‐hatchling ontogeny among crocodylians, except for some gavialoids. Therefore, we also view the presence of a lateroventrally sloping cranial table to be a paedomorphic trait of *T. rackhami*, and one that is exclusive to this taxon among currently known mekosuchines. Furthermore, the posteroventrally facing occipital surface of the basioccipital plate is a plesiomorphic feature in *T. rackhami*. The *T. rackhami* holotype individual is certainly not a hatchling, and since its cranial morphology is characterized by a greater number of morphologically mature than immature features it is unlikely to be a juvenile either.

Today, the caimanine *Paleosuchus palpebrosus* is regarded as one of the smallest extant crocodylians, with a TL of 1.5–1.6 m for males and 1.2 m in TL for females (Grigg & Kirshner, 2015; Magnusson, 1992; Ross & Magnusson, 1989). Exceptionally large males of this species are known to attain a TL of 1.8 m and in rare cases even 2 m (Campos et al., 2010; Magnusson & Campos, 2010; Ouboter, 1996). Due to its relatively small size for a crocodylian, *Pa. palpebrosus* is often referred as a “dwarf” crocodylian, with some of its common (i.e., nonbinomial) names being Cuvier's dwarf caiman or simply dwarf caiman. Other than *Pa. palpebrosus*, the extant *Osteolaemus tetraspis* (common name dwarf crocodile), and *Alligator sinensis* Fauvel, 1879 are also small‐bodied crocodylians, having TLs that rarely exceed 2 m (Eaton, 2010; Grigg & Kirshner, 2015; Shirley et al., 2017; Thorbjarnarson & Wang, 2010).

Small body‐size (a TL of ~2 m or less at maturity) among mature members of Mekosuchinae is not without precedent. A small body‐size at maturity has been hypothesized for several mekosuchines, most notably *T. rackhami* and *Mekosuchus* spp. (Balouet, 1989, 1991; Holt et al., 2007; Mead et al., 2002; Scanlon, 2006a, 2014; Stein et al., 2013, 2015; Willis, 1993, 1995a, 1995b, 1997b, 2001, 2006; Yates, 2017; Yates & Pledge, 2016). Previously, some authors have proposed a length estimate of less than 1 m for *T. rackhami* (Willis, 1995a, 1997b, 2006; Wroe, 2002). Based on such interpretations, *T. rackhami* has also been referred as a dwarf species (Stein et al., 2015, 2016; Willis, 1993; Yates & Pledge, 2016). Our TL estimates for the *T. rackhami* holotype individual (between 70 and 90 cm; see Section 2.3) are consistent with the previous estimates of less than 1 m. Therefore, like Willis (1993) we also interpret QMF16856 as a mature individual of a small‐bodied species.

### 8.2 Implications from the phylogenetic analyses

Since the earliest studies on mekosuchine systematics (Salisbury & Willis, 1996; Willis, 1997b) *Trilophosuchus* has been one of the key taxa in establishing the clade and its subclade Mekosuchini. The phylogenetic relationships of *T. rackhami* have been tested in many cladistic analyses, and all recovered it within Mekosuchinae (e.g., Azzarà et al., 2021; Brochu, 2001, 2003, 2007, 2012; Cossette et al., 2020; Lee & Yates, 2018; Molnar et al., 2002; Rio & Mannion, 2021; Salisbury & Willis, 1996; Scheyer et al., 2013; Stein et al., 2016; Willis, 1993, 1995b; Yates & Pledge, 2016). Our results also confirm the status of *Trilophosuchus* as a mekosuchine and part of the subclade Mekosuchini. However, the results from our phylogenetic analyses have greater implications for Mekosuchinae as a whole since none of them recovered Mekosuchinae as a subclade of Crocodylidae. In fact, all analyses found Mekosuchinae to be a basal clade of Crocodyloidea (e.g., Figures 24b and 25), except for one analysis (Figure 24a) which failed to recover Mekosuchinae as part of Crocodyloidea but instead as a sister clade to the rest of Gavialoidea. The results from this study, as well as results from several previous publications (Azzarà et al., 2021; Cossette et al., 2020; Lee & Yates, 2018; Rio & Mannion, 2021) imply that the rank of Mekosuchinae as a subfamily of Crocodylidae should be renounced. It can be argued that the taxonomic status of Mekosuchinae should formally be reinstated to family. Our cladistic results are further supported by the rich taxonomic make up of Mekosuchinae (current status) as well as the distinctive morphologies displayed among members of the clade. Indeed, as described in this study, *T. rackhami* is one of the most morphologically distinct mekosuchines and, arguably, crocodylians.

## AUTHOR CONTRIBUTIONS

**Jorgo Ristevski:** Conceptualization (lead); formal analysis (lead); investigation (lead); methodology (lead); visualization (lead); writing – original draft (lead); writing – review and editing (lead). **Vera Weisbecker:** Software (supporting); supervision (supporting); writing – review and editing (supporting). **John D. Scanlon:** Investigation (supporting); resources (supporting); writing – review and editing (supporting). **Gilbert J. Price:** Supervision (supporting); writing – review and editing (supporting). **Steven W. Salisbury:** Conceptualization (supporting); investigation (supporting); methodology (supporting); supervision (lead); writing – review and editing (supporting).

## Supporting information


**Appendix S1** Document S1

**Appendix S2** Document S2

**Appendix S3** Document S3

## Data Availability

Additional supplementary data is available at https://datadryad.org/stash/dataset/doi:10.5061/dryad.gmsbcc2qm and https://zenodo.org/record/6968350.
