# Supplementary material for: Cranial anatomy of the mekosuchine crocodylian Trilophosuchus rackhami Willis, 1993
Source: Anat Rec (Hoboken). 2022 Aug 29;306(2):239–97. doi: 10.1002/ar.25050 (PMC10086963; doi:10.1002/ar.25050)
Supplement: Supplementary file 1 — Appendix S1 Document S1 [file AR-306-239-s001.pdf]

SUPPLEMENTARY MATERIAL FOR

CRANIAL ANATOMY OF THE MEKOSUCHINE CROCODYLIAN *TRILOPHOSUCHUS RACKHAMI* WILLIS, 1993

SUPPLEMENTAL DOCUMENT S1: ADDITIONAL FIGURES AND MORPHOLOGICAL COMPARISONS

by JORGO RISTEVSKI[1*], VERA WEISBECKER[1,2], JOHN D. SCANLON[3], GILBERT J. PRICE[4], and STEVEN W. SALISBURY[1]

[1]School of Biological Sciences, The University of Queensland, Brisbane, 4072, Queensland, Australia

[2]College of Science and Engineering, Flinders University, Bedford Park 5042, South Australia, Australia

[3]Phoenix Environmental Sciences, Osborne Park 6017, Western Australia, Australia

[4]School of Earth and Environmental Sciences, The University of Queensland, Brisbane, 4072, Queensland, Australia

***Corresponding author:**

Jorgo Ristevski[1]

School of Biological Sciences, Goddard Building (Building 8), The University of Queensland, Brisbane 4072, Queensland, Australia

Email address: j.ristevski@uq.net.au

CONTENTS OF THE SUPPLEMENTARY DOCUMENT

This supplementary document to the study titled "Cranial anatomy of the mekosuchine crocodylian *Trilophosuchus rackhami* Willis, 1993" contains additional figures related to the description of the holotype specimen (QMF16856) of *Trilophosuchus rackhami* Willis, 1993. The supplementary figures of QMF16856 in this document are **Figs. S1.2–S1.5**, **S1.7**, **S1.11**, **S1.12**, and **S1.16**. In **Figs. S1.6**, **S1.8–S1.10** and **S1.13–S1.15** are images of other crocodylians that are relevant to the comparative anatomy with *T. rackhami*. In **Figs. S1.17–S1.35** are given the non-annotated versions of **Figs. 4**, **5** and **7–23** from the main text. Lastly, this document includes detailed morphological comparisons between *T. rackhami* and other Australian crocodylians.

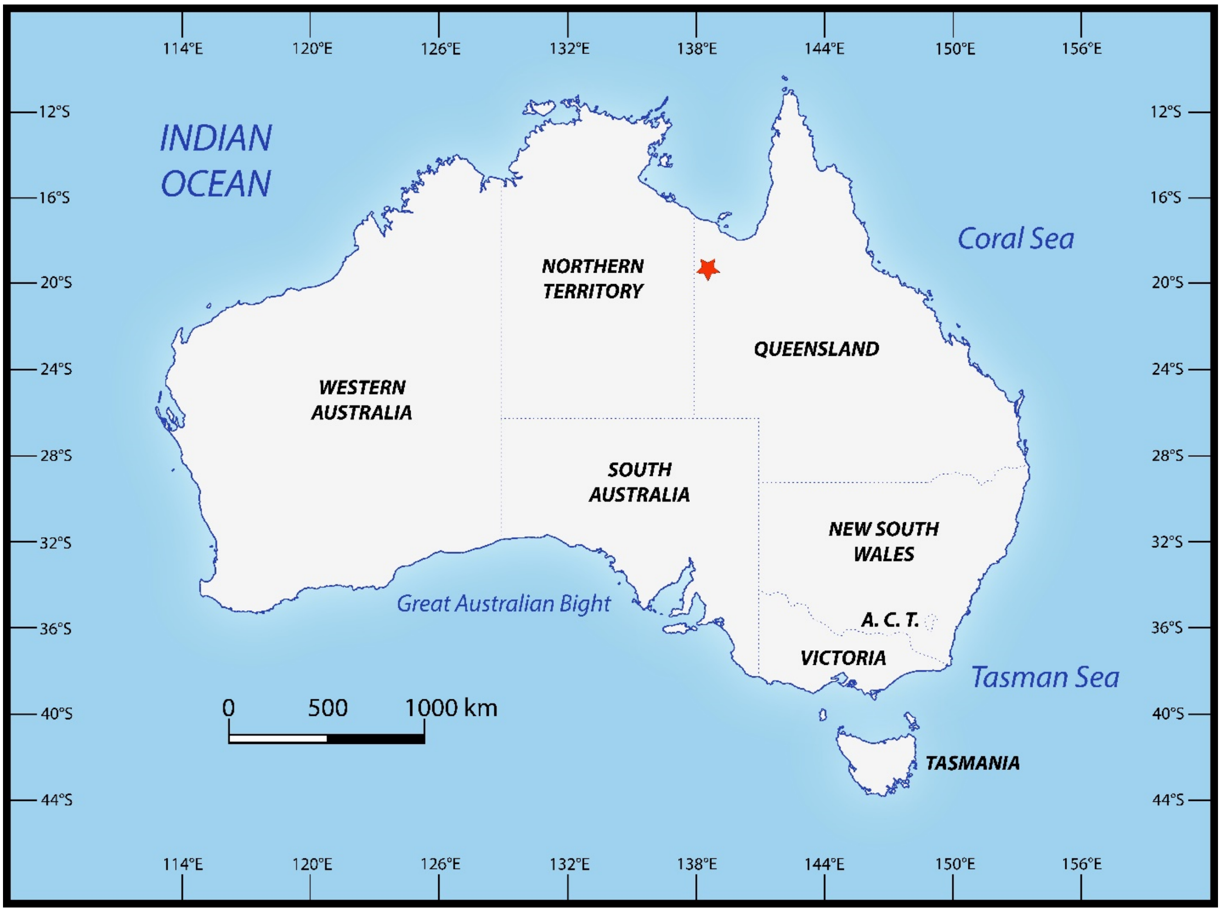

Figure S1.1 Map of Australia. The red star indicates the Riversleigh World Heritage Area, where all known specimens of *Trilophosuchus* Willis, 1993 have been discovered. For a high-resolution version of this figure, see the PDF file of **Figure S1.1** provided as a supplementary file.

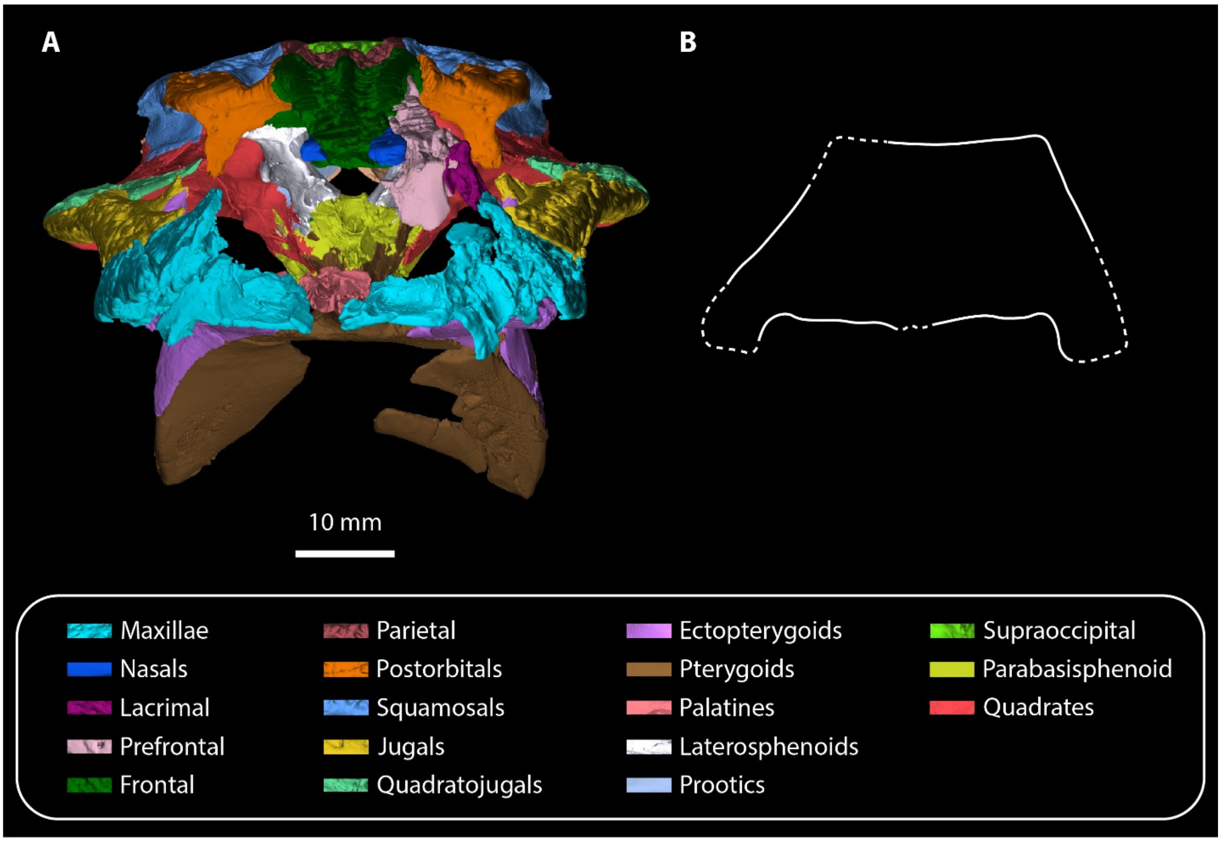

Figure S1.2 ***Trilophosuchus rackhami*** **Willis, 1993, QMF16856, holotype.** (A) Digital model of the cranium in anterior view. (B) Drawing of the cross-sectional outline of the snout (as preserved), showing its trapezoidal cross-section. The dashed lines in (B) are hypothetical outlines of the missing portions. For a high-resolution version of this figure, see the PDF file of **Figure S1.2** provided as a supplementary file.

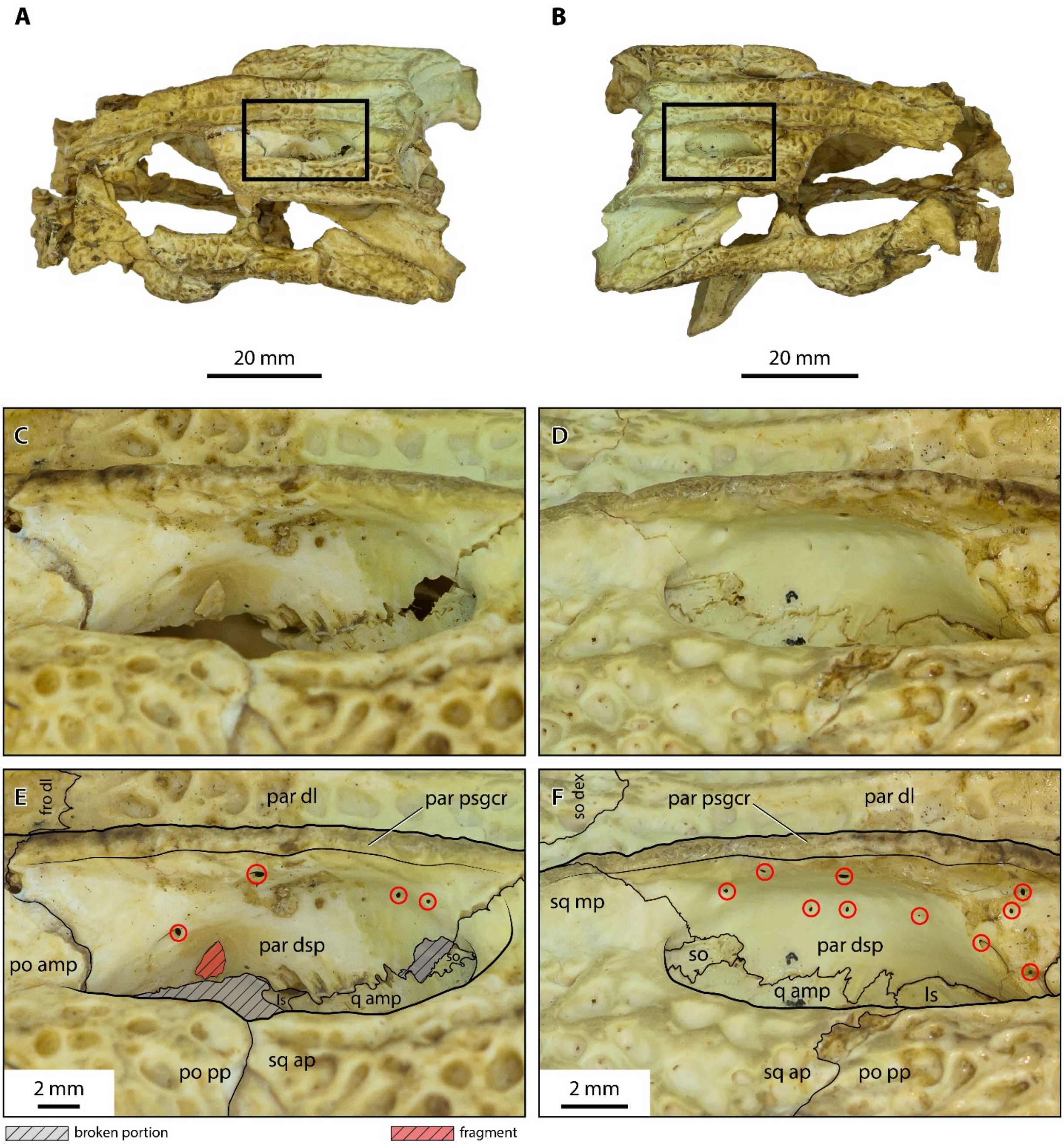

Figure S1.3 ***Trilophosuchus rackhami*** **Willis, 1993, QMF16856, holotype, close ups on the descending processes of the parietal.** (A) Cranium in left dorsolateral view, with the black box indicating the portions shown in (C) and (E). (B) Cranium in right dorsolateral view, with the black box indicating the portions shown in (D) and (F). Close up of the left parietal descending process (C) non-annotated photograph, and (E) annotated photograph. Close up of the right parietal descending process (D) non-annotated photograph, and (F) annotated photograph. The red circles in (E) and (F) indicate the foramina that perforate the parietal descending process.

Figure S1.3 (continued) The same (and additional) foramina can also be seen on the digital model (see Figs. 8C, S1.20C and the interactive 3D PDF file). For a high-resolution version of this figure, see the PDF file of **Figure S1.3** provided as a supplementary file. Abbreviations: **fro dl**, dorsal lamina of the frontal; **ls**, laterosphenoid; **par dl**, dorsal lamina of the parietal; **par dsp**, descending process of the parietal; **par psgcr**, parasagittal crest on parietal; **po amp**, anteromedial process of the postorbital; **po pp**, posterior process of the postorbital; **q amp**, anteromedial process of the quadrate; **so**, supraoccipital; **so dex**, dorsal exposure of the supraoccipital; **sq ap**, anterior process of the squamosal; **sq mp**, medial process of the squamosal.

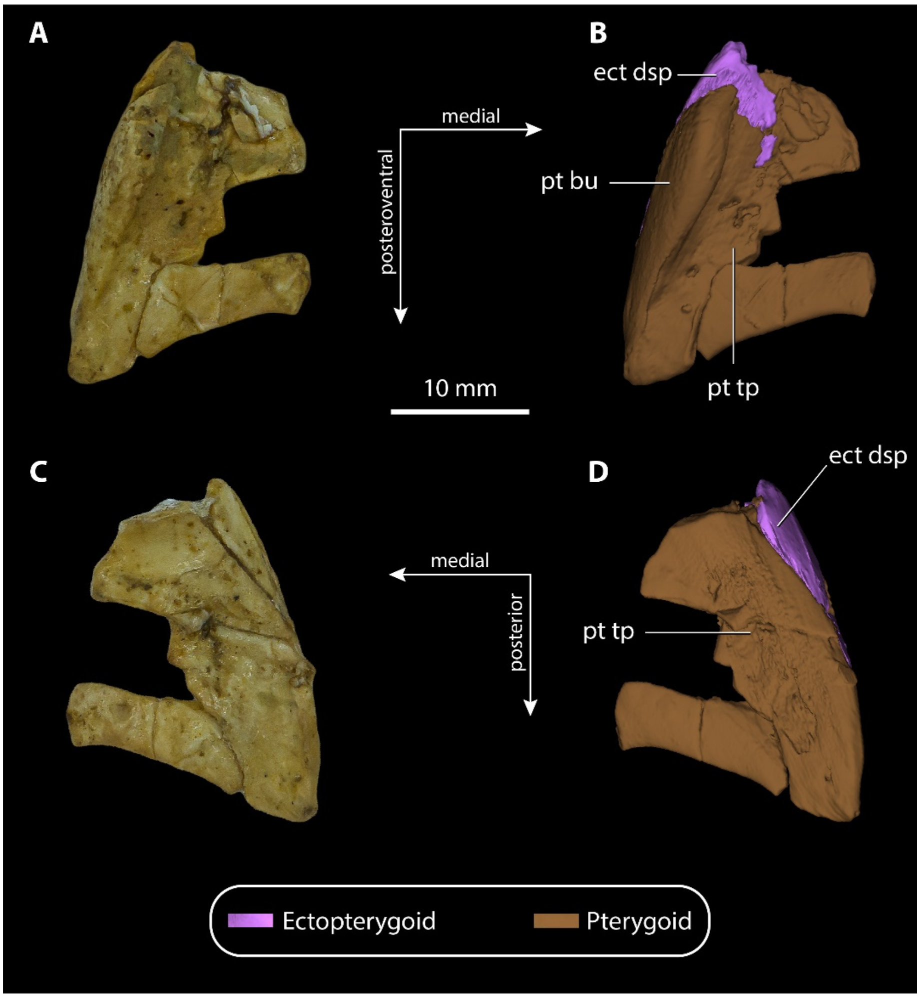

Figure S1.4 ***Trilophosuchus rackhami*** **Willis, 1993, QMF16856, holotype, broken off fragment consisting of parts of the left pterygoid and ectopterygoid.** (A) Photograph, and (B) annotated digital model of the fragment in dorsal view. (C) Photograph, and (D) annotated digital model of the fragment in ventral view. For a high-resolution version of this figure, see the PDF file of **Figure S1.4** provided as a supplementary file. Abbreviations: **ect dsp**, descending process of the ectopterygoid; **pt bu**, pterygoid buttress; **pt tp**, transverse process of the pterygoid.

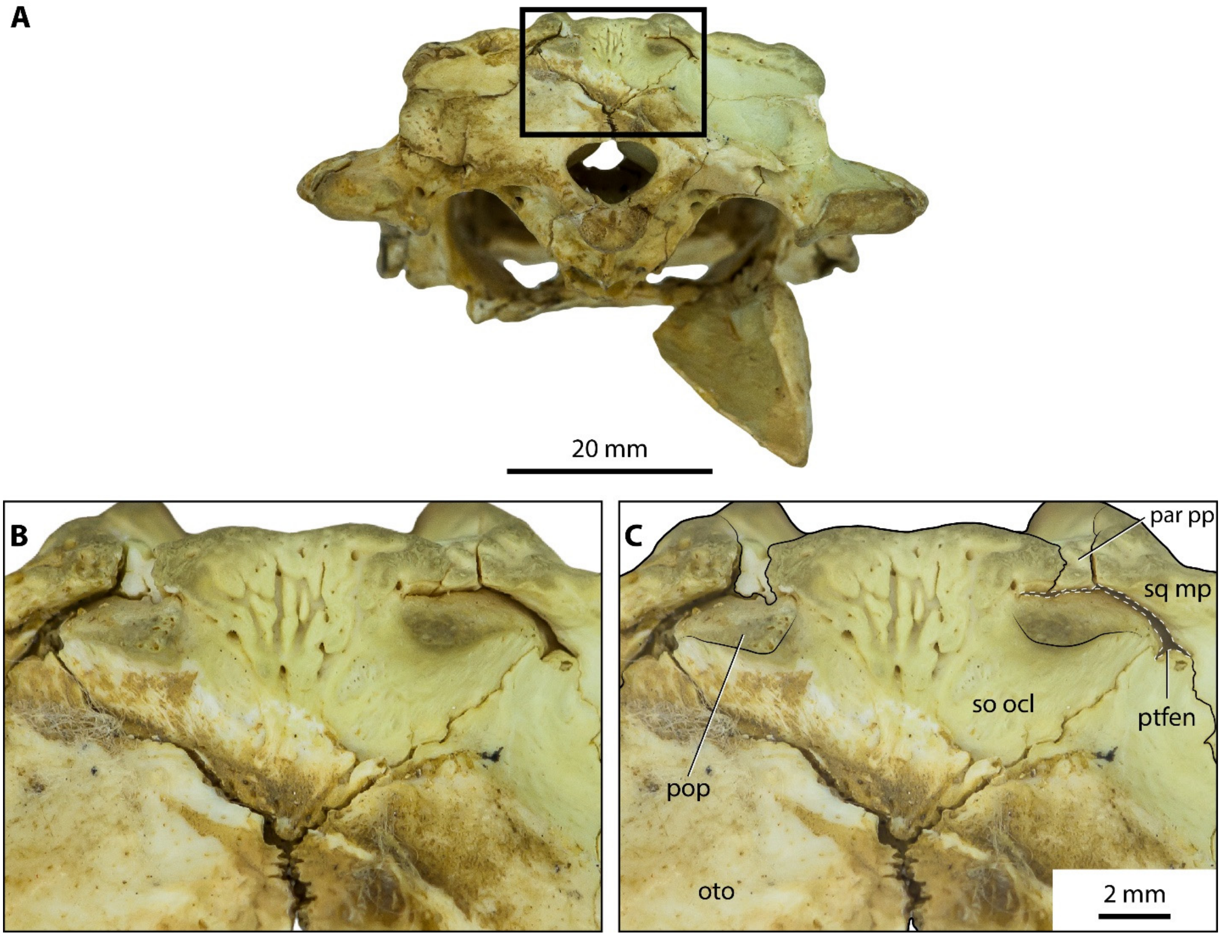

Figure S1.5 ***Trilophosuchus rackhami*** **Willis, 1993, QMF16856, holotype, close up of the supraoccipital in posterior view.** (A) Cranium in posterior view, with the black box indicating the portions shown in (B) and (C). (B) Non-annotated photograph, and (C) annotated photograph of the supraoccipital and its adjacent elements. For a high-resolution version of this figure, see the PDF file of **Figure S1.5** provided as a supplementary file. Abbreviations: **oto**, otoccipital; **par pp**, posterior process of the parietal; **pop**, postoccipital process of the supraoccipital; **ptfen**, posttemporal fenestra; **so ocl**, occipital lamina of the supraoccipital; **sq mp**, medial process of the squamosal.

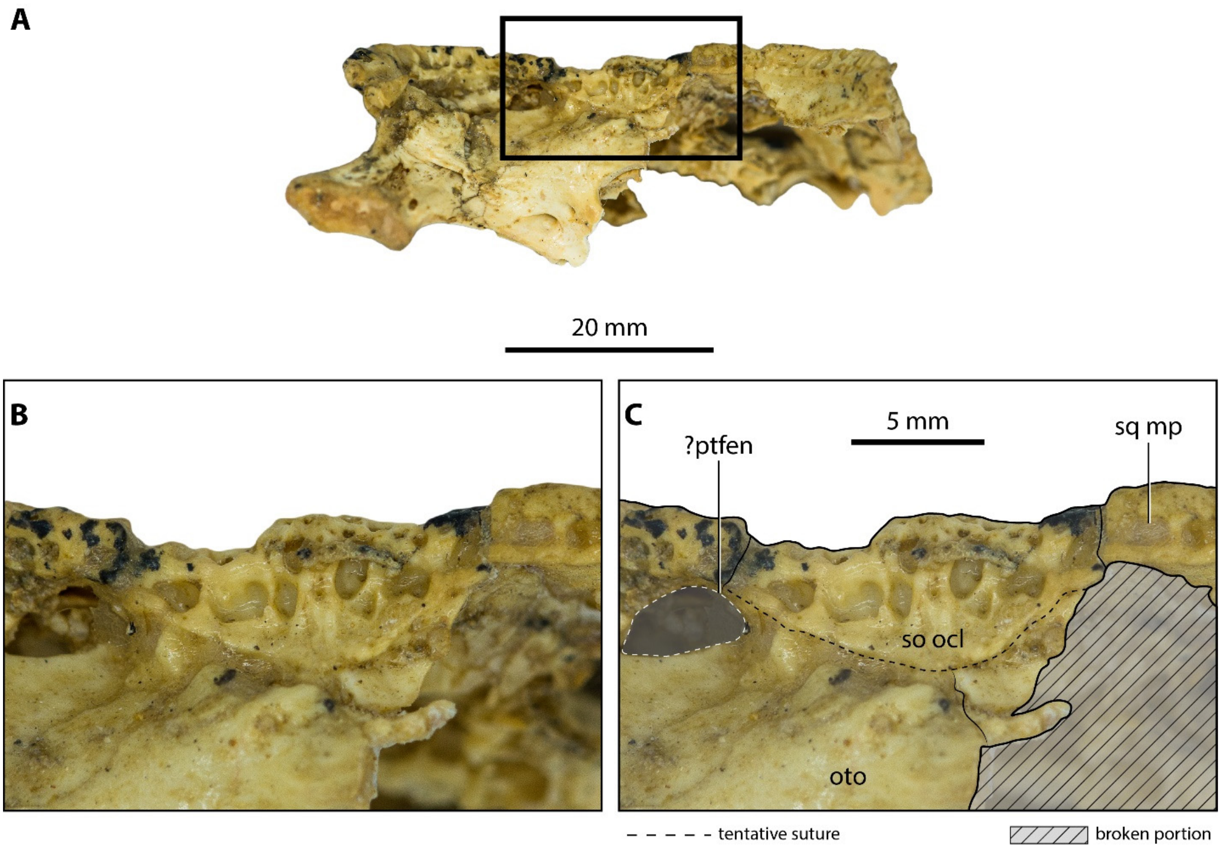

Figure S1.6 *Mekosuchus sanderi* Willis, 2001, QMF31166, close up of the supraoccipital in posterior view. (A) Cranium in posterior view, with the black box indicating the portions shown in (B) and (C). (B) Non-annotated photograph, and (C) annotated photograph of the supraoccipital and its adjacent elements. For a high-resolution version of this figure, see the PDF file of **Figure S1.6** provided as a supplementary file. Abbreviations: **oto**, otoccipital; **?ptfen**, posttemporal fenestra (tentative); **so ocl**, occipital lamina of the supraoccipital; **sq mp**, medial process of the squamosal.

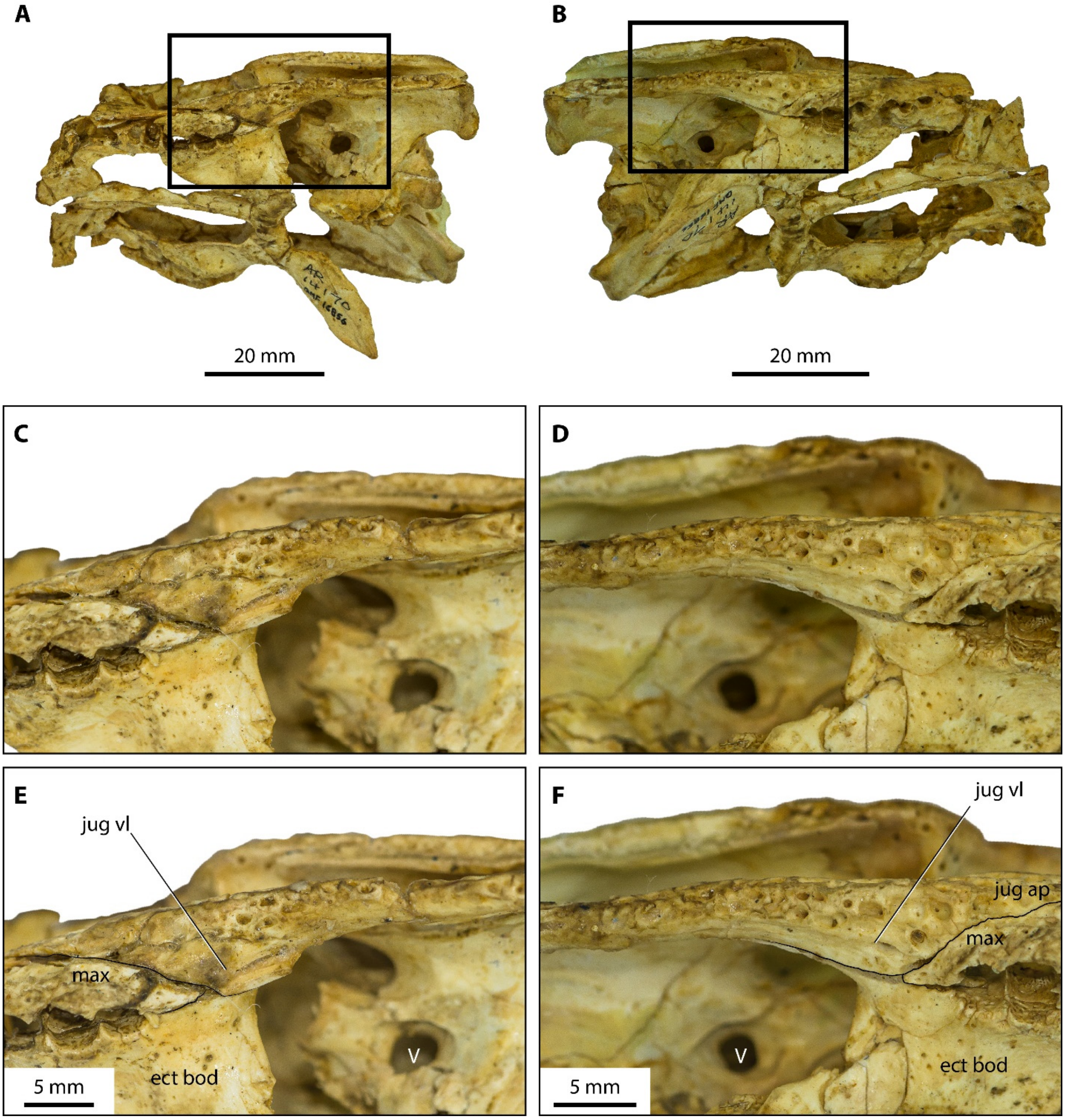

Figure S1.7 *Trilophosuchus rackhami* Willis, 1993, QMF16856, holotype, close ups on the ventral laminae of the jugals. (A) Cranium in left ventrolateral view, with the black box indicating the portions shown in (C) and (E). (B) Cranium in right ventrolateral view, with the black box indicating the portions shown in (D) and (F). Close up of the left jugal in (C) non-annotated photograph, and (E) annotated photograph. Close up of the right jugal in (D) non-annotated photograph, and (F) annotated photograph. For a high-resolution version of this figure, see the PDF file of **Figure S1.7** provided as a supplementary file. Abbreviations: **ect bod**, body of the ectopterygoid; **jug ap**, anterior process of the jugal; **jug vl**, ventral lamina of the jugal; **max**, maxilla; **V**, trigeminal foramen.

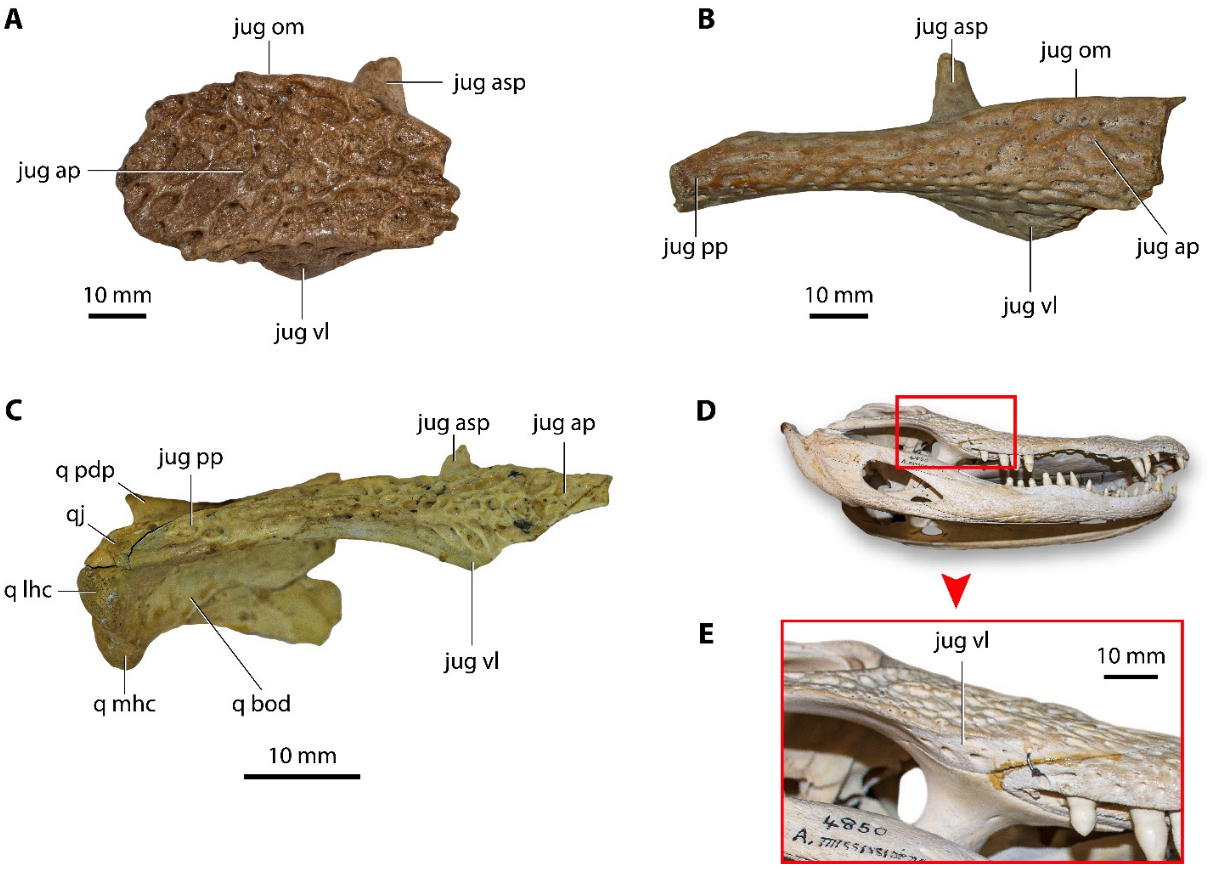

Figure S1.8 Jugals of select crocodylian taxa, showing the sharply bent and ornamented ventral laminae of the anterior process of the jugal. (A) CMC2, incomplete left jugal in lateroventral view (provisionally referred as *Quinkana*? sp. indet.). (B) *Australosuchus clarkae* Willis & Molnar, 1991, QMF18102, right jugal in lateroventral view. (C) *Mekosuchus sanderi* Willis, 2001, QMF31166, right jugal, quadratojugal and quadrate, with the focus on the jugal pictured in lateroventral view. *Alligator mississippiensis* (Daudin, 1802), QMJ4850, (D) skull and mandibles in left lateroventral view with the red box highlighting the close up of the left jugal in (E). For a high-resolution version of this figure, see the PDF file of **Figure S1.8** provided as a supplementary file. Abbreviations: **jug ap**, anterior process of the jugal; **jug asp**, ascending process of the jugal; **jug om**, orbital margin of the jugal; **jug pp**, posterior process of the jugal; **jug vl**, ventral lamina of the jugal; **q bod**, body of the quadrate; **q lhc**, lateral hemicondyle of the quadrate; **q mhc**, medial hemicondyle of the quadrate; **q pdp**, posterodorsal process of the quadrate; **qj**, quadratojugal.

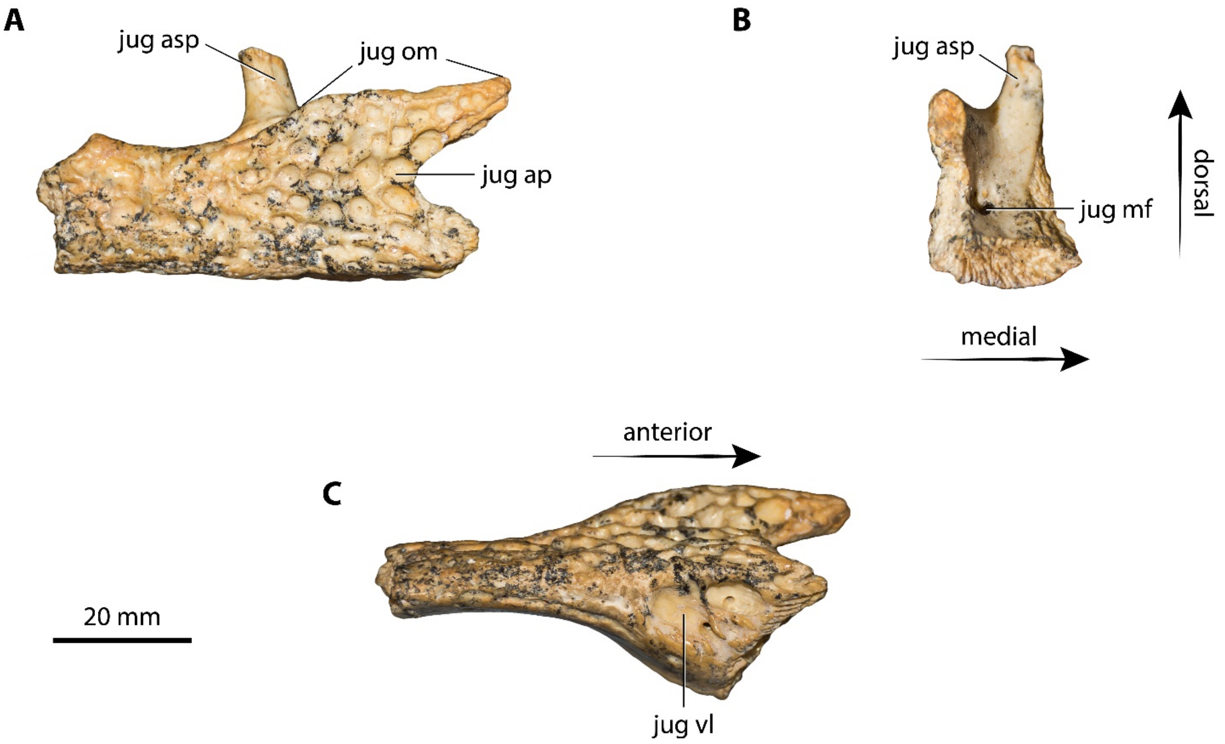

Figure S1.9 *Quinkana timara* Megirian, 1994, NTM P8697-2, incomplete right jugal. NTM P8697-2 in (A) lateral, (B) anterior, and (C) ventral views. For a high-resolution version of this figure, see the PDF file of **Figure S1.9** provided as a supplementary file. Abbreviations: **jug ap**, anterior process of the jugal; **jug asp**, ascending process of the jugal; **jug mf**, medial jugal foramen; **jug om**, orbital margin of the jugal; **jug vl**, ventral lamina of the jugal.

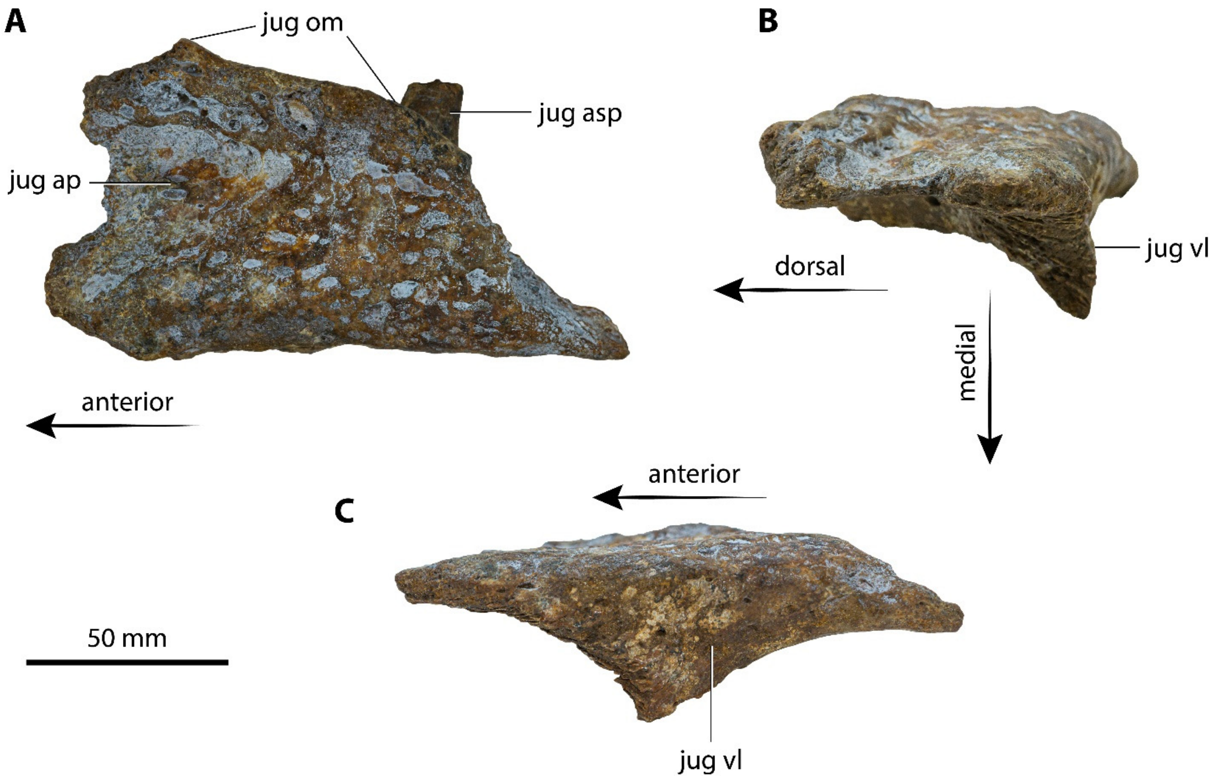

Figure S1.10 ***Quinkana*** **sp. indet., QMF1152, incomplete left jugal.** QMF1152 in (A) lateral, (B) anterior, and (C) ventral views. For a high-resolution version of this figure, see the PDF file of **Figure S1.10** provided as a supplementary file. Abbreviations: **jug ap**, anterior process of the jugal; **jug asp**, ascending process of the jugal; **jug om**, orbital margin of the jugal; **jug vl**, ventral lamina of the jugal.

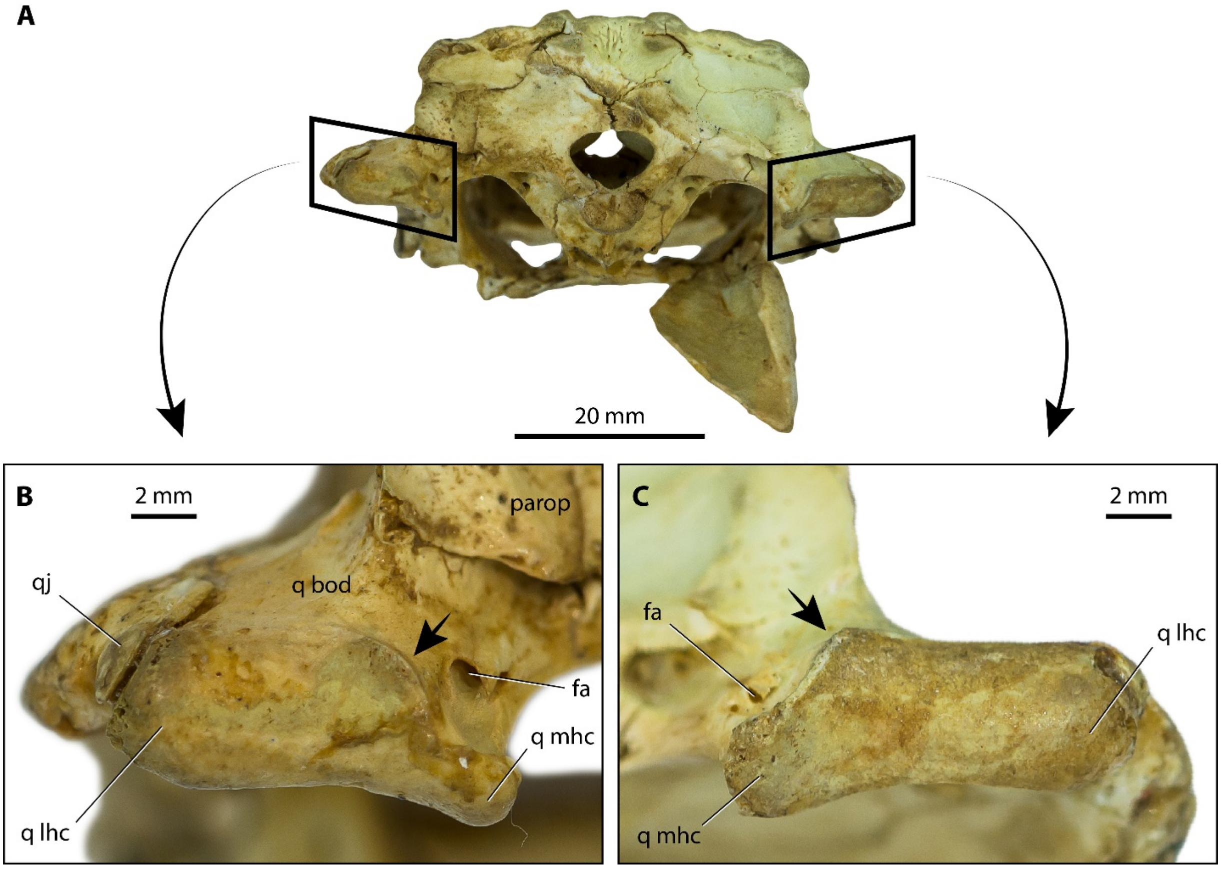

Figure S1.11 ***Trilophosuchus rackhami*** **Willis, 1993, QMF16856, holotype, close ups of the condylar surfaces of the left and right quadrates.** (A) Cranium in posterior view, with the black boxes indicating the portions shown in (B) and (C). (B) Close up of the condylar surface of the left quadrate. (C) Close up of the condylar surface of the right quadrate. The arrows in (B) and (C) indicate the dorsal projection between the lateral and medial hemicondyles. For a high-resolution version of this figure, see the PDF file of **Figure S1.11** provided as a supplementary file. Abbreviations: **fa**, foramen aëreum; **parop**, paroccipital process; **q bod**, body of the quadrate; **q lhc**, lateral hemicondyle of the quadrate; **q mhc**, medial hemicondyle of the quadrate; **qj**, quadratojugal.

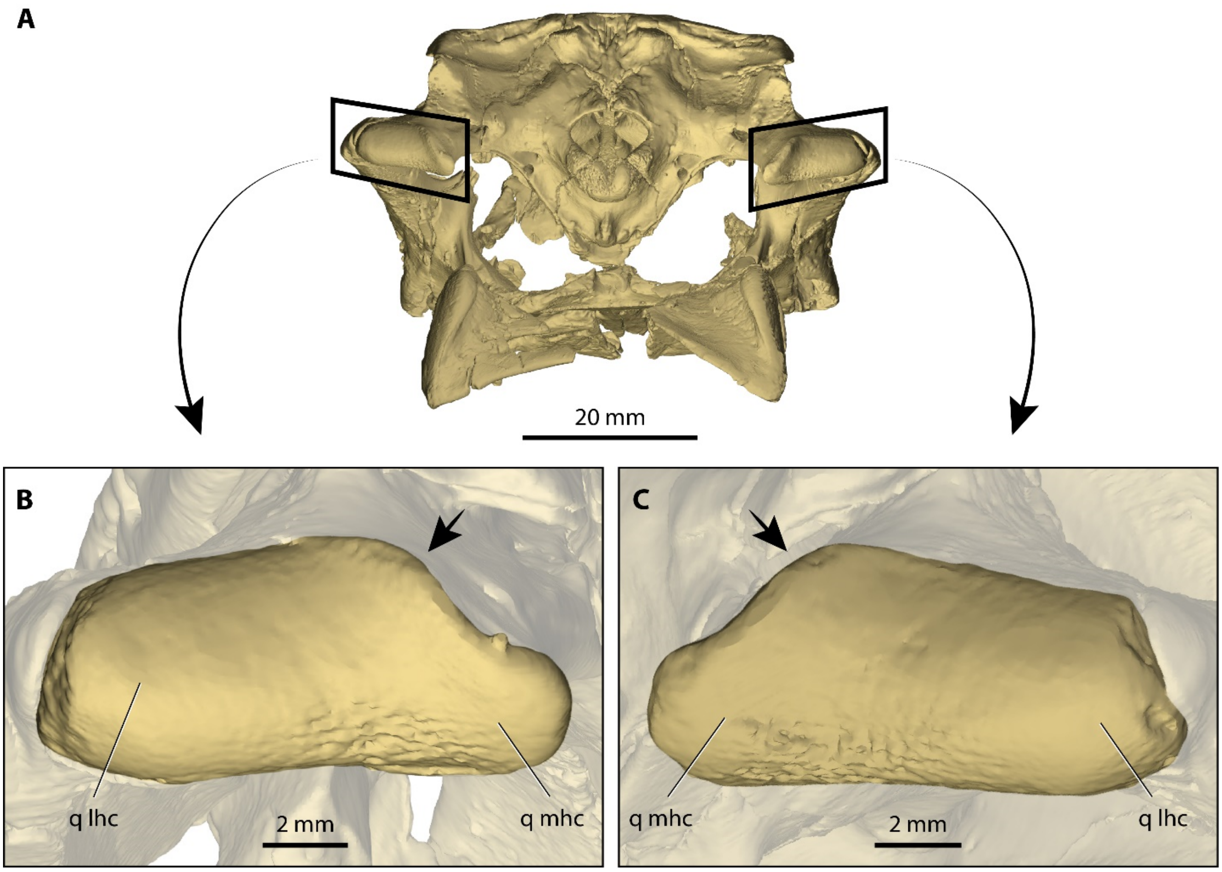

Figure S1.12 ***Trilophosuchus rackhami*** **Willis, 1993, QMF16856, holotype, close ups of the condylar surfaces of the left and right quadrates.** (A) Digital model of the cranium in posteroventral view, with the black boxes indicating the portions shown in (B) and (C). (B) Close up of the condylar surface of the left quadrate, with the condylar surface in focus. (C) Close up of the condylar surface of the right quadrate, with the condylar surface in focus. The arrows in (B) and (C) indicate the dorsal projection between the lateral and medial hemicondyles. For a high-resolution version of this figure, see the PDF file of **Figure S1.12** provided as a supplementary file. Abbreviations: **q lhc**, lateral hemicondyle of the quadrate; **q mhc**, medial hemicondyle of the quadrate.

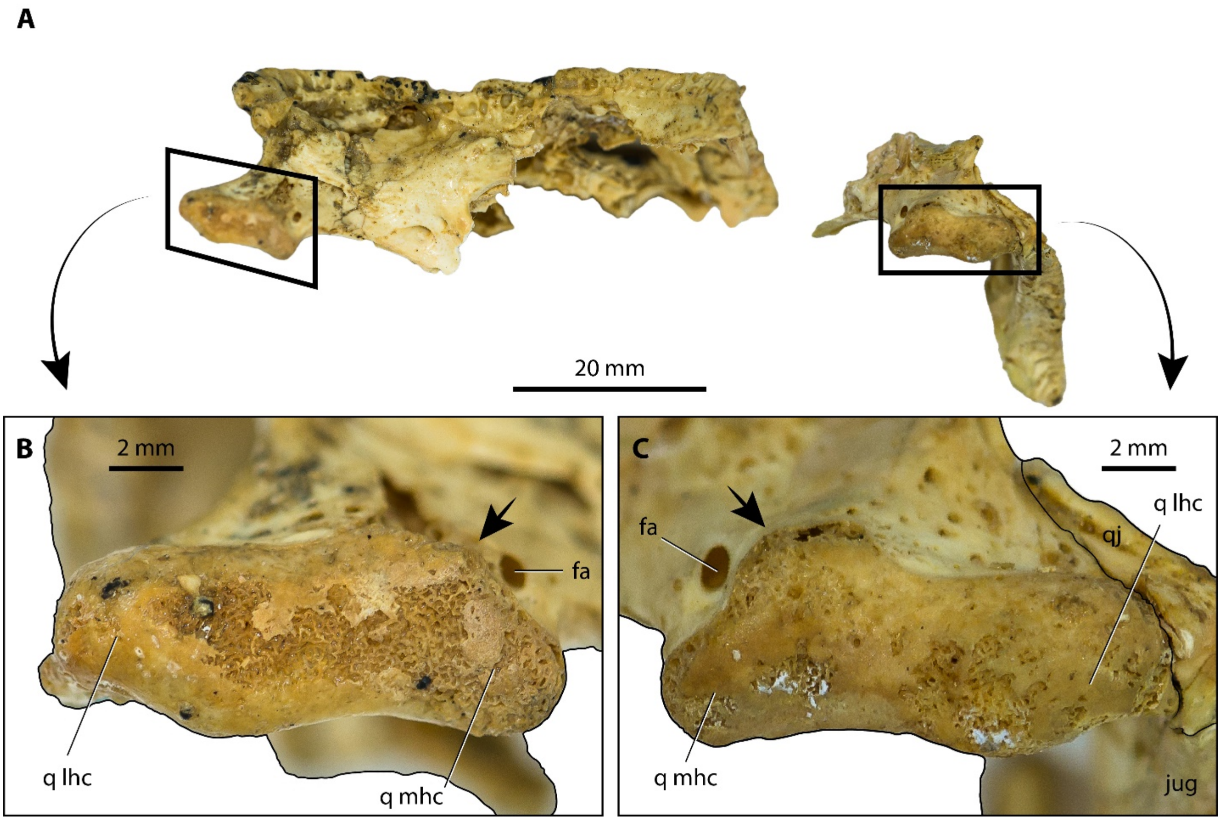

Figure S1.13 ***Mekosuchus sanderi*** **Willis, 2001, QMF31166, close ups of the condylar surfaces of the left and right quadrates.** (A) Cranium in posterior view along with associated right quadrate, quadratojugal and jugal. Note that in (A), the piece comprised of the right quadrate, quadratojugal and jugal is not in its correct anatomical position relative to the rest of the cranium. The black boxes in (A) indicate the portions shown in (B) and (C). (B) Close up of the condylar surface of the left quadrate. (C) Close up of the condylar surface of the right quadrate. The arrows in (B) and (C) indicate the dorsal projection between the lateral and medial hemicondyles. For a high-resolution version of this figure, see the PDF file of **Figure S1.13** provided as a supplementary file. Abbreviations: **fa**, foramen aëreum; **jug**, jugal; **q lhc**, lateral hemicondyle of the quadrate; **q mhc**, medial hemicondyle of the quadrate; **qj**, quadratojugal.

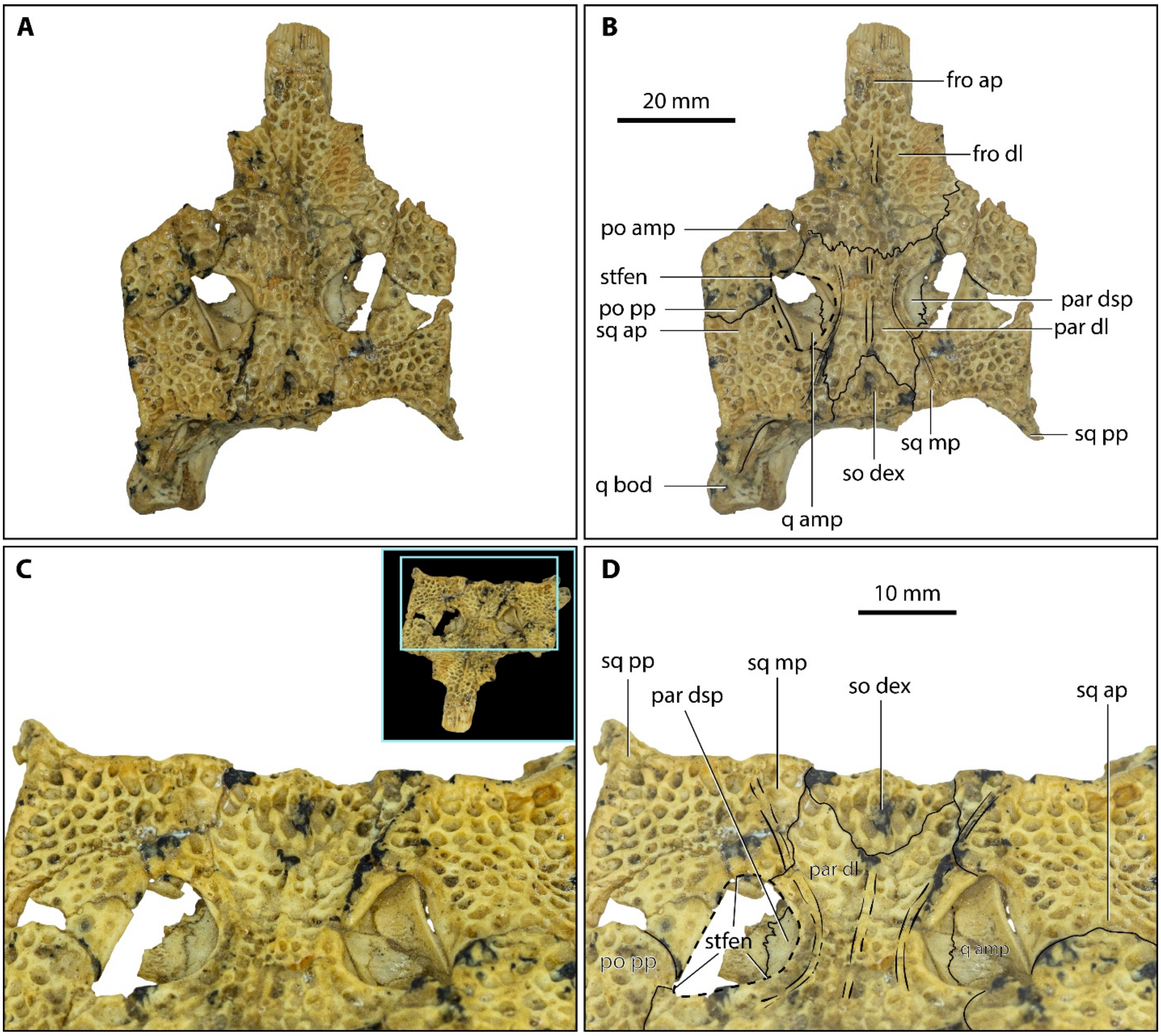

Figure S1.14 ***Mekosuchus sanderi*** **Willis, 2001, QMF31166, cranial table.** (A) Non-annotated photograph, and (B) annotated photograph of the partial cranium in dorsal view. (C) Non-annotated photograph, and (D) annotated photograph of the partial cranium in anterodorsal view, with a close up on the region around the supratemporal fenestrae. For a high-resolution version of this figure, see the PDF file of **Figure S1.14** provided as a supplementary file. Abbreviations: **fro ap**, anterior process of the frontal; **fro dl**, dorsal lamina of the frontal; **par dl**, dorsal lamina of the parietal; **par dsp**, descending process of the parietal; **po amp**, anteromedial process of the postorbital; **po pp**, posterior process of the postorbital; **q amp**, anteromedial process of the quadrate; **q bod**, body of the quadrate; **so dex**, dorsal exposure of the supraoccipital; **sq ap**, anterior process of the squamosal; **sq mp**, medial process of the squamosal; **sq pp**, posterior process of the squamosal; **stfen**, supratemporal fenestra.

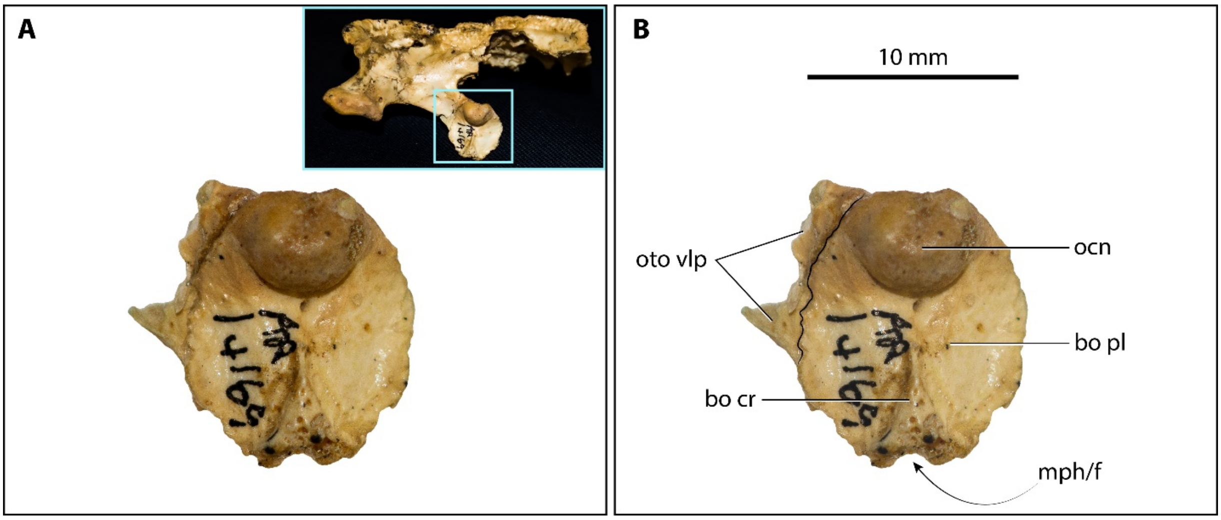

Figure S1.15 ***Mekosuchus sanderi*** **Willis, 2001, QMF31166, basioccipital.** (A) Non-annotated photograph, and (B) annotated photograph of the basioccipital in posterior view. Note that the basioccipital piece (along with the fragment from the ventrolateral process of the otoccipital) is broken off from the rest of the cranium. For a high-resolution version of this figure, see the PDF file of **Figure S1.15** provided as a supplementary file. Abbreviations: **bo cr**, basioccipital crest; **bo pl**, basioccipital plate; **mph/f**, median pharyngeal canal/foramen; **ocn**, occipital condyle; **oto vlp**, ventrolateral process of the otoccipital.

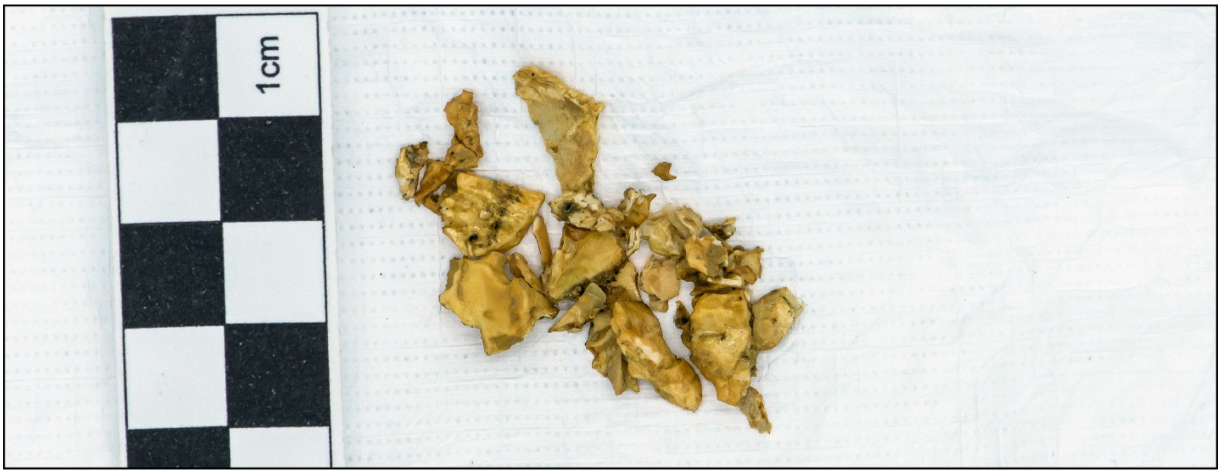

Figure S1.16 **Nondescript fragments associated with the holotype specimen, QMF16856, of *Trilophosuchus rackhami* Willis, 1993.**

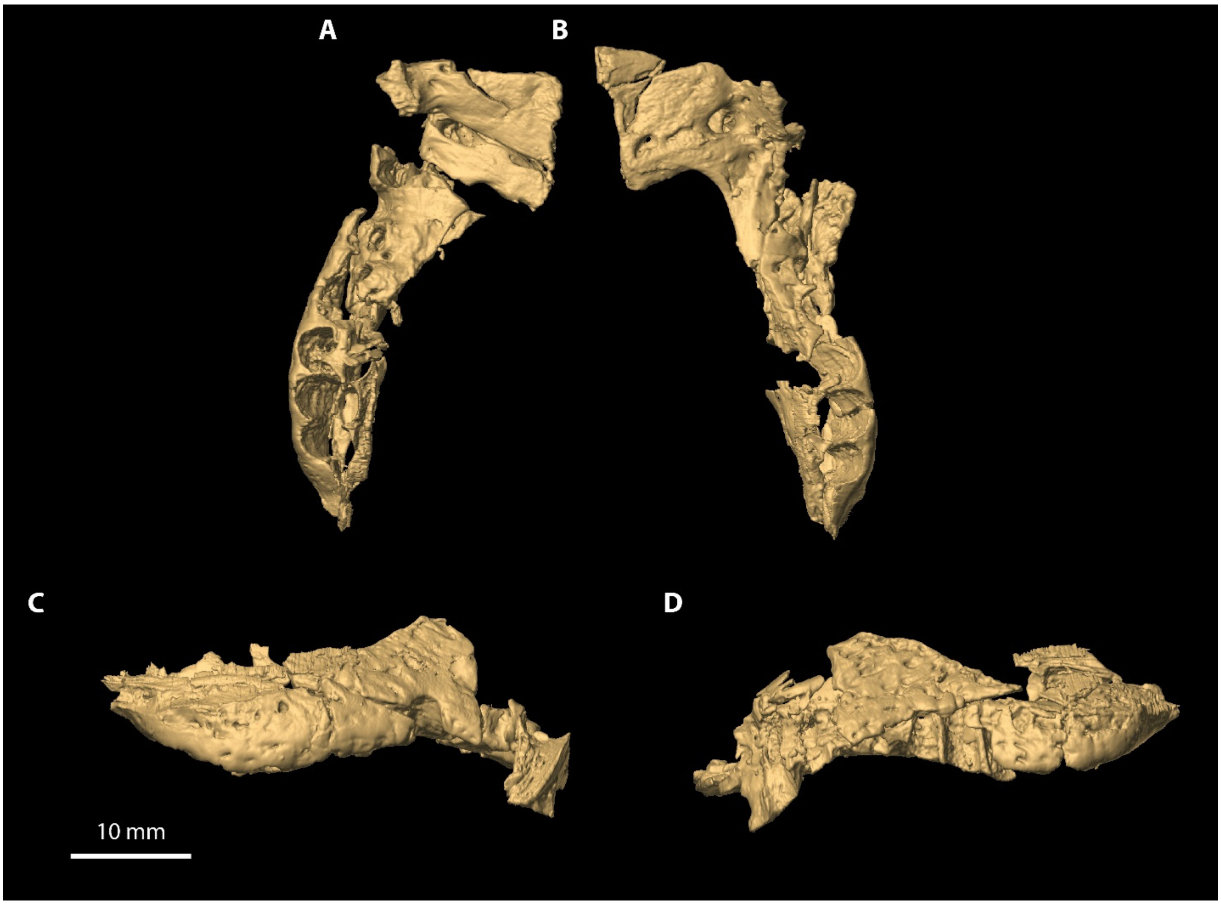

Figure S1.17 Maxillae of *Trilophosuchus rackhami* Willis, 1993, QMF16856, holotype. Right maxilla in (A) ventral, and (C) lateral views. Left maxilla in (B) ventral, and (D) lateral views. For the annotated version of this figure, see Figure 4 of the main text.

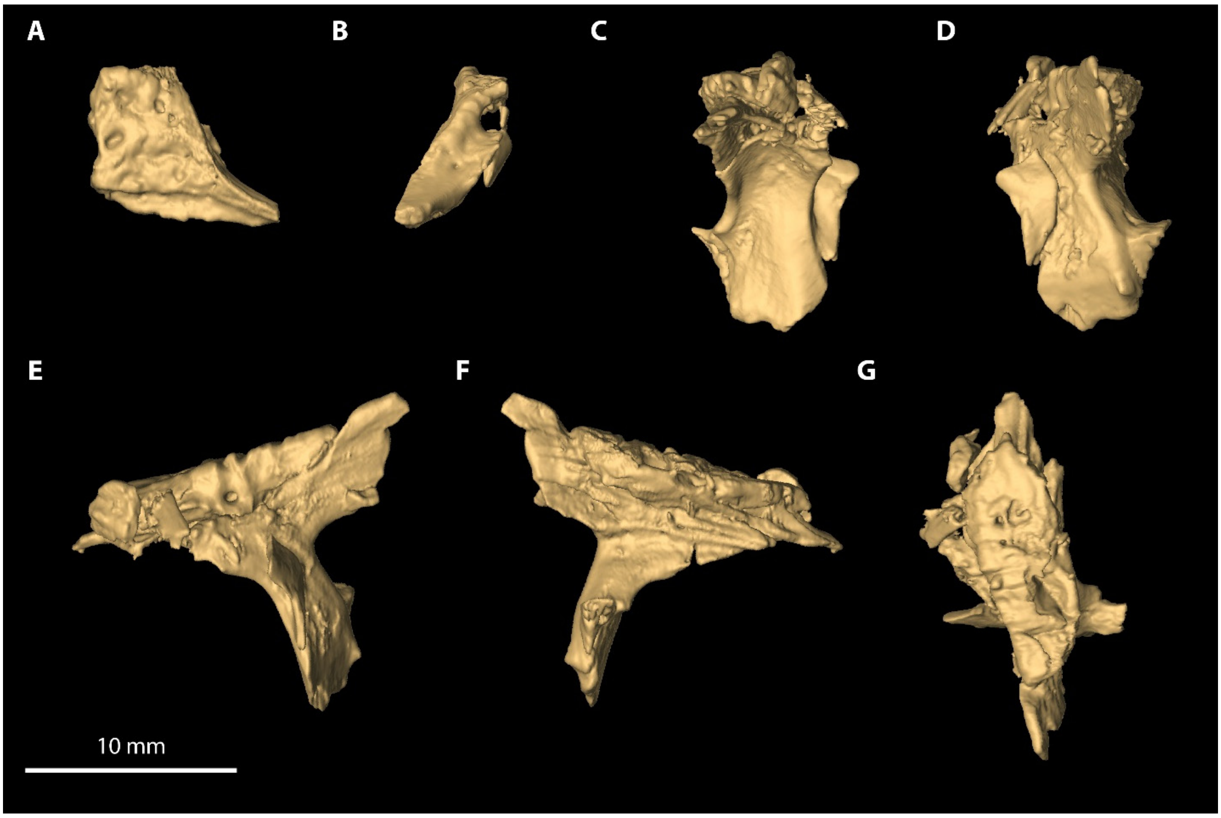

Figure S1.18 Left lacrimal and prefrontal of *Trilophosuchus rackhami* Willis, 1993, QMF16856, holotype. Left lacrimal in (A) lateral, and (B) posterior views. Left prefrontal in (C) anterior, (D) posterior, (E) lateral, (F) medial, and (G) dorsal views. For the annotated version of this figure, see Figure 5 of the main text.

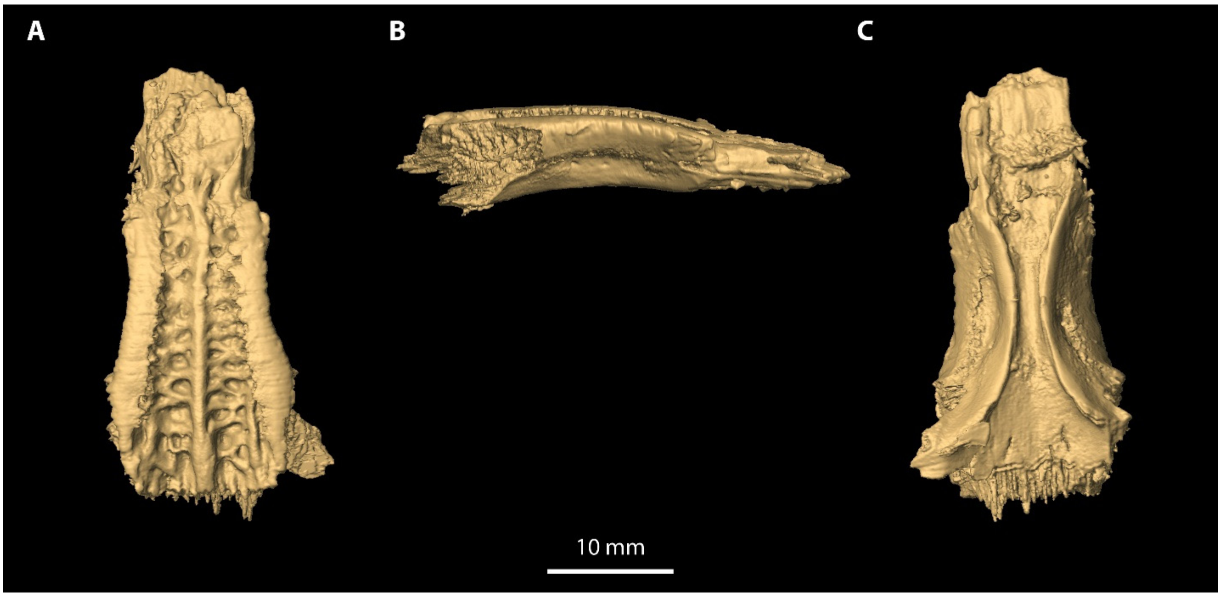

Figure S1.19 Frontal of *Trilophosuchus rackhami* Willis, 1993, QMF16856, holotype. Frontal in (A) dorsal, (B) right lateral, and (C) ventral views. For the annotated version of this figure, see Figure 7 of the main text.

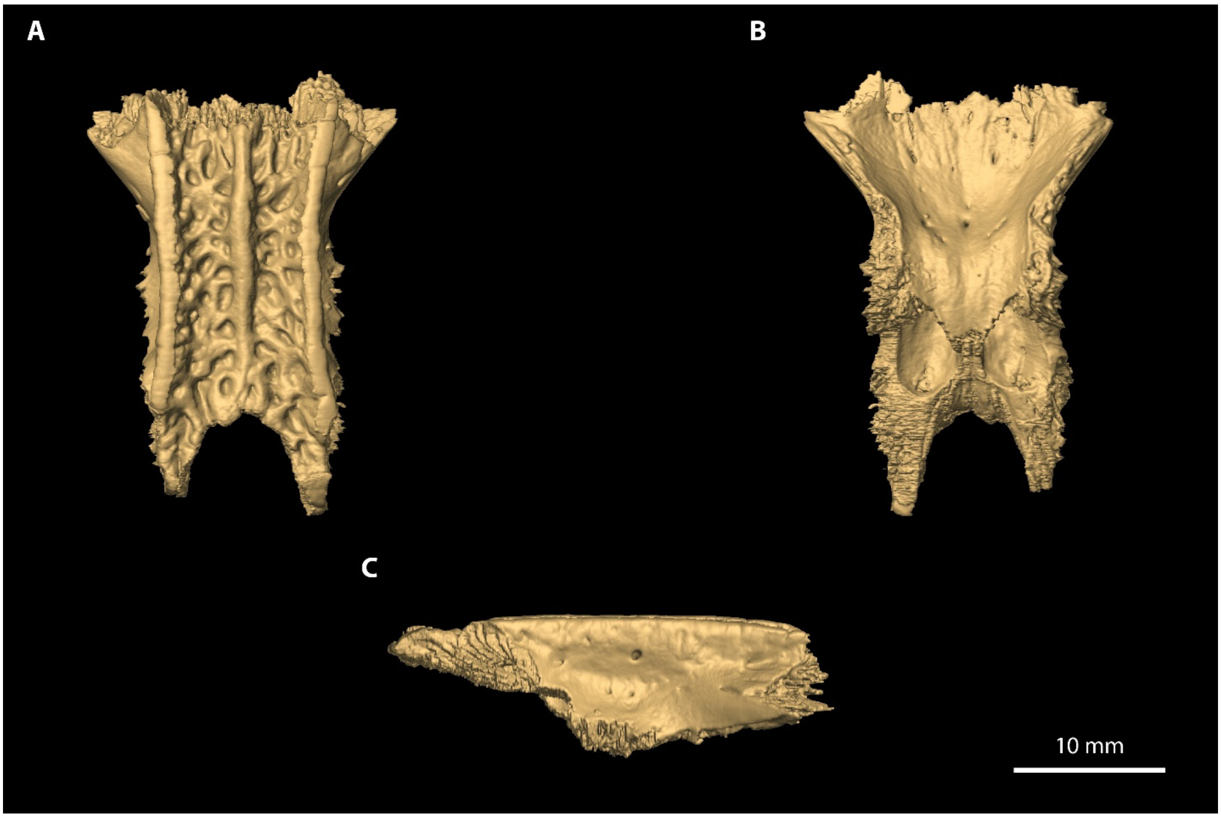

Figure S1.20 Parietal of *Trilophosuchus rackhami* Willis, 1993, QMF16856, holotype. Parietal in (A) dorsal, (B) ventral, and (C) right lateral views. For the annotated version of this figure, see Figure 8 of the main text.

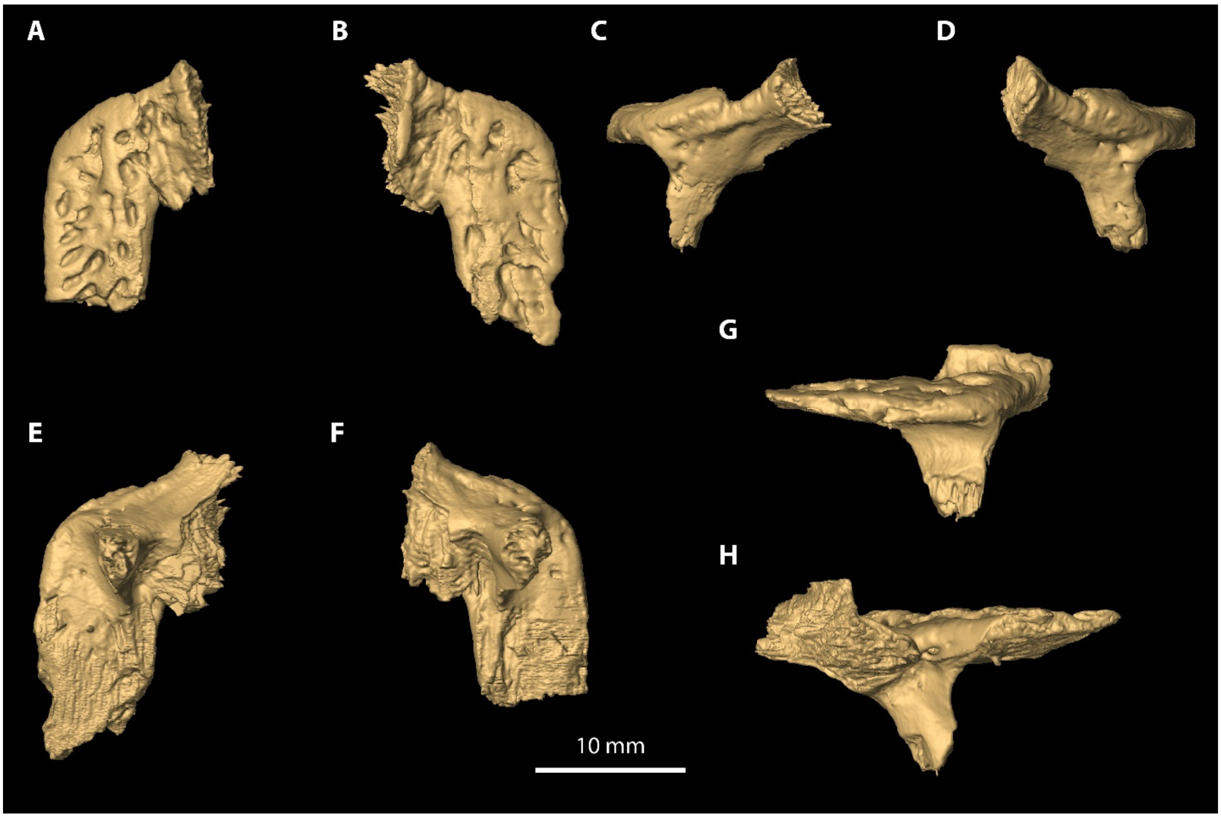

Figure S1.21 Postorbitals of *Trilophosuchus rackhami* Willis, 1993, QMF16856, holotype. Left postorbital in (A) dorsal, (D) anterior, and (F) ventral views. Right postorbital in (B) dorsal, (C) anterior, (E) ventral, (G) lateral, and (H) oblique medial views. For the annotated version of this figure, see Figure 9 of the main text.

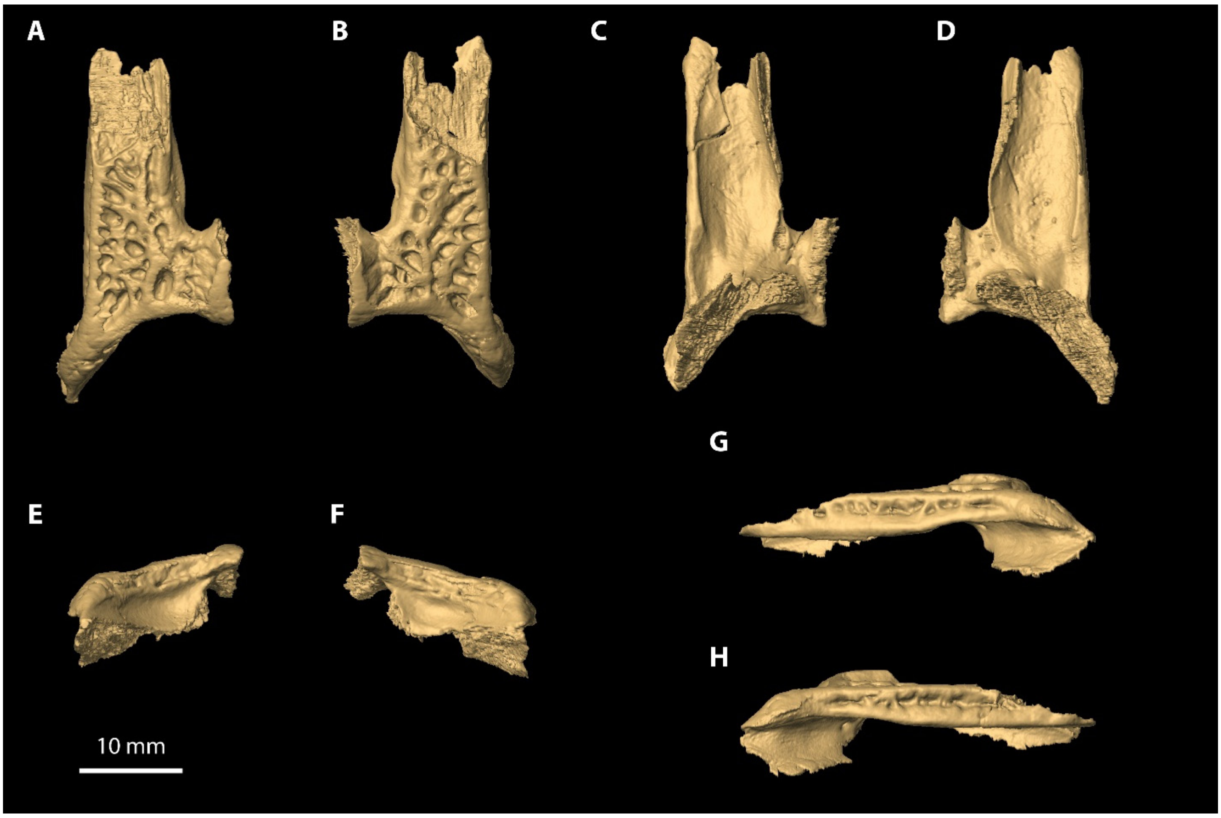

Figure S1.22 Squamosals of *Trilophosuchus rackhami* Willis, 1993, QMF16856, holotype. Left squamosal in (A) dorsal, (D) ventral, (E) posterior, and (G) lateral views. Right squamosal in (B) dorsal, (C) ventral, (F) posterior, and (H) lateral views. For the annotated version of this figure, see Figure 10 of the main text.

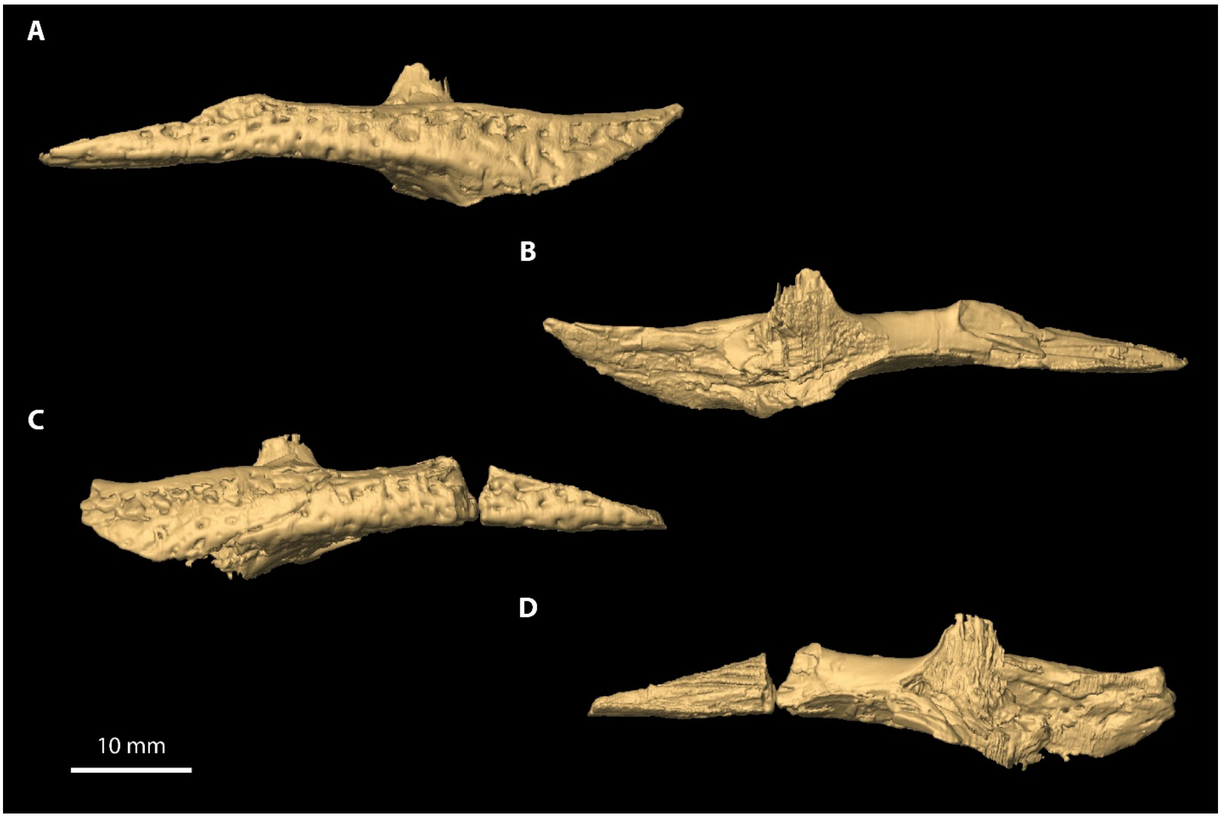

Figure S1.23 Jugals of *Trilophosuchus rackhami* Willis, 1993, QMF16856, holotype. Right jugal in (A) lateral, and (B) medial views. Left jugal in (C) lateral, and (D) medial views. For the annotated version of this figure, see Figure 11 of the main text.

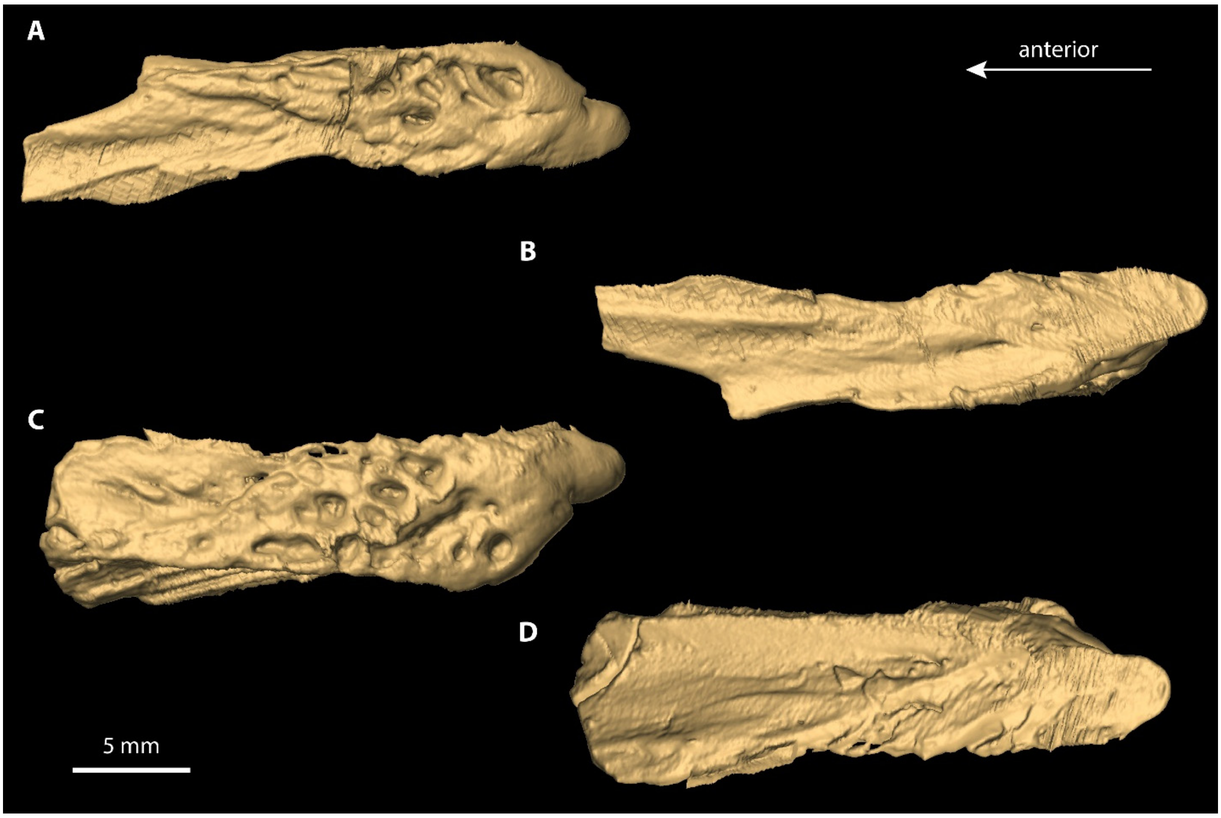

Figure S1.24 Quadratojugals of *Trilophosuchus rackhami* Willis, 1993, QMF16856, holotype. Right quadratojugal in (A) dorsolateral, and (B) ventromedial views. Left quadratojugal in (C) dorsolateral, and (D) ventromedial views. For the annotated version of this figure, see Figure 12 of the main text.

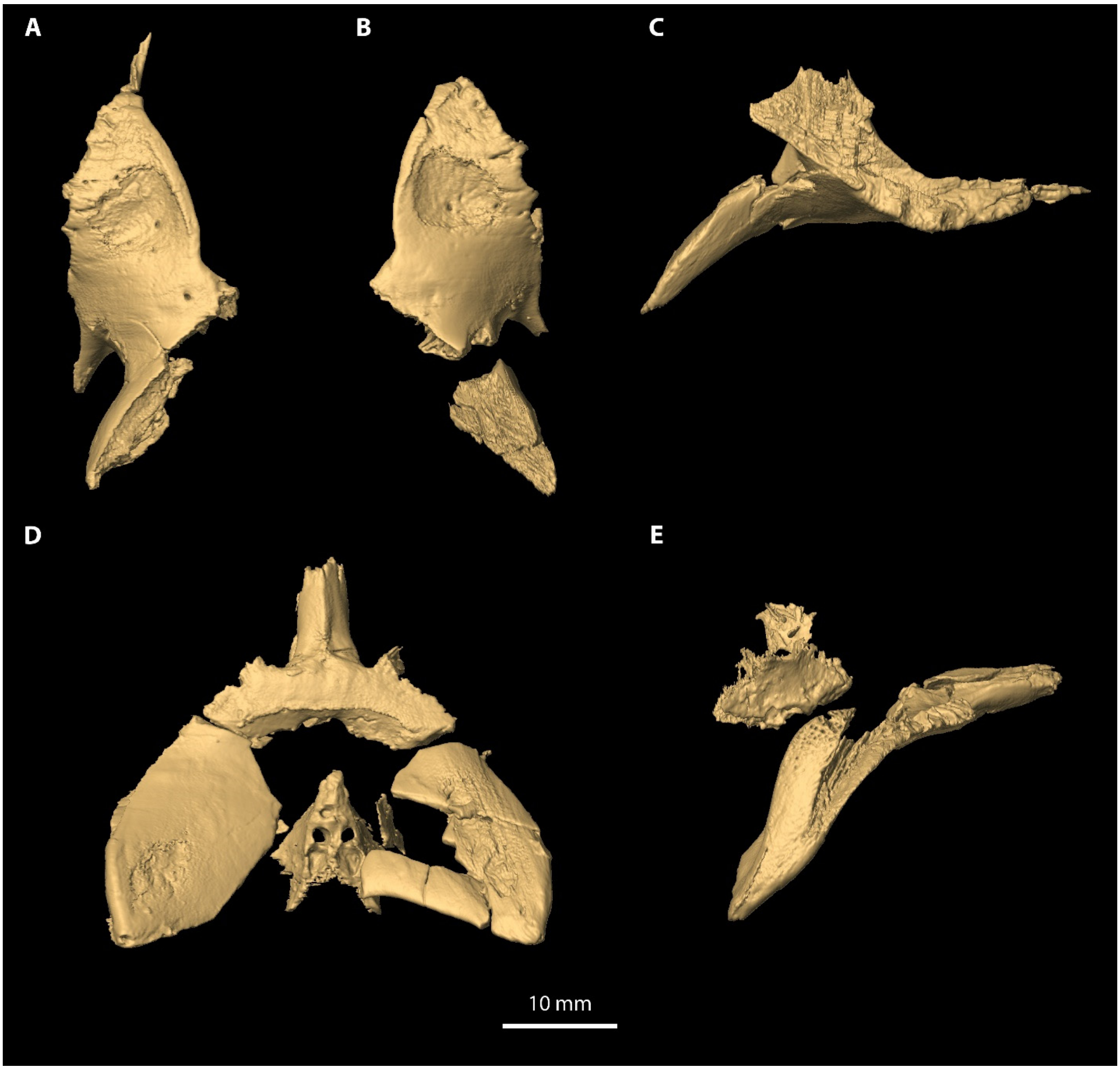

Figure S1.25 Ectopterygoids and pterygoid of *Trilophosuchus rackhami* Willis, 1993, QMF16856, holotype. Right ectopterygoid in (A) ventral, and (C) lateral views. Left ectopterygoid in (B) ventral view. Pterygoid in (D) ventral, and (E) right lateral views. For the annotated version of this figure, see Figure 13 of the main text.

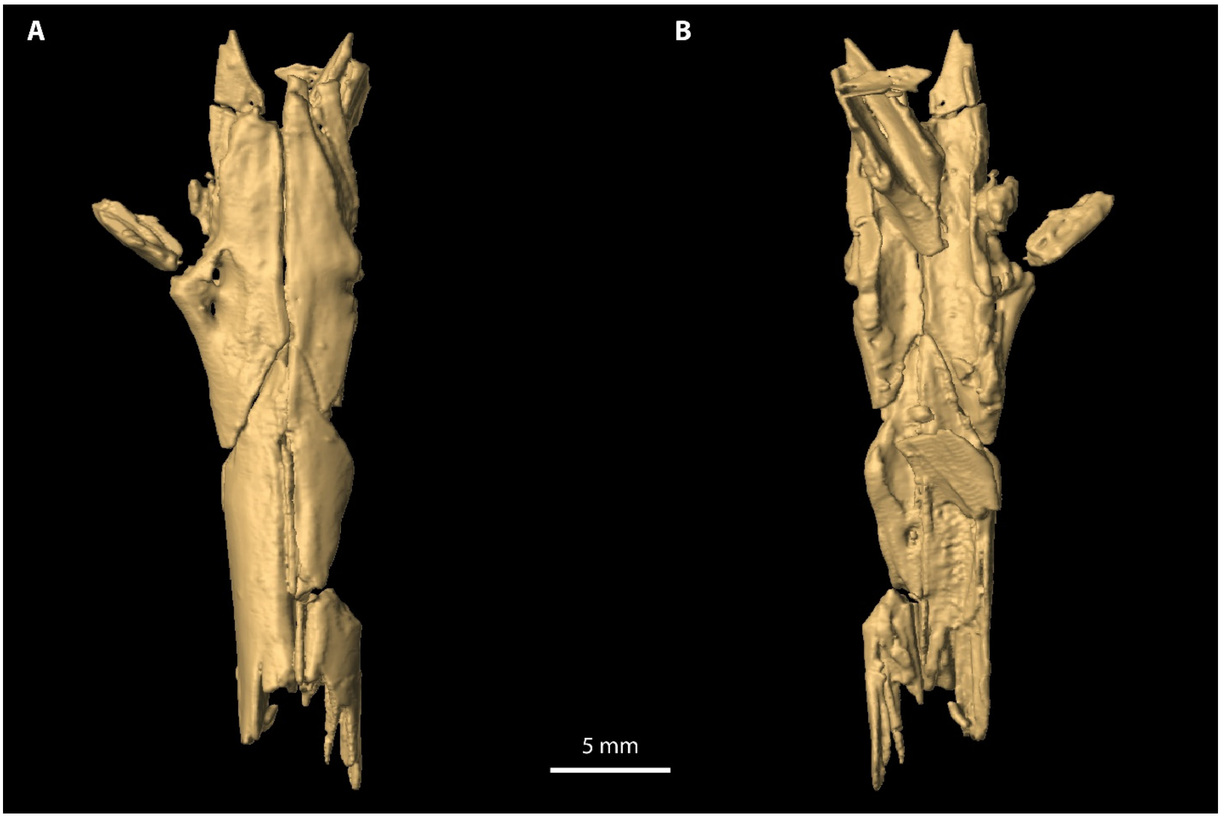

Figure S1.26 Palatines of *Trilophosuchus rackhami* Willis, 1993, QMF16856, holotype. Palatines in (A) ventral, and (B) dorsal (internal) views. For the annotated version of this figure, see Figure 14 of the main text.

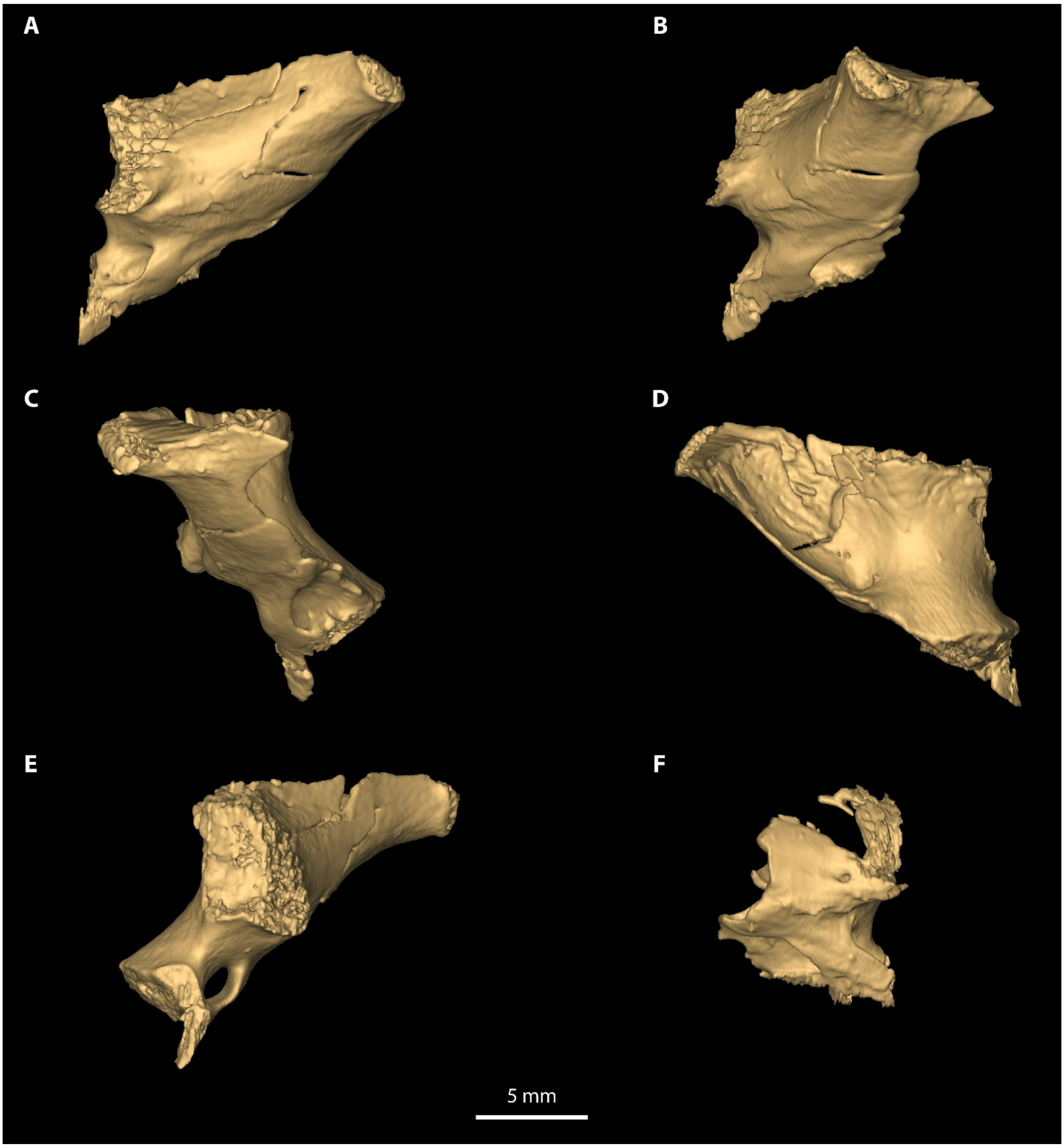

Figure S1.27 Laterosphenoids of *Trilophosuchus rackhami* Willis, 1993, QMF16856, holotype. Right laterosphenoid in (A) lateral, (B) anterolateral, (C) anterior, (D) medial, and (E) posterior views. Left laterosphenoid in (F) lateral view. For the annotated version of this figure, see Figure 15 of the main text.

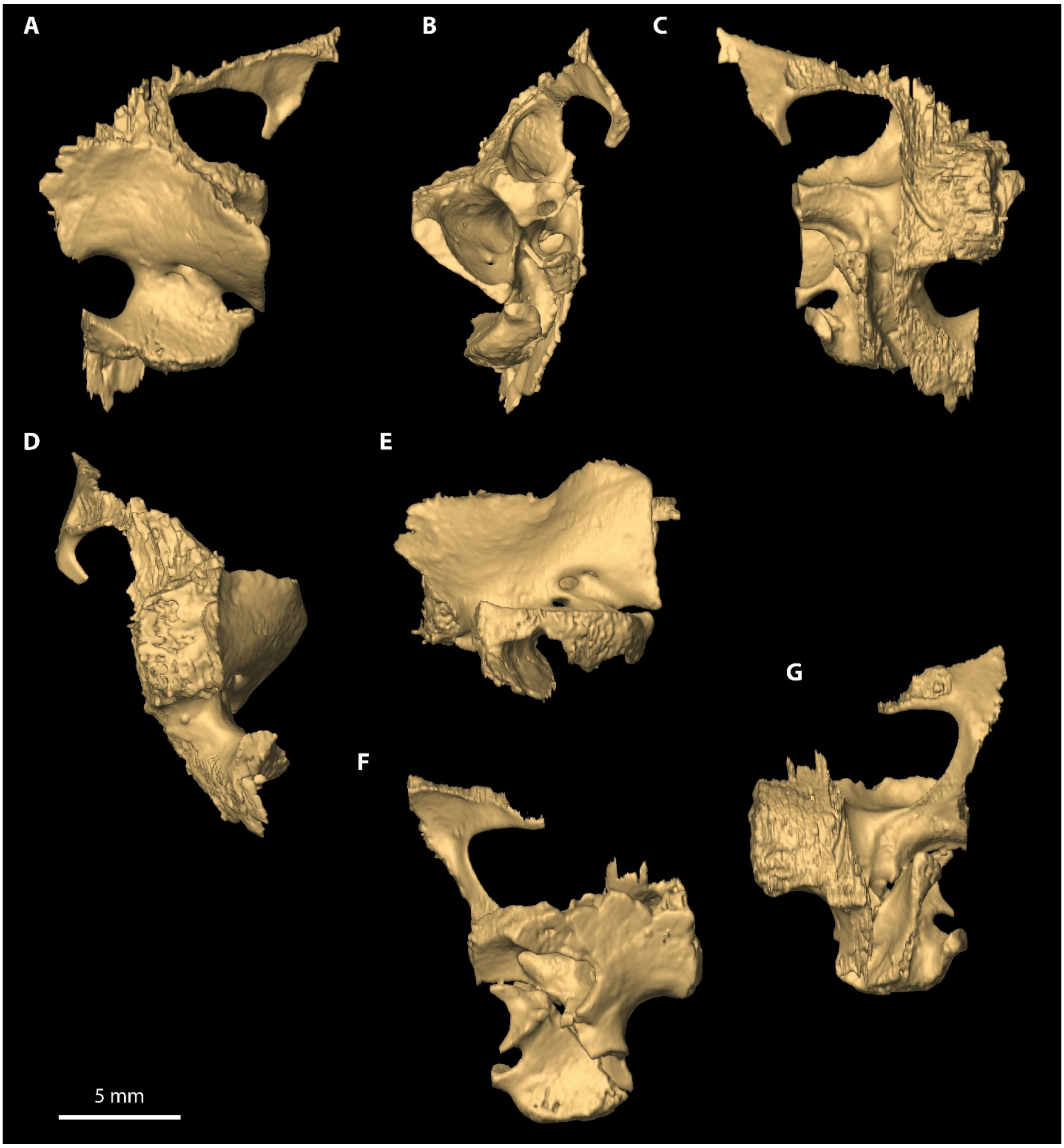

Figure S1.28 Prootics of *Trilophosuchus rackhami* Willis, 1993, QMF16856, holotype. Right prootic in (A) medial, (B) posterior, (C) lateral, (D) anterior, and (E) ventral views. Left prootic in (F) medial, and (G) lateral views. For the annotated version of this figure, see Figure 16 of the main text.

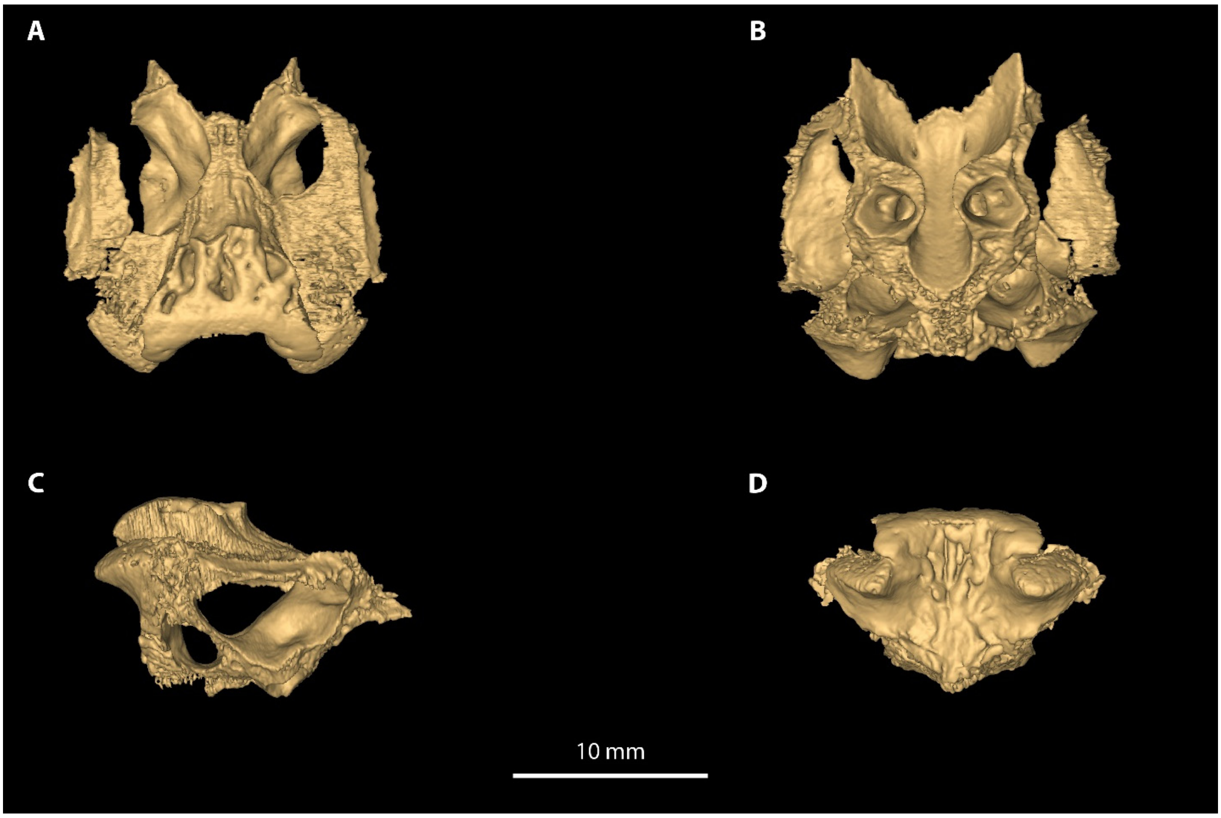

Figure S1.29 Supraoccipital of *Trilophosuchus rackhami* Willis, 1993, QMF16856, holotype. Supraoccipital in (A) dorsal, (B) ventral, (C) right lateral, and (D) posterior views. For the annotated version of this figure, see Figure 17 of the main text.

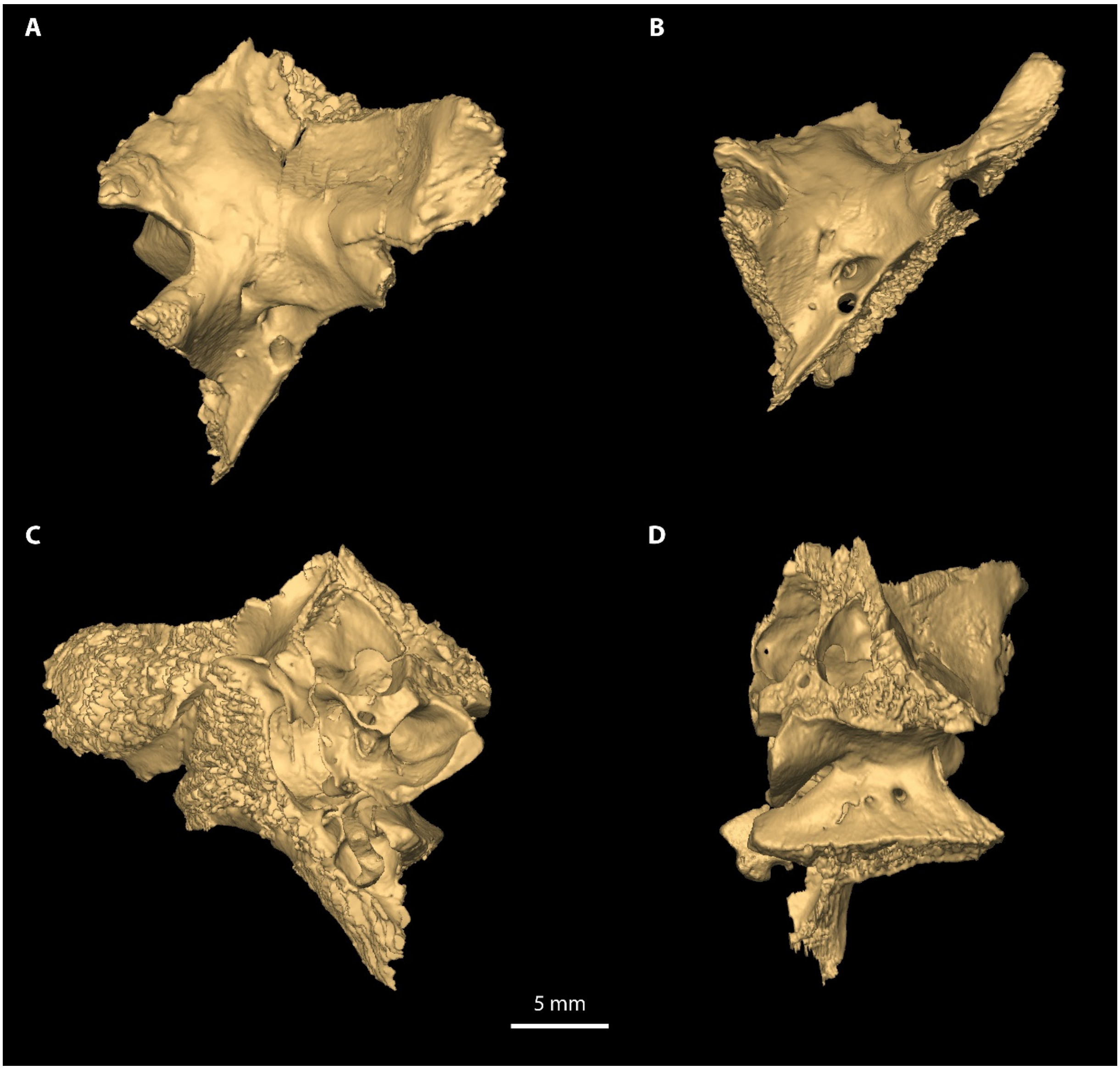

Figure S1.30 Right otoccipital of *Trilophosuchus rackhami* Willis, 1993, QMF16856, holotype. Right otoccipital in (A) posterior, (B) oblique ventral, (C) anterior, and (D) medial views. For the annotated version of this figure, see Figure 18 of the main text.

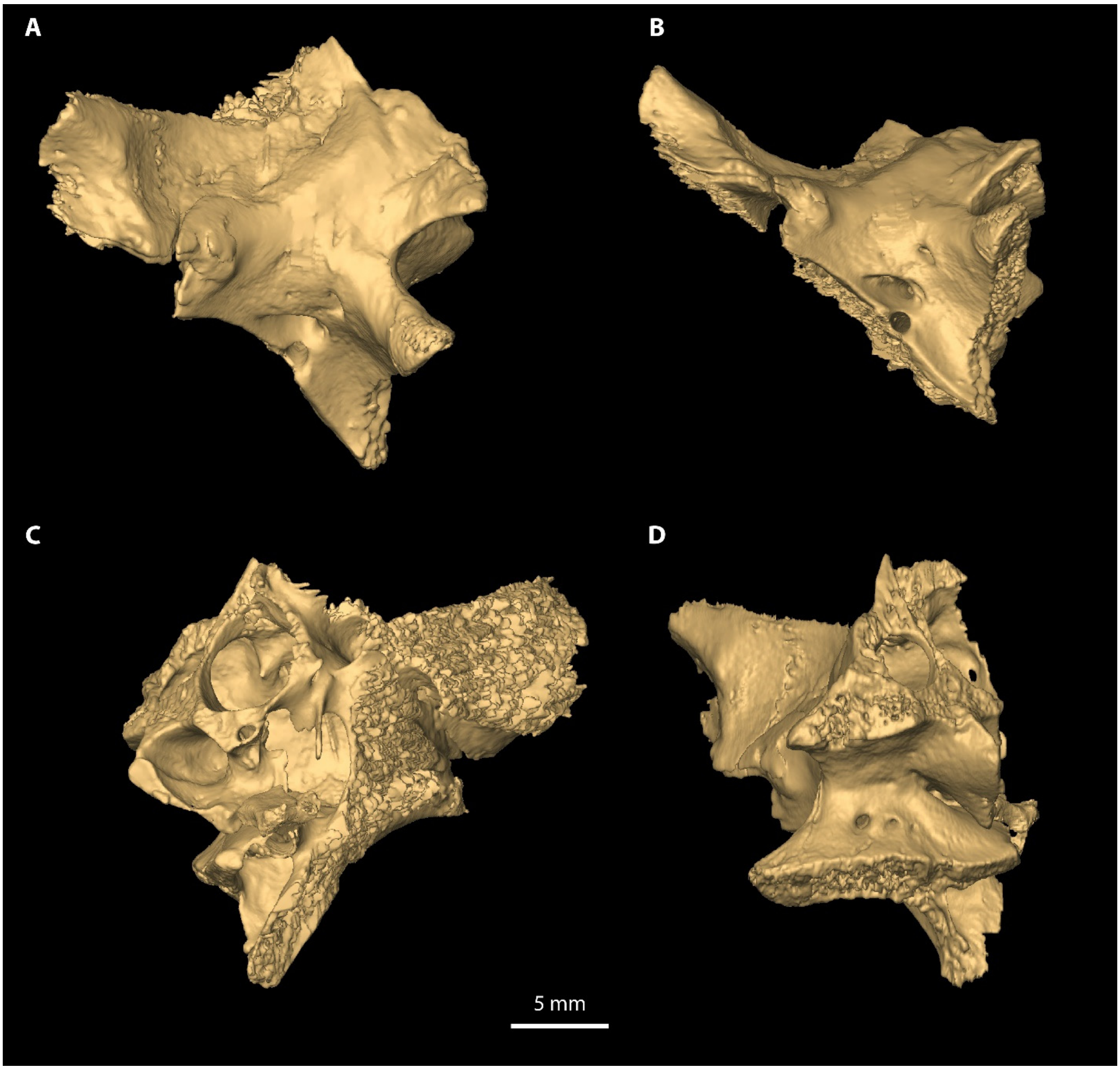

Figure S1.31 Left otoccipital of *Trilophosuchus rackhami* Willis, 1993, QMF16856, holotype. Left otoccipital in (A) posterior, (B) oblique ventral, (C) anterior, and (D) medial views. For the annotated version of this figure, see Figure 19 of the main text.

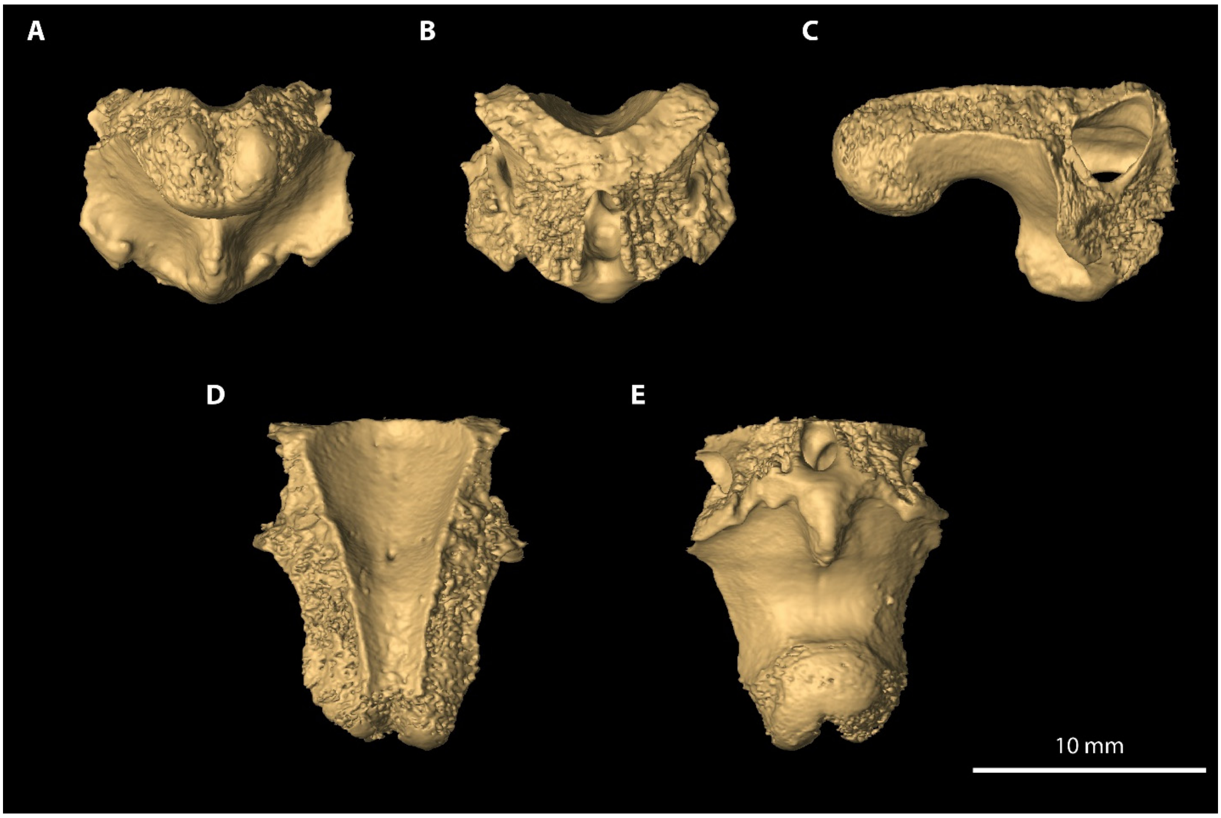

Figure S1.32 Basioccipital of *Trilophosuchus rackhami* Willis, 1993, QMF16856, holotype. Basioccipital in (A) posterior, (B) anterior, (C) right lateral, (D) dorsal, and (E) ventral views. For the annotated version of this figure, see Figure 20 of the main text.

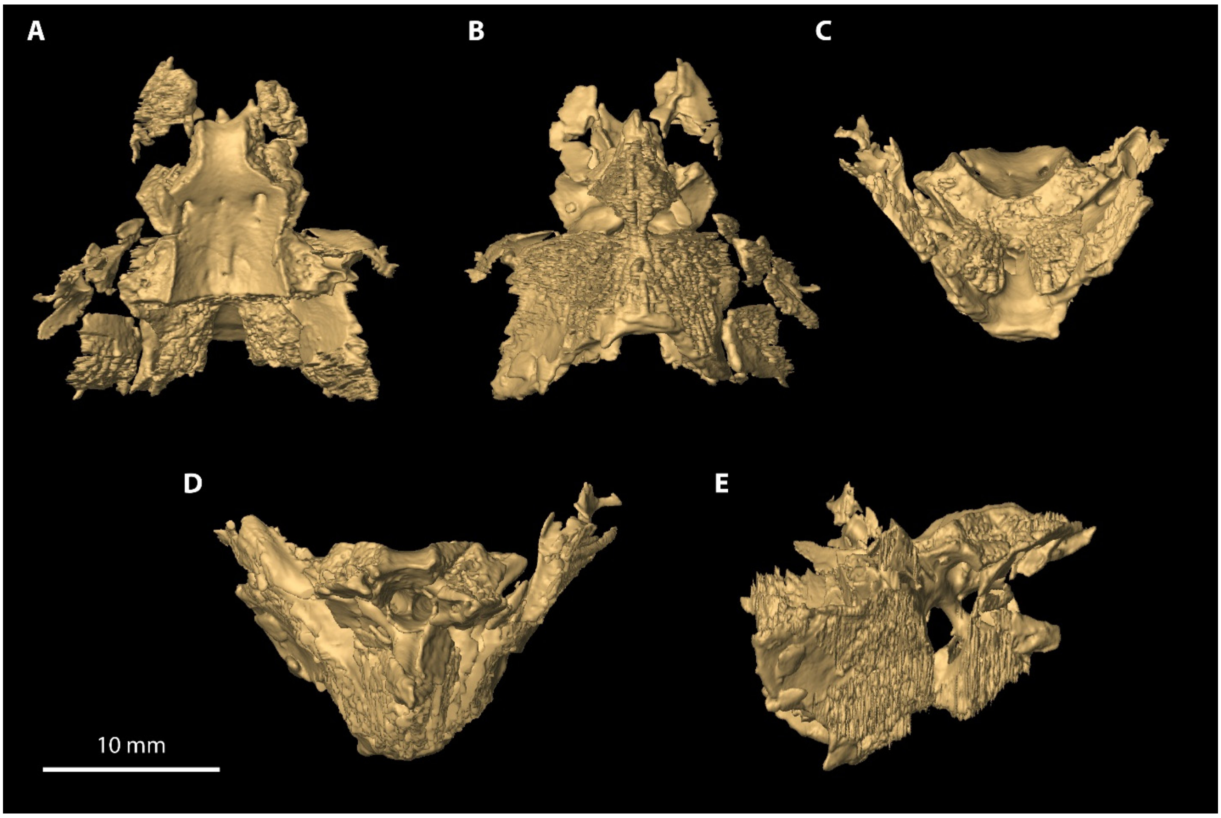

Figure S1.33 Parabasisphenoid of *Trilophosuchus rackhami* Willis, 1993, QMF16856, holotype. Parabasisphenoid in (A) dorsal, (B) ventral, (C) posterior, (D) anterior, and (E) right lateral views. For the annotated version of this figure, see Figure 21 of the main text.

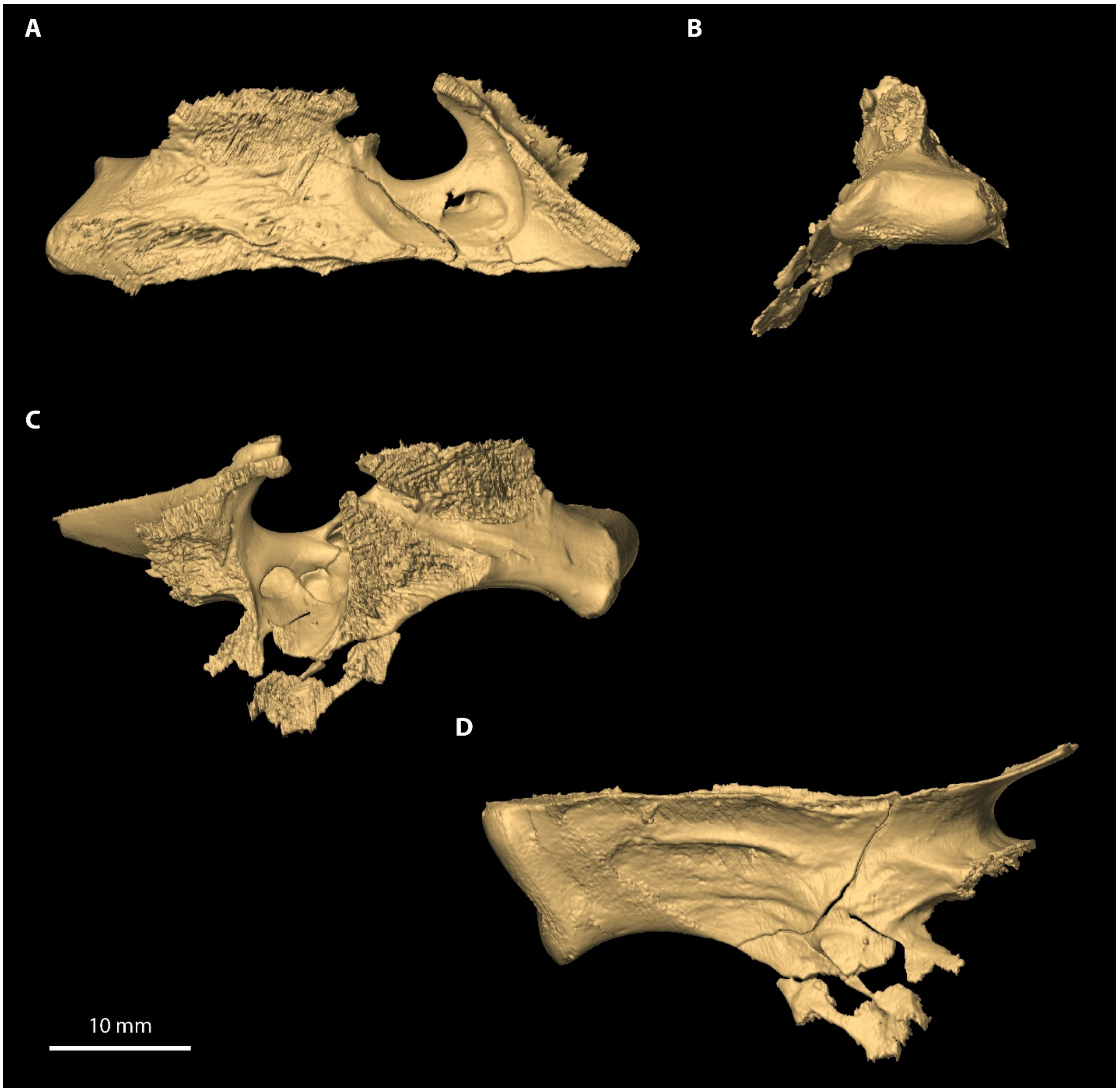

Figure S1.34 Right quadrate of *Trilophosuchus rackhami* Willis, 1993, QMF16856, holotype. Right quadrate in (A) dorsal, (B) posterior, (C) medial, and (D) ventral views. For the annotated version of this figure, see Figure 22 of the main text.

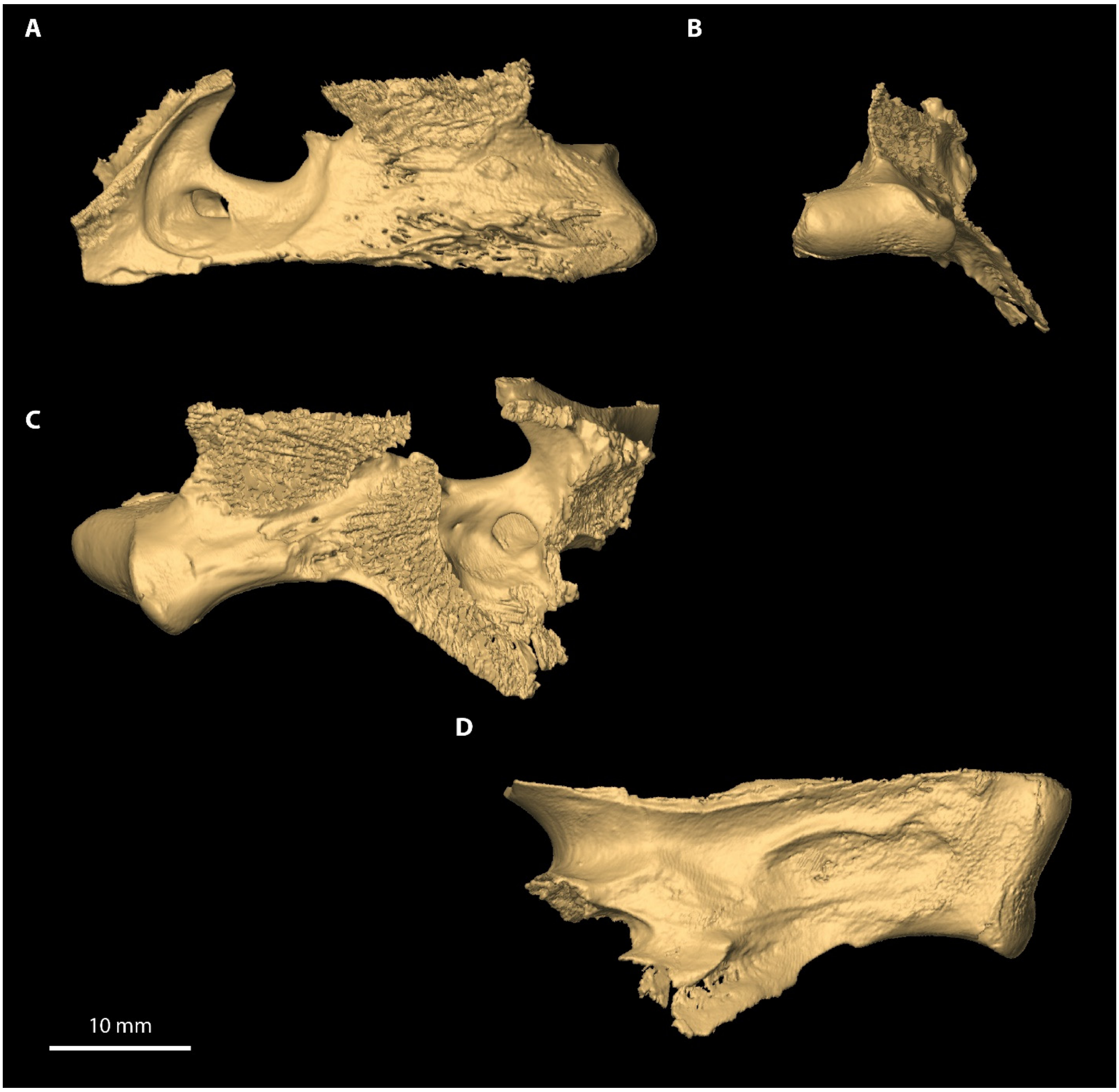

Figure S1.35 Left quadrate of *Trilophosuchus rackhami* Willis, 1993, QMF16856, holotype. Left quadrate in (A) dorsal, (B) posterior, (C) medial, and (D) ventral views. For the annotated version of this figure, see Figure 23 of the main text.

MORPHOLOGICAL COMPARISONS BETWEEN *TRILOPHOSUCHUS RACKHAMI* AND OTHER AUSTRALIAN CROCODYLIANS

The main focus of this section is to compare some notable morphological features of *Trilophosuchus rackhami* to other crocodylians, particularly mekosuchines. The taxa considered in the comparative anatomy are: the two Australian gavialoids *Gunggamarandu maunala* Ristevski *et al*., 2021 and *Harpacochampsa camfieldensis* Megirian *et al*., 1991; 15 mekosuchines: *Australosuchus clarkae* Willis & Molnar, 1991, two species of *Baru* Willis *et al*., 1990 (*Baru darrowi* Willis *et al*., 1990 and *Baru wickeni* Willis, 1997), *Kalthifrons aurivellensis* Yates & Pledge, 2016, three species of *Kambara* Willis *et al*., 1993 (*Kambara implexidens* Salisbury & Willis, 1996, *Kambara murgonensis* Willis *et al*., 1993, and *Kambara taraina* Buchanan, 2009), three species of *Mekosuchus* Balouet & Buffetaut, 1987 (*Mekosuchus inexpectatus* Balouet & Buffetaut, 1987, *Mekosuchus sanderi* Willis, 2001, and *Mekosuchus whitehunterensis* Willis, 1997), one species of *Paludirex* Ristevski *et al*., 2020a (*Paludirex vincenti* Ristevski *et al*., 2020a), and four species of *Quinkana* Molnar, 1981 (*Quinkana babarra* Willis & Mackness, 1996, *Quinkana fortirostrum* Molnar, 1981, *Quinkana meboldi* Willis, 1997, and *Quinkana timara* Megirian, 1994); and, the two extant crocodylids that inhabit Australia: *Crocodylus johnstoni* (Krefft, 1873) and *Crocodylus porosus* Schneider, 1801.

Thanks to a unique combination of morphological features, *Trilophosuchus rackhami* is readily discernable from other crocodylians. While much of the skeleton of *T. rackhami* remains unknown – at present, no dental, mandibular, or postcranial remains can be referred to it with confidence – the distinctive skull morphology of the taxon is highly informative (**Figs. 1** and **2**). Although several anatomical characteristics are unique to *T. rackhami* (such as the lateroventrally sloping cranial table, the three continuous longitudinal crests on the cranial table, the relations between the maxilla, lacrimal and jugal around the orbit, the concavity on the ventral surface of the ectopterygoid plate, and the morphology of the occipital lamina of the supraoccipital), some

occur in other mekosuchines. In the following section, we discuss some of the more notable features of *T. rackhami* in context of other crocodylians.

Inferring the morphology of the snout in *T. rackhami* is somewhat difficult due to the damage on that region in the holotype specimen. Fortunately, the preserved margins of the snout allow for a relatively confident interpretation of its gross morphology. As originally remarked by Willis (1993), the snout of *T. rackhami* would have been relatively short. Furthermore, the cross-sectional outline anterior to the orbits is strongly suggestive to have been trapezoidal (**Fig. S1.2B**). Based on the better-preserved left side (comprised by the ascending process of the maxilla and the facial lamina of the lacrimal), the snout has a sub-vertical lateral margin which is consistent with an altirostral morphology. Altirostry is present in several other mekosuchines such as species of *Quinkana*, but also *Baru* and *Mekosuchus*. A relatively short and broad altirostral snout, similar to that of *T. rackhami*, is inferred for *Mekosuchus inexpectatus*, *M. sanderi* and *M. whitehunterensis* (Holt *et al*., 2007; QMF31188 and QMF31051). In contrast, species of *Baru* and *Quinkana* tend to have proportionally longer altirostral snouts than *Trilophosuchus* Willis, 1993 or *Mekosuchus* (Willis *et al*., 1990; Megirian, 1994; Yates, 2017).

Trilophosuchus rackhami has elliptical supratemporal fenestrae (**Figs. 1A** and **2A**). A supratemporal fenestra with an elliptical outline is not a common occurrence in known mekosuchines, where most possess supratemporal fenestrae with either circular to sub-circular outlines or superficially 'D'-shaped outlines (e.g., Ristevski *et al*., 2020a, b). Similarly elliptical outlines of the supratemporal fenestrae to those of *T. rackhami* are present in *Kalthifrons aurivellensis* (Yates & Pledge, 2016; **Fig. S3.3C**; see also figures S1.14E and S1.14H in Ristevski *et al*., 2020b). A partial cranium referred to *Quinkana timara* (NMV P179632; Yates & Pledge, 2016) also displays an elliptical supratemporal fenestra with a length that is greater than its width (J. Ristevski pers. obs.).

Due to the shortness of the snout, the suborbital fenestrae of *T. rackhami* extend anteriorly to the level of either the sixth or seventh maxillary alveoli. In most crocodylians, the suborbital

fenestrae tend to reach no further than the ninth maxillary alveoli and often terminate at a more posterior level, as in most longirostrine taxa. Besides *T. rackhami*, other mekosuchines with suborbital fenestrae that extend far anteriorly include *B. darrowi* and *B. wickeni* (to the level of the seventh alveolus in both species; Willis *et al*., 1990, Willis, 1997, and Yates, 2017), *M. inexpectatus* (to the level of the sixth alveolus; Willis, 1997 and Holt *et al*., 2007), *M. kalpokasi* Mead *et al*., 2002 (to the level of the seventh alveolus; Mead *et al*., 2002), *M. sanderi* (to the level of the sixth alveolus; QMF31188), *M. whitehunterensis* (to the level of the seventh alveolus; QMF31051), *Q. babarra* (to the level of the sixth alveolus; QMF23220), and *Q. fortirostrum* (to the level of the seventh alveolus; Molnar, 1981).

One of the unique features of *T. rackhami* is the relation between the maxilla and the orbit. No other currently described crocodylian has a maxilla that closely approaches the orbital margin only to be excluded from the latter by a narrow contact formed by the lacrimal and jugal (**Fig. 6**). In other crocodylians, the maxilla is separated from the orbital margin by a comparatively wider contact between the lacrimal and jugal. The relation between the maxilla and the orbit in *T. rackhami* is closest to that of *Mekosuchus*, where the maxilla actually forms part of the lower orbital margin – this is an autapomorphy of *Mekosuchus* (Balouet & Buffetaut, 1987; Willis, 1997).

On the palatal surface of the maxilla, set close to the lingual alveolar margins is the maxillary foramen for the palatine ramus of the trigeminal nerve (**Figs. 4A** and **4B**). In *T. rackhami*, this foramen is enlarged and near in size to some maxillary alveoli. A similarly enlarged maxillary foramen for the palatine ramus of the trigeminal nerve is also present in other mekosuchines. This includes *Baru darrowi*, *B. wickeni* (Yates, 2017; NTM P8695-8 and NTM P91171-1), and *Quinkana babarra* (QMF23220). A conspicuously large foramen is also evident in *M. sanderi* (QMF31188) and *M. whitehunterensis* (QMF31051). Other Australian crocodylians, where known, possess proportionally smaller foramina on the palatal surfaces of their maxillae.

Among currently known mekosuchines, an anterior process of the frontal that is no more than 35% of the total anteroposterior length of the element is shared between *Trilophosuchus* and

Mekosuchus. In *T. rackhami* (QMF16856 and QMF16857) the anterior process of the frontal is ~28–29% of the total frontal length (**Figs. 7A**, **S2.1B** and **S2.1C**). In *M. sanderi* (**Fig. S1.14**) and *M. whitehunterensis* (QMF31052; figure 3 in Willis, 1997; figure S1.11D in Ristevski *et al*., 2020b), the anterior process of the frontal is also quite short, ranging from 30–35% of the total length of the bone. Additionally, the anterior process of the frontal of *Trilophosuchus* and *Mekosuchus* is relatively wide and with a blunt tip. Most crocodylians, where known, possess an intermediate condition where the anterior process of the frontal is between 35% and 60% the total length of the frontal. This is true of other mekosuchines that have the anterior process of the frontal sufficiently preserved (e.g., *B. wickeni*, *A. clarkae*, *Kambara* spp.; see Willis & Molnar, 1991, Salisbury & Willis, 1996, Buchanan, 2009, Yates, 2017, figure S1.6 in Ristevski *et al*., 2020b). An exception is the condition of *K. aurivellensis*, where the anterior process of the frontal is extremely elongated by comprising ~67% of the total length of the frontal (Yates & Pledge, 2016; **Fig. S3.3**). No other known mekosuchine has an anterior process of the frontal as long as that of *K. aurivellensis*. Outside of Mekosuchinae, a relatively short anterior process of the frontal is also present in species of *Purussaurus* Barbosa-Rodrigues, 1892 (see Langston, 1965 and Aguilera *et al*., 2006). Although not as common as the moderate condition, extremely long anterior frontal processes do occur in non-mekosuchine crocodyliforms, and are here recognized in the neosuchian *Theriosuchus pusillus* Owen, 1878, the 'thoracosaur' eusuchian *Eothoracosaurus mississippiensis* Brochu, 2004, the gavialoid *Piscogavialis jugaliperforatus* Kraus, 1998, and the crocodylid *Euthecodon arambourgi* Ginsburg & Buffetaut, 1978.

The combination of three continuous longitudinal crests adorning the cranial table is another autapomorphy of *Trilophosuchus*. On the other hand, a single midsagittal crest that extends over the frontal and/or parietal is not an uncommon occurrence among crocodylians, including some mekosuchines. The midsagittal crest of *T. rackhami* is prominent and continuous from the frontal and over to the parietal. In contrast, a relatively low midsagittal crest lying solely over the frontal is present in *K. aurivellensis* (Yates & Pledge, 2016). A midsagittal crest on the

dorsal surface of the frontal also occurs in *M. sanderi* and *M. whitehunterensis*, though in the known specimens for these two species the frontal crests are reduced in both acuteness and length when compared to *T. rackhami* (the midsagittal frontal crest is subtle in *M. sanderi* specimen QMF31187 and *M. whitehunterensis* specimen QMF31052). Other than *Trilophosuchus*, a midsagittal crest over the parietal is also recognized in *M. sanderi* (QMF31166), however the crest in this *M. sanderi* specimen is discontinuous with the midsagittal crest of the frontal (**Fig. S1.14**). Another peculiar feature of the parietal of *M. sanderi* QMF31166 is the presence of two lateral crests (**Fig. S1.14**; see also figure 2 in Willis, 2001). The lateral crests on the parietal of *M. sanderi* are situated close to the medial margins of the supratemporal fenestrae and are curved, which contrasts with the straight crests located over the parietal of *Trilophosuchus*. Additionally, the lateral crests of *M. sanderi* cross over to the medial processes of the squamosals, but do not extend anteriorly over the postorbitals and frontal. Therefore, some members of Mekosuchinae (i.e., *Trilophosuchus* and *M. sanderi*) possess a three-crested cranial table, where two different ornamental morphotypes can be recognized: a cranial table ornamented by three continuous longitudinal crests (*Trilophosuchus* spp.), and a cranial table ornamented by discontinuous and (a pair of) non-longitudinal crests (*M. sanderi*).

In *T. rackhami*, the ventral lamina of the jugal is bent ventrolaterally and bears ornamentation like on the lateral surface of the element (**Fig. S1.7**). This morphology is evident in other crocodylians, including some mekosuchines like *A. clarkae*, *M. sanderi* and *Quinkana* (**Figs. S1.8–S1.10**). Unlike *P. vincenti*, the jugal of *T. rackhami* does not possess a notable concavity on its ventromedial surface (see Ristevski *et al.*, 2020a and the 3D PDF in Ristevski *et al.*, 2020b). Another feature of the jugal in *T. rackhami* that merits commenting on is the relation between the ascending process (or, the ventral portion of the postorbital bar) and the lateral surface of the jugal. In *T. rackhami* (most readily observable from the STL files of the jugals and/or the 3D PDF, provided as supplementary material), *M. inexpectatus* (see figures 1 and 2 in Buffetaut, 1983) and *M. sanderi* (see figure S1.11C in Ristevski *et al.*, 2020b), the base of the postorbital bar is flush with

the lateral surface of the jugal. On the other extreme, the base of the postorbital bar is medially inset in *Baru*, *Kambara* and *P. vincenti* where the postorbital bar is separated from the lateral surface of the jugal by a deep trough (Buchanan, 2009; Yates, 2017; Ristevski *et al.*, 2020a). A somewhat intermediate condition occurs in *A. clarkae* (**Fig. S1.8B**) and *Quinkana* (*Q. timara* NTM P8697-2, **Fig. S1.9B**; *Quinkana* sp. indet. QMF1152, **Fig. S1.10**), where the base of the postorbital bar is mildly inset medially.

In *T. rackhami*, the occipital lamina of the supraoccipital is exemplified by a unique combination of anatomical features. No other known mekosuchine has a flat occipital lamina of the supraoccipital that is devoid of a nuchal crest and covered by a superficial wrinkle-like texture. Most crocodylians (including some mekosuchines; e.g., *Kambara* spp., *Q. timara*) have a nuchal crest along the midline that is usually accompanied by a pair of concavities that flank the crest laterally. Such morphology is also present and consistent throughout ontogeny among the examined *Crocodylus johnstoni* and *C. porosus* specimens available for our assessment. Some crocodylians, like the gavialoid *H. camfieldensis* and the mekosuchine *P. vincenti*, possess a nuchal crest but lack the sub-circular concavities lateral to it. While not identical to *T. rackhami*, the mekosuchines *A. clarkae* and *M. sanderi* have the most comparable morphology of the occipital lamina of the supraoccipital. The holotype specimen of *A. clarkae* (QMF16788) also has a flat occipital lamina of the supraoccipital that lacks a nuchal crest (**Fig. S3.4C**). However, unlike *T. rackhami* the occipital lamina of the supraoccipital in *A. clarkae* has a smooth texture. Like *T. rackhami* and *A. clarkae*, the occipital lamina of the supraoccipital in *M. sanderi* is also flat and without a nuchal crest or concavities. Uniquely, the occipital lamina in *M. sanderi* is heavily ornamented with conspicuous sub-circular pits akin to the cranial table (**Fig. S1.6**).

The quadrate of *T. rackhami* displays a morphology that is akin to several other mekosuchines, such as *Mekosuchus*. Two species of *Mekosuchus* – *M. inexpectatus* and *M. sanderi* – have sufficiently preserved quadrates suitable for comparisons. Like *T. rackhami*, both *M. inexpectatus* and *M. sanderi* have a condylar surface of the quadrate that has a dorsal peak situated

closer to the medial hemicondyle than to the lateral, and the dorsal and ventral margins of the condylar surface are sub-parallel (**Figs. 22B**, **23B**, **S1.11–S1.13**; see also figure 45C in Appendix 2 of Rio & Mannion, 2021). In contrast to *Trilophosuchus* and *Mekosuchus*, the condylar surface of the quadrate in *P. vincenti* lacks a prominent dorsal peak and the dorsal and ventral margins are concave (figure 28A in Ristevski *et al.*, 2020a). As in *T. rackhami*, the body of the quadrate in species of *Mekosuchus* is also relatively short, with the anteroposterior length of the body from the paroccipital process to the condylar surface being less than the width at the quadrate condyles. Indeed, this feature of the quadrate occurs in most, but not all, crocodylians including all other formally described mekosuchines that have known quadrates (e.g., *B. wickeni*, *Kambara* spp., *P. vincenti*). An exception among currently described mekosuchines is *A. clarkae*, which possesses a quadrate body that is longer than the width at the quadrate condyles (see figure 48B in Appendix 2 of Rio & Mannion, 2021).

In many mekosuchines, where known, the pterygoid process of the quadrate has exposure in occipital view, ventrolateral to the otoccipital. Such is the condition in *Baru*, *Kambara*, *P. vincenti*, *Q. timara*, and even in the Australian gavialoids *G. maunala* and *H. camfieldensis* (Megirian *et al.*, 1991; Salisbury & Willis, 1996; Buchanan, 2009; Yates, 2017; Ristevski *et al.* 2020a, 2021). Among mekosuchines, the exposure of the quadrate's pterygoid process in occipital view is especially pronounced in *B. wickeni* and *P. vincenti* (see figure 10 in Yates, 2017 and figures 24A and 24B in Ristevski *et al.*, 2020a), whereas it is exposed to a comparatively lesser degree in *Kambara* and *Q. timara* (QMF29662, QMF29663, and NMV P179632). In *T. rackhami*, the pterygoid process of the quadrate is barely visible in occipital view (**Figs. 1E** and **2E**), which is similar to *M. sanderi* (**Figs. S1.6A** and **S1.13A**).

INSTITUTIONAL ABBREVIATIONS

CMC, Chinchilla Museum Collection, Chinchilla, Queensland, Australia

NMV, Museum Victoria, Melbourne, Victoria, Australia

NTM, Museum and Art Gallery of the Northern Territory, Darwin and Alice Springs, Northern Territory, Australia (P, palaeontology)

QM, Queensland Museum, Brisbane, Queensland, Australia (F, fossil)

REFERENCES

Aguilera, O. A., Riff, D., & Bocquentin-Villanueva, J. (2006). A new giant *Purussaurus* (Crocodyliformes, Alligatoridae) from the upper Miocene Urumaco formation, Venezuela. *Journal of Systematic Palaeontology*, *4*(3), 221–232.

Balouet, J. C., & Buffetaut, E. (1987). *Mekosuchus inexpectatus*, n. g., n. sp., Crocodilien nouveau de l'Holocène de Nouvelle Calédonie. *Comptes rendus de l'Académie des sciences. Série 2, Mécanique, Physique, Chimie, Sciences de l'univers, Sciences de la Terre*, *304*(14), 853–856.

Barbosa-Rodrigues, B. (1892). Les Reptiles fossiles de la vallée de l'Amazone. *Vellosia*, *2*, 41–46.

Brochu, C. A. (2004). A new Late Cretaceous gavialoid crocodylian from eastern North America and the phylogenetic relationships of thoracosaurs. *Journal of Vertebrate Paleontology*, *24*(3), 610–633.

Buchanan, L. A. (2009). *Kambara taraina* sp. nov. (Crocodylia, Crocodyloidea), a new Eocene mekosuchine from Queensland, Australia, and a revision of the genus. *Journal of Vertebrate Paleontology*, *29*(2), 473–486.

Buffetaut, E. (1983). Sur la persistance tardive d'un Crocodilien archaïque dans le Pléistocène de l'Ile des Pins (Nouvelle-Calédonie) et sa signification biogéographique. *Compte rendus des*

séances de l'Académie des sciences. Série 2, Mécanique-physique, chimie, sciences de l'univers, sciences de la terre, *297*(1), 89–92.

Daudin, F. M. (1802). *Histoire Naturelle, Générale et Particulière des Reptiles; ouvrage faisant suit à l'Histoire naturell générale et particulière, composée par Leclerc de Buffon; et rédigee par C. S. Sonnini, membre de plusieurs sociétés savantes*. Vol. 2. F. Dufart, Paris [1802], 432 pp.

Ginsburg, L., & Buffetaut, E. (1978). *Euthecodon arambourgi* n. sp., et l'évolution du genre *Euthecodon*, Crocodilien du Néogène d'Afrique. *Géologie Méditerranéenne*, *5*(2), 291–301.

Holt, T. R., Salisbury, S. W., Worthy, T., Sand, C., & Anderson, A. (2007). New material of *Mekosuchus inexpectatus* (Crocodylia: Mekosuchinae) from the Quaternary of New Caledonia. Unpublished conference paper. Available on ResearchGate.

Kraus, R. (1998). The cranium of *Piscogavialis jugaliperforatus* n. gen., n. sp. (Gavialidae, Crocodylia) from the Miocene of Peru. *Paläontologische Zeitschrift*, *72*(3–4), 389–406.

Krefft, G. (1873). Remarks on Australian crocodiles and description of a new species. *Proceedings of the Zoological Society of London*, *1873*, 334–335.

Langston, W. (1965). Fossil crocodilians from Colombia and the Cenozoic history of the Crocodilia in South America. *University of California Publications in Geological Science*, *52*, 1–157.

Mead, J. I., Steadman, D. W., Bedford, S. H., Bell, C. J., & Spriggs, M. (2002). New extinct mekosuchine crocodile from Vanuatu, South Pacific. *Copeia*, *2002*(3), 632–641.

Megirian, D. (1994). A new species of *Quinkana* Molnar (Eusuchia: Crocodylidae) from the Miocene Camfield Beds of northern Australia. *The Beagle, Records of the Museums and Art Galleries of Northern Territory*, *11*, 145–166.

Megirian, D., Murray, P. F., & Willis, P. (1991). A new crocodile of the gavial ectomorph morphology from the Miocene of northern Australia. *The Beagle, Records of the Northern Territory Museum of Arts and Sciences*, *8*(1), 135–158.

Molnar, R. E. (1981). Pleistocene ziphodont crocodilians of Queensland. *Records of the Australian Museum*, *33*(19), 803–834.

Owen, R. (1878). On the fossils called "granicones"; being a contribution to the histology of the exo-skeleton in" Reptilia". *Journal of the Royal Microscopical Society*, *1*, 233–236.

Rio, J. P., & Mannion, P. D. (2021). Phylogenetic analysis of a new morphological dataset elucidates the evolutionary history of Crocodylia and resolves the long-standing gharial problem. *PeerJ*, *9*, e12094.

Ristevski, J., Yates, A. M., Price, G. J., Molnar, R. E., Weisbecker, V., & Salisbury, S. W. (2020a). Australia's prehistoric 'swamp king': revision of the Plio-Pleistocene crocodylian genus *Pallimnarchus* de Vis, 1886. *PeerJ*, *8*, e10466.

Ristevski, J., Yates, A. M., Price, G. J., Molnar, R. E., Weisbecker, V., & Salisbury, S. W. (2020b). Data from: Australia's prehistoric 'swamp king': revision of the Plio-Pleistocene crocodylian genus *Pallimnarchus* de Vis, 1886. *Dryad, Dataset*. DOI: 10.5061/dryad.8kprr4xkq.

Ristevski, J., Price, G. J., Weisbecker, V., & Salisbury, S. W. (2021). First record of a tomistomine crocodylian from Australia. *Scientific Reports*, *11*, 12158.

Salisbury, S. W., & Willis, P. M. A. (1996). A new crocodylian from the Early Eocene of south-eastern Queensland and a preliminary investigation of the phylogenetic relationships of crocodyloids. *Alcheringa: An Australasian Journal of Palaeontology*, *20*(3), 179–226.

Schneider, J. G. (1801). *Historiae amphibiorum naturalis et literariae. Fasciculus secundus continens Crocodilos, Scincos, Chamaesauras, Boas. Pseudoboas, Elapes, Angues, Amphisbaenas et Caecilias*. Friedrich Frommann, Jena, 365 pp.

Willis, P. M. A. (1993). *Trilophosuchus rackhami* gen. et sp. nov., a new crocodilian from the early Miocene limestones of Riversleigh, northwestern Queensland. *Journal of Vertebrate Paleontology*, *13*(1), 90–98.

Willis, P. M. A. (1997). New crocodilians from the late Oligocene White Hunter Site, Riversleigh, northwestern Queensland. *Memoirs of the Queensland Museum*, *41*(2), 423–438.

Willis, P. M. A. (2001). New crocodilian material from the Miocene of Riversleigh (northwestern Queensland, Australia). Pp. 64–74 in G. Grigg, F. Seebacher & C. E. Franklin (eds.) *Crocodilian Biology and Evolution*, Surrey Beatty & Sons, Sydney, Australia.

Willis, P. M. A. & Mackness, B. S. (1996). *Quinkana babarra*, a new species of ziphodont mekosuchine crocodile from the early Pliocene Bluff Downs local fauna, northern Australia with a revision of the genus. *Proceedings of the Linnean Society of New South Wales*, *116*, 143–151.

Willis, P. M. A., & Molnar, R. E. (1991). A new Middle Tertiary crocodile from Lake Palankarinna, South Australia. *Records of the South Australian Museum*, *25*(1), 39–55.

Willis, P. M. A., Molnar, R. E., & Scanlon, J. D. (1993). An early Eocene crocodilian from Murgon, Southeastern Queensland. *Kaupia*, *3*, 27–33.

Willis, P., Murray, P., & Megirian, D. (1990). *Baru darrowi* gen. et sp. nov., a large broad-snouted crocodyline (Eusuchia: Crocodilidae) from mid-Tertiary freshwater limestones in northern Australia. *Memoirs of the Queensland Museum*, *29*(2), 521–540.

Yates, A. M. (2017). The biochronology and palaeobiogeography of *Baru* (Crocodylia: Mekosuchinae) based on new specimens from the Northern Territory and Queensland, Australia. *PeerJ*, *5*, e3458.

Yates, A. M., & Pledge, N. S. (2016). A Pliocene mekosuchine (Eusuchia: Crocodilia) from the Lake Eyre Basin of South Australia. *Journal of Vertebrate Paleontology*, *37*(1), e1244540.
