# Supplementary material for: Cranial anatomy of the mekosuchine crocodylian Trilophosuchus rackhami Willis, 1993
Source: Anat Rec (Hoboken). 2022 Aug 29;306(2):239–97. doi: 10.1002/ar.25050 (PMC10086963; doi:10.1002/ar.25050)
Supplement: Supplementary file 2 — Appendix S2 Document S2 [file AR-306-239-s003.pdf]

SUPPLEMENTARY MATERIAL FOR

# CRANIAL ANATOMY OF THE MEKOSUCHINE CROCODYLIAN *TRILOPHOSUCHUS RACKHAMI* WILLIS, 1993

## SUPPLEMENTAL DOCUMENT S2: REFERRED SPECIMENS


by JORGO RISTEVSKI[1*], VERA WEISBECKER[1,2], JOHN D. SCANLON[3], GILBERT J. PRICE[4], and STEVEN W. SALISBURY[1]

[1]School of Biological Sciences, The University of Queensland, Brisbane, 4072, Queensland, Australia

[2]College of Science and Engineering, Flinders University, Bedford Park 5042, South Australia, Australia

[3]Phoenix Environmental Sciences, Osborne Park 6017, Western Australia, Australia

[4]School of Earth and Environmental Sciences, The University of Queensland, Brisbane, 4072, Queensland, Australia

***Corresponding author:**

Jorgo Ristevski[1]

School of Biological Sciences, Goddard Building (Building 8), The University of Queensland, Brisbane 4072, Queensland, Australia

**Email address:** j.ristevski@uq.net.au

# CONTENTS OF THE SUPPLEMENTARY DOCUMENT

This supplementary document to the study titled "Cranial anatomy of the mekosuchine crocodylian *Trilophosuchus rackhami* Willis, 1993" includes a detailed figure (**Fig. S2.1**) of the two specimens (QMF16857, an isolated frontal, and QMF16858, an isolated right postorbital) referred to *Trilophosuchus rackhami* Willis, 1993. Also, in **Fig. S2.2** is a photograph of specimen QMF16859 (fragmentary basioccipital) which in this study is regarded as Mekosuchinae gen. et sp. indet. Lastly, in **Fig. S2.3** is figured specimen QMF60374, an isolated parietal that is referred to as *Trilophosuchus* sp. At present, the specific assignment for QMF60374 is uncertain.

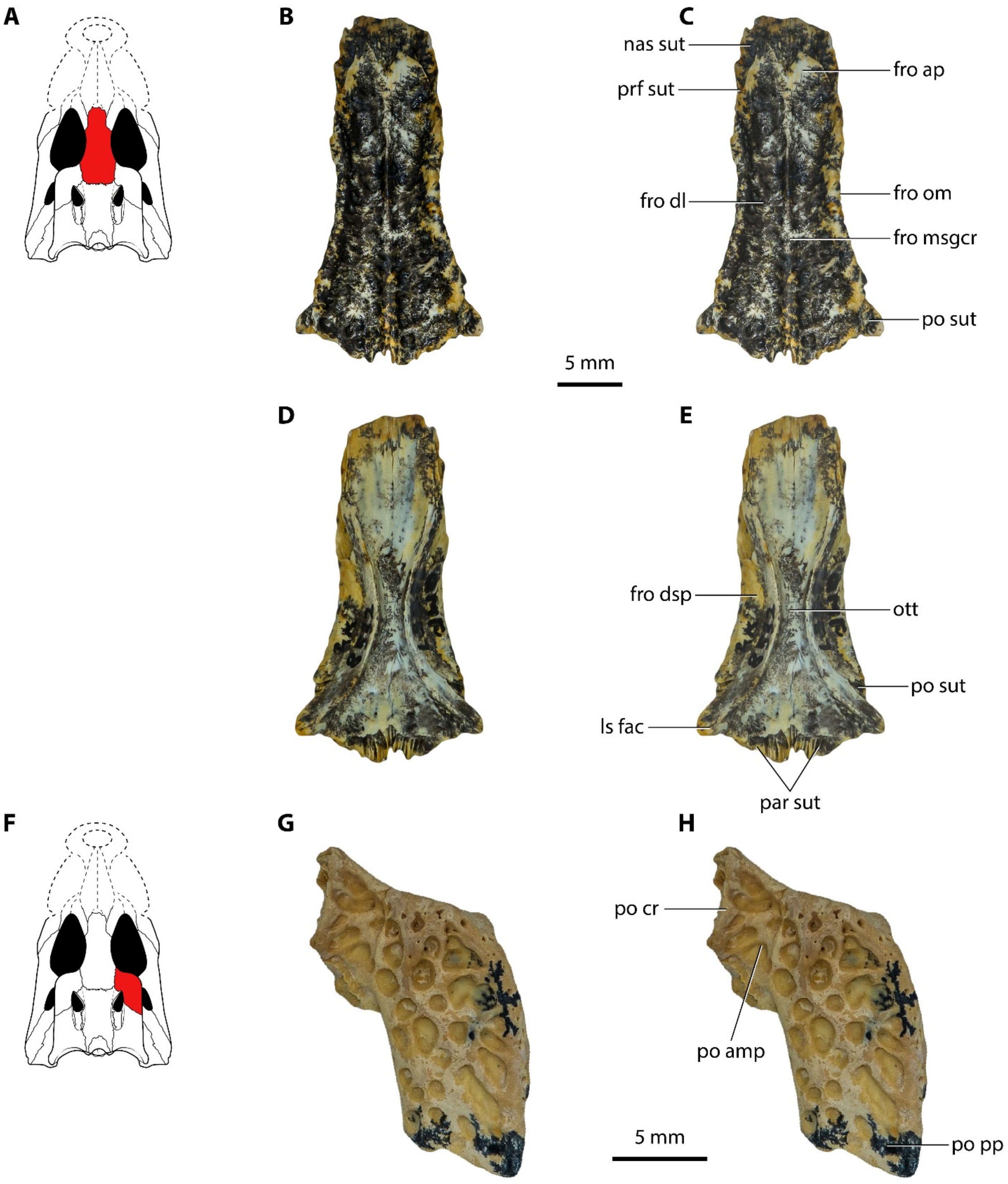
A
B
C
nas sut
fro ap
prf sut
fro om
fro dl
fro msgcr
po sut
5 mm
D
E
fro dsp
ott
po sut
ls fac
par sut
F
G
H
po cr
po amp
5 mm
po pp

**Figure S2.1 (on previous page) *Trilophosuchus rackhami* Willis, 1993, referred specimens.** (A) Interpretative drawing of a *Trilophosuchus rackhami* skull (based on QMF16856, holotype) with the frontal highlighted in red. QMF16857, frontal in dorsal view (B) non-annotated, and (C) annotated photograph. QMF16857, frontal in ventral view (D) non-annotated, and (E) annotated photograph. (F) Interpretative drawing of a *Trilophosuchus rackhami* skull (based on QMF16856, holotype) with the right postorbital highlighted in red. QMF16858, right postorbital in dorsal view (G) non-annotated, and (H) annotated photograph. The dashed lines in the interpretative drawings in (A) and (F) indicate the hypothetical outlines and sutures on the missing portions of the rostrum. For a high-resolution version of this figure, see the PDF file of **Figure S2.1** provided as a supplementary file. Abbreviations: **fro ap**, anterior process of the frontal; **fro dl**, dorsal lamina of the frontal; **fro dsp**, descending process of the frontal; **fro msgcr**, midsagittal crest on the frontal; **fro om**, orbital margin of the frontal; **ls fac**, laterosphenoid facet; **nas sut**, sutural surface for articulation with the nasal; **ott**, olfactory tract trough; **par sut**, sutural surface for articulation with the parietal; **po amp**, anteromedial process of the postorbital; **po cr**, postorbital crest; **po pp**, posterior process of the postorbital; **po sut**, sutural surface for articulation with the postorbital; **prf sut**, sutural surface for articulation with the prefrontal.

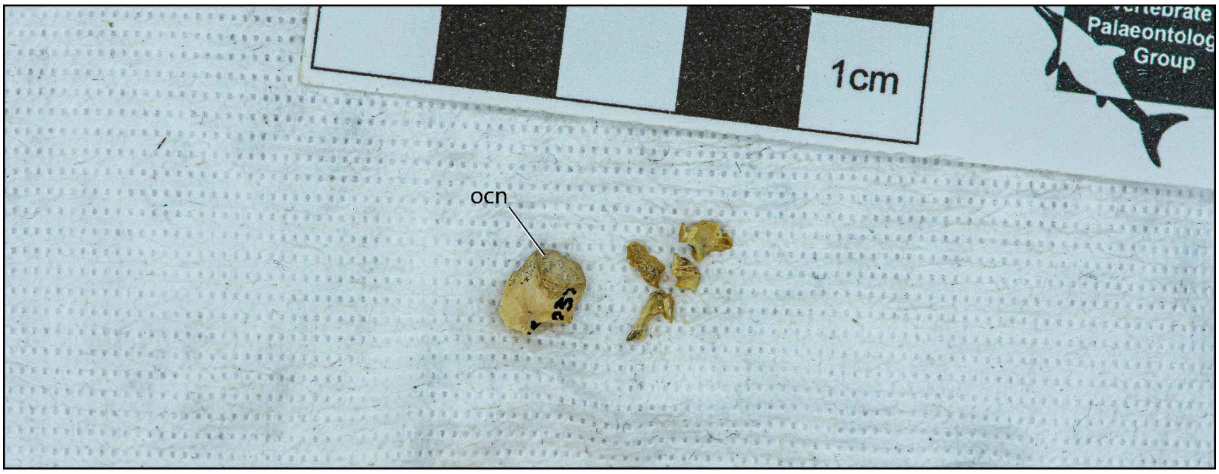


**Figure S2.2 Mekosuchinae gen. et sp. indet., QMF16859, fragmentary basioccipital.** According to its museum record, specimen QMF16859 was submitted to the Queensland Museum collection in this fractured state. The largest preserved piece is the basioccipital plate and occipital condyle, pictured in posterior view. This photograph was taken on the 23rd of January 2020. A photograph of QMF16859 in a more complete condition can be found in figure 8.5 of Willis (1995). For a high-resolution version of this figure, see the PDF file of **Figure S2.2** provided as a supplementary file. Abbreviation: **ocn**, occipital condyle.

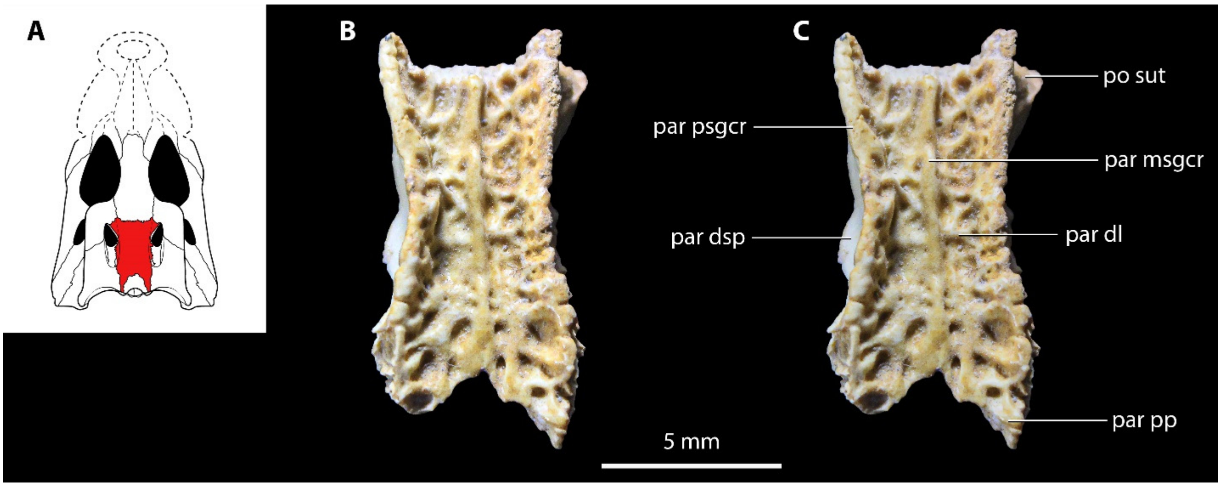


**Figure S2.3 *Trilophosuchus* sp., QMF60374, isolated parietal.** (A) Interpretative drawing of a *Trilophosuchus rackhami* skull (based on QMF16856, holotype) with the parietal highlighted in red. QMF60374, parietal in dorsal view (B) non-annotated, and (C) annotated photograph. The dashed lines in the interpretative drawing in (A) indicate the hypothetical outlines and sutures on the missing portions of the rostrum. For a high-resolution version of this figure, see the PDF file of **Figure S2.3** provided as a supplementary file. Abbreviations: **par dl**, dorsal lamina of the parietal; **par dsp**, descending process of the parietal; **par msgcr**, midsagittal crest on the parietal; **par pp**, posterior process of the parietal; **par psgcr**, parasagittal crest on the parietal; **po sut**, sutural surface for articulation with the postorbital.

# INSTITUTIONAL ABBREVIATION

**QM**, Queensland Museum, Brisbane, Queensland, Australia (F, fossil)

# REFERENCES

Willis, P. M. A. (1993). *Trilophosuchus rackhami* gen. et sp. nov., a new crocodilian from the early Miocene limestones of Riversleigh, northwestern Queensland. *Journal of Vertebrate Paleontology*, *13*(1), 90–98.

Willis, P. M. A. (1995). *Phylogenetic Systematics of Australian Crocodilians*. Unpublished PhD thesis, University of New South Wales, Sydney, 276 pp.
