# Supplementary material for: Cranial anatomy of the mekosuchine crocodylian Trilophosuchus rackhami Willis, 1993
Source: Anat Rec (Hoboken). 2022 Aug 29;306(2):239–97. doi: 10.1002/ar.25050 (PMC10086963; doi:10.1002/ar.25050)
Supplement: Supplementary file 3 — Appendix S3 Document S3 [file AR-306-239-s002.pdf]

SUPPLEMENTARY MATERIAL FOR

CRANIAL ANATOMY OF THE MEKOSUCHINE CROCODYLIAN *TRILOPHOSUCHUS RACKHAMI* WILLIS, 1993

SUPPLEMENTAL DOCUMENT S3: ADDITIONAL INFORMATION ON THE PHYLOGENETIC DATASET AND PHYLOGENETIC RESULTS

by JORGO RISTEVSKI[1*], VERA WEISBECKER[1,2], JOHN D. SCANLON[3], GILBERT J. PRICE[4], and STEVEN W. SALISBURY[1]

[1]School of Biological Sciences, The University of Queensland, Brisbane, 4072, Queensland, Australia

[2]College of Science and Engineering, Flinders University, Bedford Park 5042, South Australia, Australia

[3]Phoenix Environmental Sciences, Osborne Park 6017, Western Australia, Australia

[4]School of Earth and Environmental Sciences, The University of Queensland, Brisbane, 4072, Queensland, Australia

***Corresponding author:**

Jorgo Ristevski[1]

School of Biological Sciences, Goddard Building (Building 8), The University of Queensland, Brisbane 4072, Queensland, Australia

Email address: j.ristevski@uq.net.au

TABLE OF CONTENTS

Taxon list **2**

Links to the examined digital specimens 22

Character list and taxon matrix – modifications and updates **25**

Modification to the scores of some Australian crocodylians 27

Harpacochampsa camfieldensis 27

Modifications to the scores for *Trilophosuchus rackhami* from older matrices 30

Modifications to the scores for *Trilophosuchus rackhami* for Rio & Mannion (2021) 34

Morphological character list **42**

Explanations for scoring the new characters **77**

Results from the phylogenetic analyses **81**

Scored characters for the Australian crocodyliform operational taxonomic units **92**

Institutional abbreviations **93**

References **94**

TAXON LIST

Below is a list (in alphabetical order) of all operational taxonomic units (OTUs) included in the taxon matrix. The sources, both from the literature as well as examined specimens, used for comparative anatomy (see the main text of "Cranial anatomy of the mekosuchine crocodylian *Trilophosuchus rackhami* Willis, 1993") and scoring the new morphological characters are also provided. An asterisk ‘*’ designates a type specimen. A ‘c’ stands for cast, while a ‘*c’ stands for cast of the type specimen. A ‘p’ indicates that high-quality photographs were used for assessment. Type specimens examined from photographs are indicated with a ‘*p’. A ‘d’ stands for digital model whilst ‘*d’ stands for digital model of the type specimen, available online. Links to the examined digital models can be found below.

- *Acynodon adriaticus* Delfino *et al.*, 2008b

  Literature source: Delfino *et al.* (2008b)

- *Acynodon iberoccitanus* Buscalioni *et al.*, 1997

  Literature sources: Buscalioni *et al.* (1997); Martin (2007); Ősi (2014)

- *Agaresuchus fontisensis* Narváez *et al.*, 2016

  Literature sources: Narváez *et al.* (2016); Serrano-Martínez *et al.* (2021)

- *Agaresuchus subjuniperus* (Puértolas-Pascual *et al.*, 2014)

  Literature sources: Puértolas-Pascual *et al.* (2014); Narváez *et al.* (2016); Mateus *et al.* (2019)

- *Alligator mcgrewi* Schmidt, 1941

  Literature sources: Brochu (1999, 2004b)

- *Alligator mefferdi* Mook, 1946

  Literature sources: Mook (1946); Brochu (1999)

- *Alligator mississippiensis* (Daudin, 1802)

  Examined specimens: QMJ4850, unregistered specimen at UQ, UF35129[d]

  Literature sources: Mook (1921c); Wermuth (1953); Iordansky (1973); Brochu (1999, 2004b); Salisbury (2001); Gold (2011); de Iuliis & Pulerà (2011); Dufeau & Witmer (2015); Drumheller *et al.* (2016); Klein (2016); Porter *et al.* (2016); Sookias (2019, 2020)

- *Alligator olseni* White, 1942

  Literature sources: White (1942); Brochu (1999)

- *Alligator prenasalis* (Loomis, 1904)

  Literature sources: Mook (1932); Brochu (1999, 2004b)

- *Alligator sinensis* Fauvel, 1879

  Examined specimen: UF-H-105540[d]

  Literature sources: Mook (1923b); Wermuth (1953); Iordansky (1973); Cong *et al.* (1998); Brochu (1999); Drumheller *et al.* (2016); Sookias (2019, 2020)

- *Alligator thomsoni* Mook, 1923a

  Literature sources: Mook (1923a); Brochu (1999)

- *Allodaposuchus precedens* Nopcsa, 1928

  Literature sources: Delfino *et al.* (2008a); Mateus *et al.* (2019); Narváez *et al.* (2020)

- *Allognathosuchus polyodon* Cope, 1873

  Literature sources: Mook (1961); Brochu (1999, 2004b)

- *Allognathosuchus wartheni* Case, 1925

  Literature sources: Brochu (1999, 2004b)

- *Anteophthalmosuchus epikrator* Ristevski *et al.*, 2018

  Examined specimens: IRSNB R47, IWCMS 2001.446*, IWCMS 2005.127

  Literature sources: Salisbury (2001); Martin *et al.* (2016); Ristevski *et al.* (2018)

- *Arambourgia gaudryi* (de Stefano, 1905)

  Literature sources: Brochu (1999, 2004b)

- *Arenysuchus gascabadiolorum* Puértolas-Pascual *et al.*, 2011

  Literature sources: Puértolas-Pascual *et al.* (2011); Mateus *et al.* (2019)

- *Asiatosuchus germanicus* Berg, 1966

  Literature source: Berg (1966)

- *Australosuchus clarkae* Willis & Molnar, 1991

  Examined specimens: QMF16788*, QMF17433, QMF17983, QMF17984, QMF17985, QMF17986, QMF18102, QMF18151, QMF18152, SAM P23985[c, p], SAM P30160[p], SAM P30162[p], SAM P33761[p], SAM P33768[p], SAM P33942[c, p], SAM P36583[p], SAM P42788[p], SAM P42789[p]

  Literature source: Willis & Molnar (1991)

- *Baru darrowi* Willis *et al.*, 1990

  Examined specimens: NTM P8695-8*, NTM P87115-15

Literature sources: Willis *et al.* (1990); Yates (2017)

- *Baru wickeni* Willis, 1997

  Examined specimens: NTM P902-4, NTM P911, NTM P943-4 (P902-, P91164-), NTM P8681-14, NTM P8738-1, NTM P8778-2, NTM P8778-4, NTM P8778-5, NTM P91164-10, NTM P91171-1, QMF16822*, QMF16823, QMF16824, QMF31070, QMF31071, QMF31072, QMF31073, QMF31074

  Literature sources: Willis (1997); Yates (2017)

- *Bernissartia fagesii* Dollo, 1883

  Examined specimen: IRSNB R46*p

  Literature sources: Buffetaut (1975); Norell & Clark (1990); Salisbury (2001); Ősi (2014); Sookias (2019, 2020); Martin *et al.* (2020)

- *Borealosuchus acutidentatus* (Sternberg, 1932)

  Literature source: Brochu (1997a)

- *Borealosuchus formidabilis* (Erickson, 1976)

  Literature sources: Erickson (1976); Brochu (1997a); Brochu *et al.* (2012)

- *Borealosuchus sternbergii* (Gilmore, 1910)

  Literature sources: Gilmore (1910); Brochu (1997a); Brochu *et al.* (2012)

- *Borealosuchus threeensis* Brochu *et al.*, 2012

  Literature source: Brochu *et al.* (2012)

- *Borealosuchus wilsoni* (Mook, 1959)

  Literature sources: Mook (1959); Brochu (1997a); Brochu *et al.* (2012)

- *Bottosaurus harlani* (Meyer, 1832)

    Literature source: Cossette & Brochu (2018)

- *Boverisuchus magnifrons* Kuhn, 1938

    Literature sources: Rossmann (2000); Brochu (2012)

- *Boverisuchus vorax* (Troxell, 1925)

    Literature sources: Langston (1975); Busbey (1977); Rossmann (2000); Brochu (2012)

- *Brachychampsa montana* Gilmore, 1911

    Literature sources: Gilmore (1911); Norell *et al.* (1994); Brochu (1999, 2004b); Sullivan & Lucas (2003)

- *Brachychampsa sealeyi* Williamson, 1996

    Literature source: Williamson (1996)

- *Brachyuranochampsa eversolei* Zangerl, 1944

    Literature source: Zangerl (1944)

- *Brochuchus pigotti* (Tchernov & Van Couvering, 1978)

    Literature sources: Tchernov & Van Couvering (1978); Conrad *et al.* (2013)

- *Caiman crocodilus* (Linnaeus, 1758)

    Examined specimens: FMNH 73711[d], QMJ2094, QMJ53058

    Literature sources: Wermuth (1953); Iordansky (1973); Brochu (1999); Gold (2011); Sookias (2019, 2020)

- *Caiman latirostris* (Daudin, 1802)

  Literature sources: Wermuth (1953); Brochu (1999); Bona & Desojo (2011); Ősi (2014); Foth *et al.* (2018); Sookias (2019, 2020)

- *Caiman lutescens* (Rovereto, 1912)

  Literature sources: Langston (1965); Brochu (1999)

- *Caiman yacare* (Daudin, 1802)

  Literature sources: Wermuth (1953); Brochu (1999); Mateus *et al.* (2019); Sookias (2019, 2020)

- *Centenariosuchus gilmorei* Hastings *et al.*, 2013

  Literature source: Hastings *et al.* (2013)

- *Ceratosuchus burdoshi* Schmidt, 1938

  Literature sources: Schmidt (1938); Bartels (1984); Brochu (1999)

- '*Crocodylus*' *acer* Cope, 1882

  Literature sources: Cope (1882); Mook (1921b); Brochu (1997c)

- '*Crocodylus*' *affinis* Marsh, 1871

  Literature sources: Mook (1921a); Norell & Storrs (1989)

- '*Crocodylus*' *depressifrons* de Blainville, 1855

  Literature source: Delfino & Smith (2009)

- '*Crocodylus*' *gariepensis* Pickford, 2003

  Literature source: Pickford (2003)

- '*Crocodylus*' *megarhinus* Andrews, 1905

  Literature source: Mook, 1927

- *Crocodylus acutus* Cuvier, 1807

  Examined specimen: UF Herp 115691[d]

  Literature sources: Wermuth (1953); Iordansky (1973); Brochu (2006a)

- *Crocodylus anthropophagus* Brochu *et al.*, 2010

  Literature source: Brochu *et al.* (2010); Azzarà *et al.* (2021)

- *Crocodylus checchiai* Maccagno, 1948

  Literature sources: Hecht (1987); Brochu & Storrs (2012); Delfino *et al.* (2020)

- *Crocodylus intermedius* Graves, 1819

  Literature source: Mook (1921c)

- *Crocodylus johnstoni* (Krefft, 1873)

  Examined specimens: QMJ4280, QMJ22551, QMJ28895, QMJ39230, QMJ40259, QMJ45309, QMJ47916, QMJ58446, QMJ60590, QMJ60591, QMJ60592, QMJ65021, QMJ65022, QMJ65024, QMJ75835, QMJ84415, QMJ85977, QMJ86243, QMJ93435, QMJ94182, UQSSAL J9

  Literature source: Wermuth (1953)

- *Crocodylus mindorensis* Schmidt, 1935

  Literature source: Schmidt (1935)

- *Crocodylus moreletii* Duméril & Bibron, 1851

  Examined specimen: TMM M-4980[d]

Literature sources: Wermuth (1953); Platt *et al.* (2009)

- *Crocodylus niloticus* Laurenti, 1768

  Examined specimen: QMJ58445

  Literature sources: Mook (1921c); Wermuth (1953); Sookias (2019, 2020)

- *Crocodylus novaeguineae* (Schmidt, 1928)

  Examined specimens: QMJ5332, QMJ5664

  Literature sources: Wermuth (1953); Hall & Portier (1994)

- *Crocodylus palaeindicus* Falconer, 1859

  Literature source: Mook (1933)

- *Crocodylus palustris* Lesson, 1831

  Literature source: Wermuth (1953); Iijima (2017)

- *Crocodylus porosus* Schneider, 1801

  Examined specimens: NTM R12638, QMJ5005, QMJ13443, QMJ14478, QMJ22550, QMJ29021, QMJ39231, QMJ39232, QMJ39233, QMJ39283, QMJ39284, QMJ39853, QMJ45308, QMJ47446, QMJ47447, QMJ47448, QMJ47474, QMJ48126, QMJ48127, QMJ52809, QMJ87508, QMJ87509, QMJ93238, QMJ93350, one unregistered specimen at QM, UQSSAL J7, five unregistered specimens at UQ

  Literature sources: Mook (1921c); Wermuth (1953); Richardson *et al.* (2002); Sookias (2019, 2020)

- *Crocodylus raninus* Müller & Schlegel, 1844

  Literature source: Ross (1990)

Remark: The status of *Crocodylus raninus* as a valid species has been controversial (see Das & Charles, 2002; Gratten, 2003; Grigg & Kirshner, 2015)

- *Crocodylus rhombifer* Cuvier, 1807

  Examined specimen: NMB AB50.0171[d]

  Literature sources: Mook (1921c); Wermuth (1953); Morgan & Albury (2013); Morgan *et al.* (1993, 2018); Sookias (2019, 2020)

- *Crocodylus siamensis* Schneider, 1801

  Examined specimen: MZB 2005-0351[d], one unregistered specimen at UQ

  Literature sources: Wermuth (1953); Delfino & De Vos (2010)

- *Crocodylus thorbjarnarsoni* Brochu & Storrs, 2012

  Examined specimen: KNM-ER 1683*[d]

  Literature source: Brochu & Storrs (2012); Brochu (2020)

- *Culebrasuchus mesoamericanus* Hastings *et al.*, 2013

  Literature source: Hastings *et al.* (2013)

- *Deinosuchus riograndensis* (Colbert & Bird, 1954)

  Literature source: Colbert & Bird (1954); Cossette & Brochu (2020)

- *Diplocynodon darwini* (Ludwig, 1877)

  Literature sources: Ludwig (1877); Berg (1966)

- *Diplocynodon deponiae* (Frey *et al.*, 1987)

  Literature sources: Frey *et al.* (1987); Delfino & Smith (2012)

- *Diplocynodon hantoniensis* (Wood, 1846)

  Literature sources: Brochu (1999); Rio *et al.* (2020)

- *Diplocynodon muelleri* (Kälin, 1936)

  Literature source: Piras & Buscalioni, 2006

- *Diplocynodon ratelii* Pomel, 1847

  Literature sources: Brochu (1999); Díaz Aráez *et al.* (2017)

- *Diplocynodon tormis* Buscalioni *et al.*, 1992

  Literature sources: Jiménez Fuentes (1983); Buscalioni *et al.* (1992); Serrano-Martínez *et al.* (2019a)

- *Dollosuchoides densmorei* Brochu, 2007b

  Examined specimen: IRSNB R1748*p

  Literature source: Brochu (2007b)

- *Dongnanosuchus hsui* Shan *et al.*, 2021

  Literature source: Shan *et al.* (2021)

- *Eoalligator chunyii* Young, 1964

  Literature sources: Young (1964), Wang *et al.* (2016)

- *Eocaiman cavernensis* Simpson, 1933

  Literature sources: Simpson (1933); Brochu (1999); Godoy *et al.* (2021)

- *Eogavialis africanum* (Andrews, 1901)

  Literature source: Müller (1927)

- *Eosuchus lerichei* Dollo, 1907

  Literature source: Delfino *et al.* (2005)

- *Eosuchus minor* (Marsh, 1870)

  Literature source: Brochu (2006b)

- *Eothoracosaurus mississippiensis* Brochu, 2004a

  Literature source: Brochu (2004a)

- *Euthecodon arambourgi* Ginsburg & Buffetaut, 1978

  Literature source: Ginsburg & Buffetaut (1978)

- *Gavialis gangeticus* (Gmelin, 1789)

  Literature sources: Mook (1921c); Wermuth (1953); Iordansky (1973); Tarsitano *et al.* (1989); Salisbury (2001); Brochu (2004a); Gold (2011); Pierce *et al.* (2017); Sookias (2019, 2020); Bourke *et al.* (2021)

- *Gavialis lewisi* Lull, 1944

  Literature sources: Lull (1944); Norell & Storrs (1989)

- *Gavialosuchus eggenburgensis* Toula & Kail, 1885

  Literature source: Toula & Kail (1885)

- *Globidentosuchus brachyrostris* Scheyer *et al.*, 2013

  Literature source: Scheyer *et al.* (2013)

- *Gryposuchus colombianus* (Langston, 1965)

  Literature sources: Langston (1965); Langston & Gasparini (1997); Salas-Gismondi *et al.* (2016)

- *Gunggamarandu maunala* Ristevski *et al.*, 2021

  Examined specimen: QMF548* (=QMF14.548, old registry number)

  Literature source: Ristevski *et al.* (2021)

- *Harpacochampsa camfieldensis* Megirian *et al.*, 1991

  Examined specimens: NTM P87106-1*, NTM P87106-5, NTM P87106-6, NTM P87106-19, NTM P87106-20

  Literature source: Megirian *et al.* (1991)

- *Hassiacosuchus haupti* Weitzel, 1935

  Literature sources: Weitzel (1935); Brochu (1999, 2004b); Ősi (2014)

- *Hylaeochampsa vectiana* Owen, 1874

  Literature sources: Clark & Norell (1992); Salisbury & Naish (2011)

- *Iharkutosuchus makadii* Ősi *et al.*, 2007

  Literature sources: Ősi *et al.* (2007); Ősi (2008, 2014); Ősi & Weishampel (2009); Mateus *et al.* (2019)

- *Isisfordia duncani* Salisbury *et al.*, 2006

  Examined specimens: QMF34642, QMF36211*[c], QMF44320

  Literature sources: Salisbury *et al.* (2006); Syme & Salisbury (2018)

- *Jiangxisuchus nankangensis* Li *et al.*, 2019

  Literature source: Li *et al.* (2019)

- *Kalthifrons aurivellensis* Yates & Pledge, 2016

  Examined specimen: SAM P35062*[p]

Literature source: Yates & Pledge (2016)

- *Kambara implexidens* Salisbury & Willis, 1996

  Examined specimens: QMF21116, QMF21118, QMF21131, QMF29662*, QMF29663, QMF29678, QMF29680, QMF29684, QMF29693, QMF29708, QMF29710, QMF29714, QMF30077

  Literature source: Salisbury & Willis (1996)

- *Kambara murgonensis* Willis *et al.*, 1993

  Examined specimens: QMF11625, QMF21117, QMF21119, QMF21120, QMF21122, QMF21123, QMF21124, QMF21125, QMF21126, QMF21127, QMF21128, QMF21129, QMF21130, QMF21134, QMF29665, QMF29666, QMF29667, QMF29688, QMF29669, QMF29683, QMF29689, QMF29691, QMF29692, QMF29694, QMF29696, QMF29718, QMF30220, QMF30221

  Literature sources: Willis *et al.* (1993, 1995)

- *Kambara taraina* Buchanan, 2009

  Literature source: Buchanan (2009)

- *Kentisuchus spenceri* (Buckland, 1836)

  Examined specimens: NHMUK PV R 1753[p], NHMUK PV OR 19633*[p], NHMUK PV OR 37717[p], NHMUK PV OR 38975[p], NHMUK PV OR 38990[p], NHMUK PV OR 38991[p]

  Literature source: Brochu (2007b)

- *Krabisuchus siamogallicus* Martin & Lauprasert, 2010

  Literature source: Martin & Lauprasert (2010)

- *Leidyosuchus canadensis* Lambe, 1907

  Literature sources: Lambe (1907); Brochu (1997a); Wu *et al.* (2001a)

- *Lohuecosuchus mechinorum* Narváez *et al.*, 2015

  Literature source: Narváez *et al.* (2015)

- *Lohuecosuchus megadontos* Narváez *et al.*, 2015

  Literature sources: Narváez *et al.* (2015); Serrano-Martínez *et al.* (2019b)

- *Maomingosuchus petrolica* (Yeh, 1958)

  Literature sources: Li (1975); Shan *et al.* (2017)

- *Mecistops cataphractus* (Cuvier, 1824)

  Examined specimen: TMM M-3529[d]

  Literature sources: Mook (1921c); Wermuth (1953); Sookias (2019, 2020); Kuzmin *et al.* (2021)

- *Megadontosuchus arduini* (de Zigno, 1880)

  Examined specimen: MGPD 1Z*[p]

  Literature source: Piras *et al.* (2007)

- *Mekosuchus sanderi* Willis, 2001

  Examined specimens: QMF31166, QMF31186, QMF31187, QMF31188*

  Literature source: Willis (2001)

- *Mekosuchus whitehunterensis* Willis, 1997

  Examined specimens: QMF31051*, QMF31052, QMF31053, QMF31054, QMF31055

  Literature source: Willis (1997)

- *Melanosuchus niger* (Spix, 1825)

  Examined specimens: UF Herp 53600[d], UF Herp 62641[d]

  Literature sources: Mook (1921c); Wermuth (1953); Brochu (1999); Foth *et al.* (2015); Bona *et al.* (2017); Foth *et al.* (2018); Mateus *et al.* (2019); Sookias (2019, 2020)

- *Mourasuchus atopus* (Langston, 1965)

  Literature sources: Langston (1965, 1966)

- *Navajosuchus mooki* (Simpson, 1930)

  Literature sources: Simpson (1930); Brochu (2004b)

- *Orientalosuchus naduongensis* Massonne *et al.*, 2019

  Literature source: Massonne *et al.* (2019)

- *Orthogenysuchus olseni* Mook, 1924b

  Literature sources: Mook (1924b); Brochu (1999)

- *Osteolaemus osborni* (Schmidt, 1919)

  Literature sources: Mook (1921c); Wermuth (1953); Brochu (2006a); Conrad *et al.* (2013); Mateus *et al.* (2019); Sookias (2019, 2020)

- *Osteolaemus tetraspis* Cope, 1861

  Examined specimen: FMNH 98396[d], UF-Herp-33749[d]

  Literature sources: Mook (1921c); Wermuth (1953); Iordansky (1973); Gold (2011); Montefeltro *et al.* (2016); Sookias (2019, 2020)

- *Pachycheilosuchus trinquei* Rogers, 2003

  Literature source: Rogers (2003)

- *Paleosuchus palpebrosus* Cuvier, 1807

  Examined specimen: RVC-JRH-PP1[d], UF-Herp-87980[d]

  Literature sources: Wermuth (1953); Brochu (1999); Mateus *et al.* (2019); Sookias (2019, 2020)

- *Paleosuchus trigonatus* (Schneider, 1801)

  Examined specimens: NHMUK 1868.10.8.1[p]; USNM: amphibians & reptiles: 300660[d]

  Literature sources: Mook (1921c); Wermuth (1953); Brochu (1999); Mateus *et al.* (2019); Sookias (2019, 2020)

- *Paludirex gracilis* (Willis & Molnar, 1997)

  Examined specimens: QMF17065*, QMF17066

  Literature source: Ristevski *et al.* (2020a)

- *Paludirex vincenti* Ristevski *et al.*, 2020a

  Examined specimens: 'Geoff Vincent's specimen' (CMC2019-010 + QMF59017)*, QMF11626

  Literature source: Ristevski *et al.* (2020a)

- *Paratomistoma courti* Brochu & Gingerich, 2000

  Literature source: Brochu & Gingerich (2000)

- *Penghusuchus pani* Shan *et al*., 2009

  Literature source: Shan *et al.* (2009)

- *Pietraroiasuchus ormezzanoi* Buscalioni *et al*., 2011

  Literature source: Buscalioni *et al.* (2011)

- *Piscogavialis jugaliperforatus* Kraus, 1998

  Literature sources: Kraus (1998); Salas-Gismondi *et al.* (2016)

- *Planocrania datangensis* Li, 1976

  Literature sources: Li (1976); Brochu (2012)

- *Planocrania hengdongensis* Li, 1984

  Literature sources: Li (1984); Brochu (2012)

- *Portugalosuchus azenhae* Mateus *et al*., 2019

  Literature source: Mateus *et al.* (2019)

- *Procaimanoidea kayi* (Mook, 1941b)

  Literature sources: Mook (1941b); Brochu (2004b)

- *Procaimanoidea utahensis* Gilmore, 1946

  Literature sources: Gilmore (1946); Brochu (2004b)

- *Prodiplocynodon langi* Mook, 1941a

  Literature source: Mook (1941a)

- *Protoalligator huiningensis* Young, 1982

  Literature source: Wang *et al.* (2016)

- *Purussaurus neivensis* (Mook, 1941c)

  Literature sources: Langston (1965); Aguilera *et al.* (2006)

- *Quinkana fortirostrum* Molnar, 1981

  Examined specimen: AM F.57844*

  Literature sources: Molnar (1981); Megirian (1994)

- *Quinkana meboldi* Willis, 1997

  Examined specimens: QMF31056*, QMF31057, QMF31058, QMF31059

  Literature source: Willis (1997)

- *Quinkana timara* Megirian, 1994

  Examined specimens: NMV P179632[c], NTM P894-6, NTM P895-16, NTM P895-19*, NTM P895-38, NTM P2775-1, NTM P8691-3, NTM P8697-2; NTM P9464-167, NTM P9464-168, NTM P9464-169, NTM P9464-170, NTM P9464-177[p], NTM P9464-182

  Literature source: Megirian (1994)

- *Rimasuchus lloydi* (Fourtau, 1920)

  Examined specimen: NHMUK PV R 14154[p]

- *Shamosuchus djadochtaensis* Mook, 1924a

  Literature sources: Mook (1924a); Pol *et al.* (2009); Turner (2015)

- *Stangerochampsa mccabei* Wu *et al.*, 1996

  Literature sources: Wu *et al.* (1996); Brochu (2004b)

- *Susisuchus anatoceps* Salisbury *et al.*, 2003

  Literature sources: Salisbury (2001); Salisbury *et al.* (2003); Frey & Salisbury (2007); Figueiredo *et al.* (2011); Leite & Fortier (2018)

- *Thecachampsa antiquus* (Leidy, 1852)

  Literature source: Myrick (2001)

- *Theriosuchus pusillus* Owen, 1878

  Literature sources: Owen (1879); Joffe (1967); Clark (1986); Salisbury (2001, 2002); Tennant *et al.* (2016); Schwarz *et al.* (2017)

- *Thoracosaurus macrorhynchus* (de Blainville, 1835)

  Literature source: Brochu (2004a)

- *Thoracosaurus neocesariensis* (de Kay, 1842)

  Literature source: Carpenter (1983); Brochu (2004a)

- ‘*Tomistoma*’ *cairense* Müller, 1927

  Literature source: Müller (1927)

- *Tomistoma lusitanica* Antunes, 1961

  Literature source: Antunes (1961)

- *Tomistoma schlegelii* (Müller, 1838)

  Examined specimen: TMM M-6342[d]

  Literature sources: Mook (1921c); Wermuth (1953); Iordansky (1973); Tarsitano *et al.* (1989); Gold (2011); Sookias (2019, 2020)

- *Toyotamaphimeia machikanensis* (Kamei et Matsumoto in Kobatake *et al.*, 1965)

  Literature source: Kobayashi *et al.* (2006)

- *Trilophosuchus rackhami* Willis, 1993

  Examined specimens: QMF16856*, QMF16857, QMF16858

  Literature source: Willis (1993)

- *Tsoabichi greenriverensis* Brochu, 2010

  Literature source: Brochu (2010); Walter *et al.* (2022)

- *Voay robustus* (Grandidier & Vaillant, 1872)

  Examined specimens: NHMUK PV OR 2026[p], NHMUK PV R 36684[p], NHMUK PV R 36685[p]

  Literature sources: Brochu (2006a, 2007a); Bickelmann & Klein (2009)

- *Wannaganosuchus brachymanus* Erickson, 1982

  Literature sources: Erickson (1982); Brochu (1999, 2004b)

Links to the examined digital specimens

All below listed digital specimens are available online as of the 23rd of March 2022.

- *Alligator mississippiensis* UF35129:

  https://sketchfab.com/3d-models/alligator-mississippiensis-uf-herp-35129-albert-bd974aa4336c4f33bf14b2e3499970f4

- *Alligator sinensis* UF-H-105540:

  https://sketchfab.com/3d-models/alligator-sinensis-uf-herp-105540-87bec849ccf54be7981bc539d30814c6

- *Caiman crocodilus* FMNH 73711:

  http://www.digimorph.org/specimens/Caiman_crocodilus/

- *Crocodylus acutus* UF Herp 115691:

  https://sketchfab.com/3d-models/american-crocodile-skull-8dc8817a04a4477db22200f53a9c01f7

- *Crocodylus moreletii* TMM M-4980:

  http://www.digimorph.org/specimens/Crocodylus_moreletii/

- *Crocodylus rhombifer* NMB AB50.0171:

  http://www.digimorph.org/specimens/Crocodylus_rhombifer/

- *Crocodylus siamensis* MZB 2005-0351:

  https://sketchfab.com/3d-models/crocodylus-siamensis-skull-126fe65e526041ef8815b0f39186c5af

  https://sketchfab.com/3d-models/crocodylus-siamensis-mandible-740d91fe790d4425bcdd39ed618154bd

- *Crocodylus thorbjarnarsoni* KNM-ER 1683:

  https://africanfossils.org/fauna/knmer-1683

  Remark: As of the 23rd of March 2022, the AfricanFossils.org website (see link above) lists KNM-ER 1683 as a *Rimasuchus lloydi* specimen; this is an error, as the provided digital model is clearly the holotype specimen (skull only, but not the mandibles of KNM-ER 1683) of *Crocodylus thorbjarnarsoni* as assigned by Brochu & Storrs (2012).

- *Mecistops cataphractus* TMM M-3529:

  https://www.morphosource.org/concern/media/000114916?locale=en

- *Melanosuchus niger* UF Herp 53600 and UF Herp 62641:

  https://sketchfab.com/3d-models/black-caiman-skull-uf-herp-53600-351638b3ec5043aea5810759972b0cea

  https://sketchfab.com/3d-models/black-caiman-skull-6e93649695b6482481cdcfa010622439

- *Osteolaemus tetraspis* FMNH 98396:

  https://www.morphosource.org/concern/media/000114918?locale=en

- *Osteolaemus tetraspis* UF-Herp-33749:

  https://www.morphosource.org/concern/media/000395009?locale=en

  https://www.morphosource.org/concern/media/000395025?locale=en

- *Paleosuchus palpebrosus* RVC-JRH-PP1:

  https://sketchfab.com/3d-models/palaeosuchus-palpebrosus-hatchling-crocodylian-13321034846c475e9484f576cdde9e61

  https://osf.io/uad97/

Hutchinson (2020, March 23)

- *Paleosuchus palpebrosus* UF-Herp-87980:

  https://www.morphosource.org/concern/media/000399715?locale=en

- *Paleosuchus trigonatus* USNM: amphibians & reptiles: 300660:

  https://www.morphosource.org/Detail/SpecimenDetail/Show/specimen_id/3737

- *Tomistoma schlegelii* TMM M-6342:

  http://digimorph.org/specimens/tomistoma_schlegelii/

CHARACTER LIST AND TAXON MATRIX – MODIFICATIONS AND UPDATES

The taxon matrix and character list used in this study are updated versions of those previously published by Ristevski *et al.* (2020a, b; 2021). The character list was expanded with the addition of 32 characters (characters 226–257), with three of them being newly formulated (characters 255–257). As this iteration of the matrix is merely an update, details on the previous datasets, such as modifications to character formulations and instructions on how to score certain characters, can be found in Ristevski *et al.* (2020b, 2021). Since Ristevski *et al.* (2021), the taxon matrix was expanded with the inclusion of five additional OTUs, which brings the total number of taxa to 147. The newly incorporated taxa are (in alphabetical order) *Dongnanosuchus hsui*, *Paludirex gracilis*, *Quinkana fortirostrum* (scored exclusively from the holotype, which is currently the only known specimen definitively referable to this species), *Quinkana meboldi*, and *Trilophosuchus rackhami*.

Four characters that were previously used by Ristevski *et al.* (2020b, 2021) have been removed. These are characters 146 of Ristevski *et al.* (2020b, 2021; ch. 163 of Brochu, 1997b), 161 of Ristevski *et al.* (2020b, 2021; ch. 164 of Brochu, 1997b), 225 of Ristevski *et al.* (2020b, 2021; ch. 219 of Lee & Yates, 2018), and 228 of Ristevski *et al.* (2020b, 2021). Recent studies (Kuzmin *et al.*, 2021 and Rio & Mannion, 2021) recommended against the usage of characters 163 and 164 from Brochu (1997b), and character 219 from Lee & Yates (2018). Character 228 from Ristevski *et al.* (2020b, 2021) was removed after a reevaluation of its formulation, which indicated intraspecific variability within some taxa and therefore cast doubt on its utility. Several characters have been replaced and/or modified with versions from other datasets. Thus, characters 83, 157, 169, 179 and 216 were modified from characters 3, 110, 118, 136 and 190 in Rio & Mannion (2021); character 134 was modified from character 67 in Lee & Yates (2018); and, character 148 was modified after character 102 in Delfino *et al.* (2008a). Characters 89, 104, 142 and 160 were modified by either re-

formulating some of their states, reversing their polarities, or by removing a state. Character 84 (scoring the presence or absence of a ghara) used to be scored only for extant taxa. This character's scoring was updated by following the criterion applied by Rio & Mannion (2021) where all extinct taxa that are known from at least two premaxillae of mature individuals are applied a state other than an unknown ('?'). Characters 182 (ch. 185 in Brochu & Storrs, 2012) and 183 (ch. 186 in Brochu & Storrs, 2012) were completely rescored since in older iterations all taxa that were not scored as unknowns had state 0 applied to them, except for *Mecistops cataphractus* which was scored with state 1.

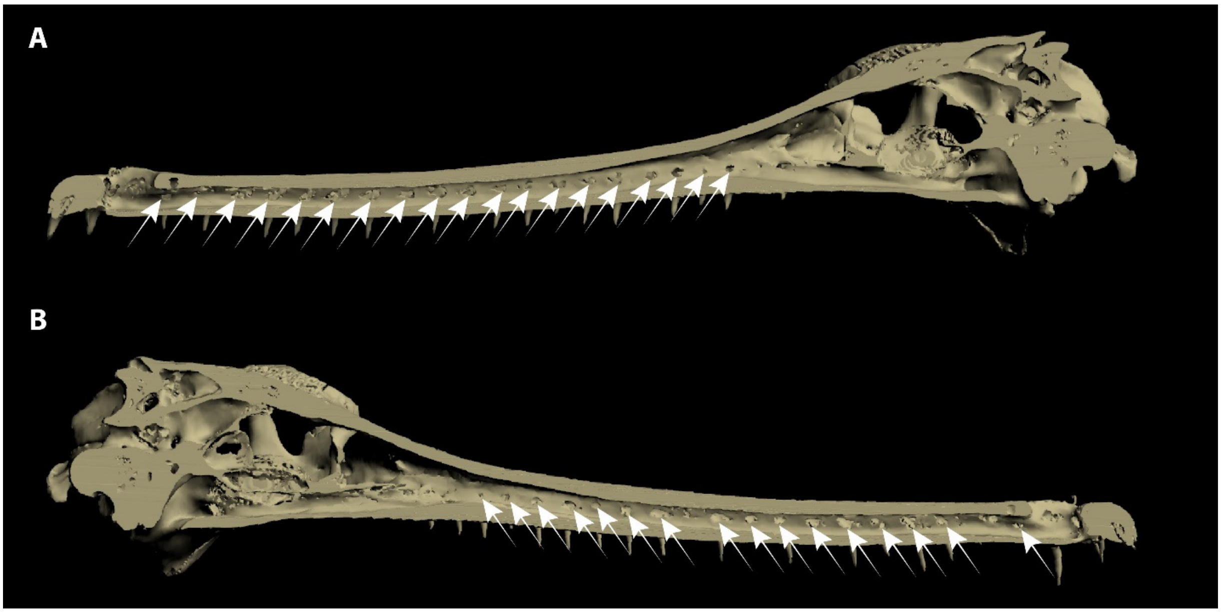

Figure S3.1 ***Gavialis gangeticus*** **(Gmelin, 1789), UMZC R.5792, sagittal section of the skull showing the cecal recesses that adorn the medial surfaces of the maxillae.** Sagittal section exposing the inside of the (A) right, and (B) left side of the skull. The white arrows indicate the linear array of pits (=cecal recesses) within the narial canal on both maxillae. The digital model shown in this figure is from the interactive supplementary 3D PDF document of Pierce *et al*. (2017). The sagittal section of the digital model was achieved by using the Toggle Cross Section function in Adobe Acrobat Pro DC version 21.0 (https://www.adobe.com/au/acrobat/acrobat-pro.html). For a high-resolution version of this figure, see the PDF file of **Figure S3.1** provided as a supplementary file.

Thanks to recently published data, the scores for some OTUs could be revised. The scores for *Crocodylus anthropophagus* were updated according to information in Azzarà *et al.* (2021), whereas the scores for *Globidentosuchus brachyrostris* and *Tsoabichi greenriverensis* were amended according to Walter *et al.* (2022). Also, character 101 (scoring the presence or absence of a linear array of pits on the medial surface of the maxilla) was changed from state 0 (surface of maxilla within narial canal imperforate) to state 1 (surface of maxilla within narial canal with a linear array of pits) for *Gavialis gangeticus*. This score is amended after figure 4B in Bourke *et al.* (2021) and observation of the 3D PDF by Pierce *et al.* (2017) containing a digital model of *Gavialis gangeticus* (**Fig. S3.1**), where a linear array of pits (=cecal recesses) is clearly present in both specimens.

Modification to the scores of some Australian crocodylians

Some of the scores for the Australian crocodyliforms were also updated after reassessment of available specimens. For the taxa included in the matrix of Ristevski *et al.* (2020a, b, 2021), the most significant changes affect *Harpacochampsa camfieldensis*. While not included in the matrices of Ristevski *et al.* (2020a, b, 2021), *Trilophosuchus rackhami* has been incorporated in many datasets based on Brochu (1997b, 1999). Thus, the scores for *T. rackhami* from the older matrices have been amended in light of the observations made in this study.

Harpacochampsa camfieldensis

The scores for *Harpacochampsa camfieldensis* in the matrices of Ristevski *et al.* (2020b, 2021) were reassessed based on a re-examination of the material assigned to this taxon (see "Taxon list" above). Based on the new observations, several characters had their scores amended from those previously assigned by Ristevski *et al.* (2020b, 2021).

Character 48: In the matrix of Ristevski *et al.* (2020b, 2021), this character was assigned state 0 (anterior dentary teeth strongly procumbent). However, the anterior alveoli of the dentary fragment NTM P87106-6 are indicative of teeth that would have projected anterodorsally (see figure 5 in Megirian *et al.*, 1991). Therefore, this character was rescored to state 1.

Character 92: Ristevski *et al.* (2020b, 2021) scored this character as an unknown ('?'). A reinspection of the preserved maxillary rostrum that is assigned to *H. camfieldensis* (NTM P87106-5) has reception pits for receiving dentary teeth during occlusion between every preserved alveolus (which is from maxillary alveolus one through five on the left maxilla; see figure 5 in Megirian *et al.*, 1991; also, **Fig. S3.2B**). This observation allows to score this character with state 2 (fully interlocking dentition with interdental reception pits from maxillary tooth one to at least tooth nine).

Character 119: Ristevski *et al.* (2020b, 2021) also had this character scored as an unknown ('?'). As can be seen from the holotype specimen of *H. camfieldensis* (NTM P87106-1), the pterygoid ramus of the ectopterygoid is straight (see figure 6B in Megirian *et al.*, 1991). Thus, the correct score for this character is state 0.

Character 126: This is another character that was scored as an unknown ('?') by Ristevski *et al.* (2020b, 2021). As evidenced from the holotype specimen of *H. camfieldensis* (NTM P87106-1; see figure 6B in Megirian *et al.*, 1991), the ectopterygoid-pterygoid flexure is not present in NTM P87106-1, which allows to score this character with state 0.

Character 127: Character 127 for *H. camfieldensis* was also scored as an unknown ('?') by Ristevski *et al.* (2020b, 2021). In their figure 6E, Megirian *et al.* (1991) correctly illustrated the preserved pterygoid on the right side of NTM P87106-1, which shows that the ectopterygoid does not extend to the posterolateral tip of the pterygoid (state 1).

Character 146 (=Character 147 in Ristevski *et al.*, 2020b, 2021): In *H. camfieldensis*, the dorsal and ventral rims of the squamosal are slightly diverging anteriorly and are thus not parallel to each other (see figures 6D and 6E in Megirian *et al.*, 1991). Although this anterior flaring is subtle, the

correct score would be state 1 instead of the previous score as 0 that was applied by Ristevski *et al.* (2020b, 2021).

Character 158 (=Character 159 in Ristevski *et al.*, 2020b, 2021): The previous score applied to this character was state 1 (the squamosal extends ventrolaterally to the lateral extent of the paroccipital process). As correctly illustrated in figure 6D of Megirian *et al.* (1991), the squamosal of *H. camfieldensis* does not extent ventrolaterally to cover the paroccipital process. Thus, this score was corrected to state 0.

Character 163 (=Character 165 in Ristevski *et al.*, 2020b, 2021): Ristevski *et al.* (2020b, 2021) had this character scored as an unknown ('?') for *H. camfieldensis*. A reinspection of NTM P87106-1 reveals that the ascending process of the pterygoid does not contribute to the formation of the lateral bridge of the laterosphenoid (state 0).

Character 165 (=Character 167 in Ristevski *et al.*, 2020b, 2021): Damage on the holotype specimen of *H. camfieldensis* makes identifying the presence of a pneumatic recess within the parietal not immediately obvious, as the breakage of the bone may lead to confusion as to what could be a pneumatic recess and what is a taphonomic induced perforation. However, a closer look at the broken parietal reveals that *H. camfieldensis* does indeed possess a pneumatic recess, which allows an amendment to this score from an unknown ('?') to state 0. Megirian *et al.* (1991) identified the parietal pneumatic recess of the *H. camfieldensis* holotype as the "?neural pocket of Tarsitano *et al.* (1989)" and "?posterior pocket of Tarsitano *et al.* (1989)" (see figure 6F in Megirian *et al.*, 1991).

Character 208 (=Character 210 in Ristevski *et al.*, 2020b, 2021): The frontoparietal fossa (*sensu* Holliday *et al.*, 2020) of *H. camfieldensis* does not extend anteromedially on the cranial table. This observation required a rescoring of this character from state 1 to 0.

Character 215 (=Character 217 in Ristevski *et al.*, 2020b, 2021): This character scores the presence or absence of pterygoid ridges around the lateral margins of the secondary choana. Previously, Ristevski *et al.* (2020b, 2021) had this character scored as an unknown ('?').

Observation of the preserved right pterygoid plate indicates that there are no ridges on the pterygoid lateral to the secondary choana. Hence, this character was amended to state 0.

Modifications to the scores for *Trilophosuchus rackhami* from older matrices

The scores for the following characters were amended from those applied in older matrices based on Brochu (1997b), such as Brochu & Storrs (2012), Narváez *et al.* (2015), Mateus *et al.* (2019), and Massonne *et al.* (2019).

Character 85: In older matrices, *Trilophosuchus rackhami* was scored with state 0 for this character (external naris opens flush with dorsal surface of premaxillae). This has been rescored as an unknown ('?') since the external naris is not preserved in any known specimen currently referred to *T. rackhami*.

Character 97: For this character, *T. rackhami* was scored with state 0 (preorbital ridges absent or very modest). Preorbital ridges typically occur immediately anterior or near the anterior margins of the orbits, which is a region that, unfortunately, is imperfectly preserved in the *T. rackhami* holotype specimen (only a small extent anterior to the left orbit remains). As such, it is more prudent to have it scored as an unknown ('?').

Character 102: Previously, *T. rackhami* was scored as state 1 for this character (medial jugal foramen very large). As can be observed on the better preserved right jugal of the *T. rackhami* holotype (**Fig. 11B**), the medial jugal foramen is not particularly large (~0.8 mm in diameter), making state 0 (medial jugal foramen small) the more appropriate score.

Character 103: The previous scoring as state 0 (maxillary foramen for palatine ramus of cranial nerve V small or not present) has been changed to state 1 (maxillary foramen for palatine ramus of cranial nerve V very large). These foramina are well preserved on both maxillae in the *T. rackhami* holotype and they are proportionally very large (**Figs. 1B**, **2B**, **4A** and **4B**).

Character 106: The previous score as an unknown ('?') has been amended to state 0 (penultimate maxillary alveolus less than twice the diameter of the last maxillary alveolus). The last two maxillary alveoli are preserved on both left and right sides of the *T. rackhami* holotype specimen, with the penultimate maxillary alveoli being inappreciably larger than the last alveoli.

Character 107: The previous score as an unknown ('?') has been changed to state 0 (prefrontal dorsal surface smooth adjacent to orbital rim), as deduced from the corresponding portion still remaining of the left prefrontal in the *T. rackhami* holotype specimen (**Figs. 1A**, **2A** and **5G**).

Character 108: This character was rescored from an unknown ('?') to state 1 (dorsal half of prefrontal pillar expanded anteroposteriorly). Observation of the *T. rackhami* holotype specimen reveals sufficient dorsal expansion of the prefrontal descending process to justify this scoring as state 1.

Character 111: This character was scored as an unknown ('?') for *T. rackhami*. Based on observations of the left prefrontal of QMF16856 (**Figs. 5C** and **5D**), the medial process of the prefrontal pillar does not display a constriction along its dorsal margin (or the constriction is mild at most). Therefore, this character was rescored into state 0.

Character 116: The old score given for this character was state 0 (palatine process generally broad anteriorly). However, the incompleteness of the anterior processes of the palatines at their tips makes their real morphology unclear (**Figs. 1B**, **2B** and **14**). Hence, this character was rescored into an unknown ('?').

Character 122: The secondary choana of the *T. rackhami* holotype specimen is insufficiently preserved to reliably determine whether it was oriented posteroventrally or anteroventrally. Thus, the scoring for this character was changed from state 0 (choana projects posteroventrally) to unknown ('?').

Character 131: In *T. rackhami*, the anterior process of the frontal is relatively broad at its anterior margin (**Figs. 1A**, **2A**, **7A** and **S2.1A–S2.1C**). Therefore, the score for this character was amended

from an unknown ('?') to state 1 (anterior tip of the frontal forms broad, complex sutural contact with the nasals).

Character 135: In *T. rackhami*, the ascending process of the jugal forming the ventral portion of the postorbital bar is flush (or faintly inset from at most) with the lateral surface of the jugal. As such, the scoring for this character was changed from state 1 (ventral margin of postorbital bar inset from lateral jugal surface) to state 0 (ventral margin of postorbital bar flush with lateral jugal surface).

Character 140: In the *T. rackhami* holotype specimen, the quadratojugal portions around both infratemporal (=laterotemporal) fenestrae are incomplete, yet sufficiently well preserved to offer no hints of quadratojugal spines. Therefore, this character's score was changed from an unknown ('?') to state 1 (quadratojugal spine greatly reduced or absent at maturity).

Characters 143 and 145: The quadratojugals of the *T. rackhami* holotype specimen are incomplete, particularly the portions that formed/contributed to the dorsal margins of the infratemporal fenestrae. Thus, it is unclear what the relations between the quadratojugals and postorbitals were like in this region. Because of this, characters 143 and 145 were rescored from state 0 and state 1, respectively, to unknowns ('?').

Character 153 (=Character 154 in Brochu & Storrs, 2012): Several small foramina perforate both descending processes of the parietals (=cristae cranii parietales; **Fig. S1.3**). Due to this, the character was rescored from state 0 (medial parietal wall of supratemporal fenestra imperforate) to state 1 (medial parietal wall of supratemporal fenestra bearing foramina).

Character 160 (=Character 162 in Brochu & Storrs, 2012): In older matrices, this character was scored as state 1 for *T. rackhami* (braincase wall lateral to parabasisphenoid rostrum smooth, without sulcus). Unfortunately, the portion of the parabasisphenoid body lateral to the (now missing) rostrum is fractured and rather poorly preserved in QMF16856. Confident identification of a sulcus, or lack thereof, is hampered. To err on the side of caution, we recommend this character be scored as an unknown ('?') instead.

Character 165 (=Character 167 in Brochu & Storrs, 2012): Thanks to the μCT data of the *T. rackhami* holotype specimen, it is evident that the parietal has a substantial pneumatic recess (**Fig. 3**), allowing to change the score for this character from an unknown ('?') to state 0 (parietal with recess communicating with pneumatic system).

Character 166 (=Character 168 in Brochu & Storrs, 2012): The lateral walls of the braincase in the *T. rackhami* holotype are poorly preserved as they are affected by fractures that obscure the true outlines of the sutures in this region. As such, accurately determining the path of the quadrate-pterygoid suture is difficult. Considering the preservational condition of this part of the skull in QMF16856, we prefer to score this character as an unknown ('?').

Character 170 (=Character 172 in Brochu & Storrs, 2012): Previously, *T. rackhami* was scored with state 0 (parabasisphenoid thin ventral to basioccipital). However, as can be seen in the holotype specimen (**Figs. 2B** and **21B**), the parabasisphenoid has an obvious exposure between the basioccipital and pterygoids in ventral view. This degree of exposure of the parabasisphenoid present in QMF16856 (~2 mm in anteroposterior length) more closely resembles the description for state 1 instead of state 0.

Character 176 (=Character 178 in Brochu & Storrs, 2012): The older scoring for this character was left as an unknown ('?'). Observation of the *T. rackhami* holotype specimen (**Fig. S1.11**) reveals that the quadrate foramina aërea are small (state 0).

Character 184 (=Character 187 in Brochu & Storrs, 2012): Previously scored as an unknown ('?'), this character has been altered to state 0 (palatine-maxillary suture intersects suborbital fenestra at its anteromedial margin). While the entirety of the palatine-maxillary sutures is not preserved in the *T. rackhami* holotype specimen, substantial lengths of the sutures are discernable around the anteromedial margins of the suborbital fenestrae (**Figs. 1B** and **2B**).

Character 185 (=Character 188 in Brochu & Storrs, 2012): Older scoring of this character had assigned state 0 (frontal lacks prominent midsagittal crest between orbits) to *Trilophosuchus*. The scoring is now revised into state 1 (frontal bears prominent midsagittal crest between orbits), as

one of the distinguishing features for *T. rackhami* is the presence of three sagittal crests on the cranial table, including a midsagittal crest that is spread over most of the frontal's dorsal lamina (**Figs. 1A**, **2A**, **7A**, **S2.1B** and **S2.1C**).

Character 194 (=Character 197 in Massonne *et al.*, 2019): In the matrix of Massonne *et al.* (2019), *T. rackhami* was scored as an unknown ('?') for this character. The scoring was amended into state 0 (sutural contact of the otoccipitals dorsal to foramen magnum long, at least half the height of the foramen magnum), as the medial sutural contact between the otoccipitals is ~4 mm, or over half the dorsoventral height of the foramen magnum which is ~6 mm in the *T. rackhami* holotype specimen.

Character 196 (=Character 200 in Massonne *et al.*, 2019): In the matrix of Massonne *et al.* (2019), this character was scored as inapplicable ('-') for *T. rackhami*. However, this character scores a mandibular feature, and since no known mandibular elements can currently be referred to *Trilophosuchus* with confidence, the character was rescored as an unknown ('?').

Character 198 (=Character 202 in Massonne *et al.*, 2019): In the matrix of Massonne *et al.* (2019), *T. rackhami* was scored as an unknown ('?') for this character as well. *Trilophosuchus rackhami* has a large dorsal exposure of the supraoccipital (state 2 for character 159), with the dorsal lamina of the supraoccipital adapting a trapezoid shape on the cranial table (state 0; **Figs. 1A**, **2A** and **17A**).

Modifications to the scores for *Trilophosuchus rackhami* for Rio & Mannion (2021)

Recently, *Trilophosuchus rackhami* was included in the matrix of Rio & Mannion (2021). Based on the observations made in this study, we suggest that some of the scores for *T. rackhami* in the matrix of Rio & Mannion (2021) be revised after the elaborations below.

Character 2: In the matrix of Rio & Mannion (2021), *T. rackhami* was scored as an unknown ('?') for character 2. Following the scoring instructions provided in figure 1B in Appendix 2 of Rio & Mannion (2021), it is possible to estimate the ratio of the mediolateral width of the rostrum at the

level of the anterior orbital margins to the mediolateral width across anterior margin of the cranial table. Although the rostrum of the *T. rackhami* holotype specimen is largely missing, it is sufficiently preserved anterior to the orbits so that it can be measured. Thus, the mediolateral width of the rostrum in QMF16856 immediately anterior to the orbits is ~43 mm, whereas the mediolateral width across the anterior margin of the cranial table is ~36 mm. As the ratio of the mediolateral rostrum width at the level of the anterior orbital margin to the mediolateral width across anterior margin of the cranial table is 1.19, this character can be scored as state 0 (ratio < 3).

Character 6: In the matrix of Rio & Mannion (2021), *T. rackhami* was scored as an unknown ('?') for this character. Fortunately, the holotype of *T. rackhami* is adequately preserved to allow scoring of this character. Following the measuring instructions provided in figure 1F in Appendix 2 of Rio & Mannion (2021), the minimum interorbital distance measured in QMF16856 is ~10.65 mm whereas the maximum width across the cranial table is ~36 mm. Therefore, as the ratio is 0.3 this character can be scored as state 0.

Character 8: Originally, *T. rackhami* was scored as an unknown ('?') for character 8. Following the measuring instructions provided in figure 1H in Appendix 2 of Rio & Mannion (2021), the maximum anteroposterior cranial table length (measured at the level of the frontal-postorbital suture) in QMF16856 is ~34 mm, while the maximum mediolateral width is of the cranial table ~37 mm (measured at the level of the postorbital-squamosal suture). Thus, the ratio between the length and width of the cranial table is 0.92, which allows this character to be scored as state 0.

Character 17: This character scores for the number of maxillary alveoli. Previously, *T. rackhami* was scored as an unknown ('?') for this character. The exact number of maxillary alveoli in the *T. rackhami* holotype specimen cannot be estimated with confidence due to the nature of preservation of the specimen (see main text). Nevertheless, the number of maxillary alveoli in QMF16856 is either 12 or 13, which allows scoring for this character with state 0 (less than 18 maxillary alveoli).

Character 37: For this character, *T. rackhami* was originally scored with state 1 (cranial table planar across entire length, or lateral edges directed dorsolaterally < 20° across entire length). As evident in QMF16856, the posterolateral edges of the cranial table slope ventrolaterally (**Figs. 1E**, **2E**, **S1.2A**, **S1.5A**, and **S1.11A**), and so the character score was changed to 0.

Character 57: *Trilophosuchus rackhami* was scored as an unknown ('?') for this character as well. Character 57 in the matrix of Rio & Mannion (2021) scores the mediolateral width of the lacrimal relative to the mediolateral width of the prefrontal. Although the left prefrontal and lacrimal are not complete in the *T. rackhami* holotype specimen (QMF16856), they are sufficiently preserved to allow scoring for this character. Because the facial lamina of the lacrimal in *T. rackhami* is primarily oriented laterally, the lacrimal was along its dorsoventral axis. The facial lamina of the lacrimal in QMF16856 is ~6 mm, whereas the maximum width of the prefrontal is ~5mm. This result indicates that the lacrimal is less than twice the maximum width of the prefrontal (state 1).

Character 63: The previous score as an unknown ('?') was changed into state 1, as the anterior tip of the jugal terminates posterior to the anterior tip of the frontal (**Figs. 1A** and **2A**).

Character 69: This character was scored as an unknown ('?'). Based on observations of the left prefrontal of QMF16856 (see **Fig. 5D**), the medial process of the prefrontal pillar does not display a constriction along its dorsal margin (or the constriction is mild at most). Therefore, this character was rescored into state 0.

Character 85: Originally, this character was scored as state 0 for *T. rackhami* (parietal medial wall of the supratemporal fenestra without foramina). However, as described in this study (**Fig. S1.3**), the descending processes of the parietal of QMF16856 are pierced by multiple foramina, and thus the correct score for this character is state 1.

Character 86: The score applied for this character by Rio & Mannion (2021) was an unknown ('?'). Thanks to the µCT scan of the *T. rackhami* holotype specimen, it is now revealed that the parietal of this taxon is occupied by a substantial pneumatic recess (state 0).

Character 92: This character scores the relations between the lateral surface of the jugal and its ascending process (i.e., the base of the postorbital bar). In the *T. rackhami* holotype specimen, the ascending process of the jugal is almost flush with the lateral surface of the jugal, only mildly inset medially at the most (see the interactive 3D PDF). The postorbital bar of *T. rackhami* is not markedly inset from the lateral surface of the jugal, and there is no sulcus to separate the base of the bar from the lateral jugal surface. Therefore, this character was rescored from state 1 (the dorsolateral margin of the jugal raised to form a ridge, with sulcus separating it from postorbital bar) to state 0 (postorbital bar is flush with the dorsolateral margin of jugal).

Character 98: The dorsal margins of both infratemporal fenestrae are incomplete in the *T. rackhami* holotype specimen. Due to this incompleteness, it is unclear if the dorsal margin of the fenestra was sub-triangular (state 0; the original state applied to *T. rackhami* by Rio & Mannion, 2021) or more broadly curved (state 1). Until a specimen that completely preserves the margins of the infratemporal fenestrae is discovered, it would be prudent to have this character scored as an unknown ('?') for *T. rackhami*.

Characters 99, 100, and 106: The quadratojugals of the *T. rackhami* holotype specimen are incomplete, particularly the portions that formed/contributed to the dorsal margins of the infratemporal fenestrae. Thus, it is unclear what the relations between the quadratojugals and postorbitals were like in this region. Because of this, characters 99, 100, and 106 were rescored from state 1, state 0, and state 0, respectively, to unknowns ('?').

Character 103: In the *T. rackhami* holotype specimen, the quadratojugal portions around both infratemporal fenestrae are incomplete, yet sufficiently well preserved to offer no hints of quadratojugal spines. Therefore, this character's score was changed from an unknown ('?') to state 1 (quadratojugal spine greatly reduced or absent at maturity).

Character 126: This character scores the relative distance between the posterior carotid foramen (lateral carotid foramen in Rio & Mannion, 2021) and the common external foramen for the glossopharyngeal, vagus, accessory, and sympathetic nerves on the otoccipital. Originally, *T.*

rackhami was assigned state 0 for this character, where the posterior carotid foramen is considered separated, and not adjacent, to the common external foramen for IX-XI + SN. In *T. rackhami* (but also *Harpacochampsa camfieldensis* and *Paludirex vincenti*, among Australian Cenozoic taxa) the posterior carotid foramen is located ventral to the common external foramen for IX-XI + SN, but separated from the former by a small distance, considerably less so than most OTUs scored with state 0 in the matrix. Therefore, *T. rackhami* was rescored to state 1.

Character 135: The character was previously scored as an unknown ('?'). As can be seen in **Fig. 2E**, the parabasisphenoid of *T. rackhami* is exposed ventral to the basioccipital in occipital view, although it is not particularly tall (state 0).

Character 137: In the matrix of Rio & Mannion (2021), this character, scoring the relative position of the premaxillary-maxillary suture to the incisive foramen, was scored with state 0. Since the premaxillae, along with the incisive foramen and the anterior of the maxillae are unknown in *T. rackhami*, the character was rescored as '?'.

Character 151: In the *T. rackhami* holotype specimen, there is one pit for a dentary tooth between the maxillary alveoli (probably between maxillary alveoli seven and eight; **Fig. 4A**) which implies partial interlocking dentition. Therefore, the previous score as an unknown ('?') was amended to state 1 (partial interlocking occlusion, with at least one pit between maxillary teeth 5–8).

Character 166: This character scores the position of the anterior-most margin of the suborbital fenestra relative to the anterior-most margin of the orbit. The previous score applied to *T. rackhami* for this character was an unknown ('?'). When the cranium of QMF16856 is observed in either dorsal (**Figs. 1A** and **2A**) or ventral (**Figs. 1B** and **2B**) views, the anterior margin of the suborbital fenestra is evidently more anterior to the anterior margin of the orbit. Based on this observation, state 0 is applied to *T. rackhami*.

Character 171: In the *T. rackhami* holotype, the posterolateral margins of the suborbital fenestrae at the ectopterygoid-pterygoids sutures are subtly bowed anteromedially (**Figs. 1B** and **2B**).

Although the anteromedial expansion is subtle, it clearly differs from the rounded margin present in most eusuchians. Therefore, this character was rescored from state 0 to state 1.

Character 176: The previous scoring as an unknown ('?') was updated to state 0. The anterior process of the ectopterygoid is mostly preserved on the right ectopterygoid, which is a singular acute process without a bifurcation.

Character 200: This character, scoring the presence or absence of ventrally directed knob-like protrusions on the quadrate body, was marked as an unknown ('?') in *T. rackhami*. Knob-like protrusions on the ventral surface of the quadrate occur in *Hylaeochampsa vectiana* and *Iharkutosuchus makadii*, whereas most other eusuchians are characterized by comparatively less-developed crests (Iordansky 1964, 1973; Ösi, 2008; Kuzmin *et al*., 2021; Rio & Mannion, 2021). As in the vast majority of eusuchians, *T. rackhami* also possesses a quadrate that bears crests that are not as hypertrophied as those of the aforementioned hylaeochampsids, and therefore the correct state for this character is 0 (**Figs. 22D** and **23D**).

Character 201: Previously, *T. rackhami* was scored with state 0 (parabasisphenoid not or poorly exposed between the basioccipital and pterygoids in ventral view, parabasisphenoid anteroposteriorly short). However, as can be seen in the holotype specimen (**Figs. 2B** and **21B**), the parabasisphenoid has an obvious exposure between the basioccipital and pterygoids in ventral view. This degree of exposure of the parabasisphenoid seen in QMF16856 (~2 mm in anteroposterior length) more closely resembles the description for state 1 instead of state 0.

Character 205: The original score applied to *T. rackhami* for this character was 0 (parabasisphenoid with sulcus on anterior braincase wall, lateral to the parabasisphenoid rostrum). Unfortunately, the portion of the parabasisphenoid body lateral to the (now missing) rostrum is fractured and rather poorly preserved in QMF16856. Confident identification of a sulcus, or lack thereof, is hampered. To err on the side of caution, we recommend this character be scored as an unknown ('?') instead.

Characters 209 and 210: These two characters score the presence or absence of the caudal bridge of the laterosphenoid (character 209), and if present, the morphology of the caudal (=posterior) bridge (character 210). The caudal bridge is absent on the right laterosphenoid of the *T. rackhami* holotype specimen, but it is present on the left (**Fig. 15**). The previous score for character 209 was state 0 (caudal bridge absent). Based on our observations of QMF16856, the lack of a caudal bridge on the right laterosphenoid bridge does not appear to be taphonomic. Due to the asymmetry in development of this feature in the same specimen, we rescored this character as both present and absent (0,1). The caudal bridge on the left laterosphenoid is relatively short, and therefore character 210 was rescored from an unknown ('?') to state 0 (caudal laterosphenoid bridge short).

Character 215: The lateral walls of the braincase in the *T. rackhami* holotype are poorly preserved as they are affected by fractures that obscure the true outlines of the sutures in this region. As such, accurately determining the path of the quadrate-pterygoid suture is difficult. Considering the preservational condition of this part of the skull in QMF16856, we prefer to score this character as an unknown ('?').

Character 311: The digital reconstruction of the brain endocast of the *T. rackhami* holotype specimen reveals that the region of the endocast dorsal to the optic lobes (mesencephalon, or midbrain region) is markedly concave, which allows this character to be scored with state 1.

Character 312: This character scores the ratio between the dorsoventral length of the median pharyngeal canal (or median pharyngeal tube) relative to the dorsoventral length of the pneumatic recesses of the parabasisphenoid (note that the parabasisphenoid of crocodylians is usually pneumatized by multiple recesses; see Kuzmin *et al*., 2021). The digital reconstruction of the paratympanic pneumatic system in QMF16856 indicates that the median pharyngeal canal is very short, which allows to have this character scored as state 1.

After the above elaborated amendments, the revised scores for *Trilophosuchus rackhami* for the matrix of Rio & Mannion (2021) are:

?	0	?	?	?	0	0	0	0	1	0	?
0	?	1	0	0	?	?	?	?	?	?	?
?	?	?	?	?	0	0	?	?	0	0	0
0	0	?	?	?	?	?	?	?	?	?	?
?	?	?	1	?	?	?	?	1	?	0	1
0	0	1	0	1	1	?	0	0	1	0	1
1	?	2	1	0	1	1	0	0	0	1	1
1	0	0	1	1	1	1	0	0	0	0	0
0	?	?	?	?	1	1	?	0	?	0	0
1	1	0	0	0	1	1	0	0	1	0	0
0	0	0	0	0	1	0	0	0	0	0	0
0	0	0	?	?	?	?	?	?	?	?	?
?	?	?	0	?	0	1	?	?	?	0	?
?	0	1	?	?	1	0	0	0	0	0	1
1	0	1	1	1	1	2	0	0	0	0	0
0	1	1	0	0	1	1	2	?	?	0	?
?	?	?	?	0	?	0	0	1	0	?	1
?	1	1	1	[01]	0	0	1	?	0	?	?
?	?	?	?	?	?	?	?	?	?	?	?
?	?	?	?	?	?	?	?	?	?	?	?
?	?	?	?	?	?	?	?	?	?	?	?
?	?	?	?	?	?	?	?	?	?	?	?
?	?	?	?	?	?	?	?	?	?	?	?
?	?	?	?	?	?	?	?	?	?	?	?
?	?	?	?	?	?	?	?	?	?	?	?
?	?	?	?	?	?	?	?	?	?	1	1
?	?	?	?	?	?	?	?	?	?	?	?
?	?	?	?	?	?						

MORPHOLOGICAL CHARACTER LIST

The following morphological character list is based on the one used by Ristevski *et al.* (2020a, b; 2021). The original source(s) for each character is cited in brackets. For more details on the previous character lists, see Ristevski *et al.* (2020b, 2021).

1. Ventral tubercle of proatlas more than one-half (0) or no more than one-half (1) the width of the dorsal crest. (*Brochu, 1997b, ch. 1*)

2. Fused proatlas boomerang-shaped (0), strap-shaped (1), or massive and block-shaped (2). (*Brochu, 1997b, ch. 2*)

3. Proatlas with prominent anterior process (0) or lacks anterior process (1). (*Brochu, 1997b, ch. 10*)

4. Proatlas has tall dorsal keel (0) or lacks tall dorsal keel; dorsal side smooth (1). (*Brochu, 1997b, ch. 17*)

5. Atlas intercentrum wedge-shaped in lateral view, with insignificant parapophyseal processes (0) or plate-shaped in lateral view, with prominent parapophyseal processes at maturity (1). (*Brochu, 1997b, ch. 5; Clark, 1994, ch. 89, modified*)

6. Dorsal margin of atlantal rib generally smooth with modest dorsal process (0) or with prominent process (1). (*Brochu, 1997b, ch. 14*)

7. Atlantal ribs without (0) or with (1) very thin medial laminae at anterior end. (*Brochu, 1997b, ch. 16*)

8. Atlantal ribs lack (0) or possess (1) large articular facets at anterior ends for each other. (*Brochu, 1997b, ch. 15*)

9. Axial rib tuberculum wide, with broad dorsal tip (0) or narrow, with acute dorsal tip (1). (*Brochu, 1997b, ch. 20*)

10. Axial rib tuberculum contacts diapophysis late in ontogeny, if at all (0) or early in ontogeny (1). (*Brochu, 1997b, ch. 21*)

11. Anterior half of axis neural spine oriented horizontally (0) or slopes anteriorly (1). (*Brochu, 1997b, ch. 11*)

12. Axis neural spine crested (0) or not crested (1). (*Brochu, 1997b, ch. 12*)

13. Posterior half of axis neural spine wide (0) or narrow (1). (*Brochu, 1997b, ch. 3*)

14. Axis neural arch lacks (0) or possesses (1) a lateral process ("diapophysis"). (*Brochu, 1997b, ch. 4; adapted from Norell, 1989, ch. 7*)

15. Axial hypapophysis located toward the center of centrum (0) or toward the anterior end of centrum (1). (*Brochu, 1997b, ch. 6*)

16. Axial hypapophysis without (0) or with (1) deep fork. (*Brochu, 1997b, ch. 19*)

17. Hypapophyseal keels present on 11th vertebra behind atlas (0), 12th vertebra behind atlas (1), or 10th vertebra behind atlas (2). (*Brochu, 1997b, ch. 7, modified*)

18. Third cervical vertebra (first postaxial) with prominent hypapophysis (0) or lacks prominent hypapophysis (1). (*Brochu, 1997b, ch. 8; adapted from Norell, 1989, ch. 12; Norell & Clark, 1990, ch. 11; Clark, 1994, ch. 91*)

19. Neural spine on third cervical long, dorsal tip at least half the length of the centrum without the cotyle (0) or short, dorsal tip acute and less than half the length of the centrum without the cotyle (1). (*Brochu, 1997b, ch. 9, modified*)

20. Cervical and anterior dorsal centra lack (0) or bear (1) deep pits on the ventral surface of the centrum. (*Brochu & Storrs, 2012, ch. 20*)

21. Presacral centra amphicoelous (0), or weakly procoelous (1), or strongly procoelous (2). **ORDERED**. (*Salisbury* et al.*, 2006, ch. 18, modified; adapted from several previous datasets, e.g., Benton & Clark, 1988; Norell & Clark, 1990, ch. 8 and 10 modified; Clark, 1994, ch. 92 and 93; Brochu, 1997b, ch. 18*)

22. Anterior sacral rib capitulum projects far anteriorly of tuberculum and is broadly visible in dorsal view (0) or anterior margins of tuberculum and capitulum nearly in same plane, and capitulum largely obscured dorsally (1). (*Brochu, 1997b, ch. 13*)

23. Scapular blade flares dorsally at maturity (0) or sides of scapular blade subparallel; minimal dorsal flare at maturity (1). (*Brochu, 1997b, ch. 22; adapted from Benton & Clark, 1988*)

24. Deltoid crest of scapula very thin at maturity, with sharp margin (0) or very wide at maturity, with broad margin (1). (*Brochu, 1997b, ch. 23*)

25. Scapulocoracoid synchondrosis closes very late in ontogeny (0) or relatively early in ontogeny (1). (*Brochu, 1997b, ch. 24*)

26. Scapulocoracoid facet anterior to glenoid fossa uniformly narrow (0) or broad immediately anterior to glenoid fossa and tapering anteriorly (1). (*Brochu, 1997b, ch. 25*)

27. Proximal edge of deltopectoral crest emerges smoothly from proximal end of humerus and is not obviously concave (0) or emerges abruptly from proximal end of humerus and is obviously concave (1). (*Brochu, 1997b, ch. 26*)

28. *M. teres major* and *m. dorsalis scapulae* insert separately on humerus; scars can be distinguished dorsal to deltopectoral crest (0) or insert with common tendon; single insertion scar (1). (*Brochu, 1997b, ch. 29*)

29. Olecranon process of ulna narrow and sub-angular (0) or wide and rounded (1). (*Brochu, 1997b, ch. 27*)

30. Distal extremity of ulna expanded transversely with respect to long axis of bone; maximum width equivalent to that of proximal extremity (0) or proximal extremity considerably wider than distal extremity (1). (*Salisbury* et al.*, 2006, ch. 173*)

31. Interclavicle flat along length, without dorsoventral flexure (0), or with moderate dorsoventral flexure (1), or with severe dorsoventral flexure (2). (*Brochu, 1997b, ch. 30*)

32. Anterior end of interclavicle flat (0) or rod-like (1). (*Brochu, 1997b, ch. 31*)

33. Pre-acetabular process of ilium present as a prominent triangular process (0) or present only as a barely perceptible bump to absent altogether (1). (*Lee & Yates, 2018, ch. 256; based on Brochu, 1997b, ch. 34)*

34. Dorsal margin of iliac blade rounded with smooth border (0), or rounded, with modest dorsal indentation (1), or rounded, with strong dorsal indentation ('wasp-waisted'; 2), or narrow, with dorsal indentation (3), or rounded with smooth border; posterior tip of blade very deep (4). (*Brochu, 1997b, ch. 28*)

35. Supraacetabular crest narrow (0) or broad (1). (*Brochu, 1997b, ch. 32*)

36. Limb bones relatively robust, and hind limb much longer than forelimb at maturity (0) or limb bones very long and slender (1). (*Brochu, 1997b, ch. 33, modified*)

37. *M. caudofemoralis* with single head (0) or with double head (*m. caudofemoralis longus* and *m. caudofemoralis brevis*; 1). (*Salisbury* et al., *2006, ch. 160*; *modified from Brochu, 1997b, ch. 160*)

38. Dorsal osteoderms keeled (0) or not keeled (1). (*Salisbury* et al., *2006, ch. 35; polarity reversed from the version in Brochu, 1997b, ch. 35; adapted from Buscalioni* et al., *1992, ch. 22*)

39. Biserial dorsal shield, dorsal osteoderms rectangular in outline with distinct medial and lateral parts either side of a sagittal keel (0), or dorsal osteoderms segmented sagittally into rectangular paravertebral osteoderms and square to round accessory osteoderms (1), or paravertebral osteoderms segmented (2). **ORDERED**. (*Salisbury* et al., *2006, ch. 36; adapted from Brochu, 1997b, ch. 36; Norell & Clark, 1990, ch. 16; Clark, 1994, ch. 95*)

40. Accessory osteoderms absent (0), or maximum of one longitudinal row of transversely contiguous accessory osteoderms (1), or maximum of two longitudinal rows of transversely contiguous accessory osteoderms (2), or maximum of three sagittal longitudinal rows of transversely contiguous accessory osteoderms (3). (*Salisbury* et al., *2006, ch. 37; adapted from Brochu, 1997b, ch. 37; Norell & Clark, 1990, ch. 12; Clark, 1994, ch. 97*)

41. Nuchal shield grades continuously into dorsal shield (0), or nuchal shield differentiated from dorsal shield into four nuchal osteoderms in two parallel rows (1), or nuchal shield differentiated from dorsal shield into six nuchal osteoderms, with four central and two lateral (2), or nuchal shield differentiated from dorsal shield into more than four nuchal osteoderms in two parallel rows (3). (*Salisbury* et al., *2006, ch. 38; modified from Brochu, 1997b, ch. 38*)

42. Ventral osteoderms present, polygonal (0), or present, square (1), or present, paired ossifications that suture together (2), or absent (3). (*Salisbury* et al., *2006, ch. 39; Brochu, 1997b, ch. 39 modified; adapted from Buscalioni* et al., *1992, ch. 21; Clark, 1994, ch. 100*)

43. Anterior margin of dorsal midline osteoderms with anterior process (0) or smooth, without process (1). (*Brochu, 1997b, ch. 40; adapted from Norell & Clark, 1990, ch. 13; Clark, 1994, ch. 96*)

44. Ventral scales have (0) or lack (1) follicle gland pores. (*Brochu, 1997b, ch. 155*)

45. Ventral collar scales not enlarged relative to other ventral scales (0), or in a single enlarged row (1), or in two parallel enlarged rows (2). (*Brochu, 1997b, ch. 156; Poe, 1996*)

46. Median pelvic keel scales form two parallel rows along most of tail length (0), or form single row along tail (1), or merge with lateral keel scales (2). (*Brochu, 1997b, ch. 157; Poe, 1996*)

47. Alveoli for dentary teeth 3 and 4 nearly same size and confluent (0) or fourth alveolus larger than third, and alveoli are separated (1). (*Brochu, 1997b, ch. 52*)

48. Anterior dentary teeth strongly procumbent (0) or project anterodorsally (1). (*Brochu, 1997b, ch. 53*)

49. Dentary symphysis extends to fourth or fifth alveolus (0), or sixth through eighth alveolus (1), or behind eighth alveolus (2). **ORDERED**. (*Brochu & Storrs, 2012, ch. 49; modified from Brochu, 2004b, ch. 166*)

50. Dentary gently curved (0), deeply curved (1), or linear (2) between fourth and tenth alveoli. **ORDERED**. (*Brochu, 1997b, ch. 68*)

51. Largest dentary alveolus immediately posterior to fourth is (0) 13 or 14, (1) between 11 or 14 and a series behind it, (2) 11 or 12, (3) no differentiation, (4) behind 14, (5) 10. (*Massonne* et al.*, 2019, ch. 51; modified from Brochu, 2004b, ch. 167*)

52. Splenial with anterior perforation for mandibular ramus of cranial nerve V (0) or lacks anterior perforation for mandibular ramus of cranial nerve V (1). (*Brochu, 1997b, ch. 41; adapted partially from Norell, 1988, ch. 15 and Norell, 1989, ch. 8*)

53. Mandibular ramus of cranial nerve V exits splenial anteriorly only (0), or splenial has singular perforation for mandibular ramus of cranial nerve V posteriorly (1), or splenial has double perforation for mandibular ramus of cranial nerve V posteriorly (2). (*Brochu, 1997b, ch. 42; adapted partially from Norell, 1988, ch. 15 and Norell, 1989, ch. 8*)

54. Splenial participates in mandibular symphysis; splenial symphysis adjacent to no more than five dentary alveoli (0), or splenial excluded from mandibular symphysis; anterior tip of splenial passes ventral to Meckelian groove (1), or splenial excluded from mandibular symphysis; anterior tip of splenial passes dorsal to Meckelian groove (2), or deep splenial symphysis, longer than five dentary alveoli; splenial forms wide 'V' within symphysis (3), or deep splenial symphysis, longer than five dentary alveoli; splenial constricted within

symphysis and forms narrow 'V' (4). **ORDERED**. (*Brochu, 1997b, ch. 43; adapted from Clark, 1994, ch. 77*)

55. Coronoid bounds posterior half of *foramen intermandibularis medius* (0), or completely surrounds *foramen intermandibularis medius* at maturity (1), or obliterates *foramen intermandibularis medius* at maturity (2). **ORDERED**. (*Brochu, 1997b, ch. 46; adapted from Norell, 1988, ch. 12*)

56. Superior edge of coronoid slopes strongly anteriorly (0) or almost horizontal (1). (*Brochu, 1997b, ch. 54*)

57. Inferior process of coronoid laps strongly over inner surface of Meckelian fossa (0) or remains largely on medial surface of mandible (1). (*Brochu, 1997b, ch. 55*)

58. Coronoid imperforate (0) or with perforation posterior to *foramen intermandibularis medius* (1). (*Brochu, 1997b, ch. 56*)

59. Process of splenial separates angular and coronoid (0) or no splenial process between angular and coronoid (1). (*Brochu, 1997b, ch. 59*)

60. Angular-surangular suture contacts external mandibular fenestra at posterior angle at maturity (0), or passes broadly along ventral margin of external mandibular fenestra late in ontogeny (1), or mandibular fenestra between dentary and angular, no surangular participation on the fenestra (2). (*Mateus* et al., *2019, ch. 60*; *Brochu, 1997b, ch. 47, modified; adapted from Norell, 1988, ch. 40*)

61. Dorsal and ventral anterior processes of surangular unequal (0) or sub-equal to equal (1). (*Brochu, 1997b, ch. 48, modified*)

62. Surangular with spur bordering the dentary toothrow lingually for at least one alveolus length (0) or lacking such spur (1). (*Brochu, 1997b, ch. 61*)

63. External mandibular fenestra absent (0), or present as narrow slit, no discrete fenestral concavity on angular dorsal margin (1), or present with discrete concavity on angular dorsal margin (2), or present and very large; most of *foramen intermandibularis caudalis* visible in lateral view (3). (*Brochu, 1997b, ch. 62 and 64, modified; adapted from Clark, 1994, ch. 75*; *includes information from Norell, 1988, ch. 14*)

64. Surangular-dentary suture intersects external mandibular fenestra anterior to posterodorsal corner (0) or at posterodorsal corner (1). (*Brochu, 1997b, ch. 65*)

65. Angular extends dorsally toward or beyond anterior end of *foramen intermandibularis caudalis*; anterior tip acute (0) or does not extend dorsally beyond anterior end of *foramen intermandibularis caudalis*; anterior tip very blunt (1). (*Brochu, 1997b, ch. 66*)

66. Surangular-angular suture lingually meets articular at ventral tip (0) or dorsal to tip (1). (*Brochu, 1997b, ch. 67, modified*)

67. Surangular continues to dorsal tip of lateral wall of glenoid fossa (0) or truncated and not continuing dorsally (1). (*Brochu, 1999, ch. 106*)

68. Articular-surangular suture simple (0), or articular bears anterior lamina dorsal to lingual foramen (1), or articular bears anterior lamina ventral to lingual foramen (2), or bears laminae above and below foramen (3). (*Brochu, 1997b, ch. 44, modified*)

69. Lingual foramen for articular artery and alveolar nerve perforates surangular entirely (0) or perforates surangular/angular suture (1). (*Brochu, 1997b, ch. 45, modified*)

70. Foramen aëreum at extreme lingual margin of retroarticular process (0) or set in from margin of retroarticular process (1). (*Brochu, 1997b, ch. 49; adapted from Norell, 1988, ch. 16*)

71. Retroarticular process projects posteriorly (0) or projects posterodorsally (1). (*Brochu, 1997b, ch. 50; adapted from Benton & Clark, 1988; Norell & Clark, 1990, ch. 7; Clark, 1994 ch. 71*)

72. Surangular extends to posterior end of retroarticular process (0) or pinched off anterior to tip of retroarticular process (1). (*Brochu, 1997b, ch. 51; adapted from Norell, 1988, ch. 42*)

73. Surangular-articular suture oriented anteroposteriorly (0) or bowed strongly laterally (1) within glenoid fossa. (*Brochu, 1997b, ch. 162*)

74. Sulcus between articular and surangular (0) or articular flush against surangular (1). (*Brochu, 1997b, ch. 60*)

75. Dorsal projection of hyoid cornu flat (0) or rod-like (1). (*Brochu, 1997b, ch. 57*)

76. Dorsal projection of hyoid cornu narrow, with parallel sides (0) or flared (1). (*Brochu, 1997b, ch. 58*)

77. Lingual osmoregulatory pores small (0) or large (1). (*Brochu, 1997b, ch. 158*)

78. Tongue with (0) or without (1) keratinized surface. (*Brochu, 1997b, ch. 159*)

79. Teeth and alveoli of maxilla and/or dentary circular to subcircular in cross-section (0), or posterior teeth labiolingually compressed (1), or all teeth labiolingually compressed (2). (*Adapted from Brochu, 2004b, ch. 165*)

80. Maxillary and dentary teeth with smooth carinae or carinae bearing superficial denticle-like crenulations (0) or serrated, bearing well-developed denticles (1). (*Brochu & Storrs, 2012, ch. 80, modified*)

81. External naris is oriented anterodorsally (0) or dorsally (1). (*Brochu, 1997b, ch. 79, modified*)

82. External naris bisected by nasals (0), or nasals contact external naris, but do not bisect it (1), or nasals excluded, at least externally, from naris; nasals and premaxillae still in contact (2), or nasals and premaxillae not in contact (3). **ORDERED**. (*Brochu, 1997b, ch. 95; adapted from Norell, 1988, ch. 3; Clark, 1994, ch. 13 and 14*)

83. External naris, mediolateral width to anteroposterior length ratio is ≤ 1 (0) or > 1 (1). (*Rio & Mannion, 2021, ch. 3; based on Brochu, 1997b, ch. 161*)

84. External naris of reproductively mature males (0) remains similar to that of females or (1) develops bony excrescence (ghara). (*Brochu & Storrs, 2012, ch. 84*)

85. External naris (0) opens flush with dorsal surface of premaxillae or (1) circumscribed by thin crest. (*Brochu & Storrs, 2012, ch. 85*)

86. Premaxillary surface lateral to naris smooth (0) or with deep notch lateral to naris (1). (*Brochu, 1997b, ch. 142*)

87. Premaxilla has five teeth (0) or four teeth (1) early in post-hatching ontogeny. (*Brochu, 1997b, ch. 97; adapted from Norell, 1988, ch. 17*)

88. Incisive foramen absent or small, less than half the greatest width of premaxillae (0), or large, more than half the greatest width of premaxillae (1), or large and intersects premaxillary-maxillary suture (2). (*Brochu, 1997b, ch. 124, modified*)

89. Incisive foramen completely situated far from premaxillary tooth row, at the level of the second or third alveolus, or posterior (0) or abuts premaxillary tooth row (1). (*Brochu, 1997b, ch. 153, modified*)

90. Dorsal premaxillary processes short, not extending beyond third maxillary alveolus (0) or long, extending beyond third maxillary alveolus (1). (*Brochu, 1997b, ch. 145*)

91. Dentary tooth 4 occludes in notch between premaxilla and maxilla early in ontogeny (0) or occludes in a pit between premaxilla and maxilla; no notch early in ontogeny (1). (*Brochu, 1997b, ch. 77; adapted from Norell, 1988, ch. 29*)

92. Occlusion of dentary teeth on the maxillae, with dentary teeth occluding lingual to maxillary teeth with no maxillary reception pits (0), partially interlocking dentition with one or two interdental reception pits between maxillary teeth five through to nine (1), or fully interlocking dentition with interdental reception pits from maxillary tooth one to at least tooth nine (2). (*Lee & Yates, 2018, ch. 27, modified*; *adapted from Norell, 1988, ch. 5; Willis, 1993, ch. 1*)

93. Largest maxillary alveolus is 3 (0), 5 (1), 4 (2), 4 and 5 are same size (3), 6 (4), or maxillary teeth homodont (5), or maxillary alveoli gradually increase in diameter posteriorly toward penultimate alveolus (6). (*Brochu & Storrs, 2012, ch. 93; adapted from Norell, 1988, ch. 1*)

94. Maxillary tooth row curved medially or linear (0) or curves laterally broadly (1) posterior to first six maxillary alveoli. (*Brochu, 1997b, ch. 135; adapted from Clark, 1994, ch. 79*)

95. Dorsal surface of rostrum curves smoothly (0) or bears medial dorsal boss (1). (*Brochu, 1997b, ch. 101*)

96. Canthi rostralii absent or very modest (0) or very prominent (1) at maturity. (*Brochu, 1997b, ch. 143; adapted from Norell, 1988, ch. 34*)

97. Preorbital ridges absent or very modest (0) or very prominent (1) at maturity. (*Brochu, 1997b, ch. 144*)

98. Antorbital fenestra present (0) or absent (1). (*Norell & Clark, 1990, ch. 2; Benton & Clark, 1988*)

99. Vomer entirely obscured by premaxilla and maxilla (0) or exposed on palate at premaxillary-maxillary suture (1). (*Brochu, 1997b, ch. 125; adapted from Norell, 1988, ch. 22*)

100. Vomer entirely obscured by maxillae and palatines (0) or exposed on palate between palatines (1). (*Brochu, 1997b, ch. 126*)

101. Surface of maxilla within narial canal imperforate (0) or with a linear array of pits (1). (*Brochu, 1997b, ch. 148, modified*)

102. Medial jugal foramen small (0) or very large (1). (*Brochu, 1997b, ch. 120*)

103. Maxillary foramen for palatine ramus of cranial nerve V small or not present (0) or very large (1). (*Brochu, 1997b, ch. 111*)

104. Ectopterygoid-maxilla suture anteromedially orientated and separated from toothrow margin, not adjacent to any maxillary alveolus (0), or ectopterygoid-maxilla suture anteromedially orientated and adjacent only to the last maxillary alveolus (1), or ectopterygoid-maxilla suture parallel and adjacent to medial toothrow margin for the length of at least two maxillary alveoli (2), or ectopterygoid forming the lingual wall of at least one maxillary alveolus (3). **ORDERED** (*Rio & Mannion, 2021, ch. 175, modified*; *Brochu, 1997b, ch. 91, modified; adapted from Norell, 1988, ch. 19*)

105. Maxilla terminates in palatal view anterior to lower temporal bar (0) or comprises part of the lower temporal bar (1). (*Brochu & Storrs, 2012, ch. 105*)

106. Penultimate maxillary alveolus less than (0) or more than (1) twice the diameter of the last maxillary alveolus. (*Brochu & Storrs, 2012, ch. 106*)

107. Prefrontal dorsal surface smooth adjacent to orbital rim (0) or bearing discrete knob-like processes (1). (*Brochu & Storrs, 2012, ch. 107*)

108. Dorsal half of prefrontal pillar narrow (0) or expanded anteroposteriorly (1). (*Brochu, 1997b, ch. 137; adapted from Norell, 1988, ch. 41*)

109. Medial process of prefrontal pillar expanded dorsoventrally (0) or anteroposteriorly (1). (*Brochu, 1997b, ch. 136*)

110. Prefrontal pillar solid (0) or with large pneumatic recess (1). (*State 1 refers to the prefrontal recess* sensu *Witmer, 1997*; *Brochu, 1999, ch. 99, modified*)

111. Medial process of prefrontal pillar wide (0) or constricted (1) at base. (*Brochu, 1997b, ch. 138*)

112. Shape of the lateral margin of the suborbital fenestra is straight to slightly curved laterally (0) or bowed medially (1). (*Lee & Yates, 2018, ch. 147; Brochu, 1997b, ch. 105, modified*)

113. Anterior face of palatine process rounded or pointed anteriorly (0) or notched anteriorly (1). (*Brochu, 1997b, ch. 108, modified*)

114. Anterior ectopterygoid process tapers to a point (0) or forked (1). (*Brochu, 1997b, ch. 109*)

115. Palatine process extends (0) or does not extend (1) significantly beyond anterior end of suborbital fenestra. (*Brochu, 1997b, ch. 110; adapted from Willis, 1993, ch. 2*)

116. Palatine process generally broad anteriorly (0) or in form of thin wedge (1). (*Brochu, 1997b, ch. 118*)

117. Lateral edges of palatines smooth anteriorly (0) or with lateral process projecting from palatines into suborbital fenestrae (1). (*Brochu, 1997b, ch. 94*)

118. Palatine-pterygoid suture terminates at the posterior limit of the suborbital fenestra (0), or suture lies a short anterior distance up the interfenestral strut so that the pterygoids form a short (less than 20%) section of it (1), or suture lies far anterior up the interfenestral strut so that the pterygoids contribute to at least 20% of the length of the strut (2). **ORDERED**. (*Lee & Yates, 2018, ch. 142, modified; adapted from Brochu, 1997b, ch. 85; the version of this character as originally*

presented by Lee & Yates [2018] had an additional state ["suture terminates medial to the posterior limit of the suborbital fenestra but not appreciably anterior to it"] which is herein removed due to its redundancy with state 1 [=state 2 of ch. 142 in Lee & Yates, 2018])

119. Pterygoid ramus of ectopterygoid straight, posterolateral margin of suborbital fenestra linear (0) or ramus bowed, posterolateral margin of fenestra concave (1). (*Brochu & Storrs, 2012, ch. 119*)

120. Lateral edges of palatines parallel posteriorly (0) or flare posteriorly, producing a 'shelf' (1). (*Brochu, 1997b, ch. 90; adapted from Norell, 1988, ch. 2*)

121. Anterior margin of the secondary choana is comprised of the palatines (0), or choana entirely surrounded by pterygoids (1). (*Brochu, 1997b, ch. 71 modified; adapted from Benton & Clark, 1988; Clark, 1994, ch. 43; Norell & Clark, 1990, ch. 1*)

122. Secondary choana projects posteroventrally (0) or anteroventrally (1) at maturity. (*Brochu, 1997b, ch. 72*)

123. Pterygoid surface lateral and anterior to secondary choana flush with choanal margin (0), or pushed inward anterolateral to choanal aperture (1), or pushed inward around choana to form neck surrounding aperture (2). (*Brochu, 1997b, ch. 73, modified*)

124. Posterior rim of secondary choana not deeply notched (0) or deeply notched (1). (*Brochu, 1997b, ch. 107*)

125. Secondary choana not septate (0), or with septum that remains recessed within choana (1), or with septum that projects out of choana (2). **ORDERED**. (*Brochu, 1997b, ch. 152*)

126. Ectopterygoid-pterygoid flexure disappears during ontogeny (0) or remains throughout ontogeny (1). (*Brochu, 1997b, ch. 116*)

127. Ectopterygoid extends (0) or does not extend (1) to posterior tip of lateral pterygoid flange at maturity. (*Brochu, 1997b, ch. 149; adapted from Norell, 1988, ch. 32*)

128. Lacrimal makes broad contact with nasal; no posterior process of maxilla (0), or maxilla with posterior process within lacrimal (1), or maxilla with posterior process between lacrimal and prefrontal (2). (*Brochu, 1997b, ch. 93*)

129. Prefrontals separated by frontals and nasals (0) or prefrontals meet medially (1). (*Norell, 1988, ch. 27*)

130. Lacrimal longer than prefrontal (0), or prefrontal longer than lacrimal (1), or lacrimal and prefrontal both elongate and nearly the same length (2). (*Brochu, 1997b, ch. 117; Norell, 1988, ch. 7, modified*)

131. Anterior tip of frontal (0) forms simple acute point or (1) forms broad, complex sutural contact with the nasals. (*Brochu & Storrs, 2012, ch. 131*)

132. Ectopterygoid extends along medial face of postorbital bar (0) or stops abruptly ventral to postorbital bar (1). (*Brochu, 1997b, ch. 133*)

133. Postorbital bar massive (0) or slender (1). (*Brochu, 1997b, ch. 70; adapted from Norell, 1989, ch. 3*)

134. Postorbital bar bears a well-developed spine or tubercle that persists into adulthood (0) or has a barely developed or lacks a spine or tubercle (1). (*Lee & Yates, 2018, ch. 67; Brochu, 1997b, ch. 134, modified; adapted from Norell, 1989, ch. 2*)

135. Ventral margin of postorbital bar flush with lateral jugal surface (0) or inset from lateral jugal surface (1). (*Brochu, 1997b, ch. 146; adapted from Benton & Clark, 1988; Norell & Clark, 1990, ch. 3*)

136. Postorbital bar continuous with anterolateral edge of cranial table (0) or inset (1). (*Salisbury* et al.*, 2006, ch. 175, modified; adapted from Norell & Clark, 1989, ch. 3*)

137. Margin of orbit flush with skull surface (0), or dorsal edges of orbits upturned (1), or orbital margin telescoped (2), or non-telescoped orbits, with frontal having broadly convex orbital margins (3). (*Ristevski* et al.*, 2020b, ch. 137; Brochu, 1997b, ch. 103, modified*)

138. Ventral margin of orbit circular (0) or with prominent notch (1). (*Brochu, 1997b, ch. 139*)

139. Palpebral forms from single ossification (0) or from multiple ossifications (1). (*Brochu, 1997b, ch. 96; adapted from Norell, 1988, ch. 8; Clark, 1994, ch. 65*)

140. Quadratojugal spine prominent at maturity (0) or greatly reduced or absent at maturity (1). (*Brochu, 1997b, ch. 69; adapted from Norell, 1989, ch. 1*)

141. Quadratojugal spine low, near posterior angle of infratemporal fenestra (0) or high, between posterior and superior angles of infratemporal fenestra (1). (*Brochu, 1997b, ch. 114*)

142. Quadratojugal forms posterior angle of infratemporal fenestra (0), or quadratojugal-jugal suture lies at posterior angle of infratemporal fenestra (1) or jugal forms posterior angle of

infratemporal fenestra (2). **ORDERED**. (*Brochu, 1997b, ch. 75, modified; adapted from Norell, 1989, ch. 10*)

143. Postorbital neither contacts quadrate nor quadratojugal medially (0), or contacts quadratojugal, but not quadrate, medially (1), or contacts quadrate and quadratojugal at dorsal angle of infratemporal fenestra (2). (*Brochu, 1997b, ch. 76, modified*)

144. Quadratojugal bears long anterior process along lower temporal bar (0) or bears modest process, or none at all, along lower temporal bar (1). (*Brochu, 1997b, ch. 83*)

145. Quadratojugal extends to superior angle of infratemporal fenestra (0) or does not extend to superior angle of infratemporal fenestra; quadrate participates in the infratemporal fenestra (1). (*Brochu, 1997b, ch. 80; adapted from Buscalioni* et al., *1992, ch. 6*)

146. Dorsal and ventral rims of squamosal groove for external ear valve musculature parallel (0) or squamosal groove flares anteriorly (1). (*Brochu, 1997b, ch. 84*)

147. Quadrate and squamosal not in contact on the external surface of the skull, posteriorly to the external auditory meatus (0), or quadratosquamosal suture extends dorsally along posterior margin of the external auditory meatus (1) or extends only to the posteroventral corner of the external auditory meatus (2). (*As modified by Delfino* et al., *2008a from Brochu, 1997b, ch. 132 and Salisbury* et al., *2006, ch. 132*)

148. Posterior margin of meatal chamber not defined and gradually merging into the otoccipital (0), or smooth and continuous with the paraoccipital process (1), or posterior margin of meatal chamber inset (2). **ORDERED**. (*Delfino* et al., *2008a, ch. 102, modified*; *Salisbury* et al., *2006, ch. 102 modified*; *adapted from Brochu, 1997b, ch. 102; meatal chamber* sensu *Montefeltro* et al., *2016*)

149. Frontoparietal suture deeply within supratemporal fenestra; frontal prevents broad contact between postorbital and parietal (0), or suture makes modest entry into supratemporal fenestra at maturity; postorbital and parietal in broad contact (1), or suture on cranial table entirely (2). **ORDERED**. (*Brochu, 1997b, ch. 81*)

150. Frontoparietal suture concavo-convex (0) or linear (1) between supratemporal fenestrae. (*Brochu, 1997b, ch. 86*)

151. Supratemporal fenestra with fossa; dermal bones of cranial table do not overhang rim at maturity (0), or dermal bones of cranial table overhang rim of supratemporal fenestra near maturity (1), or supratemporal fenestra closes during ontogeny (2), or dermal bones of cranial table overhang rim of supratemporal fenestra near maturity; fenestrae large, significantly longer than wide, with an oval shape (3). (*Cidade* et al.*, 2017, ch. 151 modified*; *based on Brochu, 1997b, ch. 87; adapted from Norell, 1988, ch. 9*)

152. Shallow fossa at anteromedial corner of supratemporal fenestra (0) or no such fossa; anteromedial corner of supratemporal fenestra smooth (1). (*Brochu, 1997b, ch. 92*)

153. Medial parietal wall of supratemporal fenestra imperforate (0) or bearing foramina (1). (*Brochu, 1997b, ch. 104; adapted from Norell, 1988, ch. 51*)

154. Parietal and squamosal widely separated by quadrate on posterior wall of supratemporal fenestra (0), or parietal and squamosal approach each other on posterior wall of supratemporal fenestra without actually making contact (1), or parietal and squamosal meet along posterior wall of supratemporal fenestra (2). (*Brochu, 1997b, ch. 131*)

155. Cranial table surface slopes ventrally from sagittal axis (0) or planar (1) at maturity. (*Brochu, 1997b, ch. 123, modified*)

156. Posterolateral margin of squamosal horizontal or nearly so (0) or upturned to form a discrete 'horn' (1). (*Brochu & Storrs, 2012, ch. 157*)

157. Squamosal, posterolateral prongs absent or very short, barely exceeding the level of the posterior wall of the cranial table behind supratemporal fenestrae (0), or long, exceeding the level of the posterior margin of the cranial table, less than half anteroposterior cranial table length (1), or long, greater than or equal to half anteroposterior cranial table length (2). **ORDERED.** (*Rio & Mannion, 2021, ch. 110; Brochu, 1997b, ch. 140, modified*)

158. Squamosal does not extend (0) or extends (1) ventrolaterally to lateral extent of paraoccipital process. (*Brochu, 1997b, ch. 150, modified*)

159. Supraoccipital exposure on dorsal cranial table absent (0), small (1), large (2), or large such that parietal is excluded from posterior edge of the cranial table (3). **ORDERED**. (*Brochu, 1997b, ch. 82, modified; adapted from Norell, 1988, ch. 11*)

160. Sulcus on anterior braincase wall lateral to parabasisphenoid rostrum (0) or braincase wall lateral to parabasisphenoid rostrum smooth; no sulcus (1). (*Brochu, 1997b, ch. 122*)

161. Parabasisphenoid not exposed extensively (0) or exposed extensively (1) on braincase wall anterior to trigeminal foramen. (*Brochu, 1997b, ch. 129, modified; adapted from Norell, 1989, ch. 5*)

162. Extensive exposure of prootic on external braincase wall (0) or prootic largely obscured by quadrate and laterosphenoid externally (1). (*Brochu, 1997b, ch. 74; adapted from Norell, 1989, ch. 5*)

163. Lateral laterosphenoid bridge comprised entirely of laterosphenoid (0) or includes contribution from the ascending process of the pterygoid (1). (*Brochu, 1997b, ch. 115, modified*)

164. Capitate process of laterosphenoid oriented laterally (0) or anteroposteriorly (1) toward midline. (*Brochu, 1997b, ch. 130*)

165. Parietal with recess communicating with pneumatic system (0) or solid, without recess (1). (*Brochu, 1997b, ch. 154, modified*)

166. Significant ventral quadrate process on lateral braincase wall (0) or quadrate-pterygoid suture linear from parabasisphenoid exposure to trigeminal foramen (1). (*Brochu, 1997b, ch. 127, modified*)

167. Posterior carotid foramen opens lateral (0) or dorsal (1) to parabasisphenoid at maturity. (*Brochu, 1997b, ch. 128, modified*)

168. External surface of basioccipital ventral to occipital condyle oriented posteroventrally (0) or posteriorly (1) at maturity. (*Brochu & Storrs, 2012, ch. 170; adapted from Hua & Jouve, 2004, ch. 167 and Salisbury* et al.*, 2006, ch. 174*)

169. Posterior pterygoid processes tall, with long axis oriented dorsoventrally (0) or dorsoventrally short and without a discernable long axis (1). (*Rio & Mannion, 2021, ch. 136; Brochu, 1997b, ch. 98, modified*)

170. Parabasisphenoid thin (0) or anteroposteriorly wide (1) ventral to basioccipital. (*Brochu, 1997b, ch. 113*)

171. Parabasisphenoid not broadly exposed ventral to basioccipital at maturity; pterygoid short ventral to median pharyngeal tube foramen (0) or parabasisphenoid exposed as broad sheet ventral to basioccipital at maturity; pterygoid tall ventral to median pharyngeal tube foramen (1). (*Brochu, 1997b, ch. 119 modified*; *terminology modified in accord with Dufeau & Witmer, 2015 and Young & Bierman, 2019*)

172. Otoccipital with very prominent boss on paroccipital process; process lateral to cranioquadrate opening short (0) or otoccipital with small or no boss on paroccipital process; process lateral to cranioquadrate opening long (1). (*Brochu, 1997b, ch. 141, modified*)

173. Pharyngotympanic foramina open dorsal (0) or lateral (1) to median pharyngeal tube foramen. (*Brochu, 1997b, ch. 147, modified; adapted from Norell, 1988, ch. 46*; *terminology modified in accord with Dufeau & Witmer, 2015 and Young & Bierman, 2019*)

174. Otoccipitals terminate dorsal to basioccipital tuberosities (0), or send robust process ventrally and participate in basioccipital tuberosities (1), or send slender process ventrally to basioccipital tuberosities (2). **ORDERED**. (*Brochu, 1997b, ch. 151, modified; adapted from Norell, 1988, ch. 20; Clark, 1994, ch. 57 and 60*)

175. Quadrate foramen aëreum on mediodorsal angle (0) or on dorsal surface (1) of quadrate. (*Brochu, 1997b, ch. 121*)

176. Quadrate foramen aëreum is small (0), comparatively large (1), or absent (2) at maturity. (*Brochu & Storrs, 2012, ch. 178*)

177. Quadrate lacks (0) or bears (1) prominent, mediolaterally thin crest on dorsal surface of ramus. (*Brochu & Storrs, 2012, ch. 179*)

178. Attachment scar for posterior mandibular adductor muscle on ventral surface of quadrate ramus forms modest crests (0) or prominent knob (1). (*Brochu & Storrs, 2012, ch. 180; adapted from Ősi* et al.*, 2007, ch. 165*)

179. Quadrate condyle, notch on the dorsal articular border is either absent or small, restricted to dorsomedial edge of quadrate articular border (0), or large, as an extensive indentation of the dorsal articular border, covering up to a third of the mediolateral width of the quadrate condyle (1), or inset from dorsomedial edge of the condyle (2). (*Rio & Mannion, 2021, ch. 118; Brochu, 1997b, ch. 112, modified*)

180. Iris (0) greenish/yellowish or (1) brown. (*Brochu & Storrs, 2012, ch. 182*)

181. Fewer than eight (0), or eight to 14 (1), or more than 14 (2) paired midline scale rows (*Brochu & Storrs, 2012, ch.184*; *there is considerable variation in this character within species, and further work is needed to clarify the situation; data from Fuchs, 2006*)

182. Ectopterygoid maxillary ramus forms less than (0) or more than (1) two-thirds of lateral margin of suborbital fenestra. (*Brochu & Storrs, 2012, ch. 185*)

183. Ectopterygoid maxillary ramus terminates at lateral margin of suborbital fenestra (0) or lateral to it, with maxilla separating the ectopterygoid from fenestra for short distance (1). (*Brochu & Storrs, 2012, ch. 186*)

184. Palatine-maxillary suture intersects suborbital fenestra at its anteromedial margin (0) or nearly at its anterior-most limit (1). (*Brochu & Storrs, 2012, ch. 187*)

185. Frontal lacks (0) or bears (1) prominent midsagittal crest between orbits. (*Brochu & Storrs, 2012, ch. 188*)

186. All cervical neural spines anteroposteriorly broad (0) or posterior neural spines thin and rod-like (1). (*Brochu & Storrs, 2012, ch. 189; adapted from Clark, 1994, ch. 90, Pol* et al.*, 2009, ch. 90*)

187. Largest premaxillary tooth is the second (0), the third (1), or the fourth (2), or the third and fourth are largest (3), or all similar in size (4), or the fourth and fifth are largest (5), or the first four equal in size (6). (*Massonne* et al.*, 2019, ch. 190; based on Wang* et al.*, 2016, ch. 190*)

188. Dorsal surface of the surangular, lateral to the glenoid fossa is smooth (0), or bears a single pit (1), or bears two pits (2), or bears a large elongated sulcus next to the anterior half of the glenoid fossa (3), or intensely ornamented as the lateral surface of the surangular (4). (*Based on Wang* et al.*, 2016, ch. 191 and Lee & Yates, 2018, ch. 204*)

189. 'U'-shaped depression of the frontal at the point of maximum constriction between the orbits absent (0) or present (1). (*Cossette & Brochu, 2018, ch. 107, modified*)

190. Skull in lateral view relatively flat (0) or wedge-shaped (1). (*Massonne* et al.*, 2019, ch. 193*)

191. Nasal bone does (0) or does not (1) reach to the height of the orbit. (*Massonne* et al.*, 2019, ch. 194*)

192. Anterior process of jugal extends anterior (0), lies at the same level as (1), or well posterior to the anterior process of frontal (2). (*Massonne* et al.*, 2019, ch. 195; modified from Jouve, 2004, ch. 177; Jouve* et al.*, 2008, ch. 174 and Jouve, 2016, ch. 174*)

193. Notch between the premaxilla and maxilla present (0), or absent (1) in adult individuals. (*Massonne* et al.*, 2019, ch. 196*)

194. Sutural contact of the otoccipitals dorsal to foramen magnum (0) long, at least half the height of the foramen magnum, (1) short, shorter than half the height of the foramen magnum, or (2) no sutural contact between the otoccipitals. (*Massonne* et al.*, 2019, ch. 197, modified*)

195. Anterior maxillary teeth without (0) or with (1) ridges on their labial surface. (*Massonne* et al.*, 2019, ch. 198, modified*)

196. If largest dentary alveolus is between 11th and 14th and a series behind it, is it the (0) 11th, (1) 12th, or (2) 13th to 14th. (*Massonne* et al.*, 2019, ch. 200; applicable only to taxa that are scored with state 1 for character 51 [see Massonne* et al.*, 2019]*)

197. Surangular-angular suture lingually originates (0) near the ventral border of the external mandibular fenestra, (1) near the dorsal border of the external mandibular fenestra and straight, (2) near the dorsal border of the external mandibular fenestra and bowed. (*Massonne* et al.*, 2019, ch. 201*)

198. Large or very large supraoccipital exposure on cranial table is (0) trapezoid, (1) triangular, or (2) block-shaped. (*Massonne* et al.*, 2019, ch. 202, modified; applicable only to taxa that have large or very large supraoccipital exposure on cranial table, i.e., applicable to taxa that are scored with either state 2 or 3 for character 159 [see Massonne* et al.*, 2019]*)

199. Anterior profile of the premaxilla in lateral view is shallowly sloping (0) or steeply angled so that it is close to vertical (1). (*Lee & Yates, 2018, ch. 1*)

200. Alveolar process of the maxilla along the first six maxillary alveoli is inconspicuous or low, with medial lamina comprising no more than 10% of total rostral height (0), or moderately developed, with medial lamina comprising between 10–25% of total rostral height (1), or tall, with medial lamina comprising 25% or more of total rostral height (2). **ORDERED**. (*Adapted from Salisbury & Willis, 1996, ch. 16 and Lee & Yates, 2018, ch. 26*)

201. Anterior margins of suborbital fenestrae set to the level of 9th maxillary alveoli or posterior (0), or between the 9th and 7th maxillary alveoli (1), or to the level of 7th maxillary alveoli or anterior (2). (*Modified from Salisbury & Willis, 1996, ch. 14 and Lee & Yates, 2018, ch. 144*)

202. Jugal-lacrimal contact expressed by a long suture that widely separates the maxilla from the orbital margin (0), or point contact that narrowly separates the maxilla from the orbital margin (1), or no jugal-lacrimal contact, with the maxilla contributing to the orbital margin (2). **ORDERED**. (*Lee & Yates, 2018, ch. 38, modified; based on information in Willis [1997, 2001]*)

203. Orientation of the ventral ornamented portion of the jugal, anterior to the postorbital bar is lateral to ventrolateral (0) or sharply bent to face ventrally (1). (*Lee & Yates, 2018, ch. 62, modified*)

204. Cross-sectional shape of the anterior ramus of the ectopterygoid subtriangular (0) or a dorsoventrally compressed plate projecting medially from the maxilla (1). (*Lee & Yates, 2018, ch. 153, modified*)

205. Outline of supratemporal fenestra at maturity is sub-square (0), circular to sub-circular (1) sub-rectangular, longer anteroposteriorly (2), sub-rectangular, longer mediolaterally (3), elliptical, with the long axis aligned parasagittaly (4), or D-shaped (5). (*Reformulated from Lee & Yates, 2018, ch. 75; adapted in part from de Andrade* et al.*, 2011, ch. 111*)

206. Minimum width between supratemporal fenestrae with respect to maximum cranial table width is 10% or less of total width (0), or between 10–20% of total width (1), or at least 20% of total width (2). (*Tennant* et al., *2016, ch. 126, modified; based on information in Schwarz & Salisbury, 2005*)

207. The bar forming the posterior border of the supratemporal fenestra (measured as the minimum distance between the posterior margin of the supratemporal fenestra and the occipital margin of the cranial table) is robust, with the minimum thickness being greater than 18% of the width of the cranial table at the level of the postorbital-squamosal suture (0), or the minimum thickness is 8–18 % of the width of the cranial table (1), or slender, with the minimum thickness being less than 8% of the width of the cranial table (2). (*Adapted from Lee & Yates, 2018, ch. 95*)

208. Anteromedial extension of the frontoparietal fossa onto the dorsal cranial roof not present at any stage of ontogeny (0) or present in all but the most aged adults (1). (*Lee & Yates, 2018, ch. 73, modified; frontoparietal fossa* sensu *Holliday* et al., *2020*)

209. Large sculpture pits on cranial table developed anterior to the supratemporal fenestrae absent (0), or present and spread over the frontal-parietal junction, with minimal or no involvement of the postorbital (1), or present and spread over the postorbital-frontal-parietal triple junction (2). (*Lee & Yates, 2018, ch. 71, modified; based on information in Willis* et al., *1993*)

210. Ventral extent of the dorsal ornamentation into the lateral squamosal attachment area for the upper earlid musculature extends no further than the dorsal margin of the attachment area (0) or rugose ornamentation that extends into the lateral squamosal sulcus so that only the ventrolateral rim remains smooth (1). (*Lee & Yates, 2018, ch. 82, modified*)

211. Angle of posteroventral descent of the subdermal portion of the squamosal at its posterolateral corner is shallow, about 45° or less (0) or steep and close to vertical (1). (*Lee & Yates, 2018, ch. 88*)

212. The pterygoid process of the quadrate has minimal or no occipital exposure (0) or evident occipital exposure (1) ventrolateral to the otoccipital. (*Lee & Yates, 2018, ch. 116, modified*)

213. Distance between the posterior tip of the paroccipital process and the quadrate condylar surface is less than the width of the quadrate condylar surface (0) or greater than the width of the quadrate condylar surface (1). (*Lee & Yates, 2018, ch. 111, modified; based on information in Molnar, 1982; distance measured from the posterior tip of the paroccipital process to the medial quadrate hemicondyle*)

214. Quadrate condyles and occipital condyle aligned on the same plane (0) or unaligned, quadrate condyles are at a lower level than the occipital condyle (1). (*Adapted from de Andrade* et al., *2011, ch. 4; based on Wu & Sues, 1996, ch. 24*)

215. Anteriorly directed ridges on the pterygoid plates extending from the lateral margins of the secondary choana absent (0) or present (1). (*Lee & Yates, 2018, ch.161*)

216. Shape of the secondary choana is circular or elliptical (0), or sub-triangular, tapering posteriorly (1), or sub-rectangular, with the long axis oriented mediolaterally (2), or sub-triangular, tapering anteriorly (3). (*Rio & Mannion, 2021, ch. 190, modified; Lee & Yates, 2018, ch. 163, modified; original version of the character from Jouve* et al., *2015, ch. 236*)

217. Shape of the ventrolateral margin of the dentary is smoothly rounded (0) or curved at approximately a 90˚ angle, resulting in a horizontal ventral surface and a vertical lateral surface (1). (*Lee & Yates, 2018, ch. 181, modified*)

218. Ventral margin of the ornamented region of the angular simple, not everted (0) or everted into a laterally to ventrolaterally projecting flange (1). (*Lee & Yates, 2018, ch. 199*)

219. Laterally directed flange along the dorsolateral margin of the surangular, bordering the ornamented region is absent (0) or present (1). (*Lee & Yates, 2018, ch. 203; Pol & Norell, 2004, ch. 187, modified*)

220. Maxillary teeth/alveoli size disparity at adulthood low, largest maxillary tooth/alveolus is less than twice the diameter of the smallest interfestoonal tooth/alveolus (0) or high, diameter of the largest tooth/alveolus more than twice that of the smallest interfestoonal tooth/alveolus (1). (*Modified from Salisbury & Willis, 1996, ch. 17 and Lee & Yates, 2018, ch. 220*)

221. Maxillary alveolar count is 11 or fewer (0), 12–16 (1), 17–20 (2), 21–28 (3), 29 or more (4) alveoli. **ORDERED**. (*Young, 2014, ch. 156*)

222. Dentary alveolar count is 14 or fewer (0), 15–19 (1), 20–29 (2), 30 or more (3) alveoli per rami. **ORDERED**. (*Young, 2014, ch. 158, modified*)

223. Alveolar processes of premaxillae at the first two alveoli are straight (0) or arched (1). (*Ristevski* et al., *2020b, ch. 226*)

224. Laterally directed flange along the dorsolateral margin of the dentary, labial to the last four to five dentary alveoli is absent (0) or present (1). (*Based on Lee & Yates, 2018, ch. 179*)

225. Postoccipital processes of the supraoccipital are small to moderately large (0), or very large and widely spaced (1), or very large and closely spaced (2). (*Ristevski* et al., *2021, ch. 229*)

226. Rostrum length in dorsal view, ratio of anteroposterior rostral length (measured from level of anterior orbital margin, to anterior-most point of rostrum), to total skull length (measured from posterior-most level of quadrate condyle to anterior-most point of rostrum) is ≤ 0.5 (0) or > 0.5 (1). (*Rio & Mannion, 2021, ch. 1; based on Wu & Sues, 1996, ch. 4 and Groh* et al., *2020, ch. 16*)

227. Rostral depth, ratio of maximum dorsoventral height of the maxilla to mediolateral width of the maxilla at the 5th maxillary alveolus is < 0.5 (0) or ≥ 0.5 (1). (*Rio & Mannion, 2021, ch. 5*)

228. Interorbital distance, ratio of minimum mediolateral width between orbits to maximum mediolateral width across anterior cranial table is < 0.5 (0) or ≥ 0.5 (1). (*Rio & Mannion, 2021, ch. 6; based on Jouve, 2004, ch. 181 and Groh* et al., *2020, ch. 26*)

229. Supratemporal fenestra size, ratio of maximum anteroposterior supratemporal fenestra length to anteroposterior cranial table length (measured from the level of the frontal-postorbital suture) is < 0.5 (0) or ≥ 0.5 (1). (*Rio & Mannion, 2021, ch. 10; based on Wu* et al., *2001b, ch. 67 and Groh* et al., *2020, ch. 36*)

230. Supratemporal fenestra shape, ratio of maximum mediolateral width to maximum anteroposterior length is ≤ 1 (0) or > 1 (1). (*Rio & Mannion, 2021, ch. 11; based on Jouve* et al., *2008, ch. 199*)

231. Width to length ratio of the cranial table in mature individuals is ≥ 1.6 (0), between 1.6 and 1.3 (1), or ≤ 1.3 (2). (*Lee & Yates, 2018, ch. 94, modified*)

232. Basioccipital tuberosities, ratio of maximum mediolateral width of basioccipital tuberosities to maximum mediolateral width of the occipital condyle is < 2 (0) or ≥ 2 (1). (*Rio & Mannion, 2021, ch. 16*)

233. External mandibular fenestra, ratio of anteroposterior length (between anterior and posterior limits) to dorsoventral height (between dorsal and ventral limits) is < 2.5 (0) or ≥ 2.5 (1). (*Rio & Mannion, 2021, ch. 18; Montefeltro* et al.*, 2013, ch. 306, modified*)

234. External mandibular fenestra, minimum angle subtended by dorsal margin of fenestra and the horizontal is < 25˚ (0) or ≥ 25˚ (1). (*Rio & Mannion, 2021, ch. 19; de Andrade* et al.*, 2011, ch. 314, modified)*

235. Articular, retroarticular process ratio of anteroposterior length (measured from the transverse ridge to the posterior-most tip of articular) to the mediolateral width across the glenoid fossa is < 1.5 (0) or ≥ 1.5 (1). (*Rio & Mannion, 2021, ch. 20; Lee & Yates, 2018, ch. 217, modified*)

236. Transverse ridge between the orbits ('spectacle') absent (0) or present (1). (*Lee & Yates, 2018, ch. 56*)

237. First three premaxillary teeth are evenly spaced (0) or second tooth much closer to third than to first (1). (*Jouve* et al.*, 2015, ch. 224 and Lee & Yates, 2018, ch. 12, modified*)

238. Premaxilla, position of the penultimate premaxillary alveolus is posterolateral or in the same line (0) or posteromedial (1) relative to the antepenultimate alveolus. (*Rio & Mannion, 2021, ch. 143, modified*)

239. Last premaxillary tooth/alveolus is set posterolateral to the penultimate premaxillary tooth/alveolus (0), or last premaxillary tooth/alveolus is set in line with penultimate (1), or last premaxillary tooth/alveolus is set posteromedial to penultimate premaxillary tooth/alveolus (2). **ORDERED**. (*Lee & Yates, 2018, ch. 16, modified; Jouve* et al.*, 2015, ch. 204, modified*)

240. Level of the anterior margin of the orbit is posterior to or in line with the 12th maxillary tooth (0) or anterior to the 12th maxillary tooth (1). (*Lee & Yates, 2018, ch. 60, modified; Jouve* et al., *2015, ch. 186, modified*)

241. Edentulous posterior extension of the maxilla is absent or shorter than the length of three posterior alveoli (0) or long and extending for a distance equal to or longer the length of three posterior alveoli (1). (*Lee & Yates, 2018, ch. 31; Jouve* et al., *2015, ch. 172, modified*)

242. Extent of ectopterygoid and maxilla on medial surface of jugal, anterior to the postorbital bar is minimal, jugal visible (0) or extensive, covering medial surface of jugal (1). (*Rio & Mannion, 2021, ch. 182, modified*)

243. Parietal lacks (0) or bears (1) a prominent midsagittal crest between supratemporal fenestrae. (*Rio & Mannion, 2021, ch. 83, modified; based on Clark, 1994, ch. 33 and Pol* et al., *2009, ch. 33*)

244. Depth of the lateral squamosal attachment area for the ear flap musculature is deeply incised and with a well-defined sulcus (0), or a flat bevelled surface with at most a weakly defined shallow depression (1). (*Lee & Yates, ch. 81*)

245. Quadrate condyle shape, dorsal and ventral margins are subparallel across length (subrectangular condyle) (0), or medially tapering (1), or constricted at mid-length (2), or ventrally reflected medial hemicondyle (3). (*Rio & Mannion, 2021, ch. 119; based on Brochu, 1997b, ch. 112*)

246. Basioccipital, the lateral margins of ventral basioccipital plate are parallel or ventrally convergent (0) or ventrally divergent (1). (*Rio & Mannion, 2021, ch. 129; based on Jouve, 2004, ch. 176*)

247. Basioccipital, concavity on ventral margin, posterior to median pharyngeal tube foramen present (0) or absent (1). (*Rio & Mannion, 2021, ch. 133, modified; based on Jouve, 2004, ch. 198 and Jouve* et al., *2008, ch. 187*)

248. Choanae, position of anterior margin is anterior to the posterior margin of suborbital fenestra (0), or level with the posterior margin of suborbital fenestra (1), or posterior to the posterior margin of suborbital fenestra (2). **ORDERED**. (*Rio & Mannion, 2021, ch. 188; based on Clark, 1994, ch. 44, Pol & Norell, 2004, ch. 44, and Pol* et al., *2009, ch. 44*)

249. Choanae, position of posterior margin relative to posterior edge of pterygoid flange is anterior to or at the same level as the posterior edge of pterygoid flange (0) or posterior to posterior edge of pterygoid flange (1). (*Rio & Mannion, 2021, ch. 189; based on Jouve, 2016, ch. 209*)

250. Secondary choanae, anterior margin shape is linear or curved (0) or invaginated (1). (*Rio & Mannion, 2021, ch. 191*)

251. Pterygoid, bulbous differentiated bullae (at maturity) absent (0) or present (1). (*Rio & Mannion, 2021, ch. 199; Lee & Yates, 2018, ch. 158, modified*)

252. Outline of dorsal margin of splenial is straight, anterodorsally inclined (0) or concave, abruptly dorsally inclined at posterior end (1). (*Rio & Mannion, 2021, ch. 232, modified; Lee & Yates, 2018, ch. 191, modified*)

253. Ventral margin of the splenial is narrow and unornamented (0) or a mediolaterally broad and with ornamented surface (1). (*Lee & Yates, 2018, ch. 189 modified*)

254. Articular, dorsal extent of retroarticular process is at the same level or ventral to posterior edge of articular fossa (0) or dorsal to posterior edge of articular fossa (1). (*Rio & Mannion, 2021, ch. 251; based on Jouve, 2004, ch. 190*)

255. Position of first premaxillary alveolus is medial to the second and approximately in line (0) or first premaxillary alveolus is positioned anteromedially to second premaxillary alveolus (1). **NEW**

256. Anterior process of the frontal short, comprising no more than 35% of the total anteroposterior length of the frontal (0), or moderately long, being 35-60% the total anteroposterior length of the frontal (1), or very long, comprising over 60% of the total anteroposterior length of the frontal (2). **NEW, ORDERED**

257. Occipital lamina of supraoccipital bears a nuchal crest that is laterally bounded by concavities (0), or occipital lamina has flat and unornamented surface that bears a nuchal crest (1), or has a flat surface that is unornamented and lacks a nuchal crest (2), or has a flat surface that lacks a nuchal crest and is sculptured with sub-circular pits (3), or has a broad convex surface that lacks a nuchal crest (4), or occipital lamina of the supraoccipital proportionately narrow, with an acutely developed nuchal crest that strongly projects posteriorly (5). **NEW** (*Based on personal observations; formulation for state 5 of this character is after Rio & Mannion, 2021, ch. 80*)

EXPLANATIONS FOR SCORING THE NEW CHARACTERS

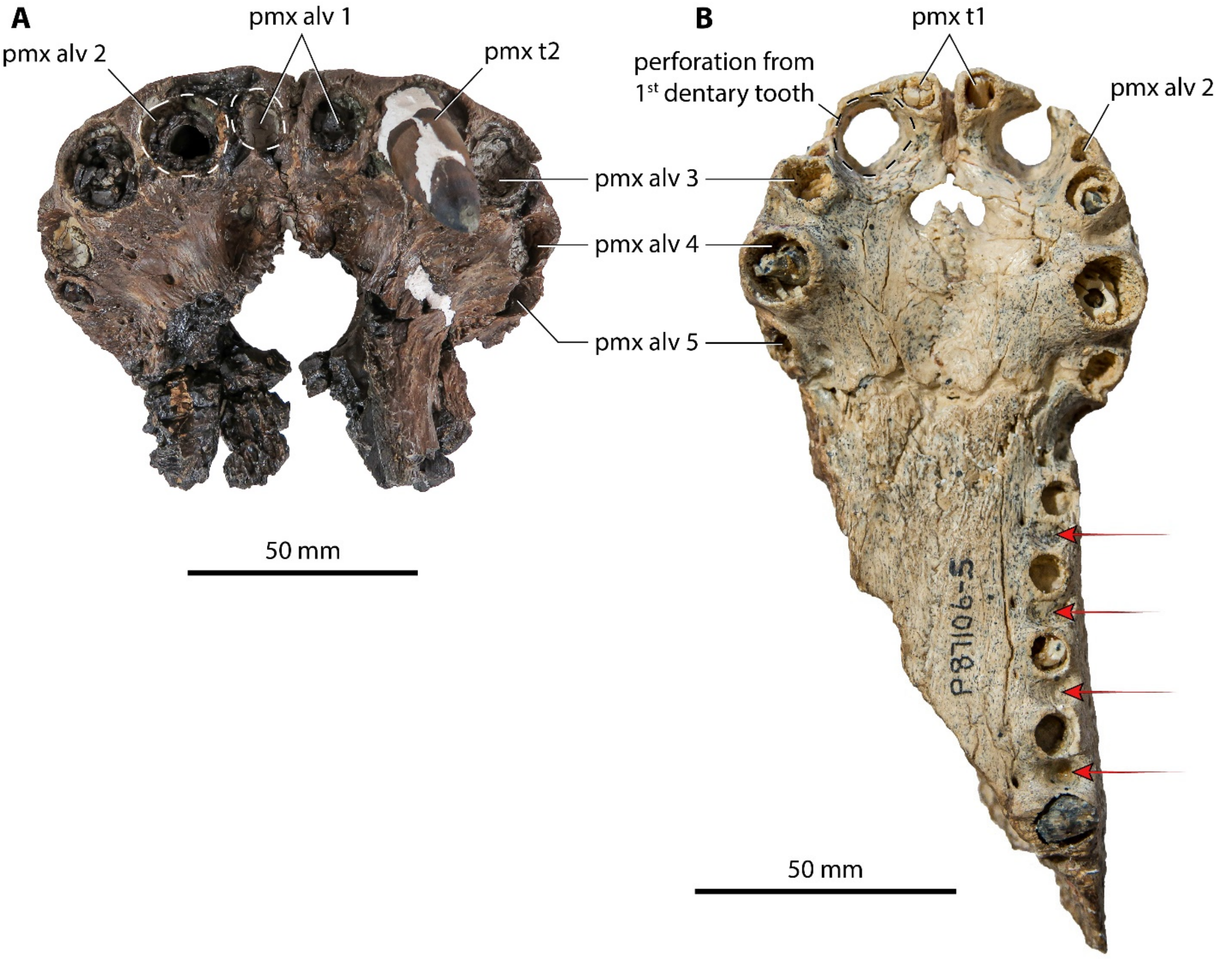

Figure S3.2 Explanation for scoring character 255 in the list. (A) *Anteophthalmosuchus epikrator* Ristevski *et al.*, 2018, IWCMS 2001.446, holotype, isolated premaxillae in ventral view. (B) *Harpacochampsa camfieldensis* Megirian *et al.*, 1991, NTM P87106-5, partial rostrum in ventral view. Notice how in (A), the first two premaxillary alveoli are positioned in line with each other (state 0), whereas in (B) the first premaxillary alveoli are notably anteromedial to the second (state 1). The red arrows in (B) indicate the pits on the maxilla for reception of dentary teeth. For a high-resolution version of this figure, see the PDF file of **Figure S3.2** provided as a supplementary file. Abbreviations: **pmx alv**, premaxillary alveolus; **pmx t**, premaxillary tooth.

Character 255: In some taxa (e.g., *Anteophthalmosuchus epikrator*, *Brachychampsa montana*, *Dongnanosuchus hsui*, *Paludirex vincenti*; **Fig. S3.2A**), the first two premaxillary teeth/alveoli are positioned almost in line with each other, where the first premaxillary alveolus is, at most,

positioned only subtly more anteriorly relative to the second (state 0). Most other taxa display a different configuration of the first two premaxillary teeth/alveoli, where the first premaxillary tooth/alveolus is positioned anteromedially relative to the second (state 1; **Fig. S3.2B**).

Character 256: In crocodylians, the anterior process of the frontal may have a different proportional length relative to the total length of the element. Most taxa have anterior processes of the frontal that are 35–60% of the total length of the element (state 1). However, few taxa exhibit lengths of the anterior processes that are no more than 35% the total length of the frontal (state 0). This condition is recognized in few taxa included in the matrix, such as *Trilophosuchus rackhami* (**Figs. 1A**, **2A**, **7A**, **S3.3A** and **S3.3B**), *Mekosuchus sanderi* and *Mekosuchus whitehunterensis* (**S1.14A** and **S1.14B**; see figure S1.11D in Ristevski *et al*., 2020b). On the other extreme, several taxa have extremely elongated anterior processes of the frontals (state 2). An example of a taxon that has an anterior process of the frontal that is extremely elongated is *Kalthifrons aurivellensis* (**Fig. S3.3C** and **S3.3D**).

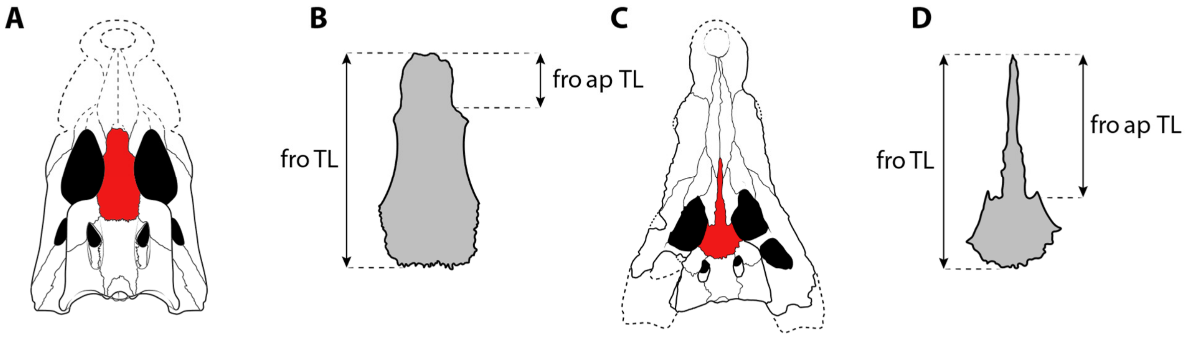

Figure S3.3 Illustrations showing the differences between proportionately short (state 0 of character 256) and extremely long (state 2 of character 256) anterior processes of the frontal in crocodylians. (A) Drawing of the cranium of *Trilophosuchus rackhami* Willis, 1993, based on QMF16856, with the frontal highlighted in red. (B) Drawing of the isolated frontal of *Trilophosuchus rackhami*, based on QMF16856 and QMF16857. (C) Drawing of the cranium of *Kalthifrons aurivellensis* Yates & Pledge, 2016, based on figures 3B and 5 of Yates & Pledge (2016), with the frontal highlighted in red. (D) Drawing of the isolated frontal of *Kalthifrons aurivellensis*, based on figure 3B of Yates & Pledge (2016). Dashed lines in (A) and (C) indicate hypothetical outlines of missing portions. Skull and frontal illustrations not to scale. For a high-resolution version of this figure, see the PDF file of **Figure S3.3** provided as a supplementary file. Abbreviations: **fro TL**, total length of the frontal; **fro ap TL**, total length of the anterior process of the frontal.

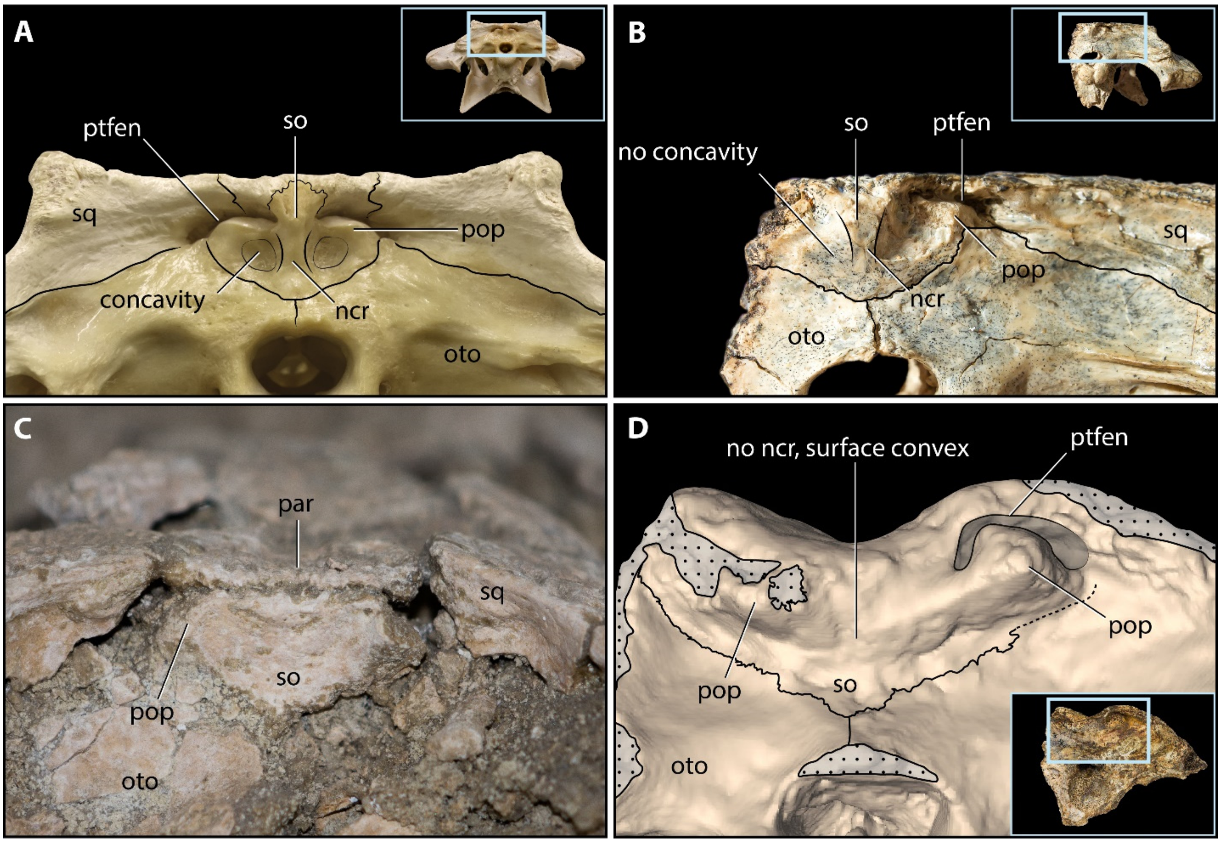

Figure S3.4 Explanation for scoring some of the states for character 257 in the list. Crocodylian skulls in posterior views, focusing on the occipital lamina of the supraoccipital. (A) *Crocodylus porosus* Schneider, 1801, QMJ48127, skull in posterior view. Notice how the nuchal crest of the supraoccipital is laterally bounded by concavities. (B) *Harpacochampsa camfieldensis* Megirian *et al*., 1991, NTM P87106-1, holotype, skull in posterior view. Notice the lack of concavities lateral to the nuchal crest of the supraoccipital. (C) *Australosuchus clarkae* Willis & Molnar, 1991, QMF16788, holotype, skull in posterior view. Notice the lack of concavities and a nuchal crest on the flat occipital lamina of the supraoccipital. (D) *Gunggamarandu maunala* Ristevski *et al*., 2021, QMF548 (=QMF14.548, old registry number), holotype, digital model of the skull in posterior view. Notice the convex occipital lamina of the supraoccipital that lacks concavities and a nuchal crest. Images in (A) and (D) modified from figure S2.1 in Ristevski *et al*. (2021). Skulls not to scale. For a high-resolution version of this figure, see the PDF file of **Figure S3.4** provided as a supplementary file. Abbreviations: **ncr**, nuchal crest; **oto**, otoccipital; **par**, parietal; **pop**, postoccipital process of the supraoccipital; **ptfen**, posttemporal fenestra; **so**, supraoccipital; **sq**, squamosal.

Character 257: In most crocodylians, the occipital lamina (=posterior surface) of the supraoccipital bears a nuchal crest that is laterally bound by a concavity on each side (state 0; **Fig.**

S3.4A). However, the nuchal crest (if present) is not always bound by concavities (state 1). This morphology is recognized in several taxa, including *Harpacochampsa camfieldensis* (**Fig. S3.4B**) and *Paludirex vincenti* (see figure 22 in Ristevski *et al.*, 2020a as well as the interactive 3D PDF in Ristevski *et al.*, 2020b). In others, the occipital lamina of the supraoccipital displays a simple morphology, where it is essentially flat and devoid of a nuchal crest or any concavities (state 2). A flat occipital lamina of the supraoccipital that is lacking a nuchal crest and concavities occurs in *Australosuchus clarkae* and *Trilophosuchus rackhami* (**Figs. 17D**, **S1.5** and **S3.4C**). Similar to *A. clarkae* and *T. rackhami*, the occipital lamina of the supraoccipital of *Mekosuchus sanderi* is also lacking a nuchal crest or concavities. However, unique to *M. sanderi* among currently known taxa is an occipital lamina of the supraoccipital that is ornamented with prominent sub-circular pits like the rest of the cranial table (state 3; **Fig. S1.6**). In *A. clarkae* and *T. rackhami*, the occipital lamina of the supraoccipital is unornamented (even though in *T. rackhami* the occipital lamina has a wrinkled texture, it is not ornamented with pits like in *M. sanderi*). Another peculiar condition is captured in state 4 of this character, which is unique to *Gunggamarandu maunala*. In *G. maunala*, the unornamented occipital lamina of the supraoccipital is deeply convex, lacking a nuchal crest or concavities (**Fig. S3.4D**). The final state formulated for this character, state 5, is exclusive to gavialids like *Gavialis gangeticus*, *Gavialis lewisi*, *Gryposuchus colombianus*, and *Piscogavialis jugaliperforatus*. In these gavialids, the occipital lamina of the supraoccipital is proportionally narrower than in other crocodylians and possesses an acute nuchal crest that projects posteriorly so that it is visible when the skull is observed from a dorsal aspect.

RESULTS FROM THE PHYLOGENETIC ANALYSES

Table S3.1 Summary of the phylogenetic results performed in this study. Abbreviations: **CI**, consistency index; **EW**, equal weighting; **IW**, implied weighting; **#MPCs**, number of most parsimonious cladograms; **RI**, retention index.

Search option	Weighting	*k* value (if IW)	Steps	#MPCs	CI	RI
Traditional	EW	-	1822	10	0.200	0.689
Traditional	IW	5	111.31639	1	0.196	0.682
Traditional	IW	12	69.94380	2	0.197	0.684
Traditional	IW	25	42.35981	3	0.199	0.688
New Technology	EW	-	1822	371	0.200	0.689
New Technology	IW	5	111.31639	1	0.196	0.682
New Technology	IW	12	69.94303	2	0.197	0.684
New Technology	IW	25	42.35363	1	0.199	0.688

Table S3.2 Character states identified as autapomorphies for *Trilophosuchus rackhami* Willis, 1993 based on the phylogenetic analyses. The 'Analysis' column indicates which analysis identified the corresponding character state as an autapomorphy. Abbreviations: **NTS, EW**, analysis performed under the New Technology search option and used the equal weighting method; **NTS, IW**, analysis performed under the New Technology search option and used the implied weighting method; **TrS, EW**, analysis performed under the Traditional search option and used the equal weighting method; **TrS, IW**, analysis performed under the Traditional search option and used the implied weighting method; **k**, concavity constant value of either 5, 12 or 25 for the implied weighting method.

Analysis	Character	State
TrS, EW; TrS, IW k = 5; NTS, EW; NTS, IW k = 5	92	1
TrS, EW; TrS, IW k = 5; TrS, IW k = 12; TrS, IW k = 25; NTS, EW; NTS, IW k = 5; NTS, IW k = 12; NTS, IW k = 25	103	1
TrS, EW; TrS, IW k = 5; TrS, IW k = 12; TrS, IW k = 25; NTS, EW; NTS, IW k = 5; NTS, IW k = 12; NTS, IW k = 25	147	2
TrS, EW; TrS, IW k = 5; TrS, IW k = 12; TrS, IW k = 25; NTS, EW; NTS, IW k = 5; NTS, IW k = 12; NTS, IW k = 25	150	1
TrS, IW k = 12; TrS, IW k = 25; NTS, IW k = 12; NTS, IW k = 25	153	1
TrS, EW; TrS, IW k = 5; TrS, IW k = 12; TrS, IW k = 25; NTS, EW; NTS, IW k = 5; NTS, IW k = 12; NTS, IW k = 25	155	0
TrS, EW; TrS, IW k = 5; TrS, IW k = 12; TrS, IW k = 25; NTS, EW; NTS, IW k = 5; NTS, IW k = 12; NTS, IW k = 25	191	0
TrS, EW; TrS IW k = 5; TrS, IW k = 12; TrS, IW k = 25; NTS, EW; NTS, IW k=5; NTS, IW k = 12; NTS, IW k = 25	200	1
TrS, EW; TrS, IW k = 12; TrS IW k = 25; NTS, EW; NTS, IW k = 12; NTS, IW k = 25	210	1
TrS, EW; TrS, IW k = 5; TrS, IW k = 12; TrS, IW k = 25; NTS, EW; NTS, IW k = 5; NTS, IW k = 12; NTS, IW k = 25	229	1
TrS, EW; TrS, IW k = 5; TrS, IW k = 12; TrS, IW k = 25; NTS, EW; NTS, IW k = 5; NTS, IW k = 12; NTS, IW k = 25	231	2

TrS, EW analysis – strict consensus topology of 10 MPCs

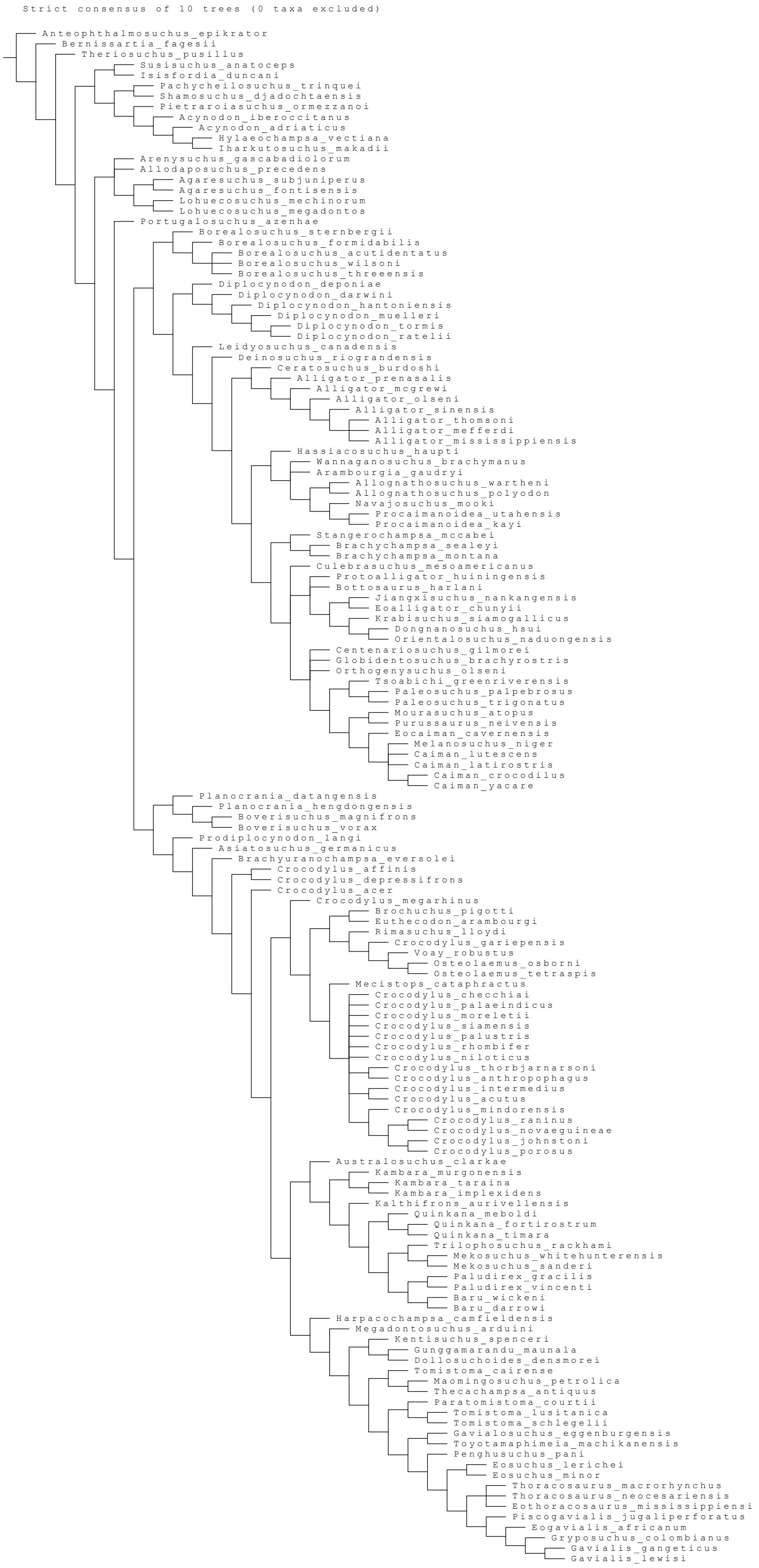

TrS, IW k = 5 analysis – strict consensus topology of 1 MPC

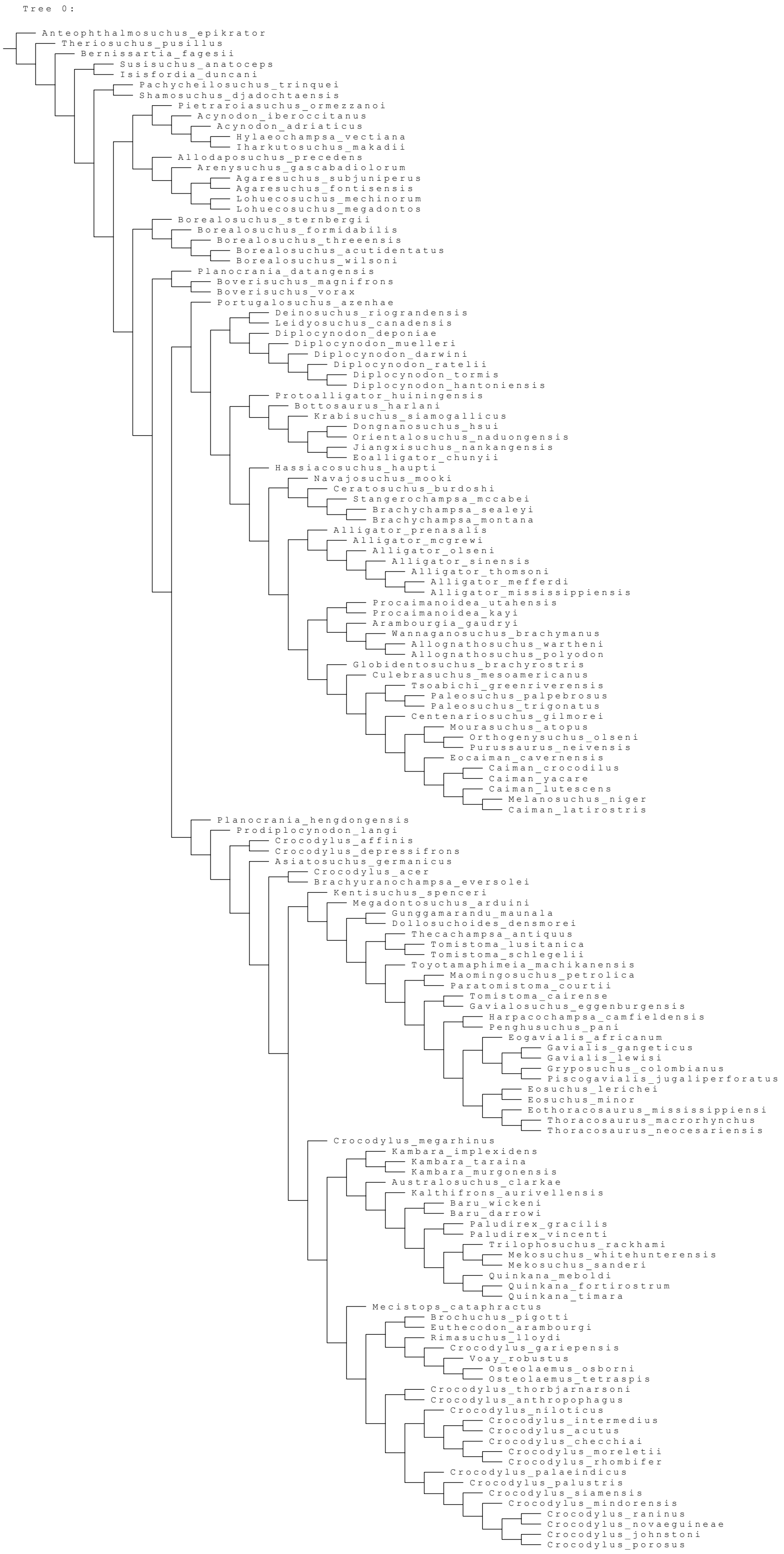

TrS, IW k = 12 analysis – strict consensus topology of 2 MPCs

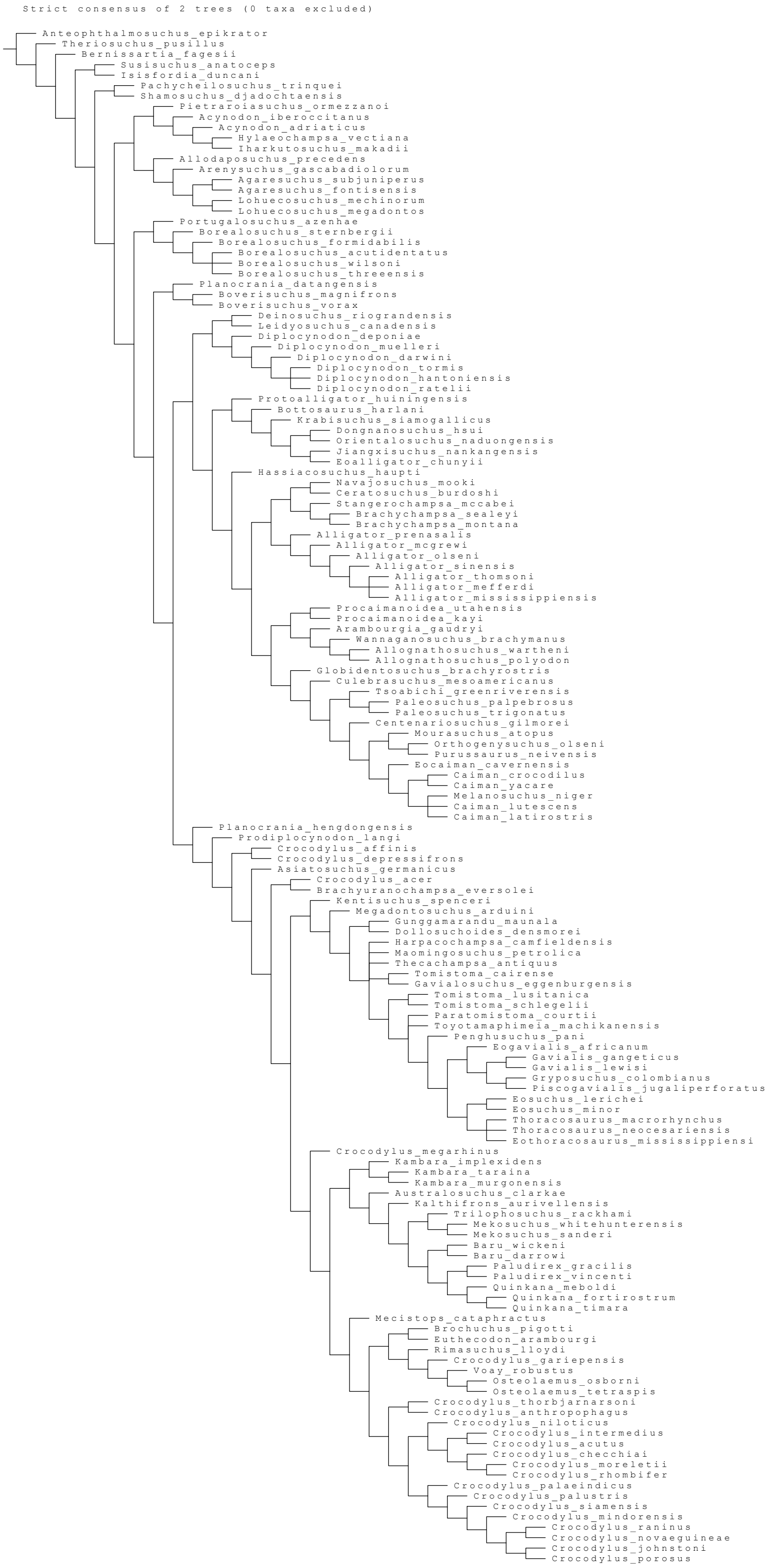

TrS, IW k = 25 analysis – strict consensus topology of 3 MPCs

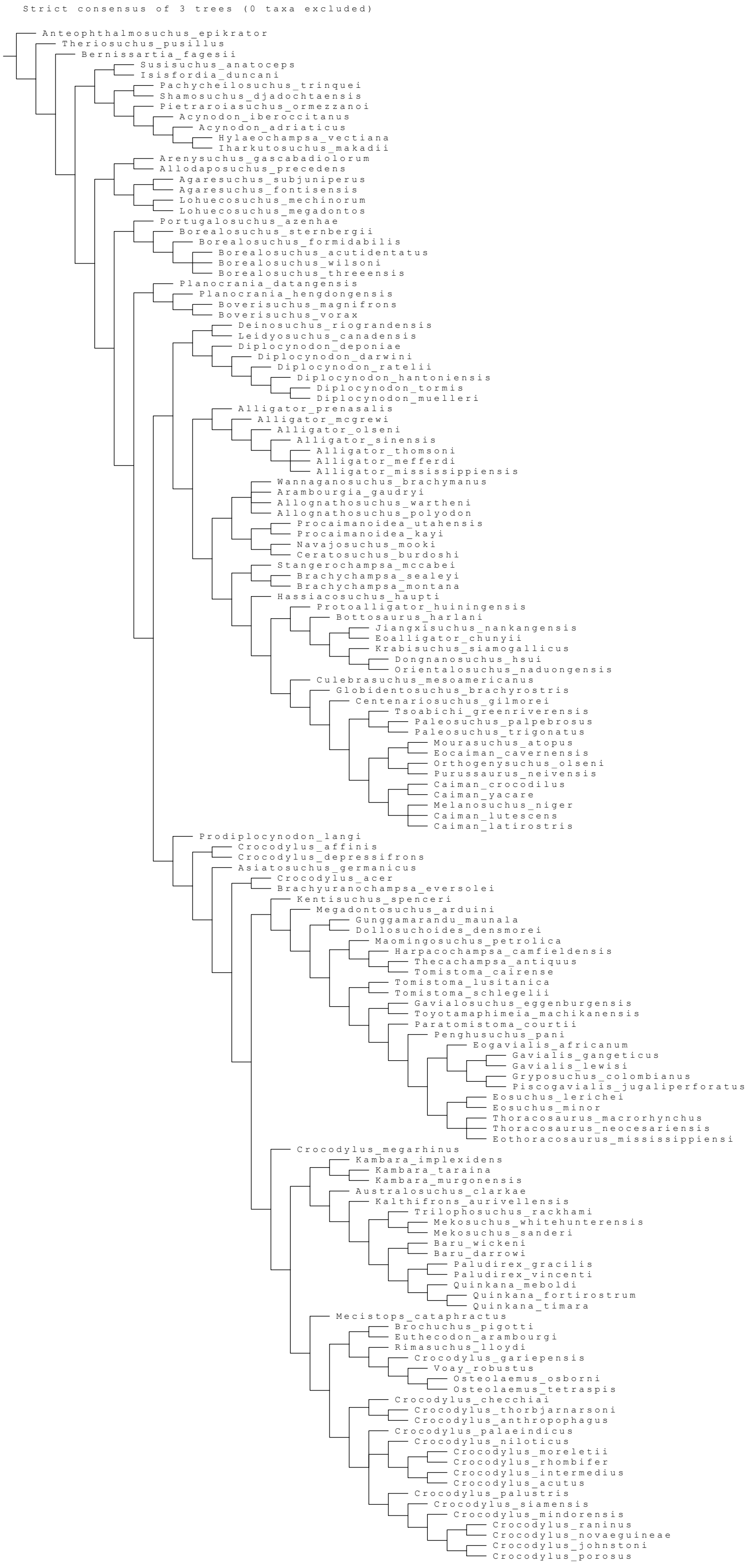

NTS, EW analysis – strict consensus topology of 371 MPCs

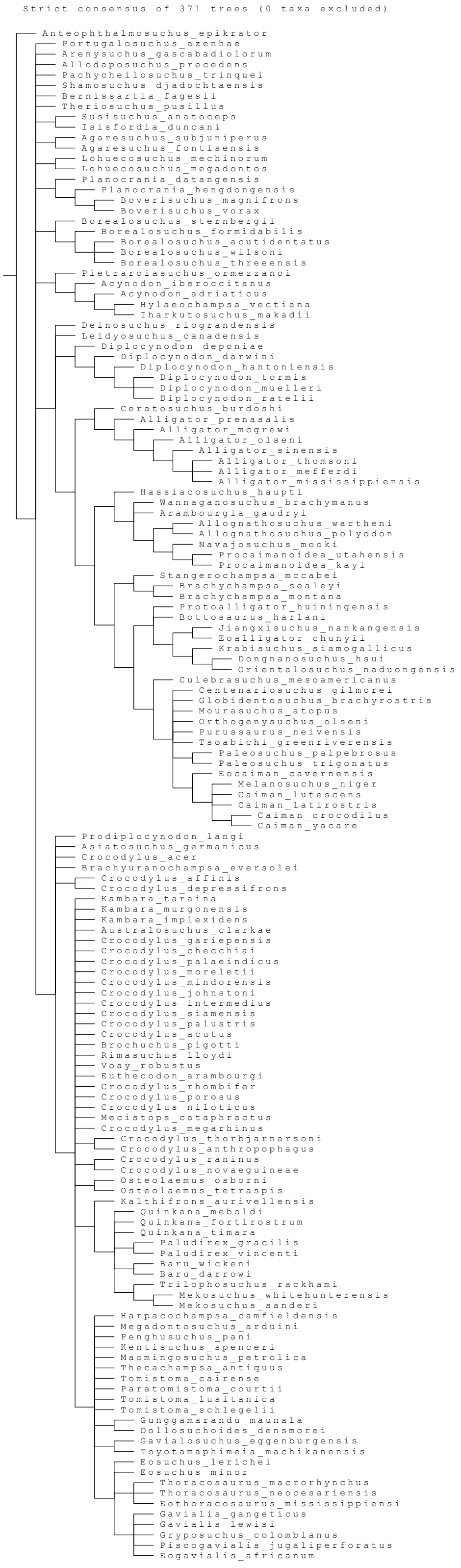

NTS, EW analysis – 50% majority-rule consensus topology

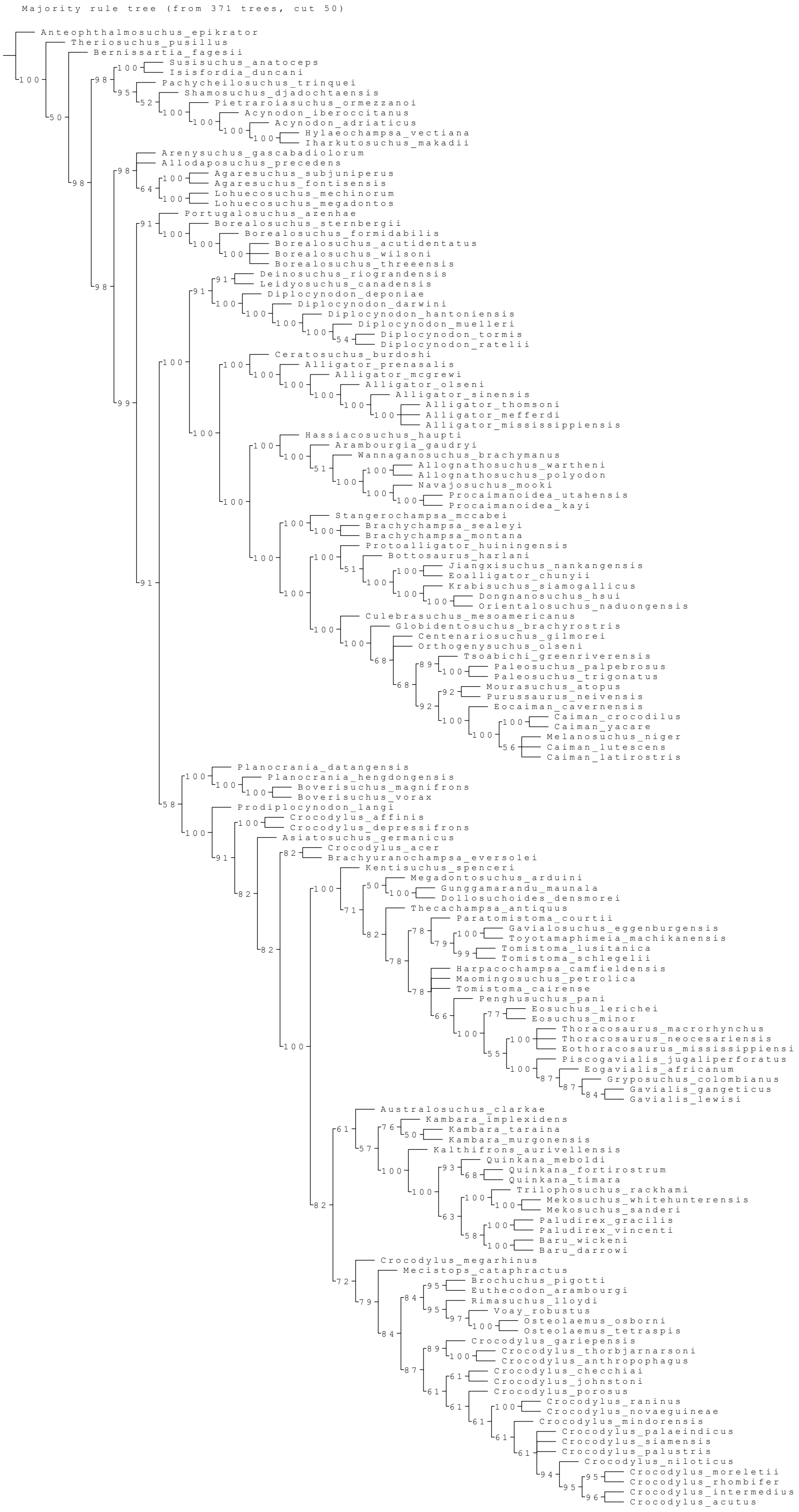

NTS, IW k = 5 analysis – strict consensus topology of 1 MPC

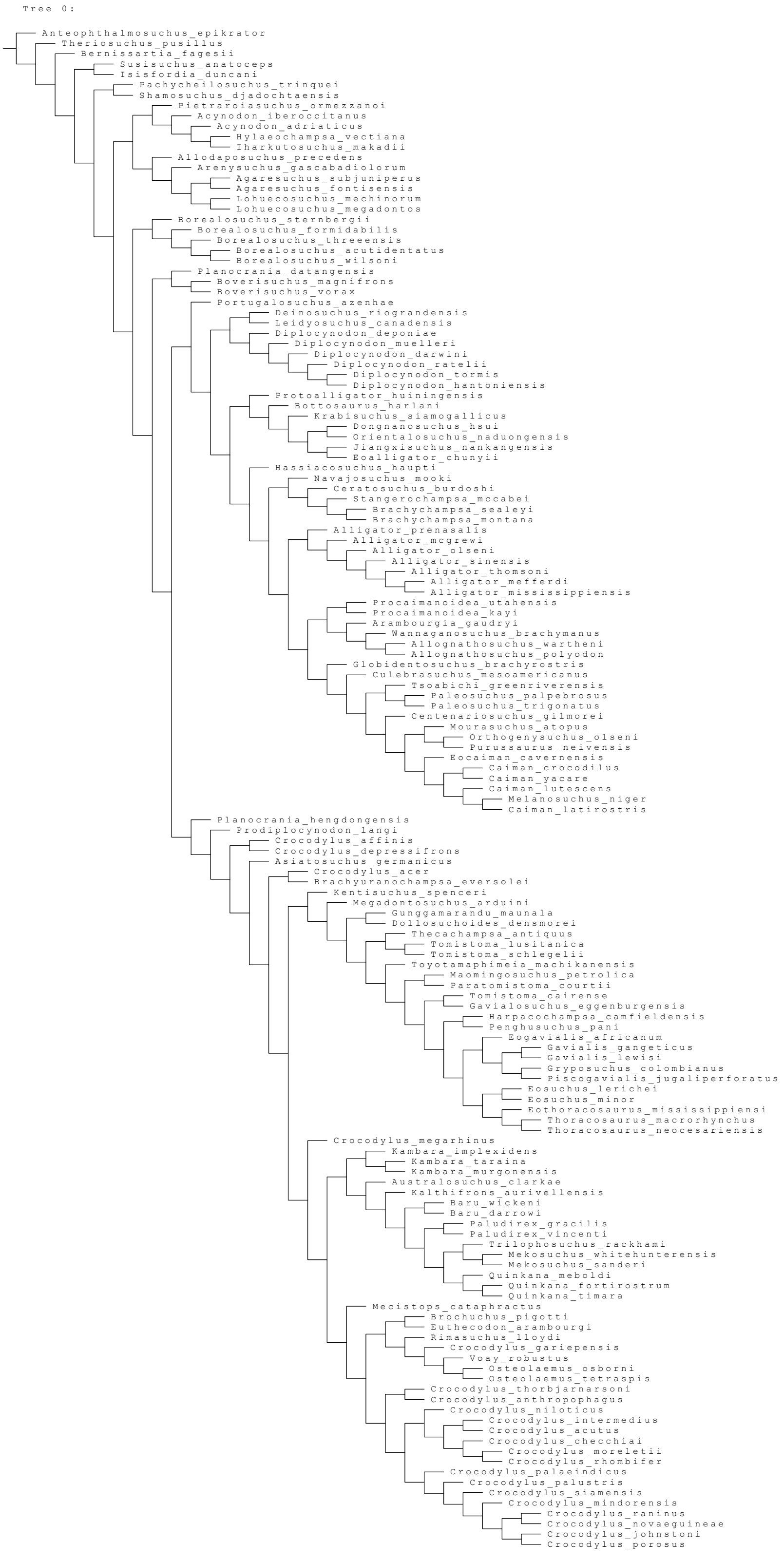

NTS, IW k = 12 analysis – strict consensus topology of 2 MPCs

Strict consensus of 2 trees (0 taxa excluded)

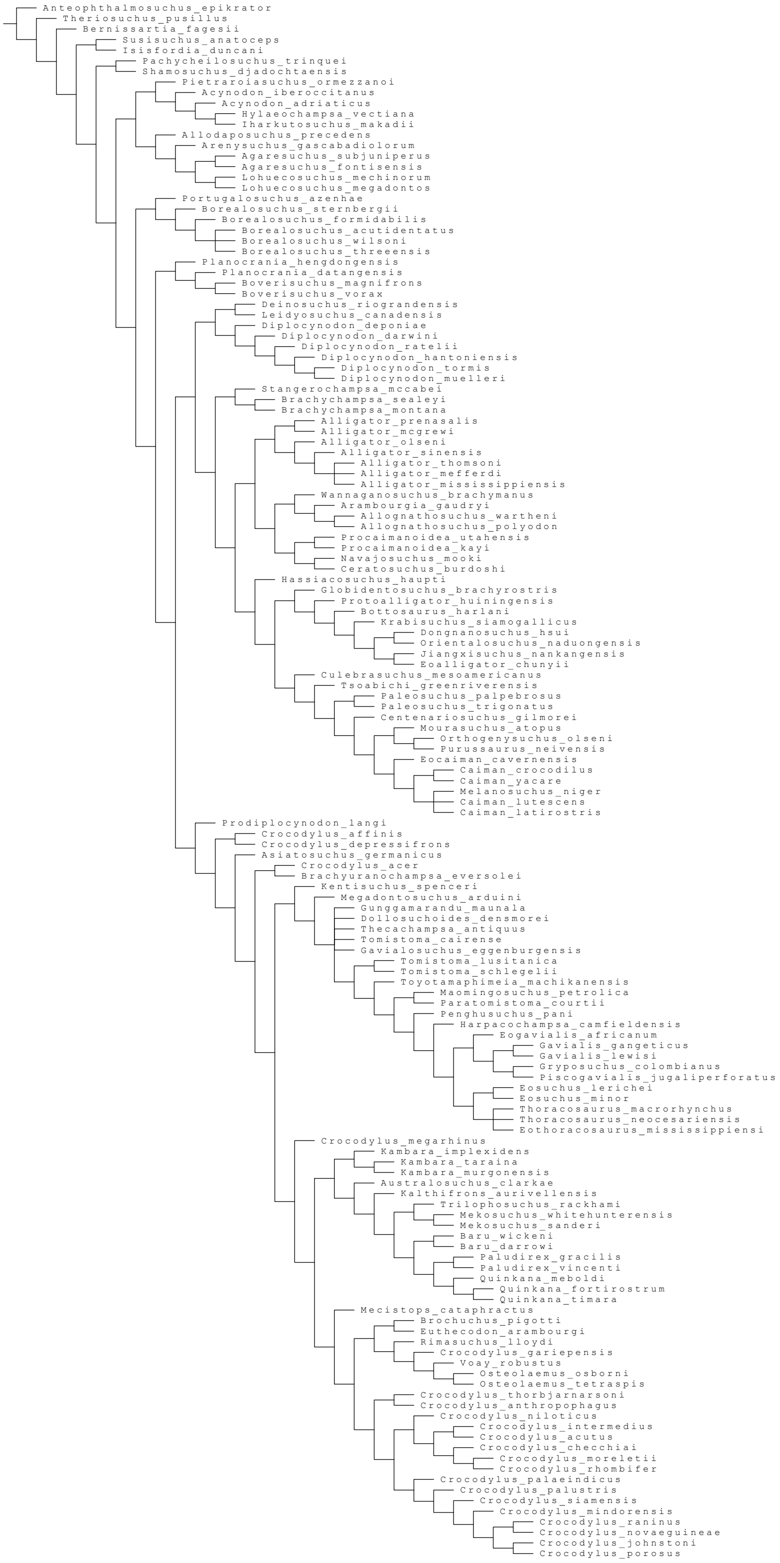

NTS, IW k = 25 analysis – strict consensus topology of 1 MPCs

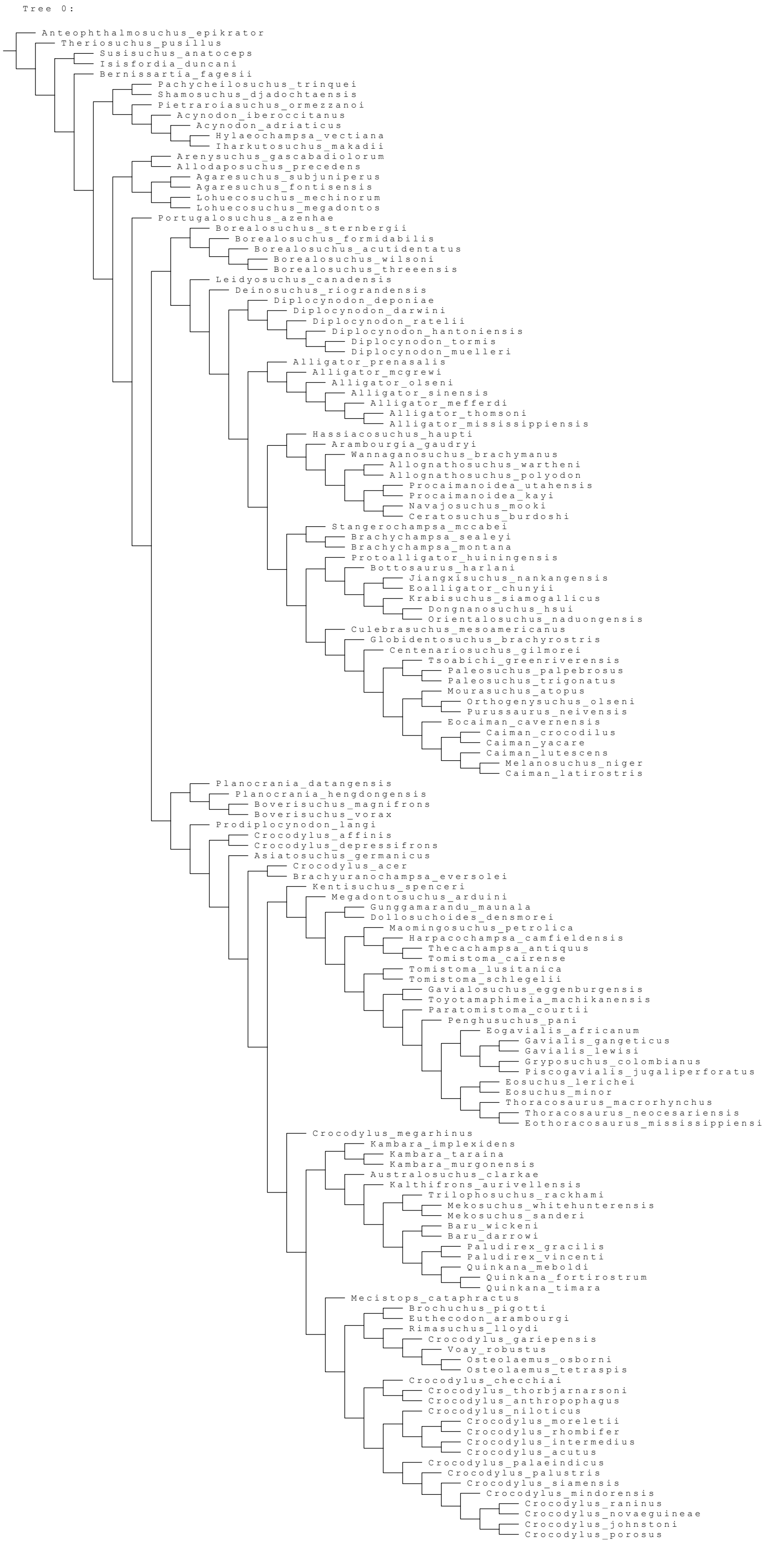

SCORED CHARACTERS FOR THE AUSTRALIAN CROCODYLIFORM OPERATIONAL TAXONOMIC UNITS

Below is a table showing the number and percentages of scored characters for the Australian crocodyliform OTUs in the matrix. The taxa are listed in alphabetical order.

Taxon	Characters scored (out of 257)	Characters scored (in %)
Australosuchus clarkae	181	70.43%
Baru darrowi	124	48.25%
Baru wickeni	162	63.04%
Crocodylus johnstoni	257	100%
Crocodylus porosus	257	100%
Gunggamarandu maunala	26	10.12%
Harpacochampsa camfieldensis	102	39.7%
Isisfordia duncani	166	64.6%
Kalthifrons aurivellensis	78	30.35%
Kambara implexidens	189	73.54%
Kambara murgonensis	118	45.91%
Kambara taraina	139	54.1%
Mekosuchus sanderi	83	32.3%
Mekosuchus whitehunterensis	52	20.23%
Paludirex gracilis	16	6.23%
Paludirex vincenti	96	37.35%
Quinkana fortirostrum	43	16.73%
Quinkana meboldi	31	12.06%
Quinkana timara	104	40.47%
Trilophosuchus rackhami	110	42.8%

INSTITUTIONAL ABBREVIATIONS

AM, Australian Museum, Sydney, New South Wales, Australia (F, fossil)

CMC, Chinchilla Museum Collection, Chinchilla, Queensland, Australia

FMNH, Field Museum of Natural History, Chicago, Illinois, U. S. A.

IRSNB, Institut Royal des Sciences Naturelles de Belgique, Brussels, Belgium

IWCMS, Isle of Wight County Museums Services (Dinosaur Isle Museum and visitor attraction) Sandown, England, United Kingdom

KNM, National Museums of Kenya, Nairobi, Kenya

MGPD, Museo di Geologia e Paleontologia, Università degli Studi di Padova, Padua, Italy

NHMUK, Natural History Museum, London, England, United Kingdom (OR, old register; R, reptiles)

NMB, National Museum of the Bahamas, Nassau, Commonwealth of the Bahamas

NMV, Museum Victoria, Melbourne, Victoria, Australia

NTM, Museum and Art Gallery of the Northern Territory, Darwin and Alice Springs, Northern Territory, Australia (P, palaeontology; R, reptile collection)

QM, Queensland Museum, Brisbane, Queensland, Australia (F, fossil)

SAM, South Australian Museum, Adelaide, South Australia, Australia (P, palaeontology)

TMM, Texas Memorial Museum, Austin, Texas, U. S. A.

UF, University of Florida Museum of Natural History, Gainesville, Florida, U. S. A. (H, herpetology division)

UMZC, University Museum of Zoology, Cambridge, England, United Kingdom

UQ, University of Queensland, Brisbane, Queensland, Australia

UQSSAL, University of Queensland Steven Salisbury collection, Brisbane, Queensland, Australia

REFERENCES

Aguilera, O. A., Riff, D., & Bocquentin-Villanueva, J. (2006). A new giant *Purussaurus* (Crocodyliformes, Alligatoridae) from the Upper Miocene Urumaco Formation, Venezuela. *Journal of Systematic Palaeontology*, *4*(3), 221–232.

de Andrade, M. B., Edmonds, R., Benton, M. J., & Schouten, R. (2011). A new Berriasian species of *Goniopholis* (Mesoeucrocodylia, Neosuchia) from England, and a review of the genus. *Zoological Journal of the Linnean Society*, *163*, S66–S108.

Andrews, C. W. (1901). II.—Preliminary note on some recently discovered extinct vertebrates from Egypt. (Part II.). *Geological Magazine*, *8*(10), 436–444.

Andrews, C. W. (1905). I.—Notes on some new Crocodilia from the Eocene of Egypt. *Geological Magazine*, *2*(11), 481–484.

Antunes, M. T. (1961). *Tomistoma lusitanica*, crocodilian du Miocène du Portugal. *Revista de Faculdade de Ciencias, Universidade de Lisboa*, *9*(*ser. 2*), 5–88.

Azzarà, B., Boschian, G., Brochu, C. A., Delfino, M., Iurino, D. A., Kimambo, J. S., Manzi, G., Masao, F. T., Menconero, S., Njau, J. K., & Cherin, M. (2021). A new cranium of *Crocodylus anthropophagus* from Olduvai Gorge, northern Tanzania. *Rivista Italiana di Paleontologia e Stratigrafia*, *127*(2), 275–295.

Bartels, W. S. (1984). Osteology and systematic affinities of the horned alligator *Ceratosuchus* (Reptilia, Crocodilia). *Journal of Paleontology*, *58*(6), 1347–1353.

Benton, M. J., & Clark, J. M. (1988). Archosaur phylogeny and the relationships of the Crocodylia. Pp. 295–338 in M. Benton (ed.) *The phylogeny and classification of the tetrapods*, *Volume 1*, Clarendon Press, Oxford, United Kingdom.

Berg, D. E. (1966). Die Krokodile, insbesondere *Asiatosuchus* und aff. *Sebecus*?, aus dem Eozän von Messel bei Darmstadt/Hessen. *Abhandlungen des Hessischen Landesamtes für Bodenforschung*, *52*, 1–105.

Bickelmann, C., & Klein, N. (2009). The late Pleistocene horned crocodile *Voay robustus* (Grandidier & Vaillant, 1872) from Madagascar in the Museum für Naturkunde Berlin. *Fossil Record*, *12*(1), 13–21.

de Blainville, H. D. (1835). Systeme d'Herpetologie. *Nouvelles Annalesdu Museum d'Histoire Naturelle*, *4*, 37–295.

de Blainville, H. D. (1855). *Ostéographie: atlas du genre* Crocodilus. *Explication des planches*. Paris.

Bona, P., & Desojo, J. B. (2011). Osteology and cranial musculature of *Caiman latirostris* (Crocodylia: Alligatoridae). *Journal of Morphology*, *272*(7), 780–795.

Bona, P., Fernandez Blanco, M. V., Scheyer, T. M., & Foth, C. (2017). Shedding light on the taxonomic diversity of the South American Miocene caimans: the status of *Melanosuchus fisheri* (Crocodylia, Alligatoroidea). *Ameghiniana*, *54*(6), 681–687.

Bourke, J. M., Fontenot, N., & Holliday, C. (2021). Septal deviation in the nose of the longest faced crocodylian: A description of nasal anatomy and airflow in the Indian gharial (*Gavialis gangeticus*) with comments on acoustics. *The Anatomical Record*, 1–21. DOI: 10.1002/ar.24831.

Brochu, C. A. (1997a). A review of "*Leidyosuchus*" (Crocodyliformes, Eusuchia) from the Cretaceous through Eocene of North America. *Journal of Vertebrate Paleontology*, *17*(4), 679–697.

Brochu, C. A. (1997b). Morphology, fossils, divergence timing, and the phylogenetic relationships of *Gavialis*. *Systematic Biology*, *46*(3), 479–522.

Brochu, C. A. (1997c). *Phylogenetic systematics and taxonomy of Crocodylia*. Unpublished PhD thesis, The University of Texas at Austin, Austin, 467 pp.

Brochu, C. A. (1999). Phylogenetics, taxonomy, and historical biogeography of Alligatoroidea. *Journal of Vertebrate Paleontology*, *19*(S2), 9–100.

Brochu, C. A. (2004a). A new Late Cretaceous gavialoid crocodylian from eastern North America and the phylogenetic relationships of thoracosaurs. *Journal of Vertebrate Paleontology*, *24*(3), 610–633.

Brochu, C. A. (2004b). Alligatorine phylogeny and the status of *Allognathosuchus* Mook, 1921. *Journal of Vertebrate Paleontology*, *24*(4), 857–873.

Brochu, C. A. (2006a). A new miniature horned crocodile from the Quaternary of Aldabra Atoll, Western Indian Ocean. *Copeia*, *2006*(2), 149–158.

Brochu, C. A. (2006b). Osteology and phylogenetic significance of *Eosuchus minor* (Marsh, 1870) new combination, a longirostrine crocodylian from the late Paleocene of North America. *Journal of Paleontology*, *80*(1), 162–186.

Brochu, C. A. (2007a). Morphology, relationships, and biogeographical significance of an extinct horned crocodile (Crocodylia, Crocodylidae) from the Quaternary of Madagascar. *Zoological Journal of the Linnean Society*, *150*, 835–863.

Brochu, C. A. (2007b). Systematics and taxonomy of Eocene tomistomine crocodylians from Britain and northern Europe. *Palaeontology*, *50*(4), 917–928.

Brochu, C. A. (2010). New alligatorid from the lower Eocene Green River Formation of Wyoming and the origin of caimans. *Journal of Vertebrate Paleontology*, *30*(4), 1109–1126.

Brochu, C. A. (2011). Phylogenetic relationships of *Necrosuchus ionensis* Simpson, 1937 and the early history of caimanines. *Zoological Journal of the Linnean society*, *163*(suppl_1), S228–S256.

Brochu, C. A. (2012). Phylogenetic relationships of Palaeogene ziphodont eusuchians and the status of *Pristichampsus* Gervais, 1853. *Earth and Environmental Science Transactions of the Royal Society of Edinburgh*, *103*(3–4), 521–550.

Brochu, C. A. (2020). Pliocene crocodiles from Kanapoi, Turkana Basin, Kenya. *Journal of Human Evolution*, *140*, 102410.

Brochu, C. A., & Gingerich, P. D. (2000). New tomistomine crocodylian from the middle Eocene (Bartonian) of Wadi Hitan, Fayum Province, Egypt. *Contributions from the Museum of Paleontology, the University of Michigan*, *30*(10), 251–268.

Brochu, C. A., & Storrs, G. W. (2012). A giant crocodile from the Plio-Pleistocene of Kenya, the phylogenetic relationships of Neogene African crocodylines, and the antiquity of *Crocodylus* in Africa. *Journal of Vertebrate Paleontology*, *32*(3), 587–602.

Brochu, C. A., Njau, J., Blumenschine, R. J., & Densmore, L. D. (2010). A new horned crocodile from the Plio-Pleistocene hominid sites at Olduvai Gorge, Tanzania. *PLoS ONE*, *5*(2), e9333.

Brochu, C. A., Parris, D. C., Grandstaff, B. S., Denton Jr, R. K., & Gallagher, W. B. (2012). A new species of *Borealosuchus* (Crocodyliformes, Eusuchia) from the Late Cretaceous–Early Paleogene of New Jersey. *Journal of Vertebrate Paleontology*, *32*(1), 105–116.

Buchanan, L. A. (2009). *Kambara taraina* sp. nov. (Crocodylia, Crocodyloidea), a new Eocene mekosuchine from Queensland, Australia, and a revision of the genus. *Journal of Vertebrate Paleontology*, *29*(2), 473–486.

Buckland, W. (1836). *Geology and Mineralogy Considered with Reference to Natural Theology*. Pickering, London, 618 pp.

Buffetaut, E. (1975). Sur l'anatomie et la position systématique de *Bernissartia fagesii* Dollo, L., 1883, crocodilien du Wealdien de Bernissart, Belgique. *Bulletin de l'Institut Royal des Sciences Naturelles de Belgique*, *51*(2), 1–20.

Busbey, A. B. III. (1977). *Functional morphology of the head of* Pristichampsus vorax *(Crocodilia, Eusuchia) from the Eocene of North America.* Unpublished Master of Arts thesis, The University of Texas at Austin, Austin, 102 pp.

Buscalioni, A. D., Sanz, J. L., & Casanovas, M. L. (1992). A new species of the eusuchian crocodile *Diplocynodon* from the Eocene of Spain. *Neues Jahrbuch für Geologie und Paläontologie, Abhandlungen*, *187*(1), 1–29.

Buscalioni, A. D., Ortega, F., & Vasse, D. (1997). New crocodiles (Eusuchia: Alligatoroidea) from the Upper Cretaceous of southern Europe. *Comptes Rendus de l'Académie des Sciences, Série IIA - Earth & Planetary Sciences, 325*(7), 525–530.

Buscalioni A. D., Piras, P., Vullo, R., Signore, M., & Barbera, C. (2011). Early eusuchia crocodylomorpha from the vertebrate-rich Plattenkalk of Pietraroia (Lower Albian, southern Apennines, Italy). *Zoological Journal of the Linnean Society*, *163*, S199–S227.

Carpenter, K. (1983). *Thoracosaurus neocesariensis* (De Kay, 1842) (Crocodylia: Crocodylidae) from the Late Cretaceous Ripley Formation of Mississippi. *Mississippi Geology*, *4*(1), 1–10.

Case, E. C. (1925). Note on a new species of the Eocene crocodilian *Allognathosuchus*, *A. wartheni*. *Contributions from the Museum of Geology, University of Michigan*, *2*(5), 93–97.

Cidade, G. M., Solórzano, A., Rincón, A. D., Riff, D., & Hsiou, A. S. (2017). A new *Mourasuchus* (Alligatoroidea, Caimaninae) from the late Miocene of Venezuela, the phylogeny of Caimaninae and considerations on the feeding habits of *Mourasuchus*. *PeerJ*, *5*, e3056.

Clark, J. M. (1986). *Phylogenetic relationships of the crocodylomorph archosaurs*. Unpublished PhD thesis, The University of Chicago, Chicago, 556 pp.

Clark, J. M. (1994). Patterns of evolution in Mesozoic Crocodyliformes. Pp. 84–97 in N. C. Fraser & H.-D. Sues (eds.) *In the Shadow of the Dinosaurs: Early Mesozoic Tetrapods*, Cambridge, New York, U. S. A.

Clark, J. M., & Norell, M. A. (1992). The Early Cretaceous crocodylomorph *Hylaeochampsa vectiana* from the Wealden of the Isle of Wight. *American Museum Novitates*, *3032*, 1–19.

Colbert, E. H., & Bird, R. T. (1954). A gigantic crocodile from the Upper Cretaceous beds of Texas. *American Museum Novitates*, *1688*, 1–22.

Cong, L., Hou, L., Wu, X., & Hou, J. (1998). *The Gross Anatomy of* Alligator sinensis *Fauvel*. Science Press, Beijing, China, 388 pp.

Conrad, J. L., Jenkins, K., Lehmann, T., Manthi, F. K., Peppe, D. J., Nightingale, S., Cossette, A., Dunsworth, H. M., Harcourt-Smith, W. E. H., & McNulty, K. P. (2013). New specimens of

‘*Crocodylus*’ *pigotti* (Crocodylidae) from Rusinga Island, Kenya, and generic reallocation of the species. *Journal of Vertebrate Paleontology*, *33*(3), 629–646.

Cope, E. D. (1861). Recent species of Emydosaurian reptiles represented in the Museum of the Academy. *Proceedings of the Academy of Natural Sciences of Philadelphia*, *1860*, 549–551.

Cope, E. D. (1873). On the extinct Vertebrata of the Eocene of Wyoming, observed by the expedition of 1872, with notes on the geology. *Annual Report of the U. S. Geological and Geographic Survey of the Territories*, *6*, 545–649.

Cope, E. D. (1882). The reptiles of the American Eocene. *The American Naturalist*, *16*(12), 979–993.

Cossette, A. P., & Brochu, C. A. (2018). A new specimen of the alligatoroid *Bottosaurus harlani* and the early history of character evolution in alligatorids. *Journal of Vertebrate Paleontology*, *38*(4), 1–22.

Cossette, A. P., & Brochu, C. A. (2020). A systematic review of the giant alligatoroid *Deinosuchus* from the Campanian of North America and its implications for the relationships at the root of Crocodylia. *Journal of Vertebrate Paleontology*, e1767638.

Cuvier, G. L. (1807). Sur les différentes espèces de crocodiles vivans et sur leurs caractères distinctifs. *Annales du Muséum National d’Histoire Naturelle, 10*, 8–86.

Cuvier, G. L. (1824). *Recherches Sur Les Ossemens Fossiles. Vol. 5. 2eme*. G. Dufour & E. d’Ocagne Libraries, Paris, 185 pp.

Das, I., & Charles, J. 2002. New record of a freshwater crocodile from Brunei. *IUCN/SSC Crocodile Specialist Group Newsletter*, *21*, 10–11.

Daudin, F. M. (1802). *Histoire Naturelle, Générale et Particulière des Reptiles; ouvrage faisant suit à l'Histoire naturell générale et particulière, composée par Leclerc de Buffon; et rédigee par C. S. Sonnini, membre de plusieurs sociétés savantes*. Vol. 2. F. Dufart, Paris [1802], 432 pp.

Delfino, M., & De Vos, J. (2010). A revision of the Dubois crocodylians, *Gavialis bengawanicus* and *Crocodylus ossifragus*, from the Pleistocene *Homo erectus* beds of Java. *Journal of Vertebrate Paleontology*, *30*(2), 427–441.

Delfino, M., Piras, P., & Smith, T. (2005). Anatomy and phylogeny of the gavialoid crocodylian *Eosuchus lerichei* from the Paleocene of Europe. *Acta Palaeontologica Polonica*, *50*(3), 565–580.

Delfino, M., Codrea, V., Folie, A., Dica, P., Godefroit, P., & Smith, T. (2008a). A complete skull of *Allodaposuchus precedens* Nopcsa, 1928 (Eusuchia) and a reassessment of the morphology of the taxon based on the Romanian remains. *Journal of Vertebrate Paleontology*, *28*(1), 111–122.

Delfino, M., Martin, J. E., & Buffetaut, E. (2008b). A new species of *Acynodon* (Crocodylia) from the Upper Cretaceous (Santonian–Campanian) of Villaggio del Pescatore, Italy. *Palaeontology*, *51*(5), 1091–1106.

Delfino, M., & Smith, T. (2009). A reassessment of the morphology and taxonomic status of ‘*Crocodylus*’ *depressifrons* Blainville, 1855 (Crocodylia, Crocodyloidea) based on the Early Eocene remains from Belgium. *Zoological Journal of the Linnean Society*, *156*(1), 140–167.

Delfino, M., & Smith, T. (2012). Reappraisal of the morphology and phylogenetic relationships of the middle Eocene alligatoroid *Diplocynodon deponiae* (Frey, Laemmert, and Riess, 1987) based on a three-dimensional specimen. *Journal of Vertebrate Paleontology*, *32*(6), 1358–1369.

Delfino, M., Iurino, D. A., Mercurio, B., Piras, P., Rook, L., & Sardella, R. (2020). Old African fossils provide new evidence for the origin of the American crocodiles. *Scientific Reports*, *10*, 11127.

Díaz Aráez, J. L., Delfino, M., Luján, À. H., Fortuny, J., Bernardini, F., & Alba, D. M. (2017). New remains of *Diplocynodon* (Crocodylia: Diplocynodontidae) from the Early Miocene of the Iberian Peninsula. *Comptes Rendus Palevol*, *16*(1), 12–26.

Dollo, L. (1883). Première note sur les crocodiliens de Bernissart. *Bulletin du Musée Royal d'Histoire Naturelle de Belgique*, *2*, 309–338.

Dollo, L. (1907). Nouvelle note sur les reptiles de l'Eocène inférieur de la Belgique et des régions voisines (*Eosuchus lerichei* et *Eosphargis gigas*). *Bulletin de la Société belge de Géologie, Paleontologie et Hydrologie*, *21*, 81–85.

Drumheller, S. K., Wilberg, E. W., & Sadleir, R. W. (2016). The utility of captive animals in actualistic research: a geometric morphometric exploration of the tooth row of *Alligator mississippiensis* suggesting ecophenotypic influences and functional constraints. *Journal of Morphology*, *277*(7), 866–878.

Dufeau, D. L., & Witmer, L. M. (2015). Ontogeny of the middle-ear air-sinus system in *Alligator mississippiensis* (Archosauria: Crocodylia). *PLoS ONE*, *10*(9), e0137060.

Duméril, M. C., & M. Aug. Duméril (1851). Catalogue méthodique de la collection des reptiles du Muséum d'Histoire Naturelle de Paris. Gide et Baudry, Paris, 224 pp.

Erickson, B. R. (1976). Osteology of the early eusuchian crocodile *Leidyosuchus formidabilis*, sp. nov. *Monographs of the Science Museum of Minnesota (Paleontology)*, *2*, 1–61.

Erickson, B. R. (1982). *Wannaganosuchus*, a new alligator from the Paleocene of North America. *Journal of Paleontology*, *56*(2), 492–506.

Falconer, H. (1859). *Descriptive catalogue of fossil remains of vertebrata from the Sewalik Hills, the Nerbudda, Perim Island, etc. in the Museum of the Asiatic Society of Bengal*. Baptist Mission Press, Calcutta, 261 pp.

Fauvel, A. A. (1879). Alligators in China: their history, description and identification. *Journal of the North-China Branch of the Royal Asiatic Society*, *1*, 1–36.

Figueiredo, R. G., Moreira, J. K. R., Saraiva, A. A. F., & Kellner, A. W. A. (2011). Description of a new specimen of *Susisuchus anatoceps* (Crocodylomorpha: Mesoeucrocodylia) from the Crato Formation (Santana Group) with comments on Neosuchia. *Zoological Journal of the Linnean Society*, *163*, S273–S288.

Foth, C., Bona, P., & Desojo, J. (2015). Intraspecific variation in the skull morphology of the black caiman *Melanosuchus niger* (Alligatoridae, Caimaninae). *Acta Zoologica*, *96*, 1–13.

Foth, C., Fernandez Blanco, M. V., Bona, P., & Scheyer, T. M. (2018). Cranial shape variation in jacarean caimanines (Crocodylia, Alligatoroidea) and its implications in the taxonomic status of extinct species: The case of *Melanosuchus fisheri*. *Journal of Morphology*, *279*(2), 259–273.

Fourtau, R. (1920). *Contribution à l'étude des vertébrés miocènes de l'Egypte*. Government Press.

Frey, E., & Salisbury, S. W. (2007). Crocodilians of the Crato Formation: evidence for enigmatic species. Pp. 463–476 in D. M. Martill, G. Bechly & R. F. Loveridge (eds.) *The Crato Fossil Beds of Brazil: Window Into an Ancient World*, Cambridge University Press, New York, U. S. A.

Frey, E., Laemmert, A., & Riess, J. (1987). *Baryphracta deponiae* n. g. n. sp. (Reptilia, Crocodylia), ein neues Krokodil aus der Grube Messel bei Darmstadt (Hessen, Bundesrepublik Deutschland). *Neues Jahrbuch für Geologie und Paläontologie, Monatschefte*, *1987*, 15–26.

Fuchs, K. (2006). *Crocodile Skin: Important Characteristics in Identifying Crocodilian Species*. Editions Chimaira, Frankfurt, 175 pp.

Gilmore, C. W. (1910). *Leidyosuchus sternbergii*, a new species of crocodile from the Cretaceous Beds of Wyoming. *Proceedings of the United States National Museum*, *38*, 485–502.

Gilmore, C. W. (1911). A new fossil alligator from the Hell Creek beds of Montana. *Proceedings of the U. S. National Museum*, *41*(1860), 297–301.

Gilmore, C. W. (1946). A new crocodilian from the Eocene of Utah. *Journal of Paleontology*, *20*(1), 62–67.

Ginsburg, L., & Buffetaut, E. (1978). *Euthecodon arambourgi* n. sp., et l'évolution du genre *Euthecodon*, Crocodilien du Néogène d'Afrique. *Géologie Méditerranéenne*, *5*(2), 291–301.

Gmelin, J. F. (1789). *Caroli a Linné systema naturae per regna tri naturae: secundum classes, ordines, genera, species, cum characteribus, differentiis, synonymis, locis. Tomus 1, Pars III. Editio decima tertia, aucta, reformata*, pp. 1057–1058. Georg Emanuel Beer, Leipzig.

Godoy, P. L., Cidade, G. M., Montefeltro, F. C., Langer, M. C., & Norell, M. A. (2021). Redescription and phylogenetic affinities of the caimanine *Eocaiman cavernensis* (Crocodylia, Alligatoroidea) from the Eocene of Argentina. *Papers in Palaeontology*, *7*(3), 1205–1231.

Gold, M. E. L. (2011). *Cranial osteology and braincase morphometrics of* Gavialis gangeticus*: implications for crocodylian phylogenetics*. Unpublished Master of Science thesis, University of Iowa, Iowa City, 103 pp.

Grandidier, A., & Vaillant, L. (1872). Sur le crocodile fossile d'Amboulintsatre (Madagascar). *Comptes Rendus de l'Academie des Sciences de Paris*, *75*, 150–151.

Gratten, J. (2003). The Molecular Systematics, Phylogeography and Population Genetics of Indo-Pacific *Crocodylus*. Unpublished PhD thesis, The University of Queensland, Brisbane, 222 pp.

Graves, M. L. (1819). Sur deux novelles espécies de crocodile. *Annales Générales des Sciences Physiques de Bruxelles*, *2*, 343–353.

Grigg, G., & Kirshner, D. (2015). *Biology and Evolution of Crocodylians*. CSIRO Publishing, Clayton South, 649 pp.

Groh, S. S., Upchurch, P., Barrett, P. M., & Day, J. J. (2020). The phylogenetic relationships of neosuchian crocodiles and their implications for the convergent evolution of the longirostrine condition. *Zoological Journal of the Linnean Society*, *188*(2), 473–506.

Hall, P. M., & Portier, K. M. (1994). Cranial morphometry of New Guinea crocodiles (*Crocodylus novaeguineae*): ontogenetic variation in relative growth of the skull and an assessment of its utility as a predictor of the sex and size of individuals. *Herpetological Monographs*, *8*, 203–225.

Hastings, A. K., Bloch, J. I., Jaramillo, C. A., Rincon, A. F., & Macfadden, B. J. (2013). Systematics and biogeography of crocodylians from the Miocene of Panama. *Journal of Vertebrate Paleontology*, *33*(2), 239–263.

Hecht, M. K. (1987). Fossil snakes and crocodilians from the Sahabi Formation in Libya. Pp. 101–106 in N. T. Boaz, A. El-Arnauti, A. W. Gaziry, J. Heinzelin & D. D. Boaz (eds.) *Neogene paleontology and geology of Sahabi*, Alan R. Liss, New York.

Holliday, C. M., Porter, W. R., Vliet, K. A., & Witmer, L. M. (2020). The frontoparietal fossa and dorsotemporal fenestra of archosaurs and their significance for interpretations of vascular and muscular anatomy in dinosaurs. *The Anatomical Record*, *303*(4), 1060–1074.

Hua, S. & Jouve, S. (2004). A primitive marine gavialoid from the Paleocene of Morocco. *Journal of Vertebrate Paleontology*, *24*(2), 341–350.

Hutchinson, J. R. (2020, March 23). hatchling *Palaeosuchus palpebrosus* RVC-JRH-PP1 whole body. DOI: 10.17605/OSF.IO/UAD97.

Iijima, M. (2017). Assessment of trophic ecomorphology in non-alligatoroid crocodylians and its adaptive and taxonomic implications. *Journal of Anatomy*, *231*(2), 192–211.

Iordansky, N. N. (1964). The jaw muscles of the crocodiles and some relating structures of the crocodilian skull. *Anatomischer Anzeiger*, *115*, 256–280.

Iordansky, N. N. (1973). The skull of the Crocodilia. Pp. 201–262 in C. Gans & T. Parsons (eds.) *Biology of the Reptilia*, *4*, Academic Press, London.

de Iuliis, G. & Pulerà, D. (2011). Reptile skulls and mandibles. Pp. 253–285 in G. de Iuliis & D. Pulerà (eds.) *The Dissection of Vertebrates: A Laboratory Manual (Second Edition)*, Academic Press, Oxford.

Jiménez Fuentes, E. (1983). Algunos restos de craneos de cocodrilo del Paleógeno de Salamanca. *Stvdia Geologica*, *19*, 79–100.

Joffe, J. (1967). The 'dwarf' crocodiles of the Purbeck Formation, Dorset: a reappraisal. *Palaeontology*, *10*(4), 629–639.

Jouve, S. (2004). *Etude des Crocodyliformes fini Crétacé-Paléogène du Bassin des Oulad Abdoun (Maroc) et comparaison avec les faunes africaines contemporaines: systématique, phylogénie et paléobiogéographie*. Unpublished PhD thesis, Muséum National d'Histoire Naturelle, Paris, 651 pp.

Jouve, S. (2016). A new basal tomistomine (Crocodylia, Crocodyloidea) from Issel (Middle Eocene; France): palaeobiogeography of basal tomistomines and palaeogeographic consequences. *Zoological Journal of the Linnean Society*, *177*(1), 165–182.

Jouve, S., Bardet, N., Jalil, N.-E., Pereda Suberbiola, X., Bouya, B., & Amaghzaz, M. (2008). The oldest African crocodylian: phylogeny, paleobiogeography, and differential survivorship of marine reptiles through the Cretaceous-Tertiary boundary. *Journal of Vertebrate Paleontology*, *28*(2), 409–421.

Jouve, S., Bouya, B., Amaghzaz, M., & Meslouh, S. (2015). *Maroccosuchus zennaroi* (Crocodylia: Tomistominae) from the Eocene of Morocco: phylogenetic and palaeobiogeographical implications of the basalmost tomistomine. *Journal of Systematic Palaeontology*, *13*(5), 421–445.

Kälin, J. A. (1936). *Hispanochampsa mülleri* nov. gen. nov. spec., ein neuer Crocodilide aus dem unteren Oligocaen von Tárrega (Catalonien). *Abhandlungen Schweizerischen Palaeontologischen Gesellschaft*, *58*, 1–40.

de Kay, J. E. (1842). *Zoology of New York*. White & Visscher, 415 pp.

Klein, G. F. (2016). *Skeletal anatomy of* Alligator *and comparison with* Thecachampsa. Calvert Marine Museum, Solomons, 70 pp.

Kobatake, N., Chiji, M., Ikebe, N., Ishida, S., Kamei, T., Nakaseko, K., & Matsumoto, E. (1965). *Discovery of a crocodile fossil from the Osaka Group* [in Japanese]. *Quaternary Research* [= *Daiyonki Kenkyu*], *4*(2), 49–58.

Kobayashi, Y., Tomida, Y., Kamei, T., & Eguchi, T. (2006). Anatomy of a Japanese tomistomine crocodylian, *Toyotamaphimeia machikanensis* (Kamei et Matsumoto, 1965), from the

middle Pleistocene of Osaka Prefecture: the reassessment of its phylogenetic status within Crocodylia. *National Science Museum Monographs*, *35*, 1–121.

Kraus, R. (1998). The cranium of *Piscogavialis jugaliperforatus* n. gen., n. sp. (Gavialidae, Crocodylia) from the Miocene of Peru. *Paläontologische Zeitschrift*, *72*(3–4), 389–406.

Krefft, G. (1873). Remarks on Australian crocodiles and description of a new species. *Proceedings of the Zoological Society of London*, *1873*, 334–335.

Kuhn, O. (1938). Die Crocodilier aus dem mittleren Eozän des Geiseltales bei Halle. *Nova Acta Leopoldina*, *39*, 313–328.

Kuzmin, I. T., Boitsova, E. A., Gombolevskiy, V. A., Mazur, E. V., Morozov, S. P., Sennikov, A. G., Skutschas, P. P., & Sues, H.-D. (2021). Braincase anatomy of extant Crocodylia, with new insights into the development and evolution of the neurocranium in crocodylomorphs. *Journal of Anatomy*, *239*(5), 983–1038.

Lambe, L. M. (1907). On a new crocodilian genus and species from the Judith River Formation of Alberta. *Transactions of the Royal Society of Canada, series 3*, *4*, 219–244.

Langston, W. (1965). Fossil crocodilians from Colombia and the Cenozoic history of the Crocodilia in South America. *University of California Publications in Geological Science*, *52*, 1–157.

Langston, W. (1966). *Mourasuchus* Price, *Nettosuchus* Langston, and the family Nettosuchidae (Reptilia: Crocodilia). *Copeia*, *1966*(4), 882–885.

Langston, W. (1975). Ziphodont crocodiles: *Pristichampsus vorax* (Troxell), new combination, from the Eocene of North America. *Fieldiana: Geology*, *33*(16), 291–314.

Langston, W., & Gasparini, Z. (1997). Crocodilians, *Gryposuchus*, and the South American gavials. Pp. 113–154 in R. F. Kay, R. H. Madden, R. L. Cifelli & J. J. Flynn (eds.) *Vertebrate Paleontology in the Neotropics–The Miocene fauna of La Venta, Colombia*, Smithsonian Institution, Washington DC.

Laurenti, J. N. (1768). *Specimen medicum, exhibens synopsin reptilium emendatam cum experimentis circa venena et antidota reptilium austriacorum, quod authoritate et consensu*. Trattnern, Vienna, 217 pp.

Lee, M. S. Y., & Yates, A. M. (2018). Tip-dating and homoplasy: reconciling the shallow molecular divergences of modern gharials with their long fossil record. *Proceedings of the Royal Society B*, *285*(1881), 20181071.

Leidy, J. (1852). Description of a new species of crocodile from the Miocene of Virginia. *Journal of the Academy of Natural Sciences of Philadelphia*, *2*(2), 135–138.

Leite, K. J., & Fortier, D. C. (2018). The palate and choanae structure of the *Susisuchus anatoceps* (Crocodyliformes, Eusuchia): phylogenetic implications. *PeerJ*, *6*, e5372.

Lesson, R. P. (1831). Reptiles. Pp. 291–336, pls. 1-7 in I. G. S. Bélanger (ed.), *Voyage aux Indes-Orientales, par le nord de l'Europe, les provinces du Caucase, la Géorgie, l'Arménie, et la Perse, suivi de détails topographiques, statistiques et autres sur le Pégou, les iles de Java, de Maurice et de Bourbon, sur le cap-de-bonne-espérance et Sainte-Hélen, pendant les années 1825, 1826, 1827, 1828 et 1829. Zoologie*. A. Bertrand, Paris, xxxix + 535 pp.

Li, C., Wu, X.-c., & Rufolo, S. J. (2019). A new crocodyloid (Eusuchia: Crocodylia) from the upper cretaceous of China. *Cretaceous Research*, *94*, 25–39.

Li, J. (1975). New materials of *Tomistoma petrolica* from Maoming, Guangdong. *Vertebrata PalAsiatica*, *13*(3), 190–194.

Li, J. (1976). Fossil of Sebecosuchia discovered from Nanxiong, Guangdong. *Vertebrata PalAsiatica*, *14*(3), 169–173.

Li, J. (1984). A new species of *Planocrania* from Hengdong, Hunan. *Vertebrata PalAsiatica*, *22*(2), 123–133.

Linnaeus, C. (1758). *Systema Naturae per regna tria naturae, secundum classes, ordines, genera, species, cum characteribus, differentiis, synonymis, locis, Tomus I. Editio decima, reformata*. Holmiae, impensis direct. Laurentii Salvii, 1–824.

Loomis, F. B. (1904). Two new river reptiles from the Titanothere beds. *American Journal of Science (ser. 4)*, *18*, 427–432.

Ludwig, R. (1877). Fossile Crocodiliden aus der Tertiärformation des mainzer Beckens. *Palaeontographica Suppl. 3*, 1–52.

Lull, R. S. (1944). Fossil gavials from north India. *American Journal of Science*, *242*(8), 417–430.

Maccagno, A. M. (1948). Descrizione di una nuova specie di "*Crocodilus*" del giacimento di Sahabi (Sirtica). *Atti della Reale Accademia Nazionale dei Lincei: Memorie della Classe di Scienze fisiche, Mathematiche e Naturale, Serie 8*, *1*(2), 63–96.

Marsh, O. C. (1870). Notice of a new species of gavial from the Eocene of New Jersey. *American Journal of Science*, *148*, 97–99.

Marsh, O. C. (1871). Notice of some new fossil reptiles from the Cretaceous and Tertiary formations. *American Journal of Science and Arts (ser. 3) 1*, *6*, 447–459.

Martin, J. E. (2007). New material of the Late Cretaceous globidontan *Acynodon iberoccitanus* (Crocodylia) from southern France. *Journal of Vertebrate Paleontology*, *27*(2), 362–372.

Martin, J. E., & Lauprasert, K. (2010). A new primitive alligatorine from the Eocene of Thailand: relevance of Asiatic members to the radiation of the group. *Zoological Journal of the Linnean Society*, *158*(3), 608–628.

Martin, J. E., Delfino, M., & Smith, T. (2016). Osteology and affinities of Dollo's goniopholidid (Mesoeucrocodylia) from the Early Cretaceous of Bernissart, Belgium. *Journal of Vertebrate Paleontology*, *36*(6), e1222534.

Martin, J. E., Smith, T., Salaviale, C., Adrien, J., & Delfino, M. (2020). Virtual reconstruction of the skull of *Bernissartia fagesii* and current understanding of the neosuchian–eusuchian transition. *Journal of Systematic Palaeontology*, *18*(13), 1079–1101.

Massonne, T., Vasilyan, D., Rabi, M., & Böhme, M. (2019). A new alligatoroid from the Eocene of Vietnam highlights an extinct Asian clade independent from extant *Alligator sinensis*. *PeerJ*, *7*, e7562.

Mateus, O., Puértolas-Pascual, E., & Callapez, P. M. (2019). A new eusuchian crocodylomorph from the Cenomanian (Late Cretaceous) of Portugal reveals novel implications on the origin of Crocodylia. *Zoological Journal of the Linnean Society*, *186*(2), 501–528.

Megirian, D. (1994). A new species of *Quinkana* Molnar (Eusuchia: Crocodylidae) from the Miocene Camfield Beds of northern Australia. *The Beagle, Records of the Museums and Art Galleries of Northern Territory*, *11*, 145–166.

Megirian, D., Murray, P. F., & Willis, P. (1991). A new crocodile of the gavial ecomorph morphology from the Miocene of northern Australia. *The Beagle, Records of the Northern Territory Museum of Arts and Sciences*, *8*(1), 135–158.

Meyer, H. von. (1832). *Paleologica zur Geshichte der Erde und irher Geschöpfe*. S. Schmerber, Frankurt-am-Main, 560 pp.

Molnar, R. E. (1981). Pleistocene ziphodont crocodilians of Queensland. *Records of the Australian Museum*, *33*(19), 803–834.

Molnar, R. E. (1982). *Pallimnarchus* and other Cenozoic crocodiles in Queensland. *Memoirs of the Queensland Museum*, *20*(3), 657–673.

Montefeltro, F. C., Larsson, H. C. E., de França, M. A. G., & Langer, M. C. (2013). A new neosuchian with Asian affinities from the Jurassic of northeastern Brazil. *Naturwissenschaften*, *100*(9), 835–841.

Montefeltro, F. C., Andrade, D. V., & Larsson, H. C. (2016). The evolution of the meatal chamber in crocodyliforms. *Journal of Anatomy*, *228*(5), 838–863.

Mook, C. C. (1921a). Description of a skull of a Bridger crocodilian. *Bulletin of the American Museum of Natural History*, *44*(11), 111–116.

Mook, C. C. (1921b). The skull of *Crocodilus acer* Cope. *Bulletin of the American Museum of Natural History*, *44*(11), 117–121.

Mook, C. C. (1921c). Skull characters of recent Crocodilia, with notes on the affinities of the recent genera. *Bulletin of the American Museum of Natural History*, *44*(11), 123–268.

Mook, C. C. (1923a). A new species of alligator from the Snake Creek Beds. *American Museum Novitates*, *73*, 1–13.

Mook, C. C. (1923b). Skull characters of *Alligator sinense* [sic.] Fauvel. *Bulletin of the American Museum of Natural History*, *48*, 553–562.

Mook, C. C. (1924a). A new crocodilian from Mongolia. *American Museum Novitates*, *117*, 1–5.

Mook, C. C. (1924b). A new crocodilian from the Wasatch Beds. *American Museum Novitates*, *137*, 1–4.

Mook, C. C. (1927). The skull characters of *Crocodilus megarhinus* Andrews. *American Museum Novitates*, *289*, 1–8.

Mook, C. C. (1932). A study of the osteology of *Alligator prenasalis* (Loomis). *Bulletin of the Museum of Comparative Zoology*, *74*(2), 19–41.

Mook, C. C. (1933). A skull with jaws of *Crocodilus sivalensis* Lydekker. *American Museum Novitates*, *670*, 1–10.

Mook, C. C. (1941a). A new crocodilian from the Lance Formation. *American Museum Novitates*, *1128*, 1–5.

Mook, C. C. (1941b). A new crocodilian, *Hassiacosuchus kayi*, from the Bridger Eocene beds of Wyoming. *Annals of the Carnegie Museum*, *28*, 207–220.

Mook, C. C. (1941c). A new fossil crocodilian from Colombia. *Proceedings of the United States National Museum*, *91*(3122), 55–58.

Mook, C. C. (1946). A new Pliocene alligator from Nebraska. *American Museum Novitates*, *1311*, 1–12.

Mook, C. C. (1959). A new species of fossil crocodile of the genus *Leidyosuchus* from the Green River beds. *American Museum Novitates*, *1933*, 1–6.

Mook, C. C. (1961). Notes on the skull characters of *Allognathosuchus polyodon*. *American Museum Novitates*, *2072*, 1–5.

Morgan, G. S., & Albury, N. A. (2013). The Cuban Crocodile (*Crocodylus rhombifer*) from late Quaternary fossil deposits in the Bahamas and Cayman Islands. *Bulletin of the Florida Museum of Natural History*, *52*(3), 162–236.

Morgan, G. S., Albury, N. A., Rímoli, R., Lehman, P., Rosenberger, A. L., & Cooke, S. B. (2018). The Cuban Crocodile (*Crocodylus rhombifer*) from late Quaternary underwater cave deposits in the Dominican Republic. *American Museum Novitates*, *3916*, 1–56.

Morgan, G. S., Franz, R., & Crombie, R. I. (1993). The Cuban Crocodile, *Crocodylus rhombifer*, from Late Quaternary Fossil Deposits on Grand Cayman. *Caribbean Journal of Science*, *29*(3–4), 153–164.

Müller, L. (1927). Ergebnisse der Forschungsreisen Prof. E. Stromers in den Wüsten Ägyptens. V. Tertiäre Wirbeltiere: 1. Beiträge zur Kenntnis der Krokodilier des ägyptischen Tertiärs. *Abhandlungen der Bayerischen Akademie der Wissenschaften, Mathematisch - naturwissenschaftliche Abteilung*, *31*(2), 1–97.

Müller, S. (1838). Waarnemingen over de Indische krokodillen en Beschrijving van eene nieuwe soort. *Tijdschrift voor Natuurlijke Geschiedenis en Physiologie. Amsterdam and Leyden*, *5*, 1–27.

Müller, S., & Schlegel, H. (1844). Over de Krokodillen van den Indischen Archipel. P. 28 in C. J. Temminck, 1839-1844. *Verhandelingen over de natuurlijke geschiedenis der Nederlandsche overzeesche bezittingen, door de leden der Natuurkundige Commissie in Indie en andere Schrijvers*. Leiden, 259 pp.

Myrick, A. C. (2001). *Thecachampsa antiqua* (Leidy, 1852) (Crocodylidae: Thoracosaurinae) from fossil marine deposits at Lee Creek Mine, Aurora, North Carolina, USA. *Smithsonian Contributions to Paleobiology*, *90*, 219–225.

Narváez, I., Brochu, C. A., Escaso, F., Pérez-García, A., & Ortega, F. (2015). New crocodyliforms from southwestern Europe and definition of a diverse clade of European Late Cretaceous basal eusuchians. *PLoS ONE*, *10*(11), e0140679.

Narváez, I., Brochu, C. A., Escaso, F., Pérez-García, A., & Ortega, F. (2016). New Spanish Late Cretaceous eusuchian reveals the synchronic and sympatric presence of two allodaposuchids. *Cretaceous Research*, *65*, 112–125.

Narváez, I., Brochu, C. A., De Celiz, A., Codrea, V., Escaso, F., Pérez-García, A., & Ortega, F. (2020). New diagnosis for *Allodaposuchus precedens*, the type species of the European Upper Cretaceous clade Allodaposuchidae. *Zoological Journal of the Linnean Society*, *189*(2), 618–634.

Nopcsa, F. (1928). Paleontological notes on Reptilia. 7. Classification of the Crocodilia. *Geologica Hungarica, Series Palaeontologica*, *1*(1), 75–84.

Norell, M. A. (1988). *Cladistic approaches to paleobiology as applied to the phylogeny of alligatorids*. Unpublished PhD thesis, Yale University, New Haven, 279 pp.

Norell, M. A. (1989). The higher level relationships of the extant Crocodylia. *Journal of Herpetology*, *23*(4), 325–335.

Norell, M. A., & Clark, J. M. (1990). A reanalysis of *Bernissartia fagesii*, with comments on its phylogenetic position and its bearing on the origin and diagnosis of the Eusuchia. *Bulletin de l'Institut Royal des Sciences Naturelles de Belgique*, *60*, 115–128.

Norell, M. A., & Storrs, G. W. (1989). Catalogue and review of the type fossil crocodilians in the Yale Peabody Museum. *Postilla*, *203*, 1–28.

Norell, M. A., Clark, J. M., & Hutchison, J. H. (1994). The Late Cretaceous alligatoroid *Brachychampsa montana* (Crocodylia): new material and putative relationships. *American Museum Novitates*, *3116*, 1–26.

Ősi, A. (2008). Cranial osteology of *Iharkutosuchus makadii*, a Late Cretaceous basal eusuchian crocodyliform from Hungary. *Neues Jahrbuch für Geologie und Paläontologie-Abhandlungen*, *248*(3), 279–299.

Ősi, A. (2014). The evolution of jaw mechanism and dental function in heterodont crocodyliforms. *Historical Biology: An International Journal of Paleobiology*, *26*(3), 279–414.

Ősi, A., & Weishampel, D. B. (2009). Jaw mechanism and dental function in the Late Cretaceous basal eusuchian *Iharkutosuchus*. *Journal of Morphology*, *270*(8), 903–920.

Ősi, A., Clark, J. M., & Weishampel, D. B. (2007). First report on a new basal eusuchian crocodyliform with multicusped teeth from the Upper Cretaceous (Santonian) of Hungary. *Neues Jahrbuch für Geologie und Paläontologie-Abhandlungen*, *243*(2), 169–177.

Owen, R. (1874). Monograph on the fossil Reptilia of the Wealden and Purbeck Formations. Supplement No. IV (*Hylaeochampsa*). *Palaeontolographical Society, Monographs*, *27*, 1–7.

Owen, R. (1878). On the fossils called "granicones"; being a contribution to the histology of the exo-skeleton in" Reptilia". *Journal of the Royal Microscopical Society*, *1*, 233–236.

Owen, R. (1879). Monograph on the fossil Reptilia of the Wealden and Purbeck Formations. Supplement No. IX. Crocodilia (*Goniopholis*, *Brachydectes*, *Nannosuchus*, *Theriosuchus* and *Nuthetes*). *Palaeontographical Society, Monographs*, *33*, 1–19.

Pickford, M. (2003). A new species of crocodile from early and middle Miocene deposits of the lower Orange River Valley, Namibia, and the origins of the Nile Crocodile (*Crocodylus niloticus*). *Geological Survey of Namibia Memoir*, *19*(2003), 51–65.

Pierce, S. E., Williams, M., & Benson, R. B. J. (2017). Virtual reconstruction of the endocranial anatomy of the early Jurassic marine crocodylomorph *Pelagosaurus typus* (Thalattosuchia). *PeerJ*, *5*, e3225.

Piras, P., & Buscalioni, A. D. (2006). *Diplocynodon muelleri* comb. nov., an Oligocene diplocynodontine alligatoroid from Catalonia (Ebro Basin, Lleida Province, Spain). *Journal of Vertebrate Paleontology*, *26*(3), 608–620.

Piras, P., Delfino, M., Del Favero, L., & Kotsakis, T. (2007). Phylogenetic position of the crocodylian *Megadontosuchus arduini* and tomistomine palaeobiogeography. *Acta Palaeontologica Polonica*, *52*(2), 315–328.

Platt, S. G., Rainwater, T. R., Thorbjarnarson, J. B., Finger, A. G., Anderson, T. A., & McMurry, S. T. (2009). Size estimation, morphometrics, sex ratio, sexual size dimorphism, and biomass of Morelet's crocodile in northern Belize. *Caribbean Journal of Science*, *45*(1), 80–93.

Poe, S. (1996). Data set incongruence and the phylogeny of crocodilians. *Systematic Biology*, *45*(4), 393–414.

Pol, D., & Norell, M. A. (2004). A new gobiosuchid crocodyliform taxon from the Cretaceous of Mongolia. *American Museum Novitates*, *3458*, 1–31.

Pol, D., Turner, A. H., & Norell, M. A. (2009). Morphology of the Late cretaceous crocodylomorph *Shamosuchus djadochtaensis* and a discussion of neosuchian phylogeny as related to the origin of Eusuchia. *Bulletin of the American Museum of Natural History*, *324*, 1–103.

Pomel, A. (1847). Note sur les animaux fossiles découverts dans le département de l'Allier. *Bulletin de la Societé Geologique de France, serie 2*, *4*, 378–385.

Porter, W. R., Sedlmayr, J. C., & Witmer, L. M. (2016). Vascular patterns in the heads of crocodilians: blood vessels and sites of thermal exchange. *Journal of Anatomy*, *229*(6), 800–824.

Puértolas-Pascual, E., Canudo, J. I., & Cruzado-Caballero, P. (2011). A new crocodylian from the late Maastrichtian of Spain: implications for the initial radiation of crocodyloids. *PLoS ONE*, *6*(6), e20011.

Puértolas-Pascual, E., Canudo, J. I., & Moreno-Azanza, M. (2014). The eusuchian crocodylomorph *Allodaposuchus subjuniperus* sp. nov., a new species from the latest Cretaceous (Upper Maastrichtian) of Spain. *Historical Biology: An International Journal of Paleobiology*, *26*(1), 91–109.

Richardson, K. C., Webb, G. J. W., & Manolis, S. C. (2002). *Crocodiles: Inside Out*. Surrey Beatty & Sons, Sydney, 172 pp.

Rio, J. P., & Mannion, P. D. (2021). Phylogenetic analysis of a new morphological dataset elucidates the evolutionary history of Crocodylia and resolves the long-standing gharial problem. *PeerJ*, *9*, e12094.

Rio, J. P., Mannion, P. D., Tschopp, E., Martin, J. E., & Delfino, M. (2020). Reappraisal of the morphology and phylogenetic relationships of the alligatoroid crocodylian *Diplocynodon hantoniensis* from the late Eocene of the United Kingdom. *Zoological Journal of the Linnean Society*, *188*(2), 579–629.

Ristevski, J., Young, M. T., de Andrade, M. B., & Hastings, A. K. (2018). A new species of *Anteophthalmosuchus* (Crocodylomorpha, Goniopholididae) from the Lower Cretaceous of the Isle of Wight, United Kingdom, and a review of the genus. *Cretaceous Research*, *84*, 340–383.

Ristevski, J., Yates, A. M., Price, G. J., Molnar, R. E., Weisbecker, V., & Salisbury, S. W. (2020a). Australia's prehistoric 'swamp king': revision of the Plio-Pleistocene crocodylian genus *Pallimnarchus* de Vis, 1886. *PeerJ*, *8*, e10466.

Ristevski, J., Yates, A. M., Price, G. J., Molnar, R. E., Weisbecker, V., & Salisbury, S. W. (2020b). Data from: Australia's prehistoric 'swamp king': revision of the Plio-Pleistocene crocodylian genus *Pallimnarchus* de Vis, 1886. *Dryad, Dataset*. DOI: 10.5061/dryad.8kprr4xkq.

Ristevski, J., Price, G. J., Weisbecker, V., & Salisbury, S. W. (2021). First record of a tomistomine crocodylian from Australia. *Scientific Reports*, *11*, 12158.

Rogers, J. V. II. (2003). *Pachycheilosuchus trinquei*, a new procoelous crocodyliform from the Lower Cretaceous (Albian) Glen Rose Formation of Texas. *Journal of Vertebrate Paleontology*, *23*(1), 128–145.

Ross, C. A. (1990). *Crocodylus raninus* S. Müller and Schlegel, a valid species of crocodile (Reptilia: Crocodylidae) from Borneo. *Proceedings of the Biological Society of Washington*, *103*(4), 955–961.

Rossmann, T. (2000). Skelettanatomische Beschreibung von *Pristichampsus rollinatii* (Gray) (Crocodilia, Eusuchia) aus dem Paläogen von Europa, Nordamerika und Ostasien. *Courier Forschungsinstitut Senckenberg*, *221*, 1–107.

Rovereto, C. (1912). Los cocodrilos fósiles en las capas de Paraná. *Anales de Museo Nacional de Buenos Aires ser. 3*, *22*, 339–369.

Salas-Gismondi, R., Flynn, J. J., Baby, P., Tejada-Lara, J. V., Claude, J., & Antoine, P.-O. (2016). A new 13 million year old gavialoid crocodylian from proto-Amazonian mega-wetlands reveals parallel evolutionary trends in skull shape linked to longirostry. *PLoS ONE*, *11*(4), e0152453.

Salisbury, S. W. (2001). *A biomechanical transformation model for the evolution of the eusuchian-type bracing system*. Unpublished PhD thesis, University of New South Wales, Sydney, 554 pp.

Salisbury, S. W. (2002). Crocodilians from the Lower Cretaceous (Berriasian) Purbeck Limestone Group of Dorset, Southern England. *Special Papers in Palaeontology*, *68*, 121–144.

Salisbury, S. W., & Naish, D. (2011). Crocodilians. Pp. 305–369 in D. J. Batten DJ (ed.) *English Wealden Fossils*, The Palaeontological Association, London.

Salisbury, S. W., & Willis, P. M. A. (1996). A new crocodylian from the Early Eocene of south-eastern Queensland and a preliminary investigation of the phylogenetic relationships of crocodyloids. *Alcheringa: An Australasian Journal of Palaeontology*, *20*(3),179–226.

Salisbury, S. W., Frey, E., Martill, D. M., & Buchy, M. C. (2003). A new crocodilian from the Lower Cretaceous Crato formation of northeastern Brazil. *Paläontographica*, *270*(1), 3–47.

Salisbury, S. W., Molnar, R. E., Frey, E., & Willis, P. M. A. (2006). The origin of modern crocodyliforms: new evidence from the Cretaceous of Australia. *Proceedings of the Royal Society B: Biological Sciences*, *273*(1600), 2439–2448.

Scheyer, T. M., Aguilera, O. A., Delfino, M., Fortier, D. C., Carlini, A. A., Sánchez, R., Carrillo-Briceño, J. D., Quiroz, L., & Sánchez-Villagra, M. R. (2013). Crocodylian diversity peak and extinction in the late Cenozoic of the northern Neotropics. *Nature Communications*, *4*, 1907.

Schmidt, K. P. (1919). Contributions to the herpetology of the Belgian Congo based on the collection of the American Museum Congo expedition, 1909–1915. Part 1. Turtles, crocodiles, lizards and chameleons. *Bulletin of the American Museum of Natural History*, *39*(2), 385–624.

Schmidt, K. P. (1928). A new crocodile from New Guinea. *Zoological Series of the Field Museum of Natural History*, *12*(14), 175–181.

Schmidt, K. P. (1935). A new crocodile from the Philippine Islands. *Zoological Series of the Field Museum of Natural History*, *20*(8), 67–70.

Schmidt, K. P. (1938). New crocodilians from the upper Paleocene of western Colorado. *Geological Series of the Field Museum of Natural History*, *6*(21), 315–321.

Schmidt, K. P. (1941). A new fossil alligator from Nebraska. *Fieldiana: Geology*, *8*, 27–32.

Schneider, J. G. (1801). *Historiae amphibiorum naturalis et literariae. Fasciculus secundus continens Crocodilos, Scincos, Chamaesauras, Boas. Pseudoboas, Elapes, Angues, Amphisbaenas et Caecilias*. Friedrich Frommann, Jena, 365 pp.

Schwarz, D., & Salisbury, S. W. (2005). A new species of *Theriosuchus* (Atoposauridae, Crocodylomorpha) from the Late Jurassic (Kimmeridgian) of Guimarota, Portugal. *Geobios*, *38*(6), 779–802.

Schwarz, D., Raddatz, M., & Wings, O. (2017). *Knoetschkesuchus langenbergensis* gen. nov. sp. nov., a new atoposaurid crocodyliform from the Upper Jurassic Langenberg Quarry (Lower Saxony, northwestern Germany), and its relationships to *Theriosuchus*. *PLoS ONE*, *12*(2), e0160617.

Serrano-Martínez, A., Knoll, F., Narváez, I., Lautenschlager, S., & Ortega, F. (2019a). Brain and pneumatic cavities of the braincase of the basal alligatoroid *Diplocynodon tormis* (Eocene, Spain). *Journal of Vertebrate Paleontology*, *39*(1), e1572612.

Serrano-Martínez, A., Knoll, F., Narváez, I., Lautenschlager, S., & Ortega, F. (2019b). Inner skull cavities of the basal eusuchian *Lohuecosuchus megadontos* (Upper Cretaceous, Spain) and neurosensorial implications. *Cretaceous Research*, *93*, 66–77.

Serrano-Martínez, A., Knoll, F., Narváez, I., Lautenschlager, S., & Ortega, F. (2021). Neuroanatomical and neurosensorial analysis of the Late Cretaceous basal eusuchian *Agaresuchus fontisensis* (Cuenca, Spain). *Papers in Palaeontology*, *7*(1), 641–656.

Shan, H.-y., Wu, X.-c., Cheng, Y.-n., & Sato, T. (2009). A new tomistomine (Crocodylia) from the Miocene of Taiwan. *Canadian Journal of Earth Sciences*, *46*(7), 529–555.

Shan, H.-Y., Wu, X.-C., Cheng, Y.-N., & Sato, T. (2017). *Maomingosuchus petrolica*, a restudy of '*Tomistoma*' *petrolica* Yeh, 1958. *Palaeoworld*, *26*(4), 672–690.

Shan, H.-y., Wu, X.-C., Sato, T., Cheng, Y.-n., & Rufolo, S. (2021). A new alligatoroid (Eusuchia, Crocodylia) from the Eocene of China and its implications for the relationships of Orientalosuchina. *Journal of Paleontology*, *95*(6), 1321–1339.

Simpson, G. G. (1930). *Allognathosuchus mooki*, a new crocodile from the Puerco Formation. *American Museum Novitates*, *445*, 1–16.

Simpson, G. G. (1933). A new crocodilian from the *Notostylops* beds of Patagonia. *American Museum Novitates*, *623*, 1–9.

Sookias, R. B. (2019, October 30). Exploring the effects of character construction and choice, outgroups, and analytical method on phylogenetic inference from discrete characters in extant crocodilians. DOI: 10.17605/OSF.IO/MGH48.

Sookias, R. B. (2020). Exploring the effects of character construction and choice, outgroups and analytical method on phylogenetic inference from discrete characters in extant crocodilians. *Zoological Journal of the Linnean Society*, *189*(2), 670–699.

Spix, J. B. (1825). *Animalia nova sive species novae lacertarum quas in itinere per Brasiliam annis MDCCCXVII–MDCCCXX jussu et auspiciis Maximiliani Josephi I. Bavariae Regis suscepto collegit et descripsit Dr. J. B. de Spix*. T. O. Weigel, Leipzig, 26 pp.

de Stefano, G. (1905). Appunti sui Batraci e rettili del Quercy appartenenti alla collezioni Rossignol. *Bolletino della Societa Geologia Italiana*, *24*, 17–67.

Sternberg, C. M. (1932). A new fossil crocodile from Saskatchewan. *The Canadian Field-Naturalist*, *44*, 128–133.

Sullivan, R. M., & Lucas, S. G. (2003). *Brachychampsa montana* Gilmore (Crocodylia, Alligatoroidea) from the Kirtland Formation (Upper Campanian), San Juan Basin, New Mexico. *Journal of Vertebrate Paleontology*, *23*(4), 832–841.

Syme, C. E., & Salisbury, S. W. (2018). Taphonomy of *Isisfordia duncani* specimens from the Lower Cretaceous (upper Albian) portion of the Winton Formation, Isisford, central-west Queensland. *Royal Society Open Science*, *5*(3), 171651.

Tarsitano, S. F., Frey, E., & Riess, J. (1989). The evolution of the Crocodilia: a conflict between morphological and biochemical data. *American Zoologist*, *29*(3), 843–856.

Tchernov, E., & van Couvering, J. (1978). New crocodiles from the early Miocene of Kenya. *Palaeontology*, *21*(4), 857–867.

Tennant, J. P., Mannion, P. D., & Upchurch, P. (2016). Evolutionary relationships and systematics of Atoposauridae (Crocodylomorpha: Neosuchia): implications for the rise of Eusuchia. *Zoological Journal of the Linnean Society*, *177*(4), 854–936.

Toula, F., & Kail, J. A. (1885). Über einen Krokodil–Schädel aus den Tertiärablagerungen von Eggenburg in Niederösterreich: eine paläontologische studie. *Denkschriften der Kaiserlichen Akademie der Wissenschaften von Wien, Mathematisch–naturwissenschaftliche Classe*, *50*, 299–355.

Troxell, E. L. (1925). The Bridger crocodiles. *American Journal of Science*, 5th Ser., *9*, 29–72.

Turner, A. H. (2015). A review of *Shamosuchus* and *Paralligator* (Crocodyliformes, Neosuchia) from the Cretaceous of Asia. *PLoS ONE*, *10*(2), e0118116.

Walter, J., Darlim, G., Massonne, T., Aase, A., Frey, E., & Rabi, M. (2022). On the origin of Caimaninae: insights from new fossils of *Tsoabichi greenriverensis* and a review of the evidence. *Historical Biology*, *34*(4), 580–595.

Wang Y.-y., Sullivan, C., & Liu, J. (2016). Taxonomic revision of *Eoalligator* (Crocodylia, Brevirostres) and the paleogeographic origins of the Chinese alligatoroids. *PeerJ*, *4*(5562), e2356.

Weitzel, K. (1935). *Hassiacosuchus haupti* n.g. n.sp., ein durophages Krokodil aus dem Mitteleozän von Messel. *Notizblatt des Vereins für Erdkunde und der Hessischen Geologischen Landesanstalt Darmstadt*, *16*, 40–49.

Wermuth, H. (1953). Systematik der rezenten Krokodile. *Mitteilungen aus dem Museum für Naturkunde in Berlin. Zoologisches Museum und Institut für Spezielle Zoologie (Berlin)*, *29*(2), 375–511.

White, T. E. (1942). A new alligator from the Miocene of Florida. *Copeia*, *1*, 3–7.

Williamson, T. E. (1996). ?*Brachychampsa sealeyi*, sp. nov., (Crocodylia, Alligatoroidea) from the Upper Cretaceous (lower Campanian) Menefee Formation, northwestern New Mexico. *Journal of Vertebrate Paleontology*, *16*(3), 421–431.

Willis, P. M. A. (1993). *Trilophosuchus rackhami* gen. et sp. nov., a new crocodilian from the early Miocene limestones of Riversleigh, northwestern Queensland. *Journal of Vertebrate Paleontology*, *13*(1), 90–98.

Willis, P. M. A. (1997). New crocodilians from the late Oligocene White Hunter Site, Riversleigh, northwestern Queensland. *Memoirs of the Queensland Museum*, *41*(2), 423–438.

Willis, P. M. A. (2001). New crocodilian material from the Miocene of Riversleigh (northwestern Queensland, Australia). Pp. 64–74 in G. Grigg, F. Seebacher & C. E. Franklin (eds.) *Crocodilian Biology and Evolution*, Surrey Beatty & Sons, Sydney, Australia.

Willis, P. M. A., & Molnar, R. E. (1991). A new Middle Tertiary crocodile from Lake Palankarinna, South Australia. *Records of the South Australian Museum*, *25*(1), 39–55.

Willis, P. M. A., & Molnar, R. E. (1997). A review of the Plio–Pleistocene crocodilian genus *Pallimnarchus*. *Proceedings of the Linnean Society of New South Wales*, *117*, 223–242.

Willis, P. M. A., Molnar, R. E., & Scanlon, J. D. (1993). An early Eocene crocodilian from Murgon, Southeastern Queensland. *Kaupia*, *3*, 27–33.

Willis, P., Murray, P., & Megirian, D. (1990). *Baru darrowi* gen. et sp. nov., a large broad-snouted crocodyline (Eusuchia: Crocodilidae) from mid-Tertiary freshwater limestones in northern Australia. *Memoirs of the Queensland Museum*, *29*(2), 521–540.

Willis, P. M. A., Robinson, J., & Kemp, A. (1995). Computerised tomographic scans of an Eocene crocodile skull from southeast Queensland. *Memoirs of the Association of Australasian Palaeontologists*, *18*, 203–208.

Witmer, L. M. (1997). The evolution of the antorbital cavity of archosaurs: a study in soft-tissue reconstruction in the fossil record with an analysis of the function of pneumaticity. *Journal of Vertebrate Paleontology*, *17*(S1), 1–73.

Wood, S. V. (1846). On the discovery of an alligator and of several new Mammalia in the Hordwell Cliff; with observations upon the geological phenomena of that locality. *London Geological Journal*, *1*, 117–122.

Wu, X. C., & Sues H.-D. (1996). Anatomy and phylogenetic relationships of *Chimaerasuchus paradoxus*, an unusual crocodyliform reptile from the Lower Cretaceous of Hubei, China. *Journal of Vertebrate Paleontology*, *16*(4), 688–702.

Wu, X.-C., Brinkman, D. B., & Russell, A. P. (1996). A new alligator from the Upper Cretaceous of Canada and the relationship of early eusuchians. *Palaeontology*, *39*(2), 351–375.

Wu, X.-C., Russell, A. P., & Brinkman, D. B. (2001a). A review of *Leidyosuchus canadensis* Lambe, 1907 (Archosauria: Crocodylia) and an assessment of cranial variation based upon new material. *Canadian Journal of Earth Sciences*, *38*, 1665–1687.

Wu, X. C., Russell, A. P., & Cumbaa, S. L. (2001b). *Terminonaris* (Archosauria: Crocodyliformes): new material from Saskatchewan, Canada, and comments on its phylogenetic relationships. *Journal of Vertebrate Paleontology*, *21*(3), 492-514.

Yates, A. M. (2017). The biochronology and palaeobiogeography of *Baru* (Crocodylia: Mekosuchinae) based on new specimens from the Northern Territory and Queensland, Australia. *PeerJ*, *5*, e3458.

Yates, A. M., & Pledge, N. S. (2016). A Pliocene mekosuchine (Eusuchia: Crocodilia) from the Lake Eyre Basin of South Australia. *Journal of Vertebrate Paleontology*, *37*(1), e1244540.

Yeh, H. (1958). A new crocodile from Maoming, Kwangtung. *Vertebrata PalAsiatica*, *2*(4), 237–242.

Young, C. C. (1964). New fossil crocodiles from China. *Vertebrata Palasiatica*, *8*(2), 189–208.

Young, C. C. (1982). *A Cenozoic crocodile from Huaining, Anhui. Selected works of Yang Zhongjian*. China: Academia Sinica, 47–48.

Young, M. T. (2014). Filling the 'Corallian Gap': re-description of a metriorhynchid crocodylomorph from the Oxfordian (Late Jurassic) of Headington, England. *Historical Biology: An International Journal of Paleobiology*, *26*(1), 80–90.

Young, B. A., & Bierman, H. S. (2019). On the median pharyngeal valve of the American alligator (*Alligator mississippiensis*). *Journal of Morphology*, *280*(1), 58–67.

Zangerl, R. (1944). *Brachyuranochampsa eversolei*, gen. et sp. nov., a new crocodilian from the Washakie Eocene of Wyoming. *Annals of the Carnegie Museum*, *30*, 77–84.

de Zigno, A. (1880). Sopra un cranio di coccodrillo scoperto nel terreno eoceno del Veronese. *Atti della Reale Accademia Lincei, Memorie della Classe di Scienze Fisiche, Matematiche e Naturali, Serie 3*, *5*, 65–72.
